# Efficacy and Safety of 2 Fingolimod Doses vs Glatiramer Acetate for the Treatment of Patients With Relapsing-Remitting Multiple Sclerosis: A Randomized Clinical Trial

**DOI:** 10.1001/jamaneurol.2020.2950

**Published:** 2020-08-24

**Authors:** Bruce A. C. Cree, Myla D. Goldman, John R. Corboy, Barry A. Singer, Edward J. Fox, Douglas L. Arnold, Corey Ford, Bianca Weinstock-Guttman, Amit Bar-Or, Susanne Mientus, Daniel Sienkiewicz, Ying Zhang, Rajesh Karan, Nadia Tenenbaum

**Affiliations:** 1 UCSF Weill Institute for Neurosciences, Department of Neurology, University of California, San Francisco, San Francisco; 2 Department of Neurology, Virginia Commonwealth University, Richmond; 3 Rocky Mountain Multiple Sclerosis Center, University of Colorado, Aurora; 4 The Multiple Sclerosis Center for Innovations in Care, Missouri Baptist Medical Center, St Louis; 5 Central Texas Neurology Consultants, Round Rock; 6 Montreal Neurological Institute, McGill University, Montreal, Quebec, Canada; 7 University of New Mexico, Albuquerque; 8 Jacobs School of Medicine and Biomedical Sciences, State University of New York at Buffalo; 9 Perelman School of Medicine, Department of Neurology, University of Pennsylvania, Philadelphia; 10 Novartis Pharma AG, Basel, Switzerland; 11 Novartis Pharmaceuticals Corporation, East Hanover, New Jersey

## Abstract

**Importance:**

Doses of fingolimod lower than 0.5 mg per day were not investigated during the fingolimod clinical development program. Whether lower doses of fingolimod might retain efficacy with fewer safety risks remains unknown.

**Objective:**

To evaluate the efficacy and safety of fingolimod, 0.5 mg, and fingolimod, 0.25 mg, compared with glatiramer acetate and to assess whether these doses of fingolimod show superior efficacy to glatiramer acetate in adult patients with relapsing-remitting multiple sclerosis.

**Interventions:**

Fingolimod, 0.5 mg, or fingolimod, 0.25 mg, orally once per day or glatiramer acetate, 20 mg, subcutaneously once per day.

**Design, Setting, and Participants:**

The Multiple Sclerosis Study Evaluating Safety and Efficacy of Two Doses of Fingolimod Versus Copaxone (ASSESS) was a phase 3b multicenter randomized rater-blinded and dose-blinded 12-month clinical trial conducted between August 9, 2012, and April 30, 2018 (including the time required to recruit participants). A total of 1461 patients aged 18 to 65 years with relapsing-remitting multiple sclerosis were screened, and 1064 participants were randomized. These participants had at least 1 documented relapse during the previous year or 2 documented relapses during the previous 2 years and an Expanded Disability Status Scale score of 0 to 6 at screening. Data were analyzed between September and November 2018.

**Main Outcomes and Measures:**

The superiority of the fingolimod doses was tested hierarchically, with fingolimod, 0.5 mg, vs glatiramer acetate, 20 mg, tested first, followed by fingolimod, 0.25 mg, vs glatiramer acetate, 20 mg. The primary end point was the reduction in annualized relapse rate (ARR). Magnetic resonance imaging parameters, safety, and tolerability were also assessed.

**Results:**

Of 1461 adult patients screened, 1064 participants (72.8%) were randomized (mean [SD] age, 39.6 [11.0] years; 792 women [74.4%]) to 3 treatment groups: 352 participants received fingolimod, 0.5 mg, 370 participants received fingolimod, 0.25 mg, and 342 participants received glatiramer acetate, 20 mg. In total, 859 participants (80.7%) completed the study. Treatment with fingolimod, 0.5 mg, was superior to treatment with glatiramer acetate, 20 mg, in reducing ARR (40.7% relative reduction); the relative reduction with fingolimod, 0.25 mg, was 14.6%, which was not statistically significant (for fingolimod, 0.5 mg, ARR, 0.15; 95% CI, 0.11-0.21; for fingolimod, 0.25 mg, ARR, 0.22; 95% CI, 0.17-0.29; for glatiramer acetate, 20 mg, ARR, 0.26; 95% CI, 0.20-0.34). Treatment with both fingolimod doses (0.5 mg and 0.25 mg) significantly reduced new or newly enlarging T2 and gadolinium-enhancing T1 lesions compared with treatment with glatiramer acetate. Adverse events were reported in similar proportions across treatment groups (312 participants [90.4%] in the fingolimod, 0.5 mg, group, 323 participants [88.3%] in the fingolimod, 0.25 mg, group, and 283 participants [87.3%] in the glatiramer acetate group).

**Conclusions and Relevance:**

Fingolimod, 0.5 mg, demonstrated superior clinical efficacy compared with glatiramer acetate, 20 mg, and had a superior benefit-risk profile compared with fingolimod, 0.25 mg, in adult participants with relapsing-remitting multiple sclerosis.

**Trial Registration:**

ClinicalTrials.gov Identifier: NCT01633112

## Key Points

**Question:**

Is treatment with a fingolimod dose of 0.5 mg or 0.25 mg superior to glatiramer acetate, 20 mg, in reducing relapse activity over 12 months in adult participants aged 18 to 65 years with relapsing-remitting multiple sclerosis?

**Findings:**

In this randomized clinical trial of 1064 adult participants with relapsing-remitting multiple sclerosis, treatment with fingolimod, 0.5 mg, reduced the annualized relapse rate by 41%, and treatment with fingolimod, 0.25 mg, reduced the annualized relapse rate by 15% compared with glatiramer acetate. The safety of fingolimod therapy was consistent with the established safety profile.

**Meaning:**

The study results suggested that fingolimod, 0.5 mg, demonstrated superior efficacy, confirming 0.5 mg to be the optimal dose for the treatment of patients with relapsing-remitting multiple sclerosis.

## Teaser

This randomized clinical trial evaluates the efficacy and safety of fingolimod, 0.5 mg, and fingolimod, 0.25 mg, compared with glatiramer acetate, 20 mg, and examines whether these fingolimod doses show superior efficacy to glatiramer acetate for the treatment of adult patients with relapsing-remitting multiple sclerosis.

## Introduction

After the introduction of interferon beta-1b in 1993,^1^ multiple therapies with differing efficacy and safety profiles were approved for relapsing forms of multiple sclerosis (MS).^2,3^ Placebo-controlled clinical trials led to regulatory approval of many therapies, resulting in a substantial change in the treatment of patients with relapsing MS. However, placebo-controlled studies do not provide data regarding the relative merits of different treatments. Head-to-head clinical trials directly compare the efficacy and safety of different interventions and are particularly helpful in therapeutic decision-making. Several studies have demonstrated the superiority of certain disease-modifying therapies, including ofatumumab vs teriflunomide,^4^ ocrelizumab vs subcutaneous interferon beta-1a,^5^ alemtuzumab vs subcutaneous interferon beta-1a,^6^ fingolimod vs intramuscular interferon beta-1a,^7^ and glatiramer acetate vs intramuscular interferon beta-1a.^8^ In contrast, some clinical trials have not shown the superiority of 1 treatment compared with another.^9,10,11^ Glatiramer acetate has not been directly compared with orally bioavailable or monoclonal antibody–based treatments in studies designed to assess superiority.

Fingolimod, 0.5 mg, a sphingosine 1-phosphate receptor modulator, was the first oral disease-modifying therapy approved for the treatment of patients with relapsing forms of MS. In the clinical trials of fingolimod,^7,12,13,14^ the annualized relapse rate (ARR) was reduced by a range of 48% to 55% with once-daily doses of 5 mg, 1.25 mg, and 0.5 mg vs placebo or interferon, suggested that a dose lower than 0.5 mg could be efficacious. In addition, a lower fingolimod dose might have fewer adverse effects than the 0.5-mg dose. Thus, the US Food and Drug Administration recommended evaluation of a dose lower than 0.5 mg.^15^ Based on pharmacokinetic-pharmacodynamic models, treatment with once-daily fingolimod, 0.25 mg, was selected as the lowest dose likely to be efficacious.

The Multiple Sclerosis Study Evaluating Safety and Efficacy of Two Doses of Fingolimod Versus Copaxone (ASSESS) was conducted to address this postapproval commitment with the US Food and Drug Administration to investigate the efficacy and safety of fingolimod, 0.25 mg, for the treatment of patients with relapsing-remitting MS. Treatment with once-daily glatiramer acetate, 20 mg (the thrice-weekly 40 mg dose was not available when the study was initiated) was chosen as a reasonable direct comparator for both fingolimod 0.25 and 0.5 mg doses because of its widespread use as an effective treatment for patients with MS and an understanding that a placebo-controlled clinical trial with a commercial product lacked equipoise. The primary results of the ASSESS clinical trial are presented in this article.

## Methods

### Study Design and Oversight

The ASSESS study was a phase 3b multicenter randomized parallel-group rater-blinded and dose-blinded 12-month clinical trial conducted between August 9, 2012, and April 30, 2018 (including the time required to recruit participants). The trial protocol is available in Supplement 1. A total of 127 sites in Argentina (2 sites), Brazil (8 sites), Canada (7 sites), Chile (2 sites), Mexico (8 sites), and the US (100 sites) participated. The study included a screening phase of up to 45 days before randomization and a 12-month treatment phase followed by a 3-month posttreatment follow-up period. Eligible adult participants were randomized (1:1:1) to receive fingolimod, 0.5 mg, or fingolimod, 0.25 mg, orally once per day or glatiramer acetate, 20 mg, subcutaneously once per day using an interactive voice response system (eFigure in Supplement 2). Participant scores from the Expanded Disability Status Scale (EDSS; the scale quantifies the severity and progression of MS; score range, 20 half-steps from 0-10 points, with higher scores indicating more severe disability) were evaluated by an independent Neurostatus-certified rater who was blinded to the clinical data. Data from magnetic resonance imaging (MRI) scans were analyzed independently by a blinded reader (D.L.A.) at a central reading site (NeuroRX Research).

The ASSESS clinical trial adhered to the International Council for Harmonisation *Guideline for Good Clinical Practice*^16^ and the Declaration of Helsinki.^17^ The study protocol was approved by all institutional review boards or ethics committees for each site (Supplement 1). All participants provided written informed consent before the study began. This study followed the Consolidated Standards of Reporting Trials (CONSORT) reporting guideline for randomized clinical trials.

Eligible participants were between ages 18 and 65 years, had received a diagnosis of relapsing-remitting MS,^18^ had experienced at least 1 documented relapse during the previous year or 2 documented relapses during the previous 2 years before randomization, had received an EDSS score between 0 and 6.0 points (both inclusive) at screening, and had not experienced a relapse within 30 days of randomization. Detailed exclusion criteria are presented in the eMethods in Supplement 2.

### Study Procedures and End Points

The primary objective was to demonstrate the superiority of at least 1 dose of fingolimod (0.5 mg or 0.25 mg) vs glatiramer acetate, 20 mg, for reducing the ARR (based on confirmed relapses, which were verified by the examining neurologist within 7 days after the onset of symptoms). To constitute a confirmed relapse, the symptoms must have been accompanied by an increase of at least 0.5 points in the EDSS score, an increase of 1 point on 2 different functional system scores, or an increase of 2 points on one of the functional system scores (excluding the bowel-bladder or cerebral functional systems portion of the EDSS) up to 12 months. Secondary end points included the number of new or newly enlarging T2 lesions, the number of gadolinium-enhancing T1 lesions and the proportion of participants free from these lesions, the volume of gadolinium-enhancing T1 lesions, the change from baseline in T2 lesion volume, the T1 hypointense lesion volume and brain volume, the change from baseline in the Treatment Satisfaction Questionnaire for Medication (which measures patient treatment satisfaction using 14 items that address 4 domains [effectiveness, side effects, convenience, and global satisfaction]; the 4 summed domain scores are transformed to a scale of 0–100, with higher scores indicating greater satisfaction), and safety and tolerability up to month 12.

Exploratory end points included scores from the Multiple Sclerosis Functional Composite (which includes 3 measures [a timed 25-foot walk, a 9-hole peg test, and the Paced Auditory Serial Addition Test 3], with scores from each measure transformed into *z* scores that are averaged to obtain a composite score; higher *z* scores indicate improvement in disability), the Symbol Digit Modalities Test (which asks patients to voice the digit associated with each of 9 symbols as rapidly as possible within 90 seconds; scoring is based on the total number correct, with higher scores indicating greater cognitive performance), the Patient-Reported Indices in Multiple Sclerosis–Activities scale (which asks patients to rate their ability to perform 15 tasks using a scale of 1-3; score range, 0-30, with higher scores indicating lower quality of health), and the 29-item Multiple Sclerosis Impact Scale (which includes 2 domains, physical and psychological; the 2 domain summary scores are calculated by summing individual items, which are then transformed to a scale of 0-100, with higher scores indicating worse health) as well as the proportion of relapse-free participants, the time to first relapse, and the severity of relapses (Supplement 1; eMethods in Supplement 2).

### Study Hypothesis and Testing

For each of the 2 doses of fingolimod, the null hypothesis that no difference in ARR would be found between participants who received fingolimod therapy and those who received glatiramer acetate therapy could be rejected if the observed significance for the between-treatment comparison was *P* < .05 (after adjustment for multiplicity). The 2 doses of fingolimod were tested against glatiramer acetate, 20 mg, hierarchically through a step-down procedure (ie, the initially approved fingolimod, 0.5 mg, dose was tested against glatiramer acetate, 20 mg, at an unpaired 2-sided significance level of *P* = .05). If this test result was rejected, the lower dose of fingolimod (0.25 mg) was tested against glatiramer acetate, 20 mg (at an unpaired 2-sided significance level of *P* = .05). The study was not designed to detect a difference in treatment effect between the 2 doses of fingolimod. Nominal *P* values were provided for secondary efficacy analyses to assist the evaluation of treatment effect even if the primary efficacy end point did not reach statistically significant differences based on a predefined hierarchical testing procedure. However, the interpretation of results in assessing treatment effect in these cases should be made with caution.

### Statistical Analysis

Sample size and power calculations were based on simulations from a negative binomial distribution with a constant dispersion parameter K. An initial sample of 2550 participants was planned, allowing for a comparison between fingolimod, 0.5 mg, and glatiramer acetate that would have more than 95% power to detect an estimated ARR reduction of 35% and for a comparison between fingolimod, 0.25 mg, and glatiramer acetate that would have 80% power to detect an estimated ARR reduction of 25% (based on pharmacokinetic and pharmacodynamic data) at an unpaired 2-sided significance level of *P* = .05. After experiencing challenges in participant recruitment and in agreement with the Food and Drug Administration in 2014, the sample was reduced to 1960 participants. Because recruitment occurred more slowly than expected, participant recruitment was terminated in January 2017, with 1064 participants randomized. Based on the final sample, the statistical power was reduced to 71% for the comparison between fingolimod, 0.5 mg, and glatiramer acetate and to 44% for the comparison between fingolimod, 0.25 mg, and glatiramer acetate.

All efficacy analyses were conducted in the full analysis set, which comprised all randomized participants who received at least 1 dose of the study drug. All efficacy analyses used the actual study time rather than derived windows. The primary end point, ARR, was tested using a negative binomial regression model with a log link, with treatment as the main effect and the number of relapses in the previous year before study enrollment, the baseline EDSS score, and the baseline number of gadolinium-enhancing T1 lesions as covariates. Time in the study (ie, number of days from the first dose of the study drug to the end of the study) was used as an offset variable, and the number of confirmed relapses for each participant was used as the response variable.

The MRI parameters were analyzed in participants who received MRI scans during the study. The numbers of new or newly enlarging T2 and gadolinium-enhancing T1 lesions were analyzed using a negative binomial regression model with a log link. A logistic regression model was used to analyze the proportion of participants who were free from lesions. The total volume and percent change in lesion volumes and brain volume were analyzed using a rank analysis of covariance model. Safety analyses included all participants who had received at least 1 dose of the study drug (safety set) and were summarized descriptively (eMethods in Supplement 2). Data were analyzed between September and November 2018.

## Results

### Study Population

**Figure 1.**
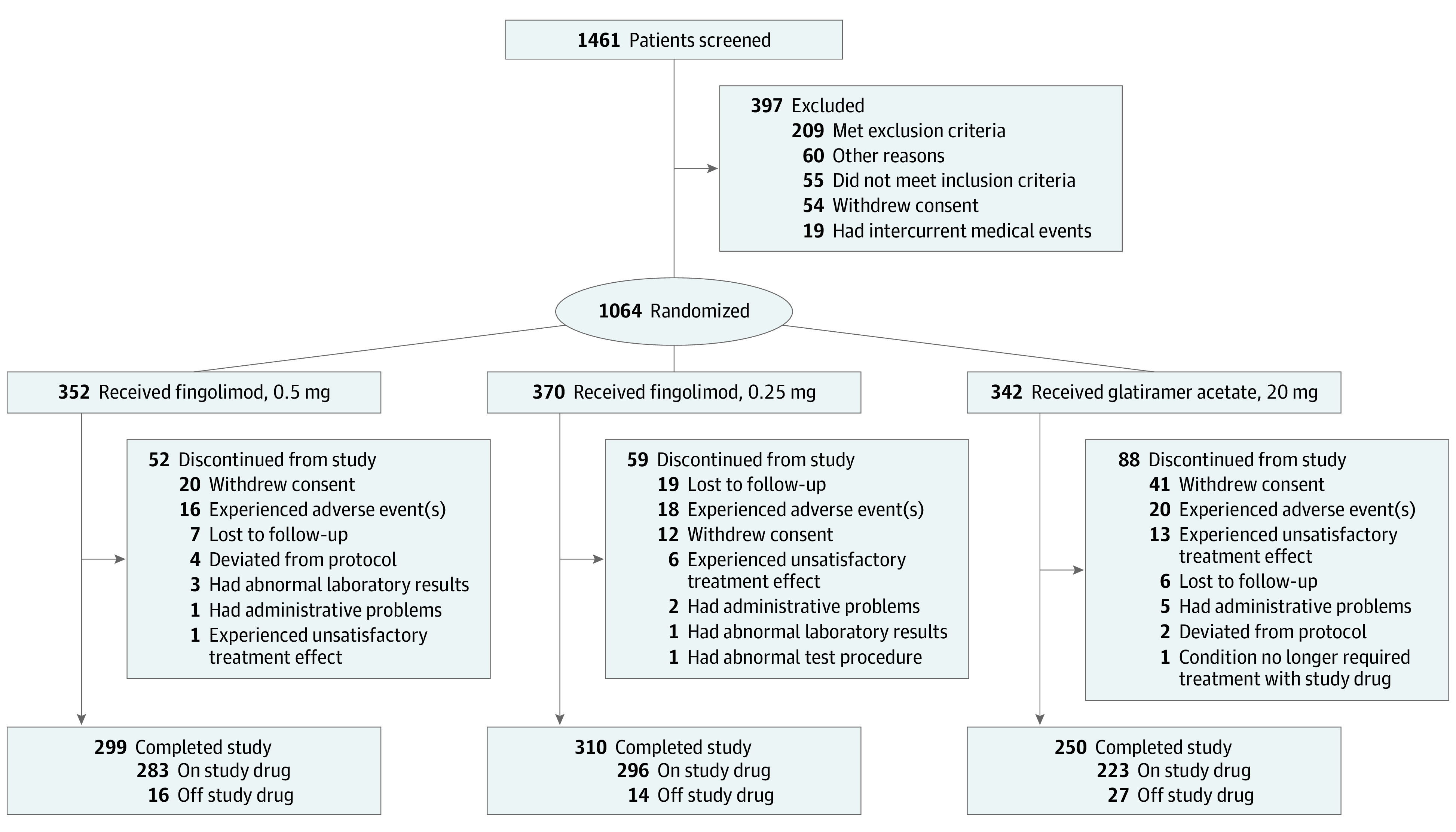
CONSORT Diagram A total of 2 participants from a closed site discontinued the study early, and 4 participants did not enter the treatment phase according to the screening log case report form. These 6 participants were not considered to be discontinued from the study because they were missing study completion case report forms. Off study drug indicates participants who completed the study but discontinued treatment with the study drug prematurely. On study drug indicates participants who received treatment with the study drug until study completion.

A total of 1461 participants were screened; of those, 1064 participants (72.8%) were randomized to 3 treatment groups: fingolimod, 0.5 mg (352 participants), fingolimod, 0.25 mg (370 participants), and glatiramer acetate, 20 mg (342 participants) (Figure 1). Of 1064 participants randomized, the mean (SD) age of participants was 39.6 (11.0) years, 792 participants (74.4%) were women, 790 participants (74.2%) were White, and 499 participants (46.9%) were treatment naive (Table 1). Overall, 859 participants (80.7%) completed the study; a higher proportion of participants in the glatiramer acetate group (88 participants [25.7%]) discontinued the study early compared with the fingolimod groups (52 participants [14.8%] in the fingolimod, 0.5 mg, group and 59 participants [15.9%] in the fingolimod, 0.25 mg, group). The primary reasons for discontinuation in the glatiramer acetate group were withdrawal of consent (41 participants [12.0%]) and adverse events (20 participants [5.8%]) (discontinuation data for all groups are available in Figure 1).

**Table 1.** Demographic and Baseline Disease Characteristics of Randomized Participants

Characteristic	Mean (SD)			
	Fingolimod groups		GA, 20 mg, group	Total
	0.5 mg	0.25 mg		
Participants, No.	352	370	342	1064
Age, y	40.3 (11.1)	38.9 (11.0)	39.6 (10.8)	39.6 (11.0)
Female sex, No. (%)	264 (75.0)	276 (74.6)	252 (73.7)	792 (74.4)
Race, No. (%)				
White	268 (76.1)	279 (75.4)	243 (71.1)	790 (74.2)
Black	34 (9.7)	43 (11.6)	41 (12.0)	118 (11.1)
American Indian	8 (2.3)	7 (1.9)	9 (2.6)	24 (2.3)
Asian	0	1 (0.3)	0	1 (0.1)
Pacific Islander	0	1 (0.3)	0	1 (0.1)
Other	42 (11.9)	39 (10.5)	49 (14.3)	130 (12.2)
Duration of MS since diagnosis, y	4.3 (5.9)	4.6 (6.4)	4.7 (6.2)	4.5 (6.2)
Previous MS therapy, No. (%)	182 (51.7)	195 (52.7)	188 (55.0)	565 (53.1)
Relapses in the last year before screening	1.4 (0.8)	1.3 (0.7)	1.4 (0.8)	1.3 (0.8)
Relapses in the last 2 y before screening	2.2 (1.6)	2.1 (1.3)	2.2 (1.5)	2.1 (1.5)
EDSS score	2.74 (1.46)	2.55 (1.41)	2.73 (1.42)	2.67 (1.43)
MSFC *z* score	–0.02 (0.68)	0.01 (0.70)	0.05 (0.66)	0.01 (0.68)
Participants without gadolinium-enhancing T1 lesions, No. (%)	227 (64.9)	232 (63.0)	219 (64.2)	678 (64.0)
Gadolinium-enhancing T1 lesions				
Count	1.7 (5.5)	2.1 (8.1)	1.6 (5.5)	1.8 (6.5)
Volume, mL	0.30 (1.06)	0.32 (1.41)	0.22 (0.83)	0.28 (1.13)
T1 hypointense lesion volume, mL	3.3 (5.3)	3.2 (5.4)	2.7 (4.1)	3.1 (5.0)
T2 lesion volume, mL	10.3 (12.2)	10.2 (13.0)	8.8 (9.9)	9.8 (11.8)
Normalized brain volume, mL	1502.2 (86.6)	1512.6 (94.2)	1509.6 (90.6)	1508.2 (90.6)

Abbreviations: EDSS, Expanded Disability Status Scale; GA, glatiramer acetate; MS, multiple sclerosis; MSFC, Multiple Sclerosis Functional Composite.

Participant demographic and disease characteristics at baseline were comparable across the 3 treatment groups. Among MRI-measured disease characteristics, mean lesion volumes were numerically higher in both fingolimod groups (mean [SD] volume of gadolinium-enhancing T1 lesions, 0.30 [1.06] mL in fingolimod, 0.5 mg, group and 0.32 [1.41] mL in fingolimod, 0.25 mg, group; mean [SD] volume of T1 hypointense lesions, 3.34 [5.39] mL in fingolimod, 0.5 mg, group and 3.21 [5.39] mL in fingolimod, 0.25 mg, group; mean [SD] volume of T2 lesions, 10.33 [12.23] mL in fingolimod, 0.5 mg, group and 10.15 [13.03] mL in fingolimod, 0.25 mg, group) compared with the glatiramer acetate group (mean [SD] volume of gadolinium-enhancing T1 lesions, 0.22 [0.83] mL; mean [SD] volume of T1 hypointense lesions, 2.69 [4.12] mL; mean [SD] volume of T2 lesions, 8.78 [9.87] mL) (Table 1).

### Efficacy

**Figure 2.**
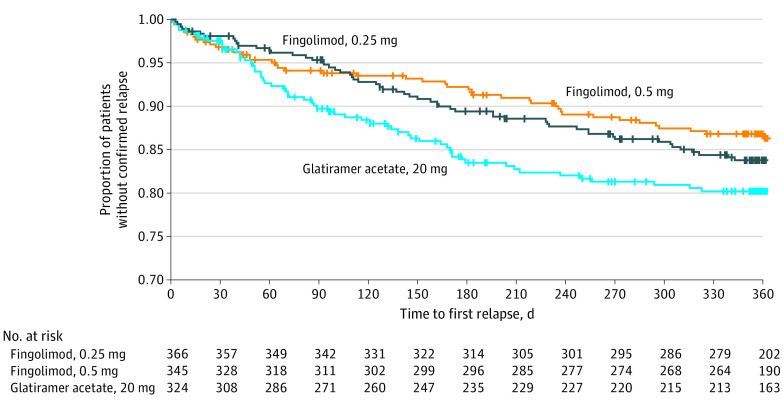
Time to First Confirmed Relapse Through Month 12 for Full Analysis Set

Treatment with fingolimod, 0.5 mg, was superior to treatment with glatiramer acetate for reducing aggregate ARR, with a significant relative reduction of 40.7% (ARR, 0.15; 95% CI, 0.11-0.21 for fingolimod, 0.5 mg, vs ARR, 0.26; 95% CI, 0.20-0.34 for glatiramer acetate; *P* = .01) (Table 2). Fingolimod, 0.25 mg, demonstrated a numerical reduction in aggregate ARR of 14.6% compared with glatiramer acetate; this reduction was not statistically significant (ARR, 0.22; 95% CI, 0.17-0.29 for fingolimod, 0.25 mg, vs ARR, 0.26; 95% CI, 0.20-0.34 for glatiramer acetate; *P* = .42). The time to first relapse was longer, risk reduction for confirmed relapse was reduced, and a higher proportion of participants were free from relapses at month 12 in both fingolimod groups (with a statistically significant higher proportion in the fingolimod, 0.5 mg, group) compared with the glatiramer acetate group (Figure 2; eTable 1 in Supplement 2).

**Table 2.** Primary and Secondary Efficacy End Points for Full Analysis Set

End point	No. (%)		
	Fingolimod groups		GA, 20 mg, group
	0.5 mg	0.25 mg	
Total participants, No.	345	366	324
**Primary**			
Aggregate ARR up to month 12 (confirmed relapses only)			
ARR estimate (95% CI)	0.15 (0.11 to 0.21)	0.22 (0.17 to 0.29)	0.26 (0.20 to 0.34)
ARR ratio vs GA 20 mg	0.59	0.85	NA
Relative reduction vs GA 20 mg, %	40.7	14.6	NA
*P* value	.01^a^	.42	NA
**Secondary**			
New or newly enlarging T2 lesions at month 12 or end of treatment			
Participants evaluated, No.	303	331	272
Mean (SD)	2.6 (5.4)	3.3 (6.9)	5.7 (10.7)
*P* value	<.001^a^	<.001^a^	NA
Participants without new or newly enlarging T2 lesions	156 (51.5)	155 (46.8)	96 (35.3)
*P* value vs GA	<.001^a^	<.001^a^	NA
Gadolinium-enhancing T1 lesions at month 12 or end of treatment			
Participants evaluated, No.	302	325	263
Mean (SD)	0.4 (1.6)	0.4 (1.6)	0.9 (3.7)
*P* value	.02^a^	.001^a^	NA
Participants without gadolinium-enhancing T1 lesions	261 (86.4)	269 (82.8)	202 (76.8)
*P* value vs GA	.004^a^	.06	NA
Change from baseline in T2 lesion volume at month 12 or end of treatment			
Participants evaluated, No.	304	332	272
Mean (SD), mL	–0.14 (1.58)	–0.05 (2.34)	0.42 (2.31)
*P* value vs GA	<.001^a^	.006^a^	NA
Change from baseline in T1 hypointense lesion volume at month 12 or end of treatment			
Participants evaluated, No.	302	331	271
Mean (SD), mL	0.33 (1.08)	0.37 (1.01)	0.25 (1.04)
*P* value vs GA	.25	.03^a^	NA
Gadolinium-enhancing T1 lesion volume at month 12 or end of treatment			
Participants evaluated, No.	302	325	263
Mean (SD), mL	0.06 (0.22)	0.05 (0.18)	0.12 (0.45)
*P* value vs GA	.005^a^	.06	NA
% Change in brain volume from baseline to month 12 or end of treatment			
Participants evaluated, No.	284	305	252
Mean (SD)	–0.65 (0.78)	–0.64 (0.81)	–0.56 (0.78)
*P* value vs GA	.10	.14	NA
Annualized rate of brain atrophy up to month 12 or end of treatment			
Participants evaluated, No.	345	366	324
Mean (95% CI)	–0.68 (–0.80 to –0.56)	–0.69 (–0.81 to –0.58)	–0.68 (–0.81 to –0.56)
*P* value vs GA	.99	.90	NA
Change from baseline in TSQM scores^b^			
Global satisfaction			
Participants evaluated, No.	185	201	180
Mean (SD)	19.2 (31.9)	20.5 (32.2)	9.4 (30.4)
*P* value vs baseline	<.001^a^	<.001^a^	<.001^a^
Effectiveness			
Participants evaluated, No.	187	198	178
Mean (SD)	16.8 (26.9)	17.9 (28.3)	8.0 (27.4)
*P* value vs baseline	<.001^a^	<.001^a^	<.001^a^
Adverse effects			
Participants evaluated, No.	185	194	174
Mean (SD)	16.2 (31.5)	17.2 (32.5)	7.6 (32.8)
*P* value vs baseline	<.001^a^	<.001^a^	.005^a^
Convenience			
Participants evaluated, No.	187	198	178
Mean (SD)	29.5 (24.4)	26.5 (26.4)	0.8 (25.7)
*P* value vs baseline	<.001^a^	<.001^a^	.46
Change from baseline in PASAT-3 score			
Participants evaluated, No.	291	317	276
Mean (SD)	2.4 (9.5)	1.7 (10.0)	1.1 (8.0)
*P* value vs GA 20 mg	.01^a^	.26	NA
Change from baseline in symbol Digit Modalities Test score			
Participants evaluated, No.	301	319	278
Mean (SD)	6.2 (12.6)	6.6 (12.3)	5.1 (13.5)
*P* value vs GA 20 mg	.29	.19	NA
Change from baseline in PRIMUS–Activities score			
Participants evaluated, No.	261	281	230
Mean (SD)	–0.12 (4.21)	0.17 (4.89)	0.55 (4.13)
*P* value vs GA 20 mg	.008^a^	.53	NA
Change from baseline in MSIS-29 score			
Physical impact score			
Participants evaluated, No.	315	336	291
Mean (SD)	–3.5 (16.2)	–1.5 (15.3)	–0.8 (17.1)
*P* value vs GA 20 mg	.007^a^	.15	NA
Psychological impact score			
Participants evaluated, No.	312	336	290
Mean (SD)	–6.6 (19.9)	–3.3 (19.8)	–2.2 (21.4)
*P* value vs GA 20 mg	.001^a^	.12	NA

Abbreviations: ARR, annualized relapse rate; GA, glatiramer acetate; MSIS-29, 29-item Multiple Sclerosis Impact Scale; PASAT-3, Paced Auditory Serial Addition Test 3; PRIMUS–Activities, Patient-Reported Indices in Multiple Sclerosis–Activities scale; TSQM, Treatment Satisfaction Questionnaire for Medication.

^a^ Indicates an unpaired 2-sided statistical significance threshold of *P* = .05.

^b^ Only participants who were receiving disease-modifying therapy for multiple sclerosis at the screening visit completed the TSQM.

Compared with glatiramer acetate, both fingolimod doses significantly reduced the number of new or newly enlarging lesions on T2-weighted MRI scans (54% reduction for fingolimod, 0.5 mg, and 42% reduction for fingolimod, 0.25 mg) and the number of gadolinium-enhancing T1 lesions (56% reduction for both doses) at month 12 (Table 2). A significantly higher proportion of participants were free from the development of new or newly enlarging T2 lesions in both fingolimod groups compared with participants in the glatiramer acetate group. A greater proportion of participants were also free from the development of gadolinium-enhancing T1 lesions in both fingolimod groups compared with participants in the glatiramer acetate group, but statistical significance was reached in the fingolimod, 0.5 mg, group only (Table 2).

Differences in the change from baseline in T2 lesion volume at month 12 were significantly in favor of treatment with both of the fingolimod doses compared with treatment with glatiramer acetate (Table 2). The mean reduction in gadolinium-enhancing T1 lesion volume was statistically significant for fingolimod, 0.5 mg, and numerically in favor of fingolimod, 0.25 mg. The T1 hypointense lesion volume increased in all treatment groups by month 12; treatment with fingolimod, 0.25 mg, showed a significantly greater volume increase than glatiramer acetate, while no significant volume difference was observed between treatment with fingolimod, 0.5 mg, and treatment with glatiramer acetate (Table 2). The mean percent change from baseline in brain volume was similar with the 2 doses of fingolimod vs glatiramer acetate (Table 2).

Treatment satisfaction improved in all 3 treatment groups across the different domains, and the improvement was 2-fold higher in the fingolimod, 0.5 mg, group and fingolimod, 0.25 mg, group compared with the glatiramer acetate group (Table 2). Patients who received treatment with fingolimod, 0.5 mg, showed greater improvement in functional disability, cognition (significant improvement on the Paced Auditory Serial Addition Test 3 but not in the Symbol Digit Modalities Test) (Table 2), and health-related quality of life outcomes (significant improvement on both the Patient-Reported Indices in Multiple Sclerosis–Activities scale and the Multiple Sclerosis Impact Scale) compared with those who received treatment with glatiramer acetate (details are provided in the eMethods and eTable 2 in Supplement 2).

### Safety and Tolerability

The mean (SD) duration of exposure to both fingolimod doses was greater than that of glatiramer acetate (for fingolimod, 0.5 mg, 301 [94] participant-years; for fingolimod, 0.25 mg, 325 [87] participant-years; for glatiramer acetate, 252 [121] participant-years). Adverse events were reported in similar proportions across the 3 treatment groups (312 participants [90.4%] in the fingolimod, 0.5 mg, group, 323 participants [88.3%] in the fingolimod, 0.25 mg, group, and 283 participants [87.3%] in the glatiramer acetate group) (Table 3). The most common adverse events reported in the fingolimod groups were headache (52 participants [15.1%] in the fingolimod, 0.5 mg, group and 52 participants [14.2%] in the fingolimod, 0.25 mg, group), fatigue (46 participants [13.3%] in the fingolimod, 0.5 mg, group and 46 participants [12.6%] in the fingolimod, 0.25 mg, group), and urinary tract infection (44 participants [12.8%] in the fingolimod, 0.5 mg, group and 43 participants [11.7%] in the fingolimod, 0.25 mg, group). The most common adverse events reported in the glatiramer acetate group were urinary tract infection (37 participants [11.4%]), injection site pain (36 participants [11.1%]), and injection site erythema (34 participants [10.5%]).

**Table 3.** Safety Profile

Adverse event	No. (%)		
	Fingolimod groups		GA, 20 mg, group
	0.5 mg	0.25 mg	
Total participants, No.	345	366	324
Events ≥3% in any group			
Any event	312 (90.4)	323 (88.3)	283 (87.3)
Headache	52 (15.1)	52 (14.2)	27 (8.3)
Fatigue	46 (13.3)	46 (12.6)	22 (6.8)
Urinary tract infection	44 (12.8)	43 (11.7)	37 (11.4)
Upper respiratory tract infection	30 (8.7)	35 (9.6)	20 (6.2)
Hypertension	26 (7.5)	34 (9.3)	12 (3.7)
Nausea	24 (7.0)	30 (8.2)	15 (4.6)
Pain in extremity	24 (7.0)	24 (6.6)	17 (5.2)
Depression	23 (6.7)	19 (5.2)	18 (5.6)
Lymphopenia	23 (6.7)	18 (4.9)	1 (0.3)
Nasopharyngitis	23 (6.7)	23 (6.3)	14 (4.3)
Diarrhea	21 (6.1)	23 (6.3)	10 (3.1)
Increase in alanine aminotransferase level	20 (5.8)	15 (4.1)	4 (1.2)
Back pain	17 (4.9)	19 (5.2)	18 (5.6)
Dyspnea	17 (4.9)	10 (2.7)	6 (1.9)
Decrease in lymphocyte count	16 (4.6)	10 (2.7)	1 (0.3)
Sinusitis	16 (4.6)	16 (4.4)	15 (4.6)
Arthralgia	15 (4.3)	20 (5.5)	13 (4.0)
Cough	15 (4.3)	13 (3.6)	11 (3.4)
Increase in γ glutamyltransferase level	15 (4.3)	12 (3.3)	2 (0.6)
Muscle spasms	14 (4.1)	15 (4.1)	11 (3.4)
Paresthesia	14 (4.1)	13 (3.6)	11 (3.4)
Vision blurred	14 (4.1)	13 (3.6)	6 (1.9)
Anxiety	13 (3.8)	21 (5.7)	9 (2.8)
Increase in hepatic enzymes	13 (3.8)	2 (0.5)	2 (0.6)
Influenza	13 (3.8)	16 (4.4)	9 (2.8)
Alopecia	12 (3.5)	17 (4.6)	3 (0.9)
Bronchitis	12 (3.5)	10 (2.7)	6 (1.9)
Dizziness	12 (3.5)	27 (7.4)	14 (4.3)
Hypoesthesia	12 (3.5)	17 (4.6)	10 (3.1)
Insomnia	12 (3.5)	27 (7.4)	9 (2.8)
Oropharyngeal pain	12 (3.5)	8 (2.2)	4 (1.2)
Migraine	11 (3.2)	17 (4.6)	8 (2.5)
Vomiting	11 (3.2)	9 (2.5)	6 (1.9)
Fall	9 (2.6)	10 (2.7)	10 (3.1)
Muscular weakness	9 (2.6)	13 (3.6)	5 (1.5)
Vertigo	9 (2.6)	12 (3.3)	6 (1.9)
Constipation	7 (2.0)	17 (4.6)	5 (1.5)
Dry eye	5 (1.4)	5 (1.4)	10 (3.1)
Eye pain	4 (1.2)	14 (3.8)	3 (0.9)
Injection site			
Erythema	0	0	34 (10.5)
Mass	0	0	12 (3.7)
Pain	0	0	36 (11.1)
Pruritus	0	0	26 (8.0)
Reaction	0	0	21 (6.5)
Serious events >0.5% in any group			
Any event	25 (7.2)	32 (8.7)	20 (6.2)
Multiple sclerosis relapse	4 (1.2)	7 (1.9)	7 (2.2)
Basal cell carcinoma	3 (0.9)	4 (1.1)	0
Headache	1 (0.3)	3 (0.8)	0
Urinary tract infection	2 (0.6)	1 (0.3)	2 (0.6)
Seizure	1 (0.3)	2 (0.5)	0
Syncope	0	0	2 (0.6)
Dyspnea	2 (0.6)	0	0
Gait disturbance	0	2 (0.5)	0
Leukocytosis	0	2 (0.5)	0
Events leading to study drug discontinuation			
Any event	32 (9.3)	27 (7.4)	45 (13.9)
Increase in hepatic enzymes	4 (1.2)	2 (0.5)	0
Drug hypersensitivity	0	0	6 (1.9)
Injection site			
Pain	0	0	6 (1.9)
Reaction	0	0	7 (2.2)

Abbreviation: GA, glatiramer acetate.

Serious adverse events were reported with a slightly higher frequency in the fingolimod, 0.25 mg, group (32 participants [8.7%]) than in the fingolimod, 0.5 mg, group (25 participants [7.2%]) or the glatiramer acetate group (20 of 324 participants [6.2%]). Serious adverse events that were reported by at least 1% of participants in any group included MS relapse, a higher incidence of MS relapse in the glatiramer acetate group, and basal cell carcinoma (which occurred in the fingolimod groups only). No deaths were reported during the study. Adverse events leading to discontinuation of the study drug were reported more frequently in the glatiramer acetate group (45 of 324 participants [13.9%]) than in the fingolimod, 0.5 mg, group (32 of 345 participants [9.3%]) and the fingolimod, 0.25 mg, group (27 of 366 participants [7.4%]).

The adverse events of increased hepatic enzymes, lymphopenia, urinary tract infection, increased alanine aminotransferase level, increased γ-glutamyltransferase level, and decreased lymphocyte count were reported with higher frequency in the fingolimod groups. Dizziness, insomnia, constipation, and eye pain also occurred with higher frequency (>2% difference) in the fingolimod, 0.25 mg, group than in the fingolimod, 0.5 mg, group. Macular edema was reported in 5 participants (3 participants [0.9%] in the fingolimod, 0.5 mg, group and 2 participants [0.5%] in the fingolimod, 0.25 mg, group) approximately 3 months after initiation of treatment with the study drug. Fingolimod was discontinued in all 5 participants with macular edema; the edema had resolved in 3 participants and was still ongoing in 2 participants at the final study visit. The most common type of malignancy observed was basal cell carcinoma, which was reported in 4 participants (1.2%) in the fingolimod, 0.5 mg, group, 4 participants (1.1%) in the fingolimod, 0.25 mg, group, and 0 participants in the glatiramer acetate group. Other malignancies, reported in 1 participant each, included acral lentiginous melanoma (glatiramer acetate group), metastatic biliary cancer (fingolimod, 0.25 mg, group), invasive ductal breast carcinoma (glatiramer acetate group), malignant melanoma (glatiramer acetate group), and squamous cell carcinoma of skin (fingolimod, 0.25 mg, group).

Nine pregnancies were reported. Six of the 9 participants had positive pregnancy test results during treatment or within 45 days of discontinuation of the study drug. These participants included 1 patient each from the fingolimod, 0.25 mg, group and glatiramer acetate groups, and 4 patients from the fingolimod, 0.5 mg, group. Of those, 1 patient exposed to fingolimod, 0.25 mg, during pregnancy experienced a spontaneous abortion, and all other patients had normal pregnancy outcomes.

#### Treatment Initiation Experience

Most participants were discharged within 6 hours of treatment initiation in both fingolimod groups (302 participants [88.0%] in the fingolimod, 0.5 mg, group and 324 participants [88.8%] in the fingolimod, 0.25 mg, group). Five of 343 participants (1.5%) in the fingolimod, 0.5 mg, group (3 participants with first-degree atrioventricular block; 1 participant at the investigator’s discretion; and 1 participant with headache, hypoesthesia of the lips or tongue, or dyspnea) and 1 participant (0.3%) in the fingolimod, 0.25 mg, group (with concomitant medication) required overnight hospitalization. As expected, a dose-dependent decrease in heart rate was observed after initiation of fingolimod treatment. The maximal decrease in mean (SD) supine pulse rate was 8.6 (9.5) beats per minute in the fingolimod, 0.5 mg, group (5 hours after treatment) and 5.3 (8.2) beats per minute in the fingolimod, 0.25 mg, group (4 hours after treatment). A pulse rate of less than 50 beats per minute was observed in 19 of 212 participants (9.0%) in the fingolimod, 0.5 mg, group and 9 of 228 participants (3.9%) in the fingolimod, 0.25 mg, group; a pulse rate of less than 45 beats per minute was observed in 6 of 212 participants (2.8%) in the fingolimod, 0.5 mg, group and 1 of 228 participants (0.4%) in the fingolimod, 0.25 mg, group. Bradycardia was reported in 6 participants (4 participants [1.2%] in the fingolimod, 0.5 mg, group and 2 participants [0.5%] in the fingolimod, 0.25 mg, group). All of these events were considered asymptomatic by the treating physician.

Electrocardiogram evaluations showed that at 6 hours after treatment, first-degree atrioventricular block was the most common abnormal result reported in the fingolimod groups (21 participants [6.2%] in the fingolimod, 0.5 mg, group and 17 participants [4.7%] in the fingolimod, 0.25 mg, group). Second-degree atrioventricular Mobitz type 1 block was observed in 2 participants (0.6%) in the fingolimod, 0.5 mg, group at 6 hours after treatment.

#### Lymphocyte Count

The mean absolute lymphocyte counts at baseline were comparable across groups (mean [SD] for the fingolimod, 0.5 mg, group, 1.99 [0.66] × 10^3^/μL; for the fingolimod, 0.25 mg, group, 2.01 [0.64] × 10^3^/μL; for the glatiramer acetate group, 2.04 [0.61] × 10^3^/μL; 10^3^**/**μL is equivalent to 10^9^/L). At month 1, lymphocyte counts were reduced by more than 60% of the baseline value in the fingolimod groups and remained stable thereafter.

Over the study period, 230 participants (67.1%) in the fingolimod, 0.5 mg, group had lymphocyte counts of less than 0.4 × 10^3^/μL compared with 140 participants (38.4%) in the fingolimod, 0.25 mg, group. In addition, 49 participants (14.3%) in the fingolimod, 0.5 mg, group, 16 participants (4.4%) in the fingolimod, 0.25 mg, group, and 1 participant (0.3%) in the glatiramer acetate group had a lymphocyte count of less than 0.2 × 10^3^/μL. An association between lymphopenia or lymphocytes counts and an increased risk of infection was not observed.

#### Recruitment Challenges and Selection Bias

Because of recruitment challenges, many sites recruited smaller than expected numbers of participants, with approximately 30% of sites enrolling 3 or fewer patients. To address potential selection bias that might have been introduced because of the large number of sites with few participants, a post hoc analysis of ARR was explored.

Sites were grouped into 6 predefined geographic regions (Latin American, northeastern, western, southwestern, southeastern, and midwestern), and region was included as a random effect in a negative binomial model. The model converged with results similar to the fixed effects observed in the primary analysis (eResults and eTable 3 in Supplement 2). Further subgroup analysis by region as well as age, sex, and the presence of gadolinium-enhanced lesions at baseline showed ARR and ARR ratios consistent with the results for the overall population.

## Discussion

Fingolimod, 0.5 mg, therapy met the primary objective of significantly reducing the rate of relapse over 12 months compared with glatiramer acetate therapy. The superiority of fingolimod, 0.5 mg, to glatiramer acetate was accompanied by improvements in other relapse-related outcomes, including a delay in the time to first relapse and an increase in the proportion of participants who were relapse-free at 12 months. Treatment with fingolimod, 0.5 mg, also showed superiority vs treatment with glatiramer acetate in various MRI measures evaluated. The efficacy of low-dose fingolimod (0.25 mg) was evaluated for the first time. For the primary end point, fingolimod, 0.25 mg, demonstrated a numerical reduction in aggregate ARR of 14.6% compared with glatiramer acetate, although this difference was not statistically significant.

Participants in both fingolimod groups had significantly lower numbers of new or newly enlarging T2 and gadolinium-enhancing T1 lesions compared with participants in the glatiramer acetate group. Treatment with fingolimod, 0.5 mg, significantly reduced T2 lesion and gadolinium-enhancing T1 lesion volumes from baseline vs treatment with glatiramer acetate; for treatment with fingolimod, 0.25 mg, the reduction was significant only in T2 lesion volume at month 12. No effect on brain volume was observed with either fingolimod dose compared with glatiramer acetate. These findings are not unexpected because glatiramer acetate has been shown to reduce the rate of brain volume loss.^19^ However, in several important clinical trials, fingolimod reduced the rate of brain volume loss compared with placebo and interferon.^7,13^ Overall, findings in the ASSESS clinical trial support the previous findings of a study of fingolimod compared with intramuscular interferon beta-1a, which indicated that fingolimod, 0.5 mg, is superior to frequently used injectable disease-modifying therapies.^7^

The parallel-group study design allowed for direct comparison of efficacy, tolerability, and safety among the 3 treatment groups. A double-blind design would have been ideal; however, this design was deemed unsuitable because administering daily placebo injections for participants in the 2 fingolimod groups was not considered ethical practice for a postmarketing study of an already approved product. A blinded rater confirmed the occurrence of relapses, and the double-blinding of the fingolimod dose provided protection against bias.

Both fingolimod doses were well tolerated, with approximately 80% of participants completing the study, and the safety profile observed with both doses was consistent with the profiles observed in other clinical trials of fingolimod, 0.5 mg,(cumulative exposure to fingolimod, 0.5 mg, in clinical trials alone is >28 000 participant-years).^7,12,13^ Safety findings were generally comparable between the 2 fingolimod dose groups. A dose-dependent increase in the incidence of fingolimod-specific pharmacodynamic effects was observed. Bradycardia, cardiac conduction defects, and decreases in lymphocyte count are known pharmacodynamic effects of sphingosine 1-phosphate receptor modulation and were reported at a lower incidence in the fingolimod, 0.25 mg, group compared with the fingolimod, 0.5 mg, group. However, the incidence of other adverse events of special interest, such as macular edema, hypertension, infections, and malignancies (including skin cancer), were comparable between the fingolimod dose groups.

### Limitations

 This study has several limitations. Participant enrollment was slower than expected, presenting a substantial challenge that eventually led to the premature termination of recruitment. The commercial availability of thrice-weekly glatiramer acetate 40 mg along with 2 other oral therapies, teriflunomide and dimethyl fumarate, might have contributed, at least in part, to slow recruitment of individuals for the glatiramer acetate group, in which participants received once-daily 20-mg subcutaneous injections. A consequence of recruitment challenges was that many sites recruited small numbers of participants, introducing a potential source of selection bias; however, post hoc analyses suggested that the results were not caused by any particular subgroup.

The recruitment of a smaller sample than initially planned reduced the statistical power to test the study hypotheses to 71% for fingolimod, 0.5 mg, vs glatiramer acetate and to 44% for fingolimod, 0.25 mg, vs glatiramer acetate. Given both of the overlapping 95% CIs of ARR point estimates between fingolimod, 0.5 mg, and fingolimod, 0.25 mg, and the lower actual effect size assumed for ARR reduction for fingolimod, 0.25 mg, during protocol development (15% reported effect size vs 25% estimated effect size), even with full recruitment of 1996 participants, the likelihood that the study would have shown that fingolimod, 0.25 mg, was superior to glatiramer acetate was small. In a retrospective observational study that investigated the efficacy and safety of fingolimod, 0.5 mg, administered every other day (which would presumably decrease the dose to 0.25 mg per day), disease reactivation was observed in a significant proportion of patients despite a reversed association with abnormal laboratory test results, including lymphopenia and elevated liver function tests, that was observed with the dose reduction.^20,21^

## Conclusions

The ASSESS study demonstrates that fingolimod, 0.5 mg, is superior to glatiramer acetate, 20 mg, in reducing the rates of relapse and activity on MRI scans. Treatment with fingolimod, 0.5 mg, also showed greater improvements across functional disability, cognition, and quality of life outcomes. Fingolimod, 0.5 mg, has a superior benefit-risk profile compared with low-dose fingolimod, 0.25 mg. These results support the use of fingolimod, 0.5 mg, as the optimal efficacious dose of fingolimod for the treatment of adult patients with relapsing-remitting MS.

## Notes

Supplement 1. Trial Protocol and Statistical Analysis Plan

Supplement 2. **eMethods.** ASSESS Steering Committee, Exclusion Criteria, Definition of Confirmed Relapse, and Statistical Analyses for Secondary and Exploratory End Points **eResults.** MSFC *z* Score and Subscales, SDMT Scores, HR-QoL Outcomes, and Post Hoc Analysis of Selection Bias **eTable 1.** Exploratory End Points **eTable 2.** Relapse Outcomes **eTable 3.** Negative Binomial Regression of Confirmed Relapses Up to 12 Months **eFigure.** Study Design eReferences

Supplement 3. Data Sharing Statement
