# Supplementary material for: Efficacy and Safety of 2 Fingolimod Doses vs Glatiramer Acetate for the Treatment of Patients With Relapsing-Remitting Multiple Sclerosis: A Randomized Clinical Trial
Source: JAMA Neurol. 2020 Aug 24;78(1):1–13. doi: 10.1001/jamaneurol.2020.2950 (PMC7445630; doi:10.1001/jamaneurol.2020.2950)
Supplement: Supplement 1. — Trial Protocol and Statistical Analysis Plan [file jamaneurol-e202950-s001.pdf]

NOVARTIS

Clinical Development

FTY720/Fingolimod

Clinical Trial Protocol CFTY720D2312

A 12-month, randomized, rater- and dose-blinded study to compare the efficacy and safety of fingolimod 0.25 mg and 0.5 mg administered orally once daily with glatiramer acetate 20 mg administered subcutaneously once daily in patients with relapsing-remitting multiple sclerosis

Authors:	[redacted]
Document type:	Amended Protocol Version
Version number:	v02 Clean
EUDRACT number:	Not applicable
Development phase:	IIIb
Release date:	20-Jul-2015

Property of Novartis
Confidential
May not be used, divulged, published, or otherwise disclosed
without the consent of Novartis
Template version 24 May 2011

Table of contents

Table of contents 2
List of tables 5
List of figures 5
List of abbreviations 6
Glossary of terms 9
Protocol synopsis 23
1 Introduction 28
1.1 Background 28
1.2 Purpose 31
2 Study objectives 32
2.1 Primary objective 32
2.2 Secondary objectives 32
2.3 Exploratory objectives 32
2.4 Objectives of the immune-cell substudy 33
3 Investigational plan 33
3.1 Study design 33
3.2 Rationale of study design 35
3.3 Rationale of dose/regimen, duration of treatment 36
3.4 Rationale for choice of comparator 36
3.5 Purpose and timing of interim analyses/design adaptations 36
4 Population 36
4.1 Inclusion criteria 37
4.2 Exclusion criteria 37
5 Treatment 41
5.1 Investigational and control treatment 41
5.2 Treatment arms 41
5.3 Treatment assignment 41
5.4 Treatment blinding 42
5.5 Treating the patient 42
5.5.1 Patient numbering 42
5.5.2 Dispensing the study treatment 43
5.5.3 Supply, storage, and tracking of study treatment 43
5.5.4 Instructions for prescribing and taking study treatment 43
5.5.5 Guidelines for monitoring patients taking their first dose of fingolimod 44

5.5.6 Permitted dose adjustments and interruptions of study treatment 46
5.5.7 Recommendations on treatment of multiple sclerosis relapse 47
5.5.8 Concomitant treatment 48
5.5.9 Prohibited treatment 48
5.5.10 Discontinuation of study treatment and premature patient withdrawal 49
5.5.11 Emergency unblinding of treatment assignment for patients on fingolimod 49
5.5.12 Study completion and post-study treatment 50
5.5.13 Early study termination 50
6 Visit schedule and assessments 50
6.1 Information to be collected on screening failures 53
6.2 Patient demographics/other baseline characteristics 53
6.3 Treatment exposure and compliance 54
6.4 Efficacy 54
6.4.1 Multiple sclerosis relapse 54
6.4.2 Expanded Disability Status Scale 55
6.4.3 Magnetic Resonance Imaging (MRI) 55
6.4.4 Multiple Sclerosis Functional Composite (MSFC) 56
6.4.5 Symbol Digit Modalities Test (SDMT) 57
6.4.6 Appropriateness of efficacy assessments 57
6.5 Safety Assessments 57
6.5.1 Physical examination 57
6.5.2 Vital signs 58
6.5.3 Laboratory evaluations 58
6.5.4 Electrocardiogram 60
6.5.5 Ophthalmologic examinations and optical coherence tomography 61
6.5.6 Pregnancy and assessments of fertility 61
6.5.7 Pharmacodynamic assessments (substudy only) 61
6.5.8 Chest x-ray 63
6.5.9 Pulmonary function tests 63
6.5.10 Dermatological examination 64
6.5.11 Columbia-Suicide Severity Rating Scale 64
6.5.12 Appropriateness of safety measurements 65
6.6 Other assessments 65
6.6.1 Health-related quality of life 65
6.6.2 Resource utilization 66

6.6.3 Pharmacogenetics/pharmacogenomics 66
6.6.4 Pharmacokinetics 66
7 Safety monitoring 67
7.1 Adverse events 67
7.2 Serious adverse event reporting 68
7.2.1 Serious adverse event notification: multiple sclerosis relapse 69
7.3 Pregnancies 69
7.4 Data safety monitoring board 69
8 Data review and database management 70
8.1 Site monitoring 70
8.2 Data collection 70
8.3 Database management and quality control 70
9 Data analysis 71
9.1 Analysis sets 71
9.2 Patient demographics and other baseline characteristics 72
9.3 Treatments (study drug, rescue medication, other concomitant therapies, compliance) 72
9.4 Analysis of the primary variable 72
9.4.1 Variable 72
9.4.2 Statistical model, hypothesis, and method of analysis 72
9.4.3 Handling of missing values, censoring, and discontinuations 74
9.5 Analysis of secondary variables 75
9.5.1 Efficacy variables 75
9.5.2 Safety variables 77
9.5.3 Resource utilization 78
9.5.4 Health related quality of life 78
9.5.5 Pharmacokinetics 79
9.5.6 Pharmacogenetics/pharmacogenomics 79
9.5.7 Biomarkers 79
9.5.8 Pharmacokinetic/pharmacodynamic 79
9.6 Sample size calculation 79
9.7 Power for the key secondary hypotheses 80
9.8 Immune-cell substudy analyses 80
9.9 Interim analysis 80
10 Ethical considerations 81
10.1 Regulatory and ethical compliance 81
10.2 Informed consent procedures 81

10.3 Responsibilities of the investigator and IRB/IEC 81
10.4 Publication of study protocol and results 81
11 Protocol adherence 82
11.1 Protocol amendments 82
12 References (Available Upon Request) 82
13 Appendices 85
Appendix 1: Clinically notable laboratory values and vital signs 85
Appendix 2: 2010 Revisions to the McDonald diagnosis criteria for MS 87
Appendix 3: Guidance on safety monitoring 89
Appendix 4: Guidance for ophthalmic monitoring 94
Appendix 5: Multiple sclerosis functional composite measure (MSFC) 96

List of tables

Table 6-1 Assessment schedule 51
Table 6-2 Abbreviated schedule of assessments for patients who discontinue study drug 52
Table 6-3 Severity of multiple sclerosis relapse 55

List of figures

Figure 3-1 Study design 34

List of abbreviations

AE	adverse event
ALT	alanine aminotransferase
ANCOVA	analysis of covariance
AP	alkaline phosphatase
ARR	annualized relapse rate
AST	aspartate aminotransferase
AV	atrioventricular
CFR	United States Code of Federal Regulations
CNS	central nervous system
CO	carbon monoxide
CHMP	Committee for Medicinal Products for Human Use
CPO	Country Pharma Organization
CRF	case report forms
CRO	contract research organization
C-SSRS	Columbia-Suicide Severity Rating Scale
D_LCO	diffusion capacity of carbon monoxide
DMD	disease modifying drug
DS&E	Drug Safety and Epidemiology
DSMB	data safety monitoring board
DTPA	diethylenetriamine penta-acetic acid
eCRF	electronic case report/record form
ECG	electrocardiogram
EDSS	Expanded Disability Status Scale
EOT	end of treatment
FA	fluorescein angiography
FACS	fluorescein activated cell sorter
FAS	full analysis set
FEV_1	forced expiratory volume in 1 second
FS	functional system
FVC	forced vital capacity
Gd	gadolinium

GGT	gamma glutamyl-transferase
HbA1c	glycosylated hemoglobin/hemoglobin A1c
HRCT	high-resolution computed tomography
HSV	herpes simplex virus
ICH	International Conference on Harmonization of Technical Requirements for Registration of Pharmaceuticals for Human Use
IEC	independent ethics committee
IFN	interferon
Ig	immunoglobulin
IgG	immunoglobulin G
IM	intramuscular
IRB	institutional review board
IV	intravenous
IVRS	interactive voice response system
MedDRA	Medical Dictionary for Regulatory Activities
MS	multiple sclerosis
MSFC	Multiple Sclerosis Functional Composite
MSIS-29	Multiple Sclerosis Impact Scale
MRI	magnetic resonance imaging
OCT	optical coherence tomography
QoL	quality of life
PASAT	paced auditory serial addition test
PBMC	peripheral blood mononuclear cell
PCR	polymerase chain reaction
PD	pharmacodynamic
PFT	pulmonary function test
PK	pharmacokinetic(s)
PPD	Pharmaceutical Product Development (designated CRO)
PRIMUS	Patient Reported Indices in Multiple Sclerosis
PRO	patient reported outcome
RRMS	relapsing-remitting multiple sclerosis

SD	standard deviation
S1P	sphingosine 1-phosphate
SAE	serious adverse event
SC	subcutaneous
SDMT	Symbol Digit Modalities Test
Treg	regulatory T cell
TSQM	Treatment Satisfaction Questionnaire for Medication
ULN	upper limit of normal
VZV	varicella zoster virus
WBC	white blood cells

Glossary of terms

Assessment	A procedure used to generate data required by the study
Baseline	The collection of pre-randomization information on subsequently randomized patients for future description and analysis. For any data analysis, the baseline for any data point will be clearly identified in the Statistical Analysis Plan.
Control drug	A study drug used as a comparator to reduce assessment bias, assess internal study validity, and/or evaluate comparative effects of the investigational drug
Enrollment	Point/time of patient entry into the study; the point at which informed consent must be obtained (i.e., before starting any of the procedures described in the protocol)
Investigational drug	The study drug whose properties are being tested in the study; this definition is consistent with US CFR 21 Section 312.3 and is synonymous with “investigational new drug.”
Medication number	A unique identifier on the label of each study drug package in studies that dispense study drug using an IVR system
Patient number	A number assigned to each patient who enrolls in the study. When combined with the center number, a unique identifier is created for each patient in the study.
Period	A minor subdivision of the study timeline; divides phases into smaller functional segments such as screening, baseline, titration, washout, etc.
Premature patient withdrawal	Point/time when the patient exits from the study before the planned completion of all study drug administration and assessments; at this time all study drug administration is discontinued and no further assessments are planned
Randomization number	A unique identifier assigned to each randomly assigned patient, corresponding to a specific treatment arm assignment
Stop study participation	Point/time at which the patient came in for a final evaluation visit or when study drug was discontinued whichever is later
Study drug	Any drug administered to the patient as part of the required study procedures; includes investigational drug and any control drugs
Study drug discontinuation	Point/time when patient permanently stops taking study drug for any reason; may or may not also be the point/time of premature patient withdrawal
Variable	Information used in the data analysis; derived directly or indirectly from data collected using specified assessments at specified timepoints

Amendment 02

Amendment rationale

This amendment implements changes to the CFTY720D2312 protocol amendment 1 (dated 20-Nov-2012) to address recommendations and requests received from the United States (US) Food and Drug Administration (FDA).

The FDA has approved the Sponsor's proposal to reduce the sample size for this study. The FDA recommended to keep the number of patients in the fingolimod (FTY720) 0.25-mg treatment arm the same and agreed to reduce the other two treatment arms by up to 50% of the original sample size. This has led to a change in the randomization ratio between the three treatment groups and as a consequence an alteration of the calculated sample size. This sample size provides approximately 90% power for the primary objective. After the introduction of protocol amendment 2, the anticipated total number of patients to be screened is reduced to approximately 2610 from original 3190 patients. A screening failure rate of 25% is anticipated (supported by the current trend in this D2312 study [status Feb 2015]) and a total of 1960 randomized patients are targeted: fingolimod 0.25 mg (total 847 patients), fingolimod 0.5 mg (total 615 patients), and glatiramer acetate 20 mg (total 498 patients), respectively.

In addition, this amendment implements modifications to the inclusion/exclusion criteria. These modifications are prompted by:

- Feedback from study investigators and consultation with the study advisory group to help widening the pool of eligible subjects
- Alignment of the protocol with fingolimod's current US prescribing information and the prescribing information in other countries which are within the scope of this study as this is a Phase IIIb study with a marketed drug

The exclusion criterion 1 related to history of malignancy was rationalized to allow inclusion of patients who are now free of the past malignancy and have no trace of malignancy for the last 5 years.

The exclusion criterion 3 related to use of previous multiple sclerosis (MS) treatments was modified bearing in mind the likely duration of pharmacodynamic (PD) action of previous therapies and the assessments inherent in the screening assessments (e.g., complete blood counts) to rule out the persistence of such PD actions. Also, the criterion was modified to introduce guidance on the washout for newly approved disease-modifying therapies namely teriflunomide and dimethyl fumarate.

The requirement for serology testing of hepatitis A, B, C, and E, at Screening was omitted. This criterion (exclusion criterion 8 in protocol amendment version 1) has been modified to be in alignment with the current US prescribing information and the prescribing information in other countries which are within the scope of this study which does not require such screening before initiating treatment with fingolimod in clinical practice. The liver functions are being tested very closely at Screening, Baseline, and periodically during the study to capture any

instances of liver infections/impairment (if any) early and are considered as adequate safeguards.

The current exclusion criterion 8 regarding mandatory serologic screening for antibodies against varicella zoster virus (VZV) is a modification which also allows for alternative acceptable evidence of immunity (as per Center for Disease Control guidance). This brings the protocol in alignment with the guidance in the current US prescribing information in this context.

The current exclusion criteria 12, 13, and 14 have modified text to align with the US prescribing information and the prescribing information in other countries which are within the scope of this study. The exclusion clauses in protocol amendment version 1 were more restrictive than that recommended in the US prescribing information resulting in exclusion of those patients with certain pre-existing cardiac conditions or on concomitant drugs prolonging heart rate or atrioventricular (AV) conduction, who would have been otherwise eligible to initiate fingolimod with extended monitoring.

Certain clarifications and corrections were also done in the protocol as follows:

The exclusion criterion 16 provides clarification that patients with controlled asthma are allowed to participate in the study.

The prohibition period for the administration of a live or live-attenuated vaccine after fingolimod discontinuation was corrected from 12 to 8 weeks (as per US prescribing information).

Clarification was provided that the patients known to be allergic to gadolinium (Gd) may participate in the trial with magnetic resonance imaging (MRI) performance modified only to forgo contrasted MRI images.

The following erroneous paragraph was deleted: Patients experiencing bradycardia that has not resolved by the end of the 6-hour monitoring period must be hospitalized overnight. For these patients, the Day 2 dose of fingolimod should be given under the supervision of the treating physician, and the 6-hour monitoring period should be repeated per the procedures outlined for the first dose.

Throughout the protocol, clarifications of wording and correction of minor inconsistencies were provided for improved study conduct. This included allowance of use of topical and inhaled steroids for indications other than MS in Section 5.5.8, relaxation on the dosing adjustments of symptomatic therapies for optimal patient care in Section 5.5.8, details of the assessments to be performed for ophthalmologic examination in Table 6-1 and Table 6-2, the guidance that only two paced auditory serial addition test (PASAT) test runs (neither 9-hole peg test nor 25-foot walk) are to be performed at Screening for the Multiple Sclerosis Functional Composite (MSFC) score in Section 6.4.4.

In Appendix 3, investigators were guided to consider appropriate antihypertensive medication/dosage adjustment for newly diagnosed hypertension as well as an aggravation of a pre-existing condition. The earlier guidance to consider the discontinuation of study drug was deleted as it was an erroneous statement and not in line with the US prescribing information of fingolimod. Similarly, clarity was provided on the repeat testing in cases of

alanine aminotransferase/aspartate aminotransferase rise above 5 times the upper limit of normal (ULN).

Also, further clarity is being provided to investigators for interruption of glatiramer acetate when the lymphocyte count decreases below 50% of the baseline levels.

The protocol is also being amended to address safety updates in the investigator brochure (Edition 18) – in particular to provide additional guidance for safety monitoring for opportunistic infections and for basal cell carcinoma.

- Notably, there have been reports of isolated cases of cryptococcal meningitis in MS patients with relapsing MS receiving fingolimod. As a result, the fingolimod local product labeling will be updated to guide prescribers for vigilance, early detection, and diagnosis of such cases, should they occur. Similarly, the infection safety monitoring guidance is being updated in this protocol (Appendix 3).
- Basal cell carcinoma has been reported in patients receiving fingolimod. The physical examination section has been amended to ask patients about any new or worsening skin lesions and to instruct investigators to refer patients to the dermatologist in cases where suspected precancerous or cancerous skin lesions are identified.

The requirement for chest x-ray at Screening was removed as it was considered unnecessary by investigators, in line with the current safety profile of fingolimod. The pulmonary function testing and clinical examination of the respiratory system are considered to be adequate screening measures to evaluate pulmonary status.

Furthermore, clarity was provided for immune-cell PD substudy sections pertaining to exploratory objectives and methodology. In addition, the number of sites initiating this substudy was increased, as was the number of patients to be screened.

Changes to the protocol

- The planned number of enrolled patients was reduced from 2250 to 1959 to provide a power of approximately 90% for the comparison of the annualized relapse rate (ARR) between fingolimod 0.5 mg to glatiramer acetate at a 2-sided significance level of 0.05. The power of the statistical test for the comparison of fingolimod 0.25 mg with glatiramer acetate will depend on whether the efficacy seen with fingolimod 0.5 mg is maintained in the lower dose (the 0.25-mg dose of fingolimod that has never been studied before). (Section 3.1, Section 4, and Section 9.6)
- The randomization ratio to the three treatment arms (fingolimod 0.25 mg, fingolimod 0.5 mg, and glatiramer acetate 20 mg) was changed from 1:1:1 to 5:3:2 to comply with the FDA recommendations on revision of the sample size in the three arms. The revised sample size calculation assumes that this switch will occur when a total of approximately 700 patients have been randomized to treatment under the original randomization ratio. (Section 3.1 and Section 4)
- The alpha-propagation procedure was replaced in protocol amendment 2 by a step-down hierarchical testing procedure in which the approved dose of fingolimod is initially tested against glatiramer acetate (at a 2-sided significance level of 0.05), and only if this initial test is significant will the low dose of fingolimod be tested against glatiramer acetate (also

at 2-sided significance level of 0.05). This change was introduced because of the alteration in the planned number of patients. (Section 9.4.2 and Section 9.6)

- Consequently, the original primary objective of the study "The primary objective is to demonstrate that at least 1 dose (0.5 mg or 0.25 mg) of fingolimod is superior to glatiramer acetate 20 mg subcutaneous (SC) in reducing the annualized relapse rate (ARR) up to 12 months in patients with relapsing-remitting MS" was reworded to: "The primary objective is to demonstrate that at least 1 dose (tested hierarchically 0.5 mg followed by 0.25 mg) of fingolimod is superior to glatiramer acetate 20 mg SC in reducing the ARR up to 12 months in patients with relapsing-remitting MS." (Section 2.1)
- The exploratory objectives of the immune-cell substudy were revised. (Section 2.4) As a consequence, the PD assessments performed as part of this substudy were also amended. (Section 6.5.7)
- The number of sites performing the immune-cell substudy was increased to approximately 10-15 sites and the number participants to be enrolled in the immune substudy was increased to approximately 48 at Screening. (Section 3.1)
- Inclusion criterion 7 was removed and appropriate text was added to exclusion criterion 3 (see the following bullet point) with addition of instruction for dimethyl fumarate. (Section 4.1 and Section 5.5.8)
- Exclusion criterion 1 was changed as follows: Patients with a history of malignancy of any organ system (other than cutaneous basal cell carcinoma) in the last 5 years that do not have confirmation of absence of a malignancy prior to randomization. (Section 4.2)
- Exclusion criterion 3 was amended. The modified exclusion criterion is as follows: (Section 4.2)
  - Intravenous (IV) immunoglobulin (Ig) within 4 weeks before randomization
  - Immunosuppressive/chemotherapeutic medications (e.g., azathioprine, cyclophosphamide, methotrexate) within 6 months before randomization
  - Natalizumab within 2 months before randomization
  - Previous treatment with lymphocyte-depleting therapies (e.g., rituximab, alemtuzumab, ofatumumab, ocrelizumab, or cladribine) within 1 year before randomization
  - Previous treatment with mitoxantrone 6 months before randomization
  - Use of teriflunomide within 3.5 months prior to randomization, except if active washout (with either cholestyramine or activated charcoal) was done. In that case, plasma levels are required to be measured and to be below 0.02 mg/L before randomization.

No washout period is necessary for patients treated with dimethyl fumarate, interferon (IFN) beta, or glatiramer acetate. Patients being treated with dimethyl fumarate, glatiramer acetate, or IFN beta at the screening visit can continue drug intake up to the day before Day 1 of this study (i.e., there is no need for a washout period).

- The earlier (in protocol amendment version 1) exclusion criteria 8 (screening for hepatitis A, B, C, and E) was removed. (Section 6.5.3.2 and Table 6-1)
- Current exclusion criterion 8 has the following modified text regarding VZV: Patients without acceptable evidence of immunity to VZV at randomization (See Appendix 3 for guidance on acceptable evidence of immunity and requirement for serologic testing). (Section 4.2, Section 6.5.3.2, and Table 6-1)
- Exclusion criterion 13 in protocol amendment version 1 has been modified and divided in exclusion criteria 12, 13, 14, and 15 (Section 4.2) as listed in the following 4 bullet points:
  - Exclusion criterion 12: Patients who in the last 6 months experienced any of the following cardiovascular conditions or findings in the screening electrocardiogram (ECG):
    - myocardial infarction
    - unstable angina
    - stroke
    - transient ischemic attack
    - decompensated heart failure requiring hospitalization or Class III/IV heart failure
  - Exclusion criterion 13: Patients with history or presence of a Mobitz Type II AV block, or a third-degree AV block or sick sinus syndrome, unless patient has a functioning pacemaker
  - Exclusion criterion 14: Patients with baseline QTc interval >500 msec
  - Exclusion criterion 15: Patients receiving Class Ia (e.g., ajmaline, disopyramide, procainamide, quinidine) or Class III antiarrhythmic drugs (e.g., amiodarone, bretylium, sotalol, ibutilide, azimilide, dofetilide)
- Exclusion criterion 15 in protocol amendment version 1 has become exclusion criterion 16 and is modified as follows: Patients with severe respiratory disease, pulmonary fibrosis, or Class III or IV chronic obstructive pulmonary disease, or with clinically significant lung pathology on chest x-ray. Patients with controlled asthma are allowed to enter the study. (Section 4.2)
- Exclusion criterion 14 in protocol amendment version 1 was deleted: Patients receiving current treatment with (at treatment initiation) beta blockers, heart-rate slowing calcium-channel blockers (e.g., ivadrabine, verapamil, or diltiazem), or other substances which may decrease heart rate such as digoxin, anticholinesterase agents, or pilocarpine. Advice from a cardiologist should be sought regarding the switch to nonheart rate lowering medicinal products. (Section 4.2)

  Similarly, text was deleted from Section 5.5.4 (Instructions for prescribing and taking study treatment): Due to a possible additive effect on heart rate reduction it is

recommended not to initiate treatment with beta blockers, calcium-channel blockers, or digoxin within 1 week before randomization. In patients randomized to treatment with fingolimod, treatment with beta blockers, calcium-channel blockers, or digoxin should also not be initiated within one week after the start of study drug administration.

- Exclusion criterion 25 in protocol amendment version 1 has become exclusion criterion 26 and revised as follows: Patients with a score of 4 or 5 on the Suicidal Ideation item of the Columbia-Suicide Severity Rating Scale (C-SSRS) within 2 years of Screening, or any "yes" on the Suicidal Behavior item of the C-SSRS at Screening. (Section 4.2)
- Guidance was provided for re-screening of patients. (Section 4)
- Additional monitoring guidelines were added for patients with any pre-existing cardiovascular conditions and those receiving concurrent therapy with drugs that slow heart rate or AV conduction (e.g., beta blockers, heart rate lowering calcium-channel blockers such as diltiazem, verapamil, or digoxin). (Section 5.5.5)
- The following paragraph was deleted: "Patients experiencing bradycardia that has not resolved by the end of the 6-hour monitoring period must be hospitalized overnight." For these patients, the Day 2 dose of fingolimod should be given under the supervision of the treating physician, and the 6-hour monitoring period should be repeated per the procedures outlined for the first dose. (Section 5.5.5)
- Topical and inhaled steroids were allowed at the discretion of the treating neurologist for indications other than MS. (Section 5.5.8)
- There was modification to the statement regarding permitted medications for symptomatic treatment of MS. The current text reads as follows: Medications for the symptomatic treatment of MS such as baclofen, fampridine, methylphenidate, or modafinil are acceptable. As far as possible, every effort should be made to keep the dosages stable from Screening through end of treatment (EOT). (Section 5.5.8)
- The prohibition period for the administration of a live or live-attenuated vaccine after fingolimod discontinuation was corrected from 12 to 8 weeks. The statement that the vaccines may be administered once the lymphocyte counts are in laboratory normal range was deleted. (Section 5.5.9 and Appendix 3)
- Clarification is provided on what assessments are to be performed for ophthalmologic examination. The current text reads: The ophthalmology examination will include eye history, visual acuity, dilated ophthalmoscopy, and any procedures necessary to assess macular edema. Optical coherence tomography and fluorescein angiography will be done only if needed to confirm macular edema. (Table 6-1 and Table 6-2)
- Guidance was provided about the patients known to be allergic to Gd: The patients known to be allergic to Gd may participate in the trial with MRI assessment modified to forgo the contrasted MRI images. (Section 6.4.3)
- Clarification is provided that only two PASAT test runs (neither 9-hole peg test nor 25-foot walk) are to be performed at Screening for the MSFC score. (Section 6.4.4)

- The additional blood sample (for possible future exploratory assessments) collection has been restricted to only the screening visit (from earlier 3 occasions: Screening, Month 6, and at EOT visit). Also, an example was provided for possible use of such a sample (e.g., relevant safety analysis like retrospective viral serology). (Section 6.5.3.2 and Table 6-1)
- Guidance was provided when an MS relapse should be reported as a serious adverse event (SAE). The text reads: Any MS relapse, as a general rule, should be reported on the relapse CRF instead of the adverse event (AE)/SAE forms. However, if, in the judgment of the investigator, an MS relapse is unusually severe or unexpected and warrants specific notification, then an SAE form must be completed and submitted according to SAE reporting procedures outlined above. (Section 7.2.1)
- Guidance on safety monitoring was revised with respect to the actions to be taken with newly diagnosed hypertension. The current text is: Newly diagnosed hypertension as well as an aggravation of a pre-existing condition must be reported as an AE and appropriate antihypertensive medication/dosage adjustment must be considered by the investigator. (Appendix 3)
- Clarification was provided under guidance on monitoring of patients with elevated liver function tests (Appendix 3). The current text is: If alanine aminotransferase/aspartate aminotransferase values reach 5 times the ULN, confirmed upon repeat testing within 2 weeks of the initial result or sooner at the discretion of the investigator, the study drug must be permanently discontinued. Patients who develop symptoms suggestive of hepatic dysfunction such as unexplained vomiting or jaundice, should have liver enzymes checked and fingolimod should be discontinued if significant liver injury is confirmed.
- Investigators were advised to be vigilant for early diagnosis and treatment of cryptococcal meningitis and basal cell carcinoma (Appendix 3: Guidance for monitoring of infections; Section 6.5.1)
- The guidance to investigators for interruption of glatiramer acetate when the lymphocyte values decrease below 50% of baseline values was revised. (Appendix 3)
- Clarity was provided on the blinded and unblinded reporting of results for fingolimod. (Appendix 3)

Changes to specific sections of the protocol are shown in the track changes version of the protocol using ~~strike through red font for deletions~~ and <u>red underlined for insertions</u>.

A copy of this amended protocol will be sent to the Institutional Review Boards (IRBs)/Independent Ethics Committees (IECs), and health authorities.

The changes described in this amended protocol require IRB/IEC approval prior to implementation. In addition, if the changes herein affect the informed consent, sites are required to update and submit for approval a revised informed consent that takes into account the changes described in this amended protocol.

Amendment 01

Amendment rationale

This amendment implements changes to the CFTY720D2312 protocol (dated 23-Sep-2011) to address recommendations and requests received from the United States Food and Drug Administration (FDA) following their review of the protocol and to align aspects of the protocol with the current revised fingolimod (Gilenya™) label. It also addresses recommendations received from the European regulator agency on the immune-cell investigation planned as a substudy within this protocol.

The main changes involve inclusion of additional safety assessments, to allow comprehensive risk/benefit assessment of the 0.5 mg per day fingolimod dose [currently in the market], the lower 0.25 mg per day dose [under investigation in this study] and glatiramer acetate [control]. The safety assessments that are now being included for all participating patients are regular pulmonary function tests, dermatological examinations, ophthalmic examinations and assessment of suicidality. Specific guidance is provided for full workup in patients developing cardiac symptoms; pulmonary hypertension; ischemic or thrombotic events proteinuria. Suicidality assessment is also included in compliance with FDA guidance applicable to all drugs that have an effect on the central nervous system.

Safety sections of this protocol are revised with regard to cardiac safety. In December 2011, the company reported a sudden, unexplained death of a patient within 24 hours after the administration of the first dose of commercial fingolimod 0.5 mg. While the cause of death is unknown, the role of fingolimod cannot be confirmed or excluded. The reporting of this event prompted a thorough review the cardiac safety data from clinical trials as well as spontaneous reports in the post-marketing setting.

In order to align the protocol with the current revised fingolimod label, this amendment additionally updates and modifies (1) specific exclusion criteria, (2) selected safety monitoring guidance and (3) revision of the requirements for first dose monitoring following fingolimod interruption.

In addition, the amendment includes changes to facilitate the conduct of the study and includes clarifications of wording and other minor corrections.

The study is currently enrolling patients, and approximately 22 patients have been enrolled at the time of release of this amendment.

In summary the changes in the amendment refer to the following:

- pulmonary function testing
- dermatological examinations
- additional ophthalmological examination at 6 months
- body weight monitoring
- addition of certain laboratory analyses
- collection of laboratory analysis data that are not associated with a regular study visit
- provision for referral of patient to a specialist in the event of new significant cardiac or pulmonary hypertension symptoms

- inclusion of patients with higher glycosylated hemoglobin (HbA1c) levels but without diabetic neuropathy
- collection of additional baseline characteristics for diabetic patients
- provision for full diagnostic workup in patients with an ischemic or thrombotic adverse event
- a suicide severity rating assessment

Other changes to the protocol either for clarification, to improve conduct of the study, or to increase patient safety include:

- deletion of exclusion criterion regarding patients with history of vitamin B12 deficiency
- clarification of effective contraception methods
- deletion of redundant exclusion criterion pertaining to treatment with high dose steroids
- additional instructions to minimize risk of unblinding of blinded site personnel
- change of day of screening to be as early as 45 days prior to randomization
- further specification of study drug storage conditions
- clarification of use of the Patient Reported Indices in Multiple Sclerosis (PRIMUS) Activities instrument to be used only in selected countries
- specification of assay for detecting hepatitis C virus
- change of pharmacokinetic (PK) sample storage and shipment specifications
- direction for central laboratory reporting of hematology results to maintain site blinding
- expansion of immune-cell substudy size to include screening samples on approximately 36 participants in substudy and to increase in blood sample volume of subsequent samples in fingolimod patients
- minor wording corrections and administrative changes

The protocol has been amended to expand the population to be evaluated to include diabetic patients whose glycosylated hemoglobin levels are moderately elevated. The changes included in this amendment will provide additional data for the safety profiles of the study treatments.

Changes to the protocol

- The authors of the protocol were changed. (Title cover page)
- Background (Section 1.1) was revised to include updated patient exposure, updated summary of adverse events, addition of atrioventricular (AV) conduction safety information, and statement regarding patients with cardiovascular or cerebrovascular disease for which fingolimod is not recommended.
- Additional exploratory objectives were added to the immune-cell substudy. (Section 2.4)
- The number of sites performing the immune-cell substudy was increased to approximately 5-10 sites and a provision to re-evaluate the sample size of the substudy after analysis of the data from 24 patients was added. The number of participants to be enrolled in the substudy was increased to approximately 36 at Screening to obtain approximately 24 participants receiving fingolimod. A 20 mL blood sample was added at the screening visit to reduce variability of baseline values (Screening and Day 1). A blood sample was

corrected to be performed at Month 3 rather than at Month 6 and the volume to be collected was increased to ensure adequate numbers of cells are obtained for the assessment. (Section 3.1, Section 3.2, Section 6.5.7, Table 6-1, Table 6-2)

- The screening visit has been changed to occur up to 45 days prior to randomization (Section 3.1, Figure 3-1, Table 6-1)
- Inclusion criterion #4 was changed to remove reference to systemic steroid use because this was covered within exclusion criterion #4. (Section 4.1)
- The exclusion criterion regarding the use of intravenous immunoglobulin was changed for clarity to exclude any patients receiving intravenous immunoglobulin within 2 months prior to randomization. (Section 4.2)
- The exclusion criterion regarding uncontrolled diabetes was changed to allow participation by diabetics if their HbA1c level is 9% or less and they are without diabetic neuropathy. (Section 4.2)
- The exclusion criterion regarding history of vitamin B12 deficiency was removed. (Section 4.2)
- The exclusion criterion regarding serological markers to detect hepatitis C was changed to remove anti-hepatitis C virus IgM and to add hepatitis C virus RNA polymerase chain reaction (PCR) as an assay. (Section 4.2, Section 6.5.3.2, Table 6-1)
- Exclusion criteria were expanded to exclude patients with additional cardiovascular conditions and patients receiving treatments which may decrease heart rate. (Section 4.2)
- Exclusion criterion #15 was expanded to exclude patients who have clinically significant lung pathology by chest x-ray at Screening. A chest x-ray was added to assessments at the screening visit if an x-ray within the previous 3 months is not available. (Section 4.2, Table 6-1, Section 6.5.8)
- Exclusion criterion #22 pertaining to effective contraception was expanded to define highly effective methods of contraception. (Section 4.2)
- An exclusion criterion was added to exclude patients with a score of “yes” on item 4 or item 5 of the Suicidal Ideation section of the Columbia-Suicide Severity Rating Scale (C-SSRS), if this ideation occurred in the past 6 months prior to Screening, or “yes” on any item of the Suicidal Behavior section, except for the “Non-Suicidal Self-Injurious Behavior” in the past 2 years. (Section 4.2)
- Redundant exclusion criterion for high dose steroid use in patients participating in the immune-cell substudy was deleted. (Section 4.2)
- Because different sourcing strategies may be applied for glatiramer acetate depending on the country and because this control treatment is unblinded to the dispensing pharmacist and treating physician, medication numbering was removed as a requirement for the label for glatiramer acetate. (Section 5.1)
- The description of study drug storage specifies that all study drug is to be stored refrigerated. (Section 5.5.3)
- Management of patients with bradycardia following the first dose of fingolimod was changed to require overnight monitoring and second dose monitoring only if the patient was treated for the bradycardia. Patients experiencing prolonged QTc at 6 hours after first

dose of fingolimod are required to be monitored overnight and second dose monitoring is required. (Section 5.5.5)

- A statement was added in Section 5.5.9 to clarify that fingolimod may be restarted after a patient receives a live or live attenuated vaccine.
- Additional diabetes-specific baseline characteristics will be collected in patients with diabetes. (Section 6.2)
- The qualifications of the rater of the Expanded Disability Status Scale were clarified. (Section 6.4.2)
- A dermatological examination will be performed at Screening, 12 months, end of treatment, and end of study to identify new skin lesions. (Table 6-1, Table 6-2, Section 6.5, Section 6.5.10, and Section 9.5.2)
- Systolic and diastolic blood pressure will be measured in the supine position followed by measurements with the patient standing, with documentation of orthostatic hypotension if it occurs. (Section 5.5.5 and Section 6.5.2)
- Discharge criteria following first dose have been expanded to include review of ECG for AV block, prolonged QTc, and decreasing heart rate. They also include requirement for overnight hospitalization in the event that pharmacologic intervention is required during first dose observation period or for prolonged QTc. (Section 5.5.5)
- The requirement for restarting study drug and on-site monitoring after fingolimod interruption and restarting was changed. (Section 5.5.6)
- Additional instructions are provided for administering the Symbol Digit Modalities Test (SDMT). (Section 6.4.5)
- Direction was added that the central laboratory will only report hematology absolute counts of eosinophils, basophils, and monocytes to maintain site blinding. The central laboratory will only report absolute total white blood cell, neutrophil, and lymphocyte counts to the site in case of a clinically notable abnormality. (Section 6.5.3.1)
- Body weight will be assessed at each visit to identify weight gain that may be indicative of edema. (Table 6-1, Section 6.5.2, Appendix 1)
- A footnote was added to Table 6-1 and Table 6-2 and text was added in Section 6.5.2 clarifying at which visits body temperature is to be measured.
- An additional ophthalmological examination will be performed at 6 months of treatment. (Table 6-1)
- Wording was added to clarify that blood samples are to be in the fasted state at the screening visit and to clarify that blood samples at subsequent visits are recommended to be in the fasted state. (Section 6.5.3)
- HbA1c will be included in all chemistry assessments. (Section 6.5.3.2)
- Uric acid and bicarbonate will be included in the serum chemistry assessments. (Section 6.5.3.2)
- Urinalysis will be performed at all visits. Procedures have been added to follow-up edema, weight gain, or proteinuria with additional urinalysis and follow-up by nephrologist if protein/creatinine ratio is abnormal. (Table 6-1, Table 6-2, and Section 6.5.3.3)

- Patients who experience new significant cardiac symptoms or symptoms of pulmonary hypertension are to be referred to a specialist for further diagnostic workup. (Section 6.5.1)
- The method for analyzing Tregs and TH17 cells in the pharmacodynamic substudy is further clarified, and methods for analysis of other immune-cell subsets are described. (Section 6.5.7)
- Pulmonary function tests (PFTs), including forced expiratory volume in 1 second (FEV_1), forced vital capacity (FVC), and diffusion capacity of carbon monoxide (D_LCO), will be performed at Screening, Month 6, Month 12, end of study, and 3 months after study drug discontinuation. (Table 6-1, Table 6-2, Section 6.5.9, Section 9.5.2, and References) Guidance for monitoring pulmonary function is provided in Appendix 3.
- Patients who are withdrawn from the study because of a respiratory adverse event(s) should be evaluated by a pulmonary specialist and further investigations (PFTs, chest x-ray or high-resolution computed tomography [HRCT], biopsy) as needed. (Section 6.5.9 and Section 7.1)
- The C-SSRS will be administered using an interactive voice response system (IVRS) at Screening and at every study visit after randomization. (Table 6-1, Table 6-2, Section 6.5.11, and Section 9.5.2)
- Instructions for storage and shipment of PK samples were changed to specify that specimens can be stored at –18°C and should be shipped monthly to the central laboratory. (Section 6.6.4)
- Patients experiencing an ischemic or thrombotic adverse event are to be referred to a specialist for a diagnostic workup and management. (Section 7.1)
- Laboratory specimens that are not in association with a regular study visit are to be sent to the central laboratory for analysis and inclusion of the data in the study data sets. (Section 7.1)
- The assessment of the performance of daily activities as measured by the PRIMUS-Activities instrument will be performed only in a subset of selected countries. (Section 2.3, Table 6-1, Table 6-2, and Section 6.6.1.2)
- Notable values for additional laboratory assessments have been added to Appendix 1 (Clinically notable laboratory values and vital signs), and some notable values were changed.
- Previous Appendix 3 was deleted because exclusion criterion referring to this appendix was deleted.
- Appendix 3 was revised to require discontinuation of study drug if clinical symptoms of infection accompany lymphopenia. Discontinuation of study drug for lymphopenia in the absence of clinical signs of infection is at the discretion of the investigator.
- Appendix 3 was revised to require discontinuation of study drug in patients receiving glatiramer acetate who develop significant lymphopenia.
- Appendix 4 was revised to include guidance on ophthalmic monitoring of patients with any visual disturbances while on therapy and in patients with diabetes mellitus or uveitis.

A copy of this amended protocol will be sent to the Institutional Review Boards (IRBs)/Independent Ethics Committees (IECs) and Health Authorities.

The changes described in this amended protocol require IRB/IEC approval prior to implementation. In addition, if the changes herein affect the Informed Consent, sites are required to update and submit for approval a revised Informed Consent that takes into account the changes described in this amended protocol.

Protocol synopsis

Title of study: A 12-month, randomized, rater- and dose-blinded study to compare the efficacy and safety of fingolimod 0.25 mg and 0.5 mg administered orally once daily with glatiramer acetate 20 mg administered subcutaneously once daily in patients with relapsing-remitting multiple sclerosis

Purpose and rationale: The purpose of this study is to compare the efficacy and safety of fingolimod 0.50 mg and fingolimod 0.25 mg to glatiramer acetate (20 mg) for the treatment of patients with relapsing-remitting multiple sclerosis (RRMS) as part of a postapproval commitment for the FDA.

A substudy will be conducted in a few select sites in approximately 24 patients to assess the impact of chronic fingolimod treatment on the proportions of Th17 and regulatory T (Treg) cells in the blood of multiple sclerosis (MS) patients chronically treated with fingolimod as part of a postapproval commitment for the CHMP of the European Medicines Agency.

Objectives:

Primary Objective:

The primary objective is to demonstrate that at least 1 dose (tested hierarchically 0.5 mg followed by 0.25 mg) of fingolimod is superior to glatiramer acetate 20 mg subcutaneous (SC) in reducing the annualized relapse rate (ARR) up to 12 months in patients with relapsing-remitting MS.

Secondary Objectives:

To evaluate in fingolimod 0.5 mg, fingolimod 0.25 mg, and glatiramer acetate 20 mg groups:

- the change from Baseline in brain volume at Month 12 or end of study
- the number of active T2 lesions (new or newly enlarging lesions compared with Baseline) at Month 12 or end of study.
- the proportion of patients free of new or newly enlarging T2 lesions compared to Baseline at Month 12 or end of study
- the change from Baseline in T2 lesion volume at Month 12 or end of study
- the change from Baseline in the number and volume of T1 hypointense lesions at Month 12 or end of study
- the number and volume of gadolinium (Gd)-enhancing T1 lesions at Month 12 or end of study
- the safety and tolerability up to Month 12
- the change from baseline in treatment satisfaction as measured by the Treatment Satisfaction Questionnaire for Medication (TSQM) at Month 12 or end of study

Exploratory Objectives:

- To explore the pharmacokinetics (PK) of fingolimod and fingolimod-phosphate
- To explore the effect of fingolimod 0.5 mg, fingolimod 0.25 mg, and glatiramer acetate on:
  - Multiple Sclerosis Functional Composite (MSFC) and Symbol Digit Modalities Test (SDMT) scores up to Month 12
  - The proportion of relapse-free patients at Month 12
  - The time to first relapse
  - The severity of relapses as assessed by the proportion of patients with mild, moderate or severe relapse, with steroid use, with hospitalization, or with incomplete recovery up to 12 months
  - The performance of daily activities as measured by the Patient Reported Indices in Multiple Sclerosis (PRIMUS-Activities) instrument at Month 12 or end of study in selected countries
  - Quality of life (QoL) as assessed by the change from Baseline in the Multiple Sclerosis Impact Scale (MSIS-29) at Month 12 or end of study

Objectives for Immune-Cell Substudy:

Primary objective:

- To measure the effect of fingolimod treatment on the profile of Th17 and Treg cells to total lymphocyte number upon reaching steady state and under chronic exposure

Secondary objectives:

To evaluate the following in MS patients chronically treated with fingolimod:

- The time course of change in Th17 and Treg proportion over 1 year of fingolimod 0.5 mg and fingolimod 0.25 mg treatment
- The effect of chronic fingolimod 0.5 mg and fingolimod 0.25 mg treatment on the ratio of CD4+ and CD8+ T-cells

Population:

The study population will consist of adult male and female patients with RRMS. Treatment-naïve patients and patients previously treated with disease-modifying therapies for MS, with the exception of S1P modulator therapy, are eligible to participate in the study. The anticipated total number of patients to be screened is approximately 2610, with approximately 1960 patients randomly assigned to study treatment.

Key Inclusion/Exclusion criteria:

Inclusion criteria:

1. Written informed consent must be obtained before any assessment is performed
2. Male and female patients 18 to 65 years of age, inclusive.
3. Patients with RRMS, as defined by 2010 revised McDonald criteria.
4. Patients must be neurologically stable with no onset of relapse within 30 days of randomization.
5. Patients with at least 1 documented relapse during the previous year or 2 documented relapses during the previous 2 years before randomization.
6. Patients with an Expanded Disability Status Scale (EDSS) score of 0 to 6.0, inclusive, at Screening. A score of 6.0 indicates unilateral assistance (cane or crutch) required to walk at least 100 meters with or without resting.

Exclusion criteria:

1. Patients with a history of malignancy of any organ system (other than cutaneous basal cell carcinoma) in the last 5 years that do not have confirmation of absence of a malignancy prior to randomization.
2. Patients with an active chronic disease (or stable but treated with immune therapy) of the immune system other than MS (e.g., rheumatoid arthritis, scleroderma, Sjogren's syndrome, Crohn's disease, ulcerative colitis) or with a known immunodeficiency syndrome (HIV-antibody positive, AIDS, hereditary immune deficiency, drug-induced immune deficiency).
3. Patients who have been treated with:
   - Intravenous (IV) immunoglobulin (Ig) within 4 weeks before randomization
   - Immunosuppressive/chemotherapeutic medications (e.g., azathioprine, cyclophosphamide, methotrexate) within 6 months before randomization
   - Natalizumab within 2 months before randomization
   - Previous treatment with lymphocyte-depleting therapies (e.g., rituximab, alemtuzumab, ofatumumab, ocrelizumab, or cladribine) within 1 year before randomization
   - Previous treatment with mitoxantrone within 6 months before randomization
   - Use of teriflunomide within 3.5 months prior to randomization, except if active washout (with either cholestyramine or activated charcoal) was done. In that case, plasma levels are required to be measured and be below 0.02 mg/L before randomization

No washout period is necessary for patients treated with dimethyl fumarate, interferon (IFN) beta, or glatiramer acetate.

Patients being treated with dimethyl fumarate, glatiramer acetate, or IFN beta at the Screening visit can continue drug intake up to the day before Day 1 of this study (i.e., there is no need for a washout period).

4. Patients who have been treated with systemic corticosteroids or adrenocorticotropic hormones in the past 30 days prior to the screening magnetic resonance imaging (MRI) procedure.
5. Patients with uncontrolled diabetes mellitus (glycosylated hemoglobin >9%) or with diabetic neuropathy.
6. Patients with a diagnosis of macular edema during Screening (patients with a history of macular edema will be allowed to enter the study provided that they do not have macular edema at Screening).
7. Patients with severe active bacterial, viral, or fungal infections.
8. Patients without acceptable evidence of immunity to varicella zoster virus (VZV) at randomization (See Appendix 3 for guidance on acceptable evidence of immunity and requirement for serologic testing).
9. Patients who have received any live or live-attenuated vaccines (including VZV, herpes simplex, or measles) within 1 month before randomization.
10. Patients who have received total lymphoid irradiation or bone marrow transplantation.
11. Patients with any unstable medical/psychiatric condition, as assessed by the primary treating physician at each site.
12. Patients who in the last 6 months experienced any of the following cardiovascular conditions or findings in the screening electrocardiogram (ECG): myocardial infarction, unstable angina, stroke, transient ischemic attack or decompensated heart failure requiring hospitalization or Class III/IV heart failure.

Investigational drug:

- Fingolimod (FTY720) 0.5-mg capsules for oral administration once daily
- Fingolimod (FTY720) 0.25-mg capsules for oral administration once daily

Both strengths of fingolimod capsule will be identical in appearance and will be packaged in identical bottles.

Control drug:

Glatiramer acetate 20-mg SC injection once daily.

Study design:

This is a multicenter, randomized, rater- and dose-blinded study to compare the efficacy and safety of 0.25 mg and 0.5 mg of fingolimod with glatiramer acetate 20 mg SC in patients with RRMS.

This study will consist of 3 periods:

- Screening Period: up to 45 days for all patients
- Treatment Period: 12 months of glatiramer acetate 20 mg, fingolimod 0.25 mg, or fingolimod 0.5 mg
- Follow-up Period will occur 3 months (12 weeks) after the last dose of study drug for all patients

After signing the informed consent, patients will enter the Screening Period to determine eligibility for the study. After inclusion/exclusion criteria are reviewed again and after safety assessments are conducted, patients will enter the Treatment Period and will be randomly assigned into one of three groups (for details about the randomization see Section 9.6 - Sample size calculation):

- Group 1 will receive fingolimod 0.5 mg orally once a day for up to 12 months
- Group 2 will receive fingolimod 0.25 mg orally once a day for up to 12 months

- Group 3 will receive glatiramer acetate 20 mg subcutaneously once a day for up to 12 months

Patients will take their first dose of study drug at Visit 2. Thereafter, patients in each fingolimod treatment arm will take 1 capsule orally once a day for 12 months. Patients in the glatiramer acetate treatment arm will self-administer an SC injection of 20 mg of glatiramer acetate once a day for 12 months. In order to ensure patient safety 6-hour first dose monitoring procedures are to be followed for patients receiving fingolimod. First-dose monitoring is to be conducted by the primary treating physician.

Starting at Visit 3/Month 1, all visits in the Treatment Period have a visit window of ±5 days. At Visit 7/end of treatment (EOT)/Month 12, the study staff should complete the study completion electronic case report form (eCRF).

The end-of-study follow-up visit will occur 3 months (12 weeks) after the last dose of study drug administration (for patients who complete the study and for patients who prematurely discontinue the study).

Efficacy assessments:

- MS ARR
- Magnetic resonance imaging (MRI)
- Multiple sclerosis functional composite (MSFC)
- Symbol digits modalities test (SDMT)

Safety assessments:

- Physical and neurological examination
- Vital sign measurements
- Laboratory evaluations
- ECG results
- Ophthalmologic examinations
- Pulmonary function tests
- Dermatological examination
- Columbia-Suicide Severity Rating Scale

Pharmacokinetics/pharmacodynamics assessments:

- Pharmacokinetic assessments will only be performed on patients treated with fingolimod.
- Pharmacodynamic (PD) assessments will only be performed on patients enrolled in the regulatory immune-cell substudy.

Quality of life assessments:

- Patient-reported outcomes for multiple sclerosis (TSQM, PRIMUS-Activities, MSIS-29)

Data analysis:

The primary outcome is the ARR which is defined as the average number of confirmed relapses per year (i.e., the total number of confirmed relapses divided by the total days in the study multiplied by 365.25). For the primary analysis, the number of relapses will include all the confirmed relapses experienced during the study. The time spent in the study will correspond to the observation period for all the relapses from first dose on study drug to end of study.

The 2 doses of fingolimod will be tested hierarchically versus glatiramer acetate in a step-down procedure. For each of the 2 fingolimod doses, the null hypothesis is that there is no difference in the ARRs between patients treated with fingolimod and patients treated with glatiramer acetate versus the alternative hypothesis that there is a difference between the 2 treatment groups. The null hypothesis will be rejected if the observed P value for the between-treatment comparison is less than the significance level as specified in multiplicity adjustment procedure.

H_{01}: $\mu_{\text{FTY 0.5 mg}} = \mu_{\text{glatiramer acetate}}$ versus H_{A1}: $\mu_{\text{FTY 0.5 mg}} \neq \mu_{\text{glatiramer acetate}}$

H_{02}: $\mu_{\text{FTY0.25 mg}} = \mu_{\text{glatiramer acetate}}$ versus H_{A2}: $\mu_{\text{FTY0.25 mg}} \neq \mu_{\text{glatiramer acetate}}$

No formal hypothesis will be tested between the 2 fingolimod doses because the study is not powered to detect a difference in treatment effect between these doses.

The hypotheses will be tested using a negative binomial regression model with log link, using treatment, number of relapses in the previous year before study enrollment, baseline EDSS, and baseline number of Gd-enhancing T1 lesions and country (or region) as covariates. In the analysis, the response variable is the number of confirmed relapses for each patient. The patient's time in the study (natural log of time in years) is used as an offset variable to obtain the ARR, adjusted for the varying lengths of patient's time in the study (time in years). The treatment effect of fingolimod doses versus glatiramer acetate will be presented as an ARR ratio with corresponding 95% confidence intervals and p values.

To adjust for multiple testing in this study, a step-down hierarchical testing procedure will be used. There are 2 hypotheses being tested (H01, H02). Because it is highly likely that the approved dose of fingolimod (0.5 mg) is more efficacious than the lower dose (0.25 mg), the approved dose of fingolimod is initially tested against glatiramer acetate at a 2-sided significance level of 0.05. Only if this initial test H01 is rejected will H02, the low dose of fingolimod, be tested against glatiramer acetate also at a 2-sided significance level of 0.05.

The sample size calculations and power considerations follow the method outlined in Keene et al., 2007 with a constant dispersion parameter k (k=0.2231). The power of the study was evaluated under various ARR assumptions and various dropout patterns based on the cumulative literature on glatiramer acetate and the Novartis data on fingolimod. The anticipated ARR for patients treated with 0.5 mg fingolimod is $\mu_{\text{FTY 0.5 mg}}=0.195$, the ARR for those treated with glatiramer acetate is $\mu_{\text{glatiramer acetate}}=0.30$. Therefore, the estimated ARR reduction for fingolimod 0.5 mg versus glatiramer acetate is 35%. Assuming a dropout rate of 15% based on data from this D2312 study (status Feb-2015), the total 1960 randomized patients with approximately 847 patients in the fingolimod 0.25-mg group, 615 patients in the fingolimod 0.5-mg group, and 498 patients in the glatiramer acetate 20-mg group will achieve approximately 90% statistical power for the comparison of fingolimod 0.5 mg versus glatiramer acetate at a 2-sided significance level of 0.05. This calculation takes into account that patients who discontinue prematurely from the study can participate with partial data to the primary endpoint.

Fingolimod 0.25 mg has never been studied in a clinical trial in MS. Based on PK/PD modeling results, it is anticipated that the ARR in patients treated with fingolimod 0.25 mg is approximately 15% higher than in those treated with fingolimod 0.5 mg. In line with the PK/PD modeling, the anticipated ARR in patients treated with fingolimod 0.25 mg is $\mu_{\text{FTY 0.25 mg}}=0.225$, which corresponds to a 25% reduction in ARR in patients treated with fingolimod 0.25 mg compared to those treated with glatiramer acetate. Following the multiplicity adjustment procedure, the power to detect a 25% reduction in ARR for patients treated with fingolimod 0.25 mg compared with patients treated with glatiramer acetate is approximately 68% at a 2-sided significance level of 0.05, if the primary objective for the fingolimod 0.5-mg dose can be rejected first. A formal comparison between the two fingolimod doses is not intended, as the study is not powered for such a comparison.

The statistical software R (Version 2.13.1, open source) and the R library packages "MASS" and "PSCL" were used for sample size calculations and power analysis.

1 Introduction

1.1 Background

Multiple sclerosis (MS) is a chronic inflammatory autoimmune disease of the central nervous system (CNS) causing pronounced neurological disability in young adults, primarily women, with disease onset typically occurring between the ages of 20 and 40 years. The approximate prevalence rate of MS in the United States is 400,000 (0.1%).

Typically recurrent acute episodes (relapses) of neurological symptoms, which are followed by a complete or partial recovery, can be observed during the relapsing-remitting multiple sclerosis (RRMS) disease course. Approximately 50% of these patients progress to secondary progressive MS (SPMS) within 10 years and 90% within 25 years. Apart from these initially relapsing forms of MS, 10% to 15% of patients present with primary progressive MS (PPMS), which is characterized by steady deterioration of impairment without prior experience of relapses (Keegan et al, 2002).

Fingolimod (FTY720) Gilenya® is an oral, once-daily immunomodulatory drug that has been approved for the treatment of relapsing MS in the United States, Europe, and other countries. Pharmacologically, fingolimod, after phosphorylation to fingolimod-phosphate (fingolimod-P) targets a class of G protein-coupled receptors, which bind the pleiotropic sphingolipid mediator sphingosine 1-phosphate (S1P) and acts in large part by down-modulating S1P/S1P receptor responses in the immune and the central nervous systems. It causes a reversible sequestration of a proportion of CD4+ and CD8+ positive T cells and B cells from blood and spleen into lymph nodes and Peyer's patches, apparently without affecting many of the functional properties of these cells. Under normal circumstances, T cells selectively require S1P1 activation for emigration from the thymus, and both T and B cells require this receptor for egress from peripheral lymphoid organs. Fingolimod-P acts as a super-agonist of the S1P1 receptor on lymphocytes, inducing its uncoupling/internalization and intracellular lysosomal degradation. The internalization and degradation of S1P1 renders these cells unresponsive to S1P, depriving them of the obligatory signal to egress from lymphoid organs and recirculate to peripheral inflammatory tissues. As a consequence, autoaggressive T cells remain trapped in the lymphoid system, i.e., in the autoantigen-draining cervical lymph nodes in experimental autoimmune encephalomyelitis MS, which reduces their recirculation to the CNS and abrogates central inflammation.

The lymphocytes trapped by fingolimod in the LNs contain the pro-inflammatory T-helper (CD4+) IL-17 producing lymphocyte subset (Th17): A T-helper subset which is derived from naïve T cells when these cells are stimulated in the presence TGF-β, IL-1β and IL-23. Th17 cells manifest a phenotype of increased IL-17 production. Th17 cells have been shown to play an important role in the induction of autoimmune disease, but are crucial for the immune response against fungi and extracellular bacteria. Regulatory T-cells (Treg) are a subset of predominantly CD4+ T-cells differentiated from naïve T-cells in the periphery after stimulation with TGFβ and antigen (iTreg) or generated in the thymus (nTreg); other regulatory T-cell subtypes exist, including CD8+ Tregs. Tregs play an important role in the regulation of immune function and prevention of autoimmune disease. A balance between Th17 and Tregs appears crucial for the maintenance of immune homeostasis.

In EAE, among other T-cells, Th17 cells transmigrate efficiently across the blood-brain-barrier (BBB), disrupt BBB tight junctions, highly express granzyme B and kill human neurons, and promote CNS inflammation through additional CD4+ T-cell recruitment (Kebir 2007). Accordingly, large numbers of Th17 cells are found in brain tissues from MS patients, particularly in acute and chronic active lesions (Tzartos 2008). Analysis of blood T-cells from fingolimod-treated MS patients now revealed strikingly reduced numbers of IL-17-producing Th17 cells in circulation (Mehling 2010), suggesting that fingolimod directly abrogates the inflammatory Th17 axis in MS.

Since fingolimod has a preferential impact on the levels of Th17 T-helper cells, the investigation of the counts and relative proportions of Th17 cells as compared to T-regulator (Treg) cells is of relevance for the determination of the effects of the drug on immune homeostasis, both in terms of immune defense as well as suppression of the auto-immunity.

In two Phase III studies in patients with RRMS fingolimod has demonstrated a significantly superior efficacy over current standard therapy with interferon (IFN) beta-1a intramuscular (IM) (standard of care) and over placebo, respectively:

In the one-year D2302 (TRANSFORMS) study in 1292 patients with RRMS, an active comparator trial against an established standard of care, fingolimod significantly reduced annualized relapse rates (ARR) by 52% (0.5 mg dose, ARR 0.21) and 38% (1.25 mg dose, ARR 0.26) vs. IFN beta-1a IM (ARR 0.43). These findings were supported by effects on disease activity as measured in brain magnetic resonance imaging (MRI) (Cohen et al, 2010).

- Results from the 2-year placebo-controlled D2301 (FREEDOMS) study in 1272 patients with RRMS show that fingolimod reduced the relapse rate by 54% for the 0.5 mg dose (ARR 0.18) and 60% for the 1.25 mg dose (ARR 0.16) compared to placebo (ARR 0.40; both comparisons $p<0.001$). In addition, fingolimod reduced the risk of progression of disability by 30 - 37% for patients on 0.5 mg ($p=0.024$) and 32 - 40% for those on 1.25 mg ($p=0.017$) compared to placebo over two years. These findings were supported by positive effects on brain lesions as measured by MRI scan (Kappos et al, 2010).

The safety profile of fingolimod has been well characterized in the MS clinical development program. As of 29 February 2012, the MS clinical trials exposure in global and local studies is estimated to be approximately 16,500 patient-years in more than 10,000 MS patients. For updated exposure and safety information, please refer to current investigator brochure.

The safety profile observed in the fingolimod MS clinical development program can be summarized as follows:

- The overall incidence of adverse events (AEs) leading to discontinuation of study drug in [Study CFTY720D2301] was comparable for the fingolimod 0.5 mg/day (7.5%) and placebo group (7.7%) but higher in the fingolimod 1.25 mg/day group (14.2%). The overall incidence of serious adverse events (SAEs) was comparable between fingolimod groups (11.9% and 10.1% for fingolimod 1.25 mg/day and fingolimod 0.5 mg/day, respectively) and placebo (13.4%).
- Specific AEs that were reported more commonly in MS patients treated with fingolimod than placebo included elevations of liver enzymes, in particular increases in alanine aminotransferase (ALT) and γ-glutamyltransferase (GGT), reductions in white blood cell (WBC) counts (lymphocytes and total WBC count), transient bradycardia after the first

dose of fingolimod, macular edema, hypertension, dyspnea, bronchitis, and diarrhea. The AEs most prominently associated with fingolimod treatment (eg, liver enzyme elevations, bradycardia, and macular edema) appeared to show a dose response. There were no AEs that appeared to be specifically related to long-term treatment with fingolimod.

- In general, the AE profile of fingolimod in MS patients did not depend on gender, age, or previous treatment with disease-modifying drugs. The only exception was liver enzyme elevations, which were more frequent in male patients than in female patients treated with fingolimod.
- The overall incidence of infections, including serious infections, was similar in the fingolimod treatment groups and the comparator arms (IFN or placebo) in both completed Phase III studies. A slightly higher frequency of lower respiratory tract infections (primarily bronchitis) was observed in fingolimod-treated patients, with an apparent dose effect. The current data showed no clear relationship between lymphocyte count and the incidence of infections on fingolimod treatment.
- The accumulated data from the MS program do not show an association of fingolimod therapy with the development of malignancies. In the FREEDOMS study, more cancers were seen in the placebo group than in the treated groups. Incidence estimates for various forms of skin cancer (and other malignancies) from pooled safety data are comparable between treatment groups and placebo. There appears to be no increased risk with greater duration of exposure (Kappos et al, 2010).
- Initiation of fingolimod treatment has been associated with atrioventricular (AV) conduction delays usually as first-degree AV blocks (prolonged PR interval on electrocardiogram [ECG]). Second-degree AV blocks, usually Mobitz Type I (Wenckebach), have been observed in less than 0.5% of patients receiving fingolimod 0.5 mg in clinical trials. The conduction abnormalities typically were transient, asymptomatic, usually did not require treatment, and resolved within the first 24 hours on treatment.
- Data on the safe use of fingolimod in pregnancy or with breast-feeding are limited, although preclinical data suggest risk to the fetus and newborn is possible.

In the post-marketing setting, isolated delayed onset events, including transient asystole and the 1 unexplained death, within 24 hours of the first dose, have occurred. These events have been confounded by concomitant medication and/or pre-existing diseases and the relationship to fingolimod is uncertain. It is recommended that fingolimod not be administered to patients with a history of cardiovascular or cerebrovascular disease or to those patients taking heart-rate lowering medication.

Glatiramer acetate consists of the acetate salts of synthetic polypeptides, containing 4 naturally occurring amino acids: L-glutamic acid, L-alanine, L-tyrosine, and L-lysine. The mechanisms by which glatiramer acetate acts in patients with MS are not fully understood. However, it is thought to act by modifying immune processes believed to be responsible for the pathogenesis of MS. This hypothesis is supported by findings of studies that have been carried out to explore the pathogenesis of experimental allergic encephalomyelitis.

Evidence supporting the effectiveness of glatiramer acetate (20 mg/day) in decreasing the frequency of relapses in patients with RRMS comes from 2 placebo-controlled studies. In a single centre study in 50 patients randomized equally to glatiramer acetate or placebo, the

average number of relapses per patient over 24 months was reduced from 2.7 on placebo to 0.6 in the glatiramer acetate arm (Bornstein et al, 1987). In a larger placebo-controlled multicentre study in 251 patients a reduction of the 2-year relapse rate from 1.68 on placebo to 1.19 on glatiramer acetate was observed (Johnson et al, 2001). Results in both studies were supported by beneficial effects of glatiramer acetate on MRI markers of inflammatory disease activity and on other relapse-related endpoints, as compared to placebo.

In addition, 3 trials comparing high-dose IFN with glatiramer acetate were conducted. In the REGARD (Rebif® versus glatiramer acetate in patients with RRMS) open-label study in 764 patients equally randomized to the two treatment arms, there were no significant differences between groups treated with 44 µg of subcutaneous (SC) IFN β-1a 3 times a week and 20 mg of SC glatiramer acetate daily after 96 weeks of treatment in the time of the first relapse, the annualized relapse rate (IFN β-1a 0.30; glatiramer acetate 0.29), or magnetic resonance imaging (MRI) outcomes (number and change in volume of T2 active lesions) (Mikol et al, 2008).

The dose-blinded BEYOND study (N = 2244 in a 1:2:2 ratio) compared 20 mg SC glatiramer acetate with 250 µg and 500 µg of SC IFN beta-1b every other day. Results showed that the ARR was similar between groups (IFN beta-1b SC 500 µg 0.33; IFN beta-1b SC 250 µg 0.36; glatiramer acetate 0.34), with no significant difference in the time of the first relapse or the proportion of patients remaining relapse free. For MRI data, there were no significant differences in gadolinium (Gd)-enhancing lesions, T1 lesions, or normalized brain volume between groups. There were significant differences in the cumulative number of new T2 lesions between the patients treated with IFN and glatiramer acetate, favouring IFN (O'Connor et al, 2009).

In the BECOME study in 75 patients with RRMS, efficacy of treatment with IFN beta-1b and glatiramer acetate was assessed by monthly MRI and relapse rate for up to 2 years. There were no significant differences in the number of combined active lesions on MRI between the 2 groups. Correspondingly, the ARR between the 2 treatment groups did not show significant differences (IFN beta-1b 0.37; GA 0.33) (Cadavid et al, 2009).

1.2 Purpose

The purpose of this study is to compare the efficacy and safety of fingolimod 0.50 mg and fingolimod 0.25 mg to glatiramer acetate (20 mg) for the treatment of patients with RRMS as part of a postapproval commitment for the FDA.

A substudy will be conducted in a few select sites in approximately 24 patients to assess the impact of chronic fingolimod treatment on the proportions of Th17 and Treg cells in the blood of MS patients chronically treated with fingolimod as part of a post-approval commitment for the CHMP of the European Medicines Agency.

2 Study objectives

2.1 Primary objective

The primary objective is to demonstrate that at least one dose (tested hierarchically 0.5 mg followed by 0.25 mg) of fingolimod is superior to glatiramer acetate 20 mg SC in reducing the ARR up to 12 months in patients with relapsing-remitting MS.

2.2 Secondary objectives

To evaluate in fingolimod 0.5 mg, fingolimod 0.25 mg, and glatiramer acetate 20 mg groups:

- the change from Baseline in brain volume at Month 12 or end of study
- the number of active T2 lesions (new or newly enlarging lesions compared with Baseline) at Month 12 or end of study.
- the proportion of patients free of new or newly enlarging T2 lesions compared to Baseline at Month 12 or end of study
- the change from Baseline in T2 lesion volume at Month 12 or end of study
- the change from Baseline in the number and volume of T1 hypointense lesions at Month 12 or end of study
- the number and volume of Gd-enhancing T1 lesions at Month 12 or end of study
- the safety and tolerability up to Month 12
- the change from baseline in treatment satisfaction as measured by the Treatment Satisfaction Questionnaire for Medication (TSQM) at Month 12 or end of study

2.3 Exploratory objectives

- To explore the pharmacokinetics (PK) of fingolimod and fingolimod-phosphate
- To explore the effect of fingolimod 0.5 mg, fingolimod 0.25 mg, and glatiramer acetate on:
    - Multiple Sclerosis Functional Composite (MSFC) and Symbol Digit Modalities Test (SDMT) scores up to Month 12
    - The proportion of relapse-free patients at Month 12
    - The time to first relapse
    - The severity of relapses as assessed by the proportion of patients with mild, moderate or severe relapse, with steroid use, with hospitalization, or with incomplete recovery up to 12 months
- The performance of daily activities as measured by the Patient Reported Indices in Multiple Sclerosis (PRIMUS-Activities) instrument at Month 12 or end of study in selected countries
- Quality of life (QoL) as assessed by the change from Baseline in the Multiple Sclerosis Impact Scale (MSIS-29) at Month 12 or end of study

2.4 Objectives of the immune-cell substudy

Primary objective:

- To measure the effect of fingolimod treatment on the ratio of Th17 and Treg cells to total lymphocyte number upon reaching steady state and under chronic exposure

Secondary objectives:

To evaluate the following in MS patients chronically treated with fingolimod:

- The time course of change in Th17 and Treg proportion over one year of fingolimod 0.5 mg and fingolimod 0.25 mg treatment
- The effect of chronic fingolimod 0.5 mg and 0.25 mg treatment on the ratio of CD4+ and CD8+ T-cells

Exploratory objectives

- To explore the effect of chronic fingolimod 0.5 mg and 0.25 mg treatment on the proportions of Th17 and Treg cells
- To explore the effect of chronic fingolimod 0.5 mg and 0.25 mg treatment on the total number of CD4+ and CD8+ T-cells
- To explore the time course of change in relative proportions of Th17 and Treg cells over 1 year of fingolimod 0.5 mg and 0.25 mg treatment
- To assess the relationship between T cell subsets stratified based on CD45RA and CCR7 expression and relative to CD4 lymphocyte counts
- To assess the relationship between PK measures and immune subset pharmacodynamic (PD) measures noted above
- To examine chronic fingolimod treatment effects on expression of selected mRNA molecules that are (i) involved in Th17 and Treg differentiation, (ii) implicated as abnormal in MS immune cells, and (iii) implicated as potential markers of response to therapy
- To measure serum levels of molecules that reflect the balance between pro- and anti-inflammatory immune responses, particularly Th17 and Treg responses (possibly including IL-1b, IL-2, IL-6, IL-7, IL-10, IL-15, IL-22, and TGF-β), and molecules that may reflect CNS injury (possibly including neurofilament light chain and heavy chain) and repair (such as brain-derived neutrotrophic factor)
- To explore the effect of chronic fingolimod 0.5 and 0.25 mg treatment on B cell proportions and relationship to absolute lymphocyte counts

3 Investigational plan

3.1 Study design

This is a multicenter, randomized, rater- and dose-blinded study to compare the efficacy and safety of 0.25 mg and 0.5 mg of fingolimod with glatiramer acetate 20 mg SC in patients with

RRMS. Originally, approximately 2550 patients with RRMS were to be randomized, which has been reduced to a total of 1960 patients as per protocol amendment 2.

This study will consist of 3 periods:

- Screening Period: up to 45 days for all patients
- Treatment Period: 12 months of glatiramer acetate 20 mg, fingolimod 0.25 mg, or fingolimod 0.5 mg
- Follow-up Period will occur 3 months (12 weeks) after the last dose of study drug for all patients

The study design is presented in Figure 3-1.

Figure 3-1 Study design

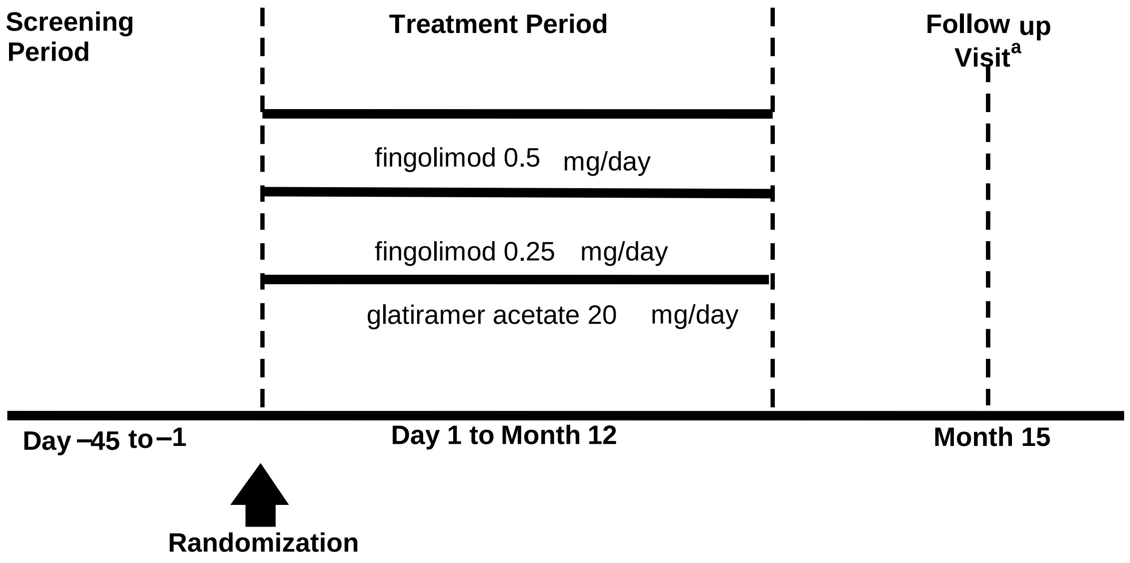

a. Follow-up will occur 3 months (12 weeks) after the last dose of study drug for all patients.

The assessment schedule is presented in Table 6-1. After signing the informed consent, patients will enter the Screening Period to determine eligibility for the study. After inclusion/exclusion criteria are reviewed again and after study assessments are conducted, patients will enter the Treatment Period and will be randomly assigned into one of the three groups (for details about the randomization, see Section 9.6 - Sample size calculation):

- Group 1 will receive fingolimod 0.5 mg orally once a day for up to 12 months
- Group 2 will receive fingolimod 0.25 mg orally once a day for up to 12 months
- Group 3 will receive glatiramer acetate 20 mg subcutaneously once a day for up to 12 months

Patients will take their first dose of study drug at Visit 2. Thereafter, patients in each fingolimod treatment arm will take 1 capsule orally once a day for 12 months. Patients in the

glatiramer acetate treatment arm will self administer an SC injection of 20 mg of glatiramer acetate once a day for 12 months. In order to ensure patient safety during the first dose administration, patients will take the first dose at the study sites and procedures in the protocol must be followed for all patients receiving oral capsules as outlined in the first-dose monitoring instructions (Section 5.5.5). First-dose monitoring is to be conducted by the treating physician.

Starting at Visit 3/Month 1, all visits in the Treatment Period have a visit window of ±5 days. Sites are encouraged to comply with this visit window as much as possible.

The end-of-study follow-up visit will occur 3 months (12 weeks) after the last dose of study drug administration (for patients who complete the study and for patients who prematurely discontinue study).

An immune-cell substudy will be initiated in approximately 48 patients at Screening to obtain approximately 24 patients randomized to fingolimod at approximately 10-15 sites with the laboratory capabilities to assess immune-cell subsets as necessary for this protocol. The sample size for the immune-cell substudy may be re-evaluated after the data analysis of the samples from the 24 fingolimod patients has been completed.

At the screening visit, patients at selected sites who provide separate written informed consent for this substudy will provide a 20 ml blood sample for the substudy. At the time of the randomization visit (Day 1), patients randomized to fingolimod who are participating in the substudy will provide a 120 ml blood sample prior to administration of the first dose of study drug. Patients randomly assigned to receive glatiramer acetate will be withdrawn from the substudy and will not provide any additional blood samples for the substudy.

Further samples for this study will be drawn at the Month 3 visit and the Month 12/End of Treatment (EOT) visit. If patients decide to discontinue study drug, a further sample of 120 ml should be drawn at the Follow-up visit 3 months after study drug discontinuation.

Handling and shipping of the blood samples will take place at the sites according to the standardized laboratory protocols. The evaluating physician will remain blinded to the results of the immune-cell assessments.

3.2 Rationale of study design

A randomized parallel-group design has been chosen as it provides an unbiased assignment to treatment and allows the direct comparison of efficacy, tolerability, and safety of the 3 treatment arms. A double-blind design was considered not suitable for this study as it would require double-dummy treatment and thus daily sham injections for the two fingolimod arms. Daily sham injections for the majority of study patients in a study of 12-month duration are not considered to be ethical.

The confirmation of relapses as the primary outcome measure by a blinded rater, and the dose blinding between the two doses of fingolimod, will provide sufficient protection against bias, as will the full blinding of all MRI endpoints. ARR has been selected as primary endpoint as it represents a clinically relevant measure of disease activity and allows determination of treatment effect within 12 months.

Blood sampling for the regulatory immune-cell substudy in all substudy participants at Screening and at subsequent visits described in Table 6-1 in patients randomized to fingolimod will allow the assessment of change in the absolute counts and proportions of immune-cell subsets of relevance to the pathophysiology of MS and immunosurveillance under chronic treatment with fingolimod, as compared to the baseline assessment.

3.3 Rationale of dose/regimen, duration of treatment

In previous Phase III studies in patients with RRMS, the doses of fingolimod 0.5 mg and fingolimod 1.25 mg were tested. Both doses showed similar efficacy in terms of relapse rate reduction, imaging parameters, and disability progression. The fingolimod 0.5-mg dose demonstrated a favorable safety profile compared with the fingolimod 1.25-mg dose based on the incidence of specific AEs of interest and the overall study drug discontinuation rate.

The minimal effective dose may not have been identified in the clinical development program, although the exposure-response model based on the ARR and T2 lesion count suggests that fingolimod 0.5 mg lies at the inflection point of the dose-response curve and that further reduction of the dose would lead to a reduction in treatment effect. Based on discussions with the FDA, the sponsor agreed to conduct further efficacy and safety assessments using a lower dose of fingolimod, with the 0.25 mg dose being the agreed-upon dose to study.

A 1-year treatment duration is suitable to detect the anticipated differences in treatment effect on relapse rate between drugs in this study.

3.4 Rationale for choice of comparator

In this study, the efficacy and safety of fingolimod 0.25 mg and fingolimod 0.5 mg will each be compared to glatiramer acetate 20 mg SC. An active comparator with established efficacy has been chosen as control for this study since placebo treatment for a duration of 1 year is not considered ethically acceptable, given the availability of several disease-modifying drugs, including fingolimod, for treatment of the target population of this study.

Glatiramer acetate is the most commonly prescribed disease modifying drug (DMD) in the United States and several other countries. Therefore it represents an acceptable alternative to treatment with fingolimod and an appropriate comparator in this study.

For the immune-cell substudy blood samples are only needed from patients treated with fingolimod, since the effect of fingolimod on immune-cell subset counts and proportions will be described as changes with respect to the pre-treatment baseline.

3.5 Purpose and timing of interim analyses/design adaptations

Not applicable.

4 Population

The study population will consist of adult male and female patients with RRMS who meet all of the inclusion criteria and none of the exclusion criteria defined in Section 4.1 and Section 4.2, respectively. Treatment-naïve patients and patients previously treated with disease-modifying therapies for MS, with the exception of S1P modulator therapy, are eligible

to participate in the study. Patients being treated with first-line DMDs at the screening visit can continue drug intake up to the day before Day 1 of this study (i.e., there is no need for a washout period).

The original anticipated total number of patients to be screened was approximately 3190, with approximately 2550 patients randomly assigned to the study treatment arms in a ratio of 1:1:1 under the assumption of a 20% screening failure rate. In protocol amendment 2, the anticipated total number of patients to be screened is reduced to approximately 2610. The screening failure rate is estimated to be 25% and a total of 1960 randomized patients are targeted. After the introduction of protocol amendment 2, and IRB approval at all sites in all countries, a new randomization ratio of 5:3:2 will be used for the 3 treatment arms of fingolimod 0.25 mg, fingolimod 0.5 mg, and glatiramer acetate 20 mg.

Refer to Section 9.6 (Sample Size Calculations) for details.

The inclusion and exclusion criteria will be assessed at Screening and Randomization Visit (before patient is randomly assigned to treatment using an interactive voice response system [IVRS]). The results of all screening assessments must be available before randomization. A subject who is under screening but has not been enrolled due to failure to meet the inclusion criteria or failure to be randomized within the specified time per protocol, may be considered for re-screening if a change in the patient's medical status or a modification of the study's eligibility criteria makes the patient potentially eligible. Re-screening will be allowed on a case-by-case basis upon approval by the study Medical Monitor. A new informed consent form should be signed in case of re-screening and a new number will be assigned to the subject. The required re-screening procedures should be discussed with the Medical Monitor.

4.1 Inclusion criteria

Patients eligible for inclusion in this study have to fulfill all of the following criteria:

1. Written informed consent must be obtained before any assessment is performed (a separate substudy-specific informed consent must be obtained before any substudy-specific assessments are performed)
2. Male and female patients 18 to 65 years of age, inclusive
3. Patients with RRMS, as defined by 2010 revised McDonald criteria (Appendix 2)
4. Patients must be neurologically stable with no onset of relapse within 30 days of randomization
5. Patients with at least 1 documented relapse during the previous year or 2 documented relapses during the previous 2 years before randomization
6. Patients with an Expanded Disability Status Scale (EDSS) score of 0 to 6.0, inclusive, at Screening. A score of 6.0 indicates unilateral assistance (cane or crutch) required to walk at least 100 meters with or without resting.

4.2 Exclusion criteria

Patients fulfilling **any** of the following criteria are not eligible for inclusion in this study:

1. Patients with a history of malignancy of any organ system (other than cutaneous basal cell carcinoma) in the last 5 years that do not have confirmation of absence of a malignancy prior to randomization.

2. Patients with an active chronic disease (or stable but treated with immune therapy) of the immune system other than MS (e.g., rheumatoid arthritis, scleroderma, Sjogren's syndrome, Crohn's disease, ulcerative colitis) or with a known immunodeficiency syndrome (HIV-antibody positive, AIDS, hereditary immune deficiency, drug-induced immune deficiency).
3. Patients who have been treated with:
   - Intravenous (IV) immunoglobulin (Ig) within 4 weeks before randomization.
   - Immunosuppressive/chemotherapeutic medications (e.g., azathioprine, cyclophosphamide, methotrexate) within 6 months before randomization.
   - Natalizumab within 2 months before randomization.
   - Previous treatment with lymphocyte-depleting therapies (e.g. rituximab, alemtuzumab, ofatumumab, ocrelizumab, or cladribine) within 1 year before randomization.
   - Previous treatment with mitoxantrone within 6 months before randomization.
   - Use of teriflunomide within 3.5 months prior to randomization, except if active washout (with either cholestyramine or activated charcoal) was done. In that case plasma levels are required to be measured and be below 0.02 mg/L before randomization.

   No washout period is necessary for patients treated with dimethyl fumarate, IFN beta, or glatiramer acetate. Patients being treated with dimethyl fumarate, glatiramer acetate, or IFN beta at the screening visit can continue drug intake up to the day before Day 1 of this study (i.e., there is no need for a washout period).
4. Patients who have been treated with systemic corticosteroids or adrenocorticotropic hormones in the past 30 days prior to the screening MRI procedure.
5. Patients with uncontrolled diabetes mellitus (glycosylated hemoglobin [HbA1c] >9%) or with diabetic neuropathy.
6. Patients with a diagnosis of macular edema during Screening (patients with a history of macular edema will be allowed to enter the study provided that they do not have macular edema at Screening).
7. Patients with severe active bacterial, viral, or fungal infections.
8. Patients without acceptable evidence of immunity to varicella zoster virus (VZV) at randomization (See Appendix 3 for guidance on acceptable evidence of immunity and requirement for serologic testing).
9. Patients who have received any live or live-attenuated vaccines (including for VZV, herpes simplex, or measles) within 1 month before randomization.
10. Patients who have received total lymphoid irradiation or bone marrow transplantation.
11. Patients with any unstable medical/psychiatric condition, as assessed by the primary treating physician at each site.

12. Patients who in the last 6 months experienced any of the following cardiovascular conditions or findings in the screening ECG:
    - Myocardial infarction
    - Unstable angina
    - Stroke
    - Transient ischemic attack
    - Decompensated heart failure requiring hospitalization or Class III/IV heart failure

(Additional monitoring guidelines for patients with any pre-existing cardiovascular conditions are provided in Section 5.5.5.)

13. Patients with history or presence of a Mobitz Type II AV block, or a third-degree AV block or sick sinus syndrome, unless patient has a functioning pacemaker.
14. Patients with baseline QTc interval >500 msec.
15. Patients receiving Class Ia (e.g., ajmaline, disopyramide, procainamide, quinidine) or Class III antiarrhythmic drugs (e.g., amiodarone, bretylium, sotalol, ibutilide, azimilide, dofetilide).
16. Patients with severe respiratory disease, pulmonary fibrosis, or Class III or IV chronic obstructive pulmonary disease, or with clinically significant lung pathology on chest x-ray. Patients with controlled asthma are allowed to enter the study.
17. Patients with any of the following hepatic conditions:
    - severe hepatic injury (Child-Pugh Class C)
    - total bilirubin greater than 2 times the upper limit of the reference range, unless in context of Gilbert's syndrome
    - conjugated bilirubin greater than 2 times the upper limit of the reference range
    - aspartate aminotransferase (AST) or alanine aminotransferase (ALT) greater than 2 times the upper limit of the reference range
    - alkaline phosphatase (AP) greater than 2 times the upper limit of the reference range
    - gamma glutamyl-transferase (GGT) greater than 2 times the upper limit of the reference range
18. Patients with a screening WBC count <3500/mm^3 or lymphocyte count <800/mm^3.
19. Patients with any of the following neurologic/psychiatric disorders:
    - history of substance abuse (drug or alcohol) in the past 5 years or any other factor (i.e., serious psychiatric condition) that may interfere with the patient's ability to cooperate and comply with the study procedures
    - progressive psychiatric/neurological condition that may affect participation in the study
20. Patients who have received an investigational drug or therapy within 180 days or 5 half-lives of randomization, whichever is longer.
21. Patients with a history of hypersensitivity to any of the study drugs, to drugs of similar chemical classes, or to mannitol.

22. Pregnant or nursing (lactating) women, where pregnancy is defined as the state of a female after conception and until the termination of gestation, confirmed by a positive human chorionic gonadotropin laboratory test.
23. Women of child-bearing potential, defined as all women physiologically capable of becoming pregnant, UNLESS they are using highly effective contraception during dosing with study treatment. Highly effective contraception includes:
    - Total abstinence (when this is in line with the preferred and usual lifestyle of the patient). Periodic abstinence (e.g., calendar, ovulation, symptothermal, post-ovulation methods) and withdrawal are not acceptable methods of contraception.
    - Female sterilization (have had surgical bilateral oophorectomy with or without hysterectomy) or tubal ligation at least 6 weeks before taking study treatment. In case of oophorectomy alone, only when the reproductive status of the woman has been confirmed by follow up hormone level assessment.
    - Male partner sterilization (at least 6 months prior to Screening). For female patients on the study, the vasectomized male partner should be the sole partner for that patient.
    - Use of oral, injected, or implanted hormonal methods of contraception or other forms of hormonal contraception that have comparable efficacy (failure rate <1%), for example, hormone vaginal ring or transdermal hormone contraception, placement of an intrauterine device or intrauterine system.

    Women are considered post-menopausal and not of child-bearing potential if they have had 12 months of natural (spontaneous) amenorrhea with an appropriate clinical profile (e.g. age appropriate, history of vasomotor symptoms) or have had surgical bilateral oophorectomy (with or without hysterectomy) or tubal ligation at least six weeks prior to Baseline. In the case of oophorectomy alone, only when the reproductive status of the woman has been confirmed by follow up hormone level assessment is she considered not of child bearing potential.
24. Patients who have previously been treated with glatiramer acetate discontinued treatment due to lack of efficacy or tolerability.
25. Patients with a history of treatment with fingolimod.
26. Patients with a score of 4 or 5 on the Suicidal Ideation item of the Columbia-Suicide Severity Rating Scale (C-SSRS) within 2 years of Screening, or any “yes” on the Suicidal Behavior item of the C-SSRS at Screening.

Additional exclusion criterion for patients being considered for participation in the regulatory immune-cell substudy:

27. Ongoing infection, including mild infections such as upper respiratory tract infections or urinary tract infections.

To ensure that the study population will be representative of all eligible patients, no additional exclusions may be applied by the investigator.

5 Treatment

5.1 Investigational and control treatment

Investigational drug

- Fingolimod (FTY720) 0.5 mg capsules for oral administration once daily
- Fingolimod (FTY720) 0.25 mg capsules for oral administration once daily

Both strengths of fingolimod capsule will be identical in appearance and will be packaged in identical bottles. Medication labels will comply with the legal requirements of each country and be printed in the local language. The medication labels will supply the medication number and information on storage conditions.

Control drug

- Glatiramer acetate 20 mg SC injection once daily.

The medication labels will supply information on storage conditions.

5.2 Treatment arms

There are 3 treatment arms. In the study, patients will be randomized to fingolimod 0.5 mg, fingolimod 0.25 mg, or glatiramer acetate 20 mg (for details about the randomization, see Section 9.6 – Sample size calculation).

5.3 Treatment assignment

At the Randomization Visit, all eligible patients will be randomized via interactive voice response system (IVRS) to 1 of the three treatment arms. The investigator, who is usually the treating physician or his /her delegate, will contact the IVRS after confirming that the patient fulfills all the inclusion/exclusion criteria. The IVRS will assign a randomization number to the patient, which will be used to link the patient to a treatment arm and will specify a unique medication number for the first package of study drug to be dispensed to the patient. The randomization number will not be communicated to the caller.

The randomization numbers will be generated using the following procedure to ensure that treatment assignment is unbiased and concealed from patients and investigator staff. A patient randomization list will be produced by the IVRS provider using a validated system that automates the random assignment of patient numbers to randomization numbers. These randomization numbers are linked to the different treatment arms, which in turn are linked to medication numbers. A separate medication list will be produced by or under the responsibility of PPD using a validated system that automates the random assignment of medication numbers to study drug packs containing each of the study drugs.

The randomization scheme for patients will be reviewed and approved by PPD Biostatistics randomization team.

5.4 Treatment blinding

In order to maintain rater-blinding, all efficacy assessments will be obtained by an Independent Evaluating Physician who must not otherwise be involved with any aspects of patient care and management. Patient care and management and other aspects of the conduct of the study at the study site will be under the responsibility of the Treating Physician.

The independent evaluating physician will remain blinded until the database lock and data analysis has been completed. In order to maintain rater-blinding, all patients will be instructed to wear appropriate clothing to completely cover typical or actual injection sites before all scheduled visits and relapse-related neurologic examinations, and not to discuss their treatment or AEs (e.g., injection site reactions) with the independent evaluating physician. These instructions will also be included on the appointment reminder cards. This procedure is essential to maintain the blinding of the efficacy assessments during the study. The treating physician and all other study staff should not discuss study treatment or AEs with the independent evaluating physician in order to maintain the “rater blind.”

All patients receiving injections will be known to the treating physician to be in the glatiramer acetate treatment arm, therefore, only patients receiving oral study drug will require first dose safety monitoring visit to be performed by the treating physician.

To maintain blinding of treatment assignment, all patients will receive the same cooling bag to carry study drug.

Because of the extended duration of first-dose monitoring of patients receiving fingolimod, it is recommended that first-dose monitoring is performed, as far as possible, in a manner that prevents blinded site personnel from becoming aware of the duration of the monitoring period.

In order to maintain the blind for Novartis and PPD staff, manual review of data listings that could potentially unblind personnel will be performed by reviewers who are independent of the study team.

5.5 Treating the patient

5.5.1 Patient numbering

Each patient is uniquely identified in the study by a combination of his/her center number and patient number. The center number is assigned by Novartis and/or PPD to the investigative site. Upon signing the informed consent form, the patient is assigned a patient number by the investigator. At each site, the first patient is assigned patient number 1, and subsequent patients are assigned consecutive numbers (e.g., the second patient is assigned patient number 2, the third patient is assigned patient number 3). The investigator or his/her staff will contact the IVRS and provide the requested identifying information for the patient to register them into the IVRS. Only the assigned patient ID will be uploaded automatically in the database. Once assigned to a patient, the patient number will not be reused. If the patient fails to be randomized for any reason, the IVRS must be notified as far as possible within 2 days that the patient was not randomly assigned. The reason for not being randomized will be entered on the screening log, and the demography eCRF should also be completed.

5.5.2 Dispensing the study treatment

Each study site will be supplied with study drug for each treatment arm. One component of the packaging has a 2-part label.

For fingolimod, each part of the label contains a medication number. At all drug dispensation visits, investigator staff will identify the fingolimod study drug package to dispense to the patient by calling the IVRS and obtaining the medication number.

In countries where the label contains a medication number, site staff will identify the glatiramer acetate study drug package to dispense to the patient by calling the IVRS and obtaining the medication number. In countries where the glatiramer acetate label does not contain a medication number, glatiramer acetate will be dispensed from bulk stock at the site. The IVRS will still be involved in tracking dispensation of glatiramer acetate study drug.

Immediately before dispensing the package to the patient, investigator staff will detach the outer part of the label from the packaging and affix it to the source document (drug label form) containing that patient's unique patient number.

5.5.3 Supply, storage, and tracking of study treatment

Study treatment must be received by a designated person at the study site, handled and stored safely and properly, and kept in a secured location to which only the investigator and designated assistants have access. Upon receipt, all study drugs should be stored refrigerated according to the instructions specified on the drug labels. Clinical supplies are to be dispensed only in accordance with the protocol.

Medication labels will be in the local language and will comply with the legal requirements of each country. They will include storage conditions for the drug but no information about the patient except for the medication number.

The investigator must maintain an accurate record of the shipment and dispensing of study drug in a drug accountability ledger. Monitoring of drug accountability will be performed by the PPD field monitor during site visits and at the completion of the trial. Patients will be asked to return all unused study drug and packaging at the end of the study or at the time of study drug discontinuation.

At the conclusion of the study, and as appropriate during the course of the study, the investigator will return all used and unused study drug, packaging, drug labels, and a copy of the completed drug accountability ledger to the PPD monitor or to the address provided in the investigator folder at each site.

Blinded study drug (fingolimod) and glatiramer acetate will be provided to the study centers; no study drug will need to be purchased locally. Fingolimod and glatiramer acetate should be stored in a limited-access area, according to the instructions on the study drug label.

5.5.4 Instructions for prescribing and taking study treatment

The study drugs (fingolimod 0.5-mg and 0.25-mg capsules and glatiramer acetate 20 mg) will be dispensed at the randomization visit (Visit 2). Drug will then be dispensed at scheduled Visits 3, 4, 5, and 6.

At Visits 2, 3, 4, 5, and 6, patients randomly assigned to fingolimod 0.25 mg or 0.5 mg will receive a bottled supply of oral medication containing a sufficient number of capsules until their next scheduled visit. At Visits 2, 3, 4, 5, and 6, patients randomly assigned to glatiramer acetate will receive a package containing a sufficient number of prefilled syringes to last until their next scheduled visit.

The fingolimod study drug needs to be taken once a day with or without food. Glatiramer acetate needs to be injected subcutaneously once a day. All study drugs should be administered preferably at the same time each day.

Site personnel will need to provide training to the patients or caregivers on the correct procedure for administration of SC injections. A patient leaflet will be provided with each supply of study drug. The patient leaflet describes information related to the study drug including: storage information, precautions, and instructions for administering SC injections. This information should be reviewed with the patients to ensure that they understand the correct procedure.

Once the eligibility of a patient for entry into the Treatment Period has been confirmed based on the study inclusion/exclusion criteria, the site will contact the IVRS to obtain a randomization number to identify study drug. Before administration of the first dose of the study drug, the investigator should reconfirm a list of concomitant medications taken by the patient.

The first dose of study drug is administered at the randomization visit (Visit 2). The treating physician will monitor the first dose intake in the clinic for at least 6 hours or longer if discharge criteria are not met in those patients receiving oral study drug (fingolimod) (see Section 5.5.5 for guidance for monitoring of patients taking their first dose of the study drug). Patients receiving injectable study drug do not require prolonged monitoring in the clinic after the first dose.

Throughout the study, careful planning of patient visits is required to make sure that the patients will have enough study drug to last until the next scheduled visit.

The investigator and study personnel should promote compliance by instructing the patient to take the study drug exactly as prescribed and by stating that compliance is necessary for the patient's safety and for the validity of the study. The patient should be instructed to contact the site if he or she is unable for any reason to take the study drug as prescribed.

All doses prescribed and dispensed to the patient and all dose interruptions during the study will be recorded in the IVRS and drug administration record in the eCRF.

5.5.5 Guidelines for monitoring patients taking their first dose of fingolimod

The patient will stay at the site for a minimum of 6 hours after the first dose of fingolimod to be monitored for signs and symptoms of bradycardia. All fingolimod patients will have an ECG performed prior to dosing and at the end of the 6-hour monitoring period. (Glatiramer acetate patients will have a single ECG during the randomization visit.) The treating physician is responsible for monitoring the patient following the first intake of fingolimod, as well as managing bradycardia symptoms should they occur. He or she must review vital signs during the 6-hour monitoring, post-dose ECG and assess discharge criteria at 6 hours after dosing.

Baseline or predose ECG should be provided by the site and be available for comparison to the postdose ECG in order to determine if discharge criteria are met.

Heart rate and blood pressure should be measured before the first dose of fingolimod, then every hour for at least 6 hours thereafter (by the treating physician or an assisting nurse). Blood pressure will be measured in the supine position and then the standing position at each time point. When obtaining the predose heart rate before the first dose, the patient should be allowed to rest in the supine position for 5 minutes before taking the heart rate. The heart rate and blood pressure measurements should be repeated twice to produce 3 baseline readings in the supine position for both heart rate and blood pressure (before the first dose of fingolimod only). Heart rate and blood pressure will then be measured in the same manner (i.e., 3 baseline readings) with the patient in the standing position before the first dose of fingolimod. For comparison to the postdose heart rate values, the lowest predose supine value and lowest predose standing value of heart rate should be used. Patients should receive the first dose of fingolimod before 12:00 PM (noon) in the outpatient setting.

Patients may be discharged after 6 hours ONLY if ALL of the following criteria are met:

- supine heart rate must be at least 45 beats per minute
- heart rate must not be the lowest hourly value measured during the observation period (which would suggest that nadir may not have been reached)
- patients must have no symptoms associated with decreased heart rate or must not have received treatment for bradycardia
- ECG at 6 hours after dosing should not show any new significant abnormalities (e.g., onset second-degree or third-degree AV block, QTc ≥500 msec) other than sinus bradycardia, that were not also observed at the patient’s predose ECG

If the discharge criteria listed above are not met after 6 hours, observation must be continued until symptoms resolve.

If a patient requires treatment for bradycardia/bradyarrhythmia during the first dose observation, the patient should be monitored overnight, and the 6-hour monitoring procedure should be repeated for the second dose of study drug.

If a patient experiences a QTc interval greater than or equal to 500 msec at 6 hours after the first dose, the patient should be monitored overnight, and the 6-hour monitoring procedure should be repeated for the second dose of study drug.

Patients with some pre-existing conditions (e.g., ischemic heart disease, history of myocardial infarction, congestive heart failure, history of cardiac arrest, cerebrovascular disease, uncontrolled hypertension, history of symptomatic bradycardia, history of recurrent syncope, severe untreated sleep apnea, AV block, sinoatrial heart block) may poorly tolerate the fingolimod-induced bradycardia, or experience serious rhythm disturbances after the first dose of fingolimod. Prior to treatment with fingolimod, these patients should have a cardiac evaluation by a physician appropriately trained to conduct such evaluation, and, if treated with fingolimod, should be monitored overnight with continuous ECG in a medical facility after the first dose.

Since initiation of fingolimod treatment results in decreased heart rate and may prolong the QT interval, patients with a prolonged QTc interval (>450 msec males, >470 msec females)

before dosing or during a 6-hour observation period, or at additional risk for QT prolongation (e.g., hypokalemia, hypomagnesemia, congenital long-QT syndrome), or on concurrent therapy with QT-prolonging drugs with a known risk of torsades de pointes (e.g., citalopram, chlorpromazine, haloperidol, methadone, erythromycin) should be monitored overnight with continuous ECG in a medical facility.

Experience with fingolimod is limited in patients receiving concurrent therapy with drugs that slow heart rate or AV conduction (e.g., beta blockers, heart rate lowering calcium-channel blockers such as diltiazem or verapamil, or digoxin). Because the initiation of fingolimod treatment is also associated with slowing of the heart rate, concomitant use of these drugs during fingolimod initiation may be associated with severe bradycardia or heart block. The possibility of switching to drugs that do not slow the heart rate or AV conduction should be evaluated by the physician prescribing these drugs before initiating fingolimod. Patients who cannot switch should have overnight continuous ECG monitoring after the first dose.

In addition to protocol mandated safety assessments and monitoring procedures, additional assessments may be required as per local prescribing information and should be followed accordingly.

Patients should have written instruction on when to return to clinic and a 24-hour contact phone number to call in the event of any new or warranted symptoms (e.g., chest pain, dizziness, palpitations, syncope, nausea, vomiting). Patients should be instructed not to drive on the same day after the first dose of fingolimod administration.

Recommendations for management of bradycardia

Prior to the administration of study drug, if, in the opinion of the investigator, the patient's cardiovascular condition or medical history carry a significant risk for study participation, the patient should not be enrolled in the study, and the advice of a cardiologist should be sought.

Atropine (SC or IV) is recommended as the first line treatment of bradycardia, up to a maximum daily dose of 3 mg.

Furthermore, the common guidelines for treatment of bradycardia (e.g., ACLS guidelines) should be followed as appropriate:

- In case of clinical symptoms or hypotension, administration of atropine 1 mg, repeated administration in 3-5 minutes
- If heart rate and/or blood pressure remains unresponsive, consider administration of dopamine drip 5-20 ug/kg/min or epinephrine drip 2-10 ug/min
- Performance of transcutaneous pacing may also be considered

In the setting of decreased blood pressure, isoproterenol should be avoided/used with caution.

5.5.6 Permitted dose adjustments and interruptions of study treatment

Dose adjustments will not be allowed; however, drug interruptions will be allowed based on the judgment of the investigator. If an investigator determines that study drug should be stopped, then the SC injections or oral capsules, respectively, need to be stopped. Conditions or events that may lead to study drug interruptions based on investigator judgment and overall clinical assessment are:

- SAE
- emergency medical condition or unplanned hospitalization involving the use of prohibited medications (Section 5.5.9)
- abnormal laboratory value(s) or abnormal test or examination result(s) (e.g., ECG values, ophthalmic findings). See Appendix 3 and Appendix 4 for guidance on monitoring of patients with specific test abnormalities
- hypersensitivity to the study drug
- patient noncompliance
- vaccination

The 6-hour monitoring procedure when restarting study drug is mandatory in the following cases:

- the treatment lasted for 14 days or less and was interrupted for 1 day or more, or
- the treatment lasted for more than 14 days and less than 29 days and was interrupted for more than 7 consecutive days, or
- the treatment lasted for 4 weeks or more and was interrupted for more than 14 consecutive days.

Should a patient randomized to one of the fingolimod treatment arms interrupt the study drug as described above and should the investigator decide to reinitiate treatment with the study drug, the first dose intake at restart must take place at the study site to ensure at least 6-hour monitoring by the treating physician in a similar manner as the first intake of the study drug (see for guidelines for monitoring of patients taking their first dose of the study drug in Section 5.5.5).

A reason for the interruption of treatment and dates of interruption should be documented in the source documents as well as in the dosage administration eCRF.

5.5.7 Recommendations on treatment of multiple sclerosis relapse

A standard course of intravenous corticosteroids (methylprednisolone) on an inpatient or outpatient basis is allowed for treatment of relapses as clinically warranted. Steroid treatment should consist of 3 to 5 days and up to 1000 mg methylprednisolone/day.

Standard-of-care procedures will be followed during treatment of relapses.

Use of any oral tapering is not permitted.

The use of steroid therapy should be recorded in the use of steroids for treatment of relapses eCRF. Refer to Section 6.4.3 for the instruction on conduct of the study MRI during relapse and steroid treatment.

Investigators should consider the added immunosuppressive effects of corticosteroid therapy and increase vigilance regarding infections during such treatment and in the weeks following administration.

Should a patient show evidence or suspicion of infection, please refer to the guidance on monitoring of patients with infections outlined in Appendix 3.

Should a patient develop any neurological symptoms or signs, unexpected for MS in the opinion of the investigator or accelerated neurological deterioration, the investigator should immediately schedule an MRI and follow the guidance on monitoring of patients with symptoms or signs of neurological deterioration inconsistent with MS outlined by Appendix 3. Steroids should not be taken before conducting the unscheduled MRI.

5.5.8 Concomitant treatment

All concomitant medications taken within 30 days before Screening (Visit 1) and during the study must be recorded. Both the start date and the end date for each medication should be captured on the prior medications/significant nondrug therapies eCRF, the previous MS treatment eCRF, the concomitant medications/significant nondrug therapies eCRF, and the steroid treatment of MS relapses eCRF.

Patients taking dimethyl fumarate, glatiramer acetate, or IFN beta before study entry may continue to take this medication up to the last day before randomization. These patients will be randomly assigned to and start study drug on the day of their next scheduled dose.

A standard short course of corticosteroids (methylprednisolone IV) is allowed for treatment of relapses (Section 5.5.7). Treatment with topical corticosteroids in the eyes, ears, or nose, on the skin or through inhalation, is also allowed, if the potential systemic absorption of the corticosteroid component due to that treatment can be regarded as negligible and the condition being treated does not warrant study discontinuation per protocol. If a course of systemic corticosteroid treatment is required for treatment of uveitis, this is also acceptable.

The medications allowed for the treatment of adverse reactions and relapses are not considered study supplies and, therefore, need to be supplied by the study site.

Medications for the symptomatic treatment of MS such as baclofen, fampridine, methylphenidate, or modafinil are acceptable. As far as possible, every effort should be made to keep the dosages stable from Screening through EOT.

The investigator should instruct the patient to notify the study site about any new medications that they take after the start of the study drug. All medications and significant nondrug therapies (including physical therapy and blood transfusions) administered after the patient starts treatment with study drug must be recorded on the concomitant medications/significant nondrug therapies eCRF and steroid therapies eCRF.

5.5.9 Prohibited treatment

Use of the following treatments is NOT allowed after randomization:

- Immunosuppressive medication (e.g., cyclosporine, azathioprine, methotrexate, cyclophosphamide, mitoxantrone, cladribine)
- Other concomitant medications: immunoglobulins, monoclonal antibodies (including natalizumab), IFN beta, adrenocorticotropic hormone, other disease-modifying medications approved for MS including those approved subsequent to the start of this study

The administration of any live or live-attenuated vaccine (including for measles) is prohibited while patients are receiving fingolimod and for 8 weeks after fingolimod discontinuation.

5.5.10 Discontinuation of study treatment and premature patient withdrawal

Patients may voluntarily withdraw from the study for any reason at any time. They may be considered withdrawn if they state an intention to withdraw, fail to return for visits, or become lost to follow-up for any other reason.

If premature withdrawal occurs for any reason, the investigator must make every effort to determine the primary reason for a patient's premature withdrawal from the study and record this information on the study completion eCRF.

Patients may be withdrawn from the study for any of the following reasons:

- Withdrawal of the informed consent
- Lost to follow-up
- Withdrawal at the investigator's discretion

Patients who discontinue study drug should NOT be considered withdrawn from the study unless one of the previous reasons apply. These patients are required to follow the abbreviated schedule of assessment (see Table 6-2). Patients who discontinue the study drug should be treated according to the best standard of care. Protocol violations should not lead to patient withdrawal unless they indicate a significant risk to the patient's safety.

For patients who are lost to follow-up (i.e., those patients whose status is unclear because they fail to appear for study visits without stating an intention to withdraw), the investigator should show due diligence by documenting in the source documents steps taken to contact the patient (e.g., dates of telephone calls, registered letters). In case of death, a patient will be considered withdrawn from the study.

The appropriate personnel from the site and Novartis and/or PPD will assess whether study treatment should be discontinued for any patient whose treatment code has been broken inadvertently for any reason. Patients who discontinue study treatment should NOT be considered withdrawn from the study. A Study Drug Discontinuation Form should be completed, giving the date and primary reason for stopping study treatment. See Section 6 for the required assessments of these patients after discontinuation of study treatment. The investigator must also contact the IVRS to register the patient's discontinuation from study drug.

5.5.11 Emergency unblinding of treatment assignment for patients on fingolimod

Emergency unblinding should only be undertaken when it is essential to treat the patient safely and efficaciously. Most often, study drug discontinuation and knowledge of the possible treatment assignments are sufficient to treat a study patient who presents with an emergency condition. Emergency code breaks are performed using the IVRS. When the investigator contacts the system to unblind a patient, he or she must provide the requested patient identifying information and confirm the necessity to unblind the patient. The investigator will then receive details of the drug treatment for the specified patient and a fax or e-mail confirming this information. The system will automatically inform the PPD monitor for the site and the clinical leader at Novartis and/or PPD that the code has been broken.

It is the investigator's responsibility to ensure that there is a procedure in place to allow access to the IVRS in case of emergency. The investigator will inform the patient how to contact his/her backup in cases of emergency when he or she is unavailable. The investigator will provide protocol number, study drug name if available, patient number, and instructions for contacting the local Novartis CPO (or any entity to which it has delegated responsibility for emergency code breaks) to the patient in case emergency unblinding is required at a time when the investigator and backup are unavailable.

5.5.12 Study completion and post-study treatment

The study will be considered completed for an individual patient when he or she completes Visit 7/EOT.

Patients who are prematurely withdrawn from the study will not be replaced.

The investigator also must provide follow-up medical care for all patients who are prematurely withdrawn from the study or must refer them for appropriate ongoing care.

5.5.13 Early study termination

The study can be terminated at any time for any reason by Novartis. Should this be necessary, the patient should be seen as soon as possible and treated as described in Table 6-2 for a prematurely withdrawn patient. The investigator may be informed of additional procedures to be followed in order to ensure that adequate consideration is given to the protection of the patient's interests. The investigator will be responsible for informing the institutional review boards (IRBs) or independent ethics committees (IECs) of the early termination of the trial.

6 Visit schedule and assessments

The assessment schedule listing all assessments and visits to be performed under this protocol is provided in Table 6-1.

Starting at Visit 3, the visit window for all study visits is ±5 days. Sites are encouraged to comply with this visit window as much as possible. Follow-up will occur within 3 months after the last dose of study drug for all patients (i.e., 3 months from the last study drug dose/EOT for patients who completed the study or 3 months from the last study drug dose for patients who were discontinued from the study). For any patients who permanently discontinue study drug early but decide to continue in the study, if the follow-up visit falls within the visit window for a scheduled visit, then assessments for both visits should be combined and completed. In addition to the scheduled visits, patients may have unscheduled visits following an onset of MS relapse or for other reasons as indicated in the protocol. All information should be collected per the unscheduled visit eCRF.

Table 6-1 Assessment schedule

Period	Screening	Treatment Period						FU[a]
**Visit**	**1**	**2**	**3**	**4**	**5**	**6**	**7/EOT**	
**Month**	**Day –45 to –1**	**Day 1 Randomization**	**1**	**3**	**6**	**9**	**12**	**EOT +3**
**Visit window (days)**			**±5**	**±5**	**±5**	**±5**	**±5**	**±5**
Obtain informed consent	X							
Background/demography	X							
Inclusion/exclusion criteria	X	X						
Medical history	X							
MS history/MS treatment	X							
Prior/concomitant medication	X	X	X	X	X	X	X	X
Pregnancy test (serum)	X							
Pregnancy test (urine dipstick)		X	X	X	X	X	X	X
Physical examination	X			X			X	X
Ophthalmologic examination[b]	X			X	X		X	
Dermatology examination	X						X	
Randomization		X						
Vital signs[c]/body weight	X	X	X	X	X	X	X	X
Pulmonary function tests	X				X		X	X
12-lead electrocardiogram	X	X[d]					X	
Blood chemistry and hematology	X		X	X	X	X	X	X
Retrospective analysis blood sample	X							
Pharmacokinetics					X		X	
Serology[e]	X							
Immune-cell blood sample[f] (optional)	X	X		X			X	X
First-dose monitoring		X						
Urinalysis	X	X	X	X	X	X	X	X
Study drug dispensation		X	X	X	X	X		
Study drug accountability			X	X	X	X	X	
Magnetic resonance imaging	X						X	
EDSS	X						X	X
MSFC	X						X	X
SDMT	X						X	X
MS relapse[g]	X	X	X	X	X	X	X	X
PRIMUS-Activities[h]	X				X		X	
MSIS-29	X				X		X	
TSQM	X				X		X	
C-SSRS	X	X		X	X	X	X	X
Adverse events/SAEs	X	X	X	X	X	X	X	X
Study completion form							X	

Abbreviations: C-SSRS=Columbia-Suicide Severity Rating Scale; ECG=electrocardiogram; EDSS=expanded disability status scale; EOT=end of treatment; FU=follow-up; HSV=herpes simplex virus; MS=multiple sclerosis; MSFC=multiple sclerosis functional composite; MSIS-29=multiple sclerosis impact scale; PRIMUS=patient reported indices in multiple sclerosis; SAE=serious adverse event; SDMT=symbol digits modalities test; TSQM=treatment satisfaction questionnaire for medication.

a. Follow-up will occur 3 months (12 weeks) after the last dose of study drug for all patients.

b. The ophthalmology examination will include eye history, visual acuity, dilated ophthalmoscopy, and any procedures necessary to assess macular edema. Optical coherence tomography and fluorescein angiography will be done only if needed to confirm macular edema.

c. Body temperature is collected only at Screening, Visit 7/EOT, and at the follow-up visit.

d. A 12-lead ECG will be performed before fingolimod and glatiramer acetate administration, and 6 hours after the fingolimod administration only.

e. Patients will be tested for human immunodeficiency virus, varicella zoster virus (as per guidance in Appendix 3), HSV-1, HSV-2, and rubeola virus.

f. Screening sample is 20 mL of whole blood. Day 1 (randomization) and follow-up samples are whole blood, 120 mL each, for patients randomly assigned to receive fingolimod. The Day 1 sample must be collected after randomization and before administration of the first dose of study drug. All other samples may be collected with the other study samples.

g. Patients should report symptoms indicative of a relapse at a scheduled visit or at any other time and will be instructed to immediately contact the treating physician if they develop new or reoccurring or worsening neurological symptoms. A neurological examination by the independent evaluating physician must be arranged **as soon as possible, preferably** within 7 days of the onset of symptoms.

h. Performed only in selected countries.

Table 6-2 Abbreviated schedule of assessments for patients who discontinue study drug

**Period**	**Off-Treatment**		
**Visit**	**5**	**6**	**7/EOS**
**Month**	**6**	**9**	**12**
**Visit window (days)**	**±5**	**±5**	**±5**
MS relapses[a]	X	X	X
MS treatment/steroids	X	X	X
Concomitant medications	X	X	X
Magnetic Resonance Imaging			
EDSS			X
MSFC			X
SDMT			X
PRIMUS-Activities[b]			X
MSIS-29			X
TSQM			X
C-SSRS	X	X	X
Physical exam	X		X
Ophthalmologic exam[c]			X
Dermatology examination			X
Vital signs[d]/body weight	X	X	X
Pulmonary function tests			X
Laboratory values	X[e]	X[e]	X
Immune-cell blood sample[f]			X
Adverse events	X	X	X
SAE reporting	X	X	X

Abbreviations: C-SSRS=Columbia-Suicide Severity Rating Scale; EDSS=expanded disability status scale; EOS=end of study; MS=multiple sclerosis; MSFC=multiple sclerosis functional composite; MSIS-29=multiple sclerosis impact scale; PRIMUS=patient reported indices in multiple sclerosis; SAE=serious adverse event; SDMT=symbol digits modalities test; TSQM=treatment satisfaction questionnaire for medication.

a. Patients should report symptoms indicative of a relapse at a scheduled visit or at any other time and will be instructed to immediately contact the treating physician if they develop new or reoccurring or worsening neurological symptoms. A neurological examination by the independent evaluating physician must be arranged **as soon as possible, preferably** within 7 days of the onset of symptoms.

b. Performed only in selected countries.

c. The ophthalmology examination will include eye history, visual acuity, dilated ophthalmoscopy, and any

procedures necessary to assess macular edema. Optical coherence tomography and fluorescein angiography will be done only if needed to confirm macular edema.

d. Body temperature is collected only at Visit 7/EOS.

e. Urinalysis only.

f. Whole blood sample is 120 mL.

6.1 Information to be collected on screening failures

Those patients who have signed informed consent and fail to meet all inclusion/exclusion criteria will not be randomized and will be deemed screening failures. A reason will be documented on the screening log eCRF.

6.2 Patient demographics/other baseline characteristics

Demographic data will be collected and recorded in the demography eCRF including date of birth, sex, and race. Multiple sclerosis history and previous MS treatment will be recorded in the MS history and previous MS treatment eCRF pages, respectively. The majority of evaluations (Table 6-1) will be performed at Screening. Only review of inclusion/exclusion criteria, vital signs, ECG, and urine pregnancy test (if applicable) will be repeated at Day 1, (randomization). The baseline laboratory values should be available before randomization (part of assessment of inclusion/exclusion criteria).

The treating physician will ask about relevant diseases of the following organs and organ systems: skin; eyes, ears, nose, throat, head and neck (including the thyroid); lungs; heart; breasts; abdomen; lymph nodes; musculoskeletal (including extremities and spine); genitourinary, gynecological organs; and rectum. History of allergies, in particular with respect to Gd-diethylenetriamine penta-acetic acid (Gd-DTPA) will be queried. Patients will be asked if they have a history of alcohol or drug abuse or a history of psychiatric disorders. Any previous disease or surgeries will also be documented. Medical history should be supplemented by review of the patient’s medical chart and/or by documented dialog with the patient’s treating physician. The source of information will be recorded in the relevant medical history/current medical conditions eCRF.

Previous MS history (including history of relapses) needs to be documented in the patient’s medical chart and/or in documented dialog with the patient’s treating physician.

Information relating to MS history will be collected in the study including: date of MS diagnosis, date of MS symptoms, eye history (e.g., optic neuritis or uveitis), MS relapse history (i.e., number of relapses since first MS symptoms and number of relapses that required steroid treatment), and history of medications used to treat MS. These data will be collected and recorded in the MS medical history, ophthalmology screening, and MS treatment eCRFs. Medications used to treat MS may include glatiramer acetate, IM IFN beta-1a, SC IFN beta-1a, IFN beta-1b, natalizumab, azathioprine, methotrexate, or any other medications used as MS treatments.

During the study medications to treat MS-related symptoms should be recorded in the concomitant medication eCRF. Multiple sclerosis history should be supplemented by review of the patient’s medical chart and/or by documented dialog with the patient’s primary treating physician.

In patients with diabetes, diabetes-specific medical history to be collected and recorded in the eCRF will include type 1 vs. type 2, date of diabetes diagnosis, antidiabetic therapy, and presence of diabetes-related complications.

6.3 Treatment exposure and compliance

In order to collect accurate information about the study drug exposure, the following records should be maintained for each randomly assigned patient: records of study drug dispensed and returned, dosages administered (in blinded fashion) and intervals between visits. These data should be transcribed on the dosage administration record eCRF.

Compliance will be assessed by the investigator and/or study personnel at each visit using capsule and syringe counts and information provided by the patient. A monitor will perform and document drug accountability during site visits and at the end of the study.

6.4 Efficacy

6.4.1 Multiple sclerosis relapse

Patients should be instructed to report symptoms indicative of an MS relapse at any time, regardless of whether a visit is scheduled. A patient must be instructed to immediately contact the study site if a new, reoccurring, or worsening neurological symptom develops.

- **General definition of relapse**: appearance of a new neurological abnormality or worsening of previously stable or improving preexisting neurological abnormality, separated by at least 30 days from onset of a preceding clinical demyelinating event. The abnormality must be present for at least 24 hours and occur in the absence of fever (<37.5°C) or infection.
- **Definition of confirmed relapse**: a relapse must be confirmed by the independent evaluating physician (neurologist). It is recommended that this occur as soon as possible but no more than 7 days from the onset of symptoms. A relapse is confirmed when it is accompanied by an increase of at least half a step (0.5) on the EDSS or an increase of 1 point on 2 different functional systems (FSs) of the EDSS or 2 points on 1 of the FS (excluding bowel and bladder and cerebral FSs).

Reporting of relapse

In order to maintain rater-blinding, a special coordination between the treating physician and the independent evaluating physician (blinded rater) must be followed for relapse assessment. A patient may report symptoms indicative of a relapse at a scheduled visit or at any other time. Patients will be instructed to immediately contact the treating physician if they develop new or reoccurring or worsening neurological symptoms.

Upon reporting symptoms indicative of a relapse, the treating physician will assess whether the symptoms had onset in the presence of fever or infection. If fever or infection can be excluded, a neurological examination by the independent evaluating physician must be arranged **as soon as possible, preferably** within 7 days of the onset of symptoms. If fever or infection cannot be excluded, the neurological examination will be postponed until the fever or the infection has ceased (provided that the symptoms indicative of a relapse are still

present). Treatment with steroids should not begin before the assessment by the independent evaluating physician.

Based on results of the neurological examination (change in FS and EDSS scores) provided by the independent evaluating physician, the treating physician will record if the relapse meets the criteria for "confirmed relapse" as per protocol and assess the severity (see Table 6-3). All relapses, whether confirmed or unconfirmed, and the severity of each relapse should be recorded on the summary of MS relapses eCRF.

Table 6-3 Severity of multiple sclerosis relapse

Mild relapse	Moderate relapse	Severe relapse
EDSS increase of 0.5 point<br>or<br>1-point FS change in 1 to 3 systems	EDSS increase of 1 or 2 points<br>Or<br>2-point FS change in 1 or 2 systems<br>Or<br>1-point change in 4 or more systems	Exceeding moderate criteria

EDSS = Expanded Disability Status Scale, FS = function system.

Reference: Panitch, 2002

6.4.2 Expanded Disability Status Scale

The EDSS is a scale for assessing neurologic impairment in MS and will be used to confirm an MS relapse. It is a 2-part system including: (1) a series of scores in each of the 8 FSs and (2) steps ranging from 0 (normal) to 10 (death due to MS). The FSs are Visual, Brain Stem, Pyramidal, Cerebellar, Sensory, Bowel and Bladder, Cerebral, and other functions. Fatigue is not to be considered in determining the Cerebral Functional score.

The EDSS will be derived based on the neurological examination performed by the independent evaluating physician, a neurologist with experience with MS patients. Other qualified individuals (e.g., registered nurse or nurse practitioner) with documented experience in MS clinical trials might be considered for the role of EDSS rater if approved by Novartis' Medical Advisor. At site initiation, the EDSS rater must have received level "C" Neurostatus certification within the previous year unless the EDSS rater regularly performs the assessment, in which case the certification can be up to 2 years old. All EDSS raters must be recertified every 2 years. It is recommended that a backup EDSS rater be available at each site. The EDSS assessments will be done as detailed in Table 6-1 or Table 6-2 for patients that discontinued study drug or as needed during relapse assessments.

6.4.3 Magnetic Resonance Imaging (MRI)

All MRIs will be performed at the visits as indicated in Table 6-1 or Table 6-2 for patients that discontinued study drug.

The treating physician will be able to review all MRIs during the study. For analysis purposes, the scheduled MRIs will be reviewed and measures recorded by a blinded central MRI reader.

Restrictions for magnetic resonance imaging schedule

To avoid potential interference caused by steroids used for the treatment of MS relapses, the following restrictions apply for this study:

- In case of relapse, if an MRI is scheduled within 30 days of the initiation of steroid treatment, this MRI should be performed before steroid treatment is initiated
- No MRI scan should be performed while a patient is on steroid therapy or within 30 days after termination of steroid therapy

Scanning T1-weighted image before and after administration of contrast medium (0.1 mmol/kg Gd-DTPA) as well as T2-weighted (T2 and proton density) images will be performed.

The contrast agent may occasionally cause nausea and vomiting. Allergic reactions may also occur very rarely and, in extremely rare instances, can be potentially serious and require immediate anti-anaphylactic treatment (IV epinephrine, dopamine, steroids, etc.). The patients known to be allergic to Gd may participate in the trial with MRI assessment modified to forgo contrasted MRI images.

Before the start of the study, a radiologist and technician from each center will receive an MRI manual outlining technical implementation, image quality requirements, and MRI administrative procedures. Each site will be asked to program the MRI scanner that is designated for evaluation of the study patients and perform and submit a “dummy scan” or “dry run” to assess the image quality and to evaluate the compatibility of the electronic data carrier. Once the dummy scan has been approved by the central MRI reader, all parameter settings for the study-specific MRI sequences must remain unchanged for the duration of the study.

Data handling and evaluation

The quality of each scan performed will be assessed by the blinded MRI reader. As soon as the scan is received by the central MRI reading center, it will be evaluated for quality, completeness, and adherence to the protocol. Confirmation of MRI quality or a description of the quality problems, if detected, will be communicated to the site. If the scan is incomplete or incorrectly performed, the study center will be asked to repeat it as soon as possible. After completion of the quality check, all scans will be analyzed according to the MRI protocol.

6.4.4 Multiple Sclerosis Functional Composite (MSFC)

The MSFC is a composite measure encompassing the major clinical dimensions of arm, leg, and cognitive function. The MSFC consists of 3 objective quantitative tests of neurological function:

- Nine hole peg test (arm dimension) measurement: right and left arm scores; metric: time in seconds to insert and remove 9 pegs
- Timed walk (leg dimension); measurement: a walk of 25 feet; metric: time taken in seconds
- PASAT-3 min (cognitive function); measurement: paced auditory serial addition test, 3 minute version; metric: number of correct answers

The scores for these 3 components are combined to create a single score that is used to detect changes over time. This is done by creating z-scores for each component and averaging them to create the overall z-score. In general, z-scores involve comparing each test result with that found in a reference population (patient baseline data), a process called standardization. The z-score for each component is calculated by subtracting the mean of the reference population from the test result and then dividing by the standard deviation of the reference population.

The PASAT-3 min auditory test tapes will be provided to the sites in the local language. Refer to Appendix 5 for information on MSFC. Two training sessions of the PASAT must be performed during Screening (at Visit 1) as rehearsals before performing the baseline PASAT. The MSFC assessment will be performed either by the independent evaluating physician or by a qualified individual not involved in the treatment of patients.

6.4.5 Symbol Digit Modalities Test (SDMT)

The SDMT is used to measure cognitive function over time in response to treatment and will be assessed by the independent evaluating physician or qualified individual at each site at the time points indicated in Table 6-1 or Table 6-2 for patients that discontinued study drug. Using a reference key, the patient has 90 seconds to pair specific numbers with given geometric features.

6.4.6 Appropriateness of efficacy assessments

The ARR and MRI are sensitive and validated assessments to evaluate the disease activity and severity. MSFC and EDSS are common tools to assess level of disability and disease progression in the patient population that has been selected for this study. These are standard efficacy instruments which are also accepted by regulatory agencies. SDMT is a validated cognitive scale for use in MS studies and less affected by practice effect than PASAT.

6.5 Safety Assessments

Safety assessments include the following:

- Physical and neurological examination
- Vital sign measurements
- Laboratory evaluations
- ECG results
- Ophthalmologic examinations
- Pulmonary function tests (PFTs)
- Dermatological examination
- C-SSRS

6.5.1 Physical examination

A complete physical examination will be performed at visits as described in Table 6-1 or Table 6-2 for patients that discontinued study drug and will include an assessment of skin, head and neck, lymph nodes, heart, lungs, abdomen, and back, and comments on general appearance. Initial neurological examination (with fundoscopic examination) will be a part of the physical examination at Screening and if warranted by an unscheduled visit. Investigators

should ask the patient if they have any new or changed skin lesions as part of each physical examination. If skin lesions (suspected to be precancerous or cancerous) are identified during the physical examination, the patients should be referred to a dermatologist. (Also refer to Section 6.5.10 for dermatological examination).

All significant findings that are present at Screening must be reported on the relevant medical history/current medical conditions eCRF. Significant findings made after randomization that meet the definition of an AE must be recorded on the AEs eCRF.

Patients who experience new significant cardiac symptoms (e.g., congestive heart failure, new murmur, ischemic heart disease) or symptoms consistent with pulmonary hypertension (e.g., exertional dyspnea, chest pain, and syncope), should be referred to a specialist (e.g., cardiologist) for further diagnostic workup (e.g., echocardiography) and appropriate management as close as possible to the time of the onset of symptoms.

6.5.2 Vital signs

Vital signs will be collected as described in Table 6-1. These will include supine and standing heart rate and systolic and diastolic blood pressures. After the patient has been supine for 5 minutes, systolic and diastolic blood pressure will be measured 3 times using an automated validated device (e.g., OMRON) with an appropriately sized cuff. The repeat measurements will be made at 1- to 2-minute intervals, and the mean of the 3 measurements will be used. In case the cuff sizes available are not large enough for the patient's arm circumference, a sphygmomanometer with an appropriately sized cuff may be used. Heart rate and blood pressure will then be measured in the same manner with the patient in the standing position. A sudden, significant fall in blood pressure (>20 mm Hg systolic or >10 mm Hg diastolic) between 2 and 5 minutes after standing from the supine position will be defined as orthostatic hypotension.

Body weight will be measured as described in Table 6-1. Body temperature will be measured only at Screening, Visit 7/EOT, and at follow-up.

Clinically notable vital signs are defined in Appendix 1.

6.5.3 Laboratory evaluations

Routine blood samples will be collected as described in Table 6-1 and Table 6-2 and analyzed by the central laboratory. Blood samples taken at the screening visit are to be in the fasting state. Blood samples taken at subsequent visits are recommended to be in the fasting state. Details regarding collection of samples, shipment of samples, reporting of results, laboratory reference ranges, and alerting abnormal values will be supplied to the site before site initiation in a study laboratory manual. The results of the analysis will be made available to each site by the central laboratory, at the earliest, 48 hours after receipt of the samples by the central laboratory.

Investigators will be asked to comment on those abnormalities on the respective laboratory result page, including a notation of the clinical significance of each abnormal finding in the patient's source documents. The laboratory sheets will be filed with the patient's source documents. Abnormal laboratory values should not be recorded on the AE eCRF; however,

any diagnoses (or signs or symptoms if a diagnosis is not possible) associated with the abnormal findings should be recorded on the AE eCRF.

Clinically notable laboratory findings are defined in Appendix 1.

6.5.3.1 Hematology

Hematology parameter blood samples will be collected as described in Table 6-1 and will include: red blood cell count, total and differential WBC count (basophils, eosinophils, lymphocytes, monocytes, and neutrophils), platelet count, hemoglobin, hematocrit, mean corpuscular volume, mean corpuscular hemoglobin, mean corpuscular hemoglobin concentration, and red blood cell morphology. In a subset of patients CD4+, CD8+, T_h17, T_{reg}, and other T-lymphocyte subsets will be determined.

For study blinding purposes (of fingolimod arms) only the absolute counts for eosinophils, basophils, and monocytes will be communicated to sites by the central laboratory. The absolute total WBC, neutrophil, and lymphocyte counts will be measured at each visit by the central laboratory and will be blinded from the sponsor and the Investigator and will only be communicated to the site in case of a notable abnormality as defined in Appendix 1 and as per guidance in Appendix 3.

6.5.3.2 Clinical chemistry and blood serology

Blood samples will be collected as described in Table 6-1 and will include the following parameters: sodium, potassium, chloride, bicarbonate, calcium, magnesium, phosphate, blood urea nitrogen, uric acid, random glucose, albumin, AP, creatinine, ALT, AST, GGT, amylase, total bilirubin, conjugated bilirubin, HbA1c, total cholesterol, triglycerides, high-density lipoprotein, and low-density lipoprotein. Abnormal laboratory parameters inconsistent with clinical presentation of MS or suspicious of underlying medical condition should be repeated for accuracy.

If an increase of amylase above the clinically notable value (≥300 U/L) is observed at any postrandomization visit, lipase should be tested to determine the origin of elevated amylase (pancreatic versus extra-pancreatic).

Serology testing will also be performed in all patients at Screening to determine the patient's immune status with respect to the following viruses:

- Herpes simplex virus-1 (HSV-1)
- Herpes simplex virus-2 (HSV-2)
- Rubeola **(**Measles**)**
- Human immunodeficiency antibodies

Patients without acceptable evidence of immunity against VZV (per Appendix 3) at Screening will be excluded from the study. Patients testing positive for HIV are excluded from the study.

Patients who are negative for HSV-1 immunoglobulin G (IgG), HSV-2 IgG or rubeola IgG antibodies should be informed of their status and be instructed to promptly report any exposure to these viruses e.g., to a person with cold sores, herpes genitalis, or measles, respectively. In case of exposure, early treatment with appropriate antiviral drugs and/or immunoglobulin should be considered in consultation with a local infectious disease expert.

A positive IgG antibody result does not indicate active infection per se, but only evidence of prior exposure to viral antigens through past infection or vaccination. Patients with prior infection may be at risk of viral reactivation (e.g., cold sores, genital ulcers or shingles) and should be instructed to inform the investigator of any signs or symptoms suggestive of these conditions, so that prompt treatment may be initiated.

If a patient experiences symptoms of a new ischemic or thrombotic AE, the following additional laboratory assessments should be performed: prothrombin time/partial thromboplastin time, homocysteine, activated protein C, lupus anticoagulant, antiphospholipid antibody, protein C, protein S, fibrinogen, and antithrombin III.

A serum pregnancy test will be conducted at Screening on all females, regardless of child-bearing potential.

An additional blood sample (3 mL) will be collected at Screening, and will be stored for possible future exploratory assessments (e.g., relevant safety analysis like retrospective viral serology).

6.5.3.3 Urinalysis

Urinalysis will be performed as indicated in Table 6-1 and Table 6-2. The following parameters will be analyzed: leukocytes, specific gravity, bilirubin, blood, glucose, ketones, pH, protein, and urobilinogen.

If a patient experiences edema or significant weight gain (Appendix 1), urinalysis by dipstick for proteinuria will be performed. If dipstick urinalysis detects proteinuria, urinalysis should be repeated. If proteinuria is confirmed upon repeat testing, the protein/creatinine ratio should be determined in a spot urine sample. In case of abnormal spot urine protein/creatinine ratio, the patient should be referred to a nephrologist for further evaluation and assessments, including possible 24-hour urine collection.

6.5.4 Electrocardiogram

Standard 12-lead ECGs will be collected as described in Table 6-1. Digital ECG devices will be provided to each clinical site by the central ECG reader for the duration of the study. The screening ECG report from the central reader must be available to confirm patient eligibility before randomization. A 12-lead ECG will be performed at Visit 2 before study drug administration for patients receiving fingolimod and patients receiving glatiramer acetate and 6 hours after the study drug administration, for patients receiving fingolimod only.

See Section 5.5.5 for guidelines for monitoring patients taking their first dose of fingolimod who had a prolonged QTc interval (>450 msec males, >470 msec females) before dosing or during a 6-hour observation period, or at additional risk for QT prolongation (e.g., hypokalemia, hypomagnesemia, congenital long-QT syndrome), or on concurrent therapy with QT-prolonging drugs with a known risk of torsades de pointes.

Detailed instructions describing the process for recording and transmission of the ECGs will be outlined in the study-specific manual and provided to the site before the start of the study. Paper ECGs will be printed, photocopied to preserve the ink if necessary, and kept at the site as source documentation.

Interpretation of the tracing must be made by a qualified physician. Each ECG tracing should be labeled with the study number, patient initials, patient number, date, and kept in the source documents at the study site. Only clinically significant abnormalities should be reported as medical history/current medical conditions or AEs on the eCRF. Clinically significant findings must be discussed with the Medical Monitor before enrolling the patient in the study.

6.5.5 Ophthalmologic examinations and optical coherence tomography

An ophthalmic examination will be performed to screen for macular edema as described in Table 6-1 and Table 6-2 for patients that discontinued study drug. An optical coherence tomography (OCT) and fluorescein angiography (FA) will be done only if needed to confirm macular edema. If there is a suspicion of macular edema at Screening, an OCT and FA should be performed to confirm the diagnosis. If the diagnosis is confirmed, the patient should be deemed a screening failure and should not be randomly assigned.

Patients with a history of or newly diagnosed uveitis after initiation of study drug may require more frequent ophthalmic evaluations. Refer to the Guidance for Ophthalmic Monitoring (Appendix 4) for details on monitoring of patients with uveitis during the study.

Study drug must be discontinued in any patient who has a diagnosis of macular edema confirmed by OCT and FA.

Patients with a diagnosis of macular edema must be followed up monthly and more frequently if needed based on the ophthalmologist’s judgment. Further ophthalmologic evaluations will be conducted until such time as resolution is confirmed or no further improvement is expected by the ophthalmologist (based on a follow-up period of not less than 3 months). If the patient does not show definite signs of improvement on examination by specialized testing (e.g., OCT) 6 to 8 weeks after discontinuation of study drug, then therapy for macular edema in conjunction with an ophthalmologist experienced in the management of this condition should be initiated.

6.5.6 Pregnancy and assessments of fertility

Serum pregnancy test will be performed for all women by the central laboratory as detailed in Table 6-1. Patients becoming pregnant will be recommended to interrupt the study drug. Additional pregnancy testing may be performed at the investigator’s discretion during the study.

6.5.7 Pharmacodynamic assessments (substudy only)

Pharmacodynamics assessments for the immune cell substudy will be initiated at Screening in all patients who have signed a separate informed consent to participate in the substudy. Subsequent pharmacodynamic assessments will only be performed in patients randomized to fingolimod and enrolled in the substudy.

Whole blood samples will be taken for all samples by direct venipuncture. One baseline sample will be taken at Screening on all substudy participants. A second baseline sample will be taken on Day 1 after randomization and prior to administration of the first dose of study drug. All other samples will be taken together with the regular safety laboratory samples.

Samples will be immediately shipped to Quintiles central facility for peripheral blood mononuclear cell (PBMC) processing.

Methods: Since Tregs and Th17 cells are predominantly within the CD4+ T cell contingent and are therefore strongly reduced in fingolimod-treated patients, classic staining techniques of whole blood may not be sensitive enough to achieve sufficient accuracy. Therefore, PBMC will be concentrated from whole blood prior to fluorescein activated cell sorter (FACS) analysis in order to reliably determine the ratio of Th17 versus Treg cells that are in circulation.

Other immune-cell subset analyses will include FACS measures of CCR7 expressing CD4+ and CD8+ T cells (which can then be related to total lymphocyte counts) by staining with antibody combinations to CD3/4/8/CCR7.

Detection of $CD4^{+}CD25^{+}FoxP3^{+}CD127^{dim}$ regulatory T cells: The test should be done as described (Miltenyi Biotec datasheet: human Treg Detection Kit). Briefly, cells are stained with fluorescently labeled antibodies to, amongst other antibodies markers, CD4, CD25, and CD127. Cells are fixed, permeabilized, and stained with labeled anti-FoxP3 antibodies. $CD4^{+}CD25^{+}FoxP3^{+}CD127^{dim}$ regulatory T cells are detected by flow cytometry.

Detection of TH17 cells: This will be undertaken by flow cytometry using cell surface marker only staining and/or cell surface and intracellular staining. For cell surface only staining, Th17 cells will be defined, amongst other antibody markers, as CD4+CD45RA-CD183-CD196+CD194+ using standard cell surface staining techniques. For intracellular staining, Th17 cells will be defined, amongst other antibody markers, as CD4+IL-17+.

Briefly, cells are stained with surface markers such as CD4-FITC, fixed, permeabilized, and stained with Anti-IL-17.

Frequencies of cytokine-defined T-cell subsets (e.g., IL 17) are expected to be quite low in freshly isolated PBMC. Treatment effects of interest may only be captured when assessing the ability to induce T-cell subset responses upon activation. Therefore, it may be necessary to stimulate T-cell populations according to standard practices (e.g., phorbol 12-myristate 13-acetate/ionomycin and GolgiStop) to capture cytokine expression that reflects in vivo differentiation of cells.

If technically feasible, additional definition of Th17 cell subsets may be undertaken by analyzing intracellular markers such as STAT3-P and RORgt by flow cytometry. Analysis of these markers will require brief cytokine exposure (e.g., IL-6) followed by phospho-STAT and/or transcription factor analysis to capture the expression of these markers.

Importantly, inter-assay variability may confound the ability to compare pre-treatment and on-treatment samples. To overcome this, PBMC will be cryopreserved at all time points. This will enable side-by-side comparison of pre-treatment and on-treatment samples using phenotyping and activation protocols described (e.g., Bar-Or et al, 2010; Darlington et al, 2013).

Serum – specific molecules involved in differentiation of Treg and Th17 cell differentiation (including IL-10, TGF-β, IL-1b, IL-6, IL-22) and extrathymic proliferation (IL-7, IL-2, IL-15) will be assessed by Luminex®.

6.5.8 Chest x-ray

This section is no longer applicable after the changes made at protocol amendment 2.

6.5.9 Pulmonary function tests

Pulmonary function tests evaluating FEV_1, FVC, and D_LCO will be performed at Screening, Month 6, Month 12, End of Study, and 3 months after study drug discontinuation as indicated in Table 6-1 and Table 6-2. This test will be conducted in all patients who are enrolled into the study and in the manner consistent with the standard pulmonary laboratory practice.

Any condition that might affect the outcome of PFTs including infection, respiratory symptoms, occupational exposures (including asbestos), and cigarette smoking, needs to be collected before every PFT testing and transcribed to the PFT eCRF page.

Patients who discontinue study drug due to respiratory AE(s) should be evaluated by a pulmonary specialist and further investigations (e.g., PFTs, chest x-ray or HRCT, biopsy) should be performed as needed.

Spirometry

The technician should demonstrate the appropriate technique to the patient and follow the standard procedure. The quality of the tests must be accounted for including the technicians' comments (especially when, despite proper coaching of the patient, full collaboration cannot be achieved). The FEV_1/FVC ratio will be calculated centrally by the trial statistician.

A minimum of 3 acceptable maneuvers will be performed at each visit. The acceptability criteria are a satisfactory start of test and a satisfactory end of test. In addition, the technician should observe that the patient understood the instructions and performed the maneuver with a maximum inspiration, a good start, a smooth continuous exhalation, and maximal effort. The largest FVC and the largest FEV_1 will be recorded, after examining the data from all of the acceptable curves, even if the 2 values do not come from the same curve. Please refer to the American Thoracic Society/European Respiratory Society guidelines for standardization of spirometry (Miller et al, 2005a; Miller et al, 2005b) and single breath determination of carbon monoxide uptake in the lung (MacIntyre et al, 2005).

If for any reason a patient is permanently discontinued from study medication the patient will have PFTs performed at his last visit or within 30 days of the study drug discontinuation. Refer to Appendix 3 for Guidance on monitoring patients with pulmonary function safety concern.

Forced expiratory volume in 1 second (FEV_1):

The FEV_1 describes the volume (in Liters) that is expelled within one second of forced expiration after a maximal inspiration and reflects the large airway resistance. A decrease in FEV_1 serves as a good parameter for detection of an obstructive ventilatory defect.

Forced vital capacity (FVC):

The FVC denotes the volume of gas (in Liters) which is exhaled during a forced expiration starting from a position of full inspiration and ending at complete expiration. This parameter

is normal or might be slightly decreased in obstructive disease but shows a mild to severe decrease in restrictive disease.

Diffusion capacity of carbon monoxide (D_LCO):

Gas exchange is assessed by the D_LCO evaluated by the single breath holding method. It is a measurement of carbon monoxide (CO) transfer from the lung to pulmonary capillary blood over a breath-holding period. Due to ability of CO to bind to hemoglobin, the diffusion capacity needs to be corrected for hemoglobin to reflect an altered lung gas transport rather than altered hemoglobin.

The average of at least 2 acceptable tests that meet the repeatability requirement of either being with 3 mL CO/min/mm Hg (or 1 mmol/min/kPa) of each other or being within 10% of the highest value should be reported.

A D_LCO test is acceptable if it fulfills all the following criteria:

- Use of proper quality-controlled equipment
- Inspired volume of greater than 85% of largest vital capacity in less than 4 seconds
- A stable calculated breath hold for 10 (±2) seconds. There should be no evidence of leaks or Valsalva or Mueller maneuvers
- Expiration in less than 4 seconds (and sample collection time less than 3 seconds), with appropriate clearance of dead space and proper sampling/analysis of alveolar gas

All values must be corrected for hemoglobin concentration in the study.

The D_LCO is usually expressed in European CI conventional units: mL CO/min/torr (or mL CO/min/mm Hg), whereas the US mainly uses SI units: mmol CO/min/kPa (Conversion factor: SI units × 2.986 = CI units).

6.5.10 Dermatological examination

A dermatologist will complete a dermatological examination as outlined in Table 6-1 and Table 6-2 to support monitoring of patients for the potential development of new skin cancers during the study. For patients who discontinue treatment early, a dermatological examination should be performed at end of study. Study drug should be discontinued in patients who develop a new skin cancer during the study.

6.5.11 Columbia-Suicide Severity Rating Scale

The C-SSRS, is a semi-structured interview designed to systematically assess and track suicidal AEs (behavior and ideation) throughout different settings including clinical studies.

The C-SSRS captures the occurrence, severity, and frequency of suicide-related thoughts and behaviors. The C-SSRS will be administered by an IVRS with the patient entering responses directly into the IVRS. Caregivers will not be allowed to answer the C-SSRS questions on behalf of the patient. Sites must review reports received from the system for any answers indicative of suicidal ideation and AEs. Adverse events ascertained through the administration of the C-SSRS will be documented. This scale was developed by researchers at Columbia University and will be administered in this study as specified in the assessment schedules

(Table 6-1 and Table 6-2). In case the score is 4 or above, the patient must be referred to the health care professional for further assessment and/or treatment.

6.5.12 Appropriateness of safety measurements

The safety assessments selected are standard for this indication and patient population. Ophthalmic evaluation has been included for detection of macular edema. Macular edema is an AE of special interest in patients treated with fingolimod because of the higher incidence seen in treated patients compared with active and placebo control groups in previous studies.

6.6 Other assessments

6.6.1 Health-related quality of life

Patients must complete the TSQM, PRIMUS, and MSIS-29 questionnaires before other clinical assessments at any given visit. Completed questionnaires will be reviewed and examined by the investigator before the clinical examination for responses that may indicate potential AEs or SAEs. The investigator should review not only the responses to the questions in the questionnaires but also for any unsolicited comments written by the patient.

If the occurrence of AEs or SAEs is confirmed, the physician must record the events. Investigators should not encourage the patients to change the responses reported in the questionnaires.

6.6.1.1 Treatment Satisfaction Questionnaire for Medication v1.4 (TSQM v1.4)

Treatment Satisfaction Questionnaire for Medication (TSQM) was developed and validated as a general measure for treatment satisfaction (Atkinson 2004). The TSQM version 1.4 contains 14 items assessing the following 4 domains: Global Satisfaction, Effectiveness, Side Effects, and Convenience. This PRO must be completed prior to any other study visits assessments at the baseline, 6 and 12 month visits.

6.6.1.2 Patient Reported Indices of MS-Activities scale (PRIMUS-Activities)

PRIMUS is an instrument developed specifically for MS from the perspective of patients. It contains three independent scales which assess symptoms, overall quality of life and the ability of patients to perform typical daily activities; these scales were designed to be used in combination or as a stand-alone measure (Doward 2009; Twiss 2010). In this study in selected countries, the PRIMUS-Activities scale will be used and is intended to complement existing clinical measures of disability by providing a patient-reported account of the benefits of treatment on the ability to independently perform daily activities. It consists of 15 items covering typical daily activities that have been identified by individuals with MS, and assesses their ability to do the activities during the last week. The PRIMUS-Activities scale is to be completed at baseline, 6 months, and 12 months.

6.6.1.3 Multiple Sclerosis Impact Scale (MSIS-29)

Health-related quality of life will be assessed with the Multiple Sclerosis Impact Scale (MSIS-29). It is a 29-item, self-administered questionnaire that includes two domains, physical and psychological. It is a clinically useful and scientifically sound measure of the

impact of MS from the patient's perspective suitable for clinical trials and epidemiological studies (Hobart et al, 2001). It is considered a reliable, valid and responsive PRO measure that complements other indicators of disease severity used to improve our understanding of the impact of MS. The questions in the scale ask the patient for their views about the impact of MS on their day-to-day life during the past two weeks. The MSIS-29 takes approximately 5 minutes to complete, and has been translated into many languages. The MSIS-29 is to be completed at baseline, 6 months, and 12 months.

6.6.2 Resource utilization

Not applicable.

6.6.3 Pharmacogenetics/pharmacogenomics

Not applicable.

6.6.4 Pharmacokinetics

Blood samples will be collected from all patients for PK analysis to obtain data on fingolimod 0.25 mg and fingolimod 0.5 mg as outlined in Table 6-1. No PK sample will be collected from patients in the glatiramer acetate group.

Patients should be instructed NOT to take the dose of study medication at home on the study visit days. The patients should be asked to bring their bottle with them to the clinic and should take their study drug after the blood sample has been drawn by either direct venipuncture or an insertion of an indwelling cannula into a forearm vein.

Collection of blood

For each scheduled PK sample, 1.8 mL of venous blood will be collected into a vacuum tube containing sodium citrate. Immediately after the blood is drawn, the tubes should be inverted gently several times to ensure mixing of contents (e.g., anticoagulant). Avoid prolonged sample contact with the rubber stopper. Within 30 minutes of blood collection the tubes must be frozen at ≤-18°C pending analysis. Alternatively, the tubes can be transferred directly to a ≤-70°C immediately after sample collection. The PK samples should be batch shipped to the central laboratory monthly on dry ice. Sample should not remain on-site longer than 3 months from the day of collection.

All samples will be given a unique sample number and a collection number. The actual sample collection date and time will be entered on the PK blood collection page of the eCRF. Sampling problems will be commented on in the eCRF.

Analytical Method

Fingolimod and fingolimod-P whole blood concentrations will be determined using a liquid chromatography-mass spectrometry method with a lower limit of quantification of 0.08 ng/mL and 0.1 ng/mL (or smaller), respectively.

7 Safety monitoring

7.1 Adverse events

An AE is the appearance or worsening of any undesirable sign, symptom, or medical condition occurring after starting the study drug even if the event is not considered to be related to study drug. Study drug includes the investigational drug under evaluation and the comparator drug that is given during any phase of the study. Medical conditions and diseases present before starting study drug are only considered AEs if they worsen after starting study drug. Abnormal laboratory values or test results constitute AEs only if they induce clinical signs or symptoms, are considered clinically significant, or require therapy.

The occurrence of AEs should be sought by nondirective questioning of the patient at each visit during the study. Adverse events also may be detected when they are volunteered by the patient during or between visits or through physical examination, laboratory test, or other assessments. All AEs must be recorded in the AEs eCRF with the following information:

1. the severity grade (mild, moderate, or severe)
2. its relationship to the study drug(s) (suspected/not suspected)
3. its duration (start and end dates or if continuing at final examination)
4. whether it constitutes a serious AE (SAE)

An SAE is defined as an event which:

- is fatal or life threatening
- results in persistent or significant disability/incapacity
- constitutes a congenital anomaly/birth defect
- requires inpatient hospitalization or prolongation of existing hospitalization, unless hospitalization is for:
  - routine treatment or monitoring of the studied indication, not associated with any deterioration in condition
  - elective or preplanned treatment for a preexisting condition that is unrelated to the indication under study and has not worsened since the start of study drug
  - treatment on an emergency outpatient basis for an event not fulfilling any of the definitions of a SAE given above and not resulting in hospital admission
  - social reasons and respite care in the absence of any deterioration in the patient’s general condition
- is medically significant, i.e., defined as an event that jeopardizes the patient or may require medical or surgical intervention to prevent one of the outcomes previously listed

Unlike routine safety assessments, SAEs are monitored continuously and have special reporting requirements (Section 7.2).

All AEs should be treated appropriately. Treatment may include one or more of the following: no action taken (i.e., further observation only); study drug dose adjusted/temporarily

interrupted; study drug permanently discontinued due to this AE; concomitant medication given; nondrug therapy given; or patient hospitalized/patient's hospitalization prolonged. The action taken to treat the AE should be recorded on the AE CRF.

For patients who experience an ischemic or thrombotic AE, specialist advice should be sought to determine diagnostic and therapeutic procedures. If a laboratory screen for coagulation abnormalities is deemed appropriate, it should include the following additional assessments: prothrombin time/partial thromboplastin time, homocysteine, activated protein C, lupus anticoagulant, antiphospholipid antibody, protein C, protein S, fibrinogen, and antithrombin III.

Patients who discontinue due to respiratory AEs should be evaluated by a pulmonary specialist and additional investigations (e.g., PFTs, chest x-ray or HRCT, biopsy) should be performed as needed.

Once an AE is detected, it should be followed until its resolution or until it is judged to be permanent, and assessment should be made at each visit (or more frequently, if necessary) of any changes in severity, the suspected relationship to the study drug, the interventions required to treat it, and the outcome. Sites should send all unscheduled laboratory samples to the central laboratory to ensure that the laboratory data are included in the clinical database and reported.

Information about common side effects already known about the investigational drug can be found in the investigator brochure or will be communicated between investigator brochure updates in the form of investigator notifications. This information will be included in the patient informed consent and should be discussed with the patient during the study as needed.

7.2 Serious adverse event reporting

To ensure patient safety, every SAE, regardless of suspected causality, occurring after the patient has provided informed consent and until 30 days after the patient has stopped study participation (defined as time of last dose of study drug taken or last visit whichever is later), must be reported to Novartis within 24 hours of learning of its occurrence.

Any SAEs experienced after this 30-day period should only be reported to Novartis if the investigator suspects a causal relationship to the study drug.

Recurrent episodes, complications, or progression of the initial SAE must be reported as follow-up to the original episode, regardless of when the event occurs. This report must be submitted within 24 hours of the investigator receiving the follow-up information. An SAE that is considered completely unrelated to a previously reported one should be reported separately as a new event.

Information about all SAEs is collected and recorded on the Serious Adverse Event Report Form. The investigator must assess the relationship of any SAE to study drug, complete the Serious Adverse Event Report Form in English, and send the completed, signed form by fax within 24 hours to the local Novartis Drug Safety and Epidemiology Department. The telephone and telecopy number of the contact persons in the local Clinical Safety and Epidemiology Department, specific to the site, are listed in the investigator folder provided to each site. The original copy of the SAE Report Form and the fax confirmation sheet must be kept with the CRF documentation at the study site.

Follow-up information is sent to the same person to whom the original Serious Adverse Event Report Form was sent, using a new Serious Adverse Event Report Form stating that this is a follow-up to the previously reported SAE and giving the date of the original report. The follow-up information should describe whether the event has resolved or continues, if and how it was treated, whether the blind was broken or not, and whether the patient continued or withdrew from study participation.

If the SAE is not previously documented in the investigator’s brochure or package insert (new occurrence) and is thought to be related to the Novartis study drug, a Drug Safety and Epidemiology Department associate may urgently require further information from the investigator for health authority reporting. Novartis may need to issue an investigator notification to inform all investigators involved in any study with the same drug that this SAE has been reported. Suspected unexpected serious adverse reactions will be collected and reported to the competent authorities and relevant ethics committees in accordance with Directive 2001/20/EC or as per national regulatory requirements in participating countries.

7.2.1 Serious adverse event notification: multiple sclerosis relapse

Any MS relapses, as a general rule, should be reported on the relapse CRF instead of the AE/SAE forms. However, if, in the judgment of the investigator, any MS relapse is unusually severe and warrants specific notification, then an SAE Report Form must be completed and submitted according to SAE reporting procedures outlined above.

7.3 Pregnancies

To ensure patient safety, each pregnancy in a patient on study drug must be reported to Novartis within 24 hours of learning of its occurrence. The pregnancy should be followed up to determine outcome, including spontaneous or voluntary termination, details of the birth, and the presence or absence of any birth defects, congenital abnormalities, or maternal and/or newborn complications.

Pregnancy should be recorded on a Clinical Trial Pregnancy Form and reported by the investigator to the local Novartis Drug Safety and Epidemiology Department. Pregnancy follow-up should be recorded on the same form and should include an assessment of the possible relationship to the Novartis study drug of any pregnancy outcome. Any SAE experienced during pregnancy must be reported on the SAE Report Form.

7.4 Data safety monitoring board

An FTY720 data safety monitoring board (DSMB) has been established with a primary goal to monitor the safety of patients participating in all FTY720 studies, including the present study. The DSMB is an external board comprising specialists with specific knowledge of MS and other areas related to the safety of FTY720. Specific AEs of interest from this study will be submitted for review and evaluation by the DSMB.

8 Data review and database management

8.1 Site monitoring

Before study initiation, at a site initiation visit or at an investigator's meeting, a Novartis representative (i.e., CRO monitor or clinical team manager) will review the protocol and (e)CRFs with the investigators and their staff. During the study, the field monitor will visit the site regularly to check the completeness of patient records, the accuracy of entries on the (e)CRFs, the adherence to the protocol and to Good Clinical Practice, the progress of enrollment, and to ensure that study drug is being stored, dispensed, and accounted for according to specifications. Key study personnel must be available to assist the field monitor during these visits.

The investigator must maintain source documents for each patient in the study, consisting of case and visit notes (hospital or clinic medical records) containing demographic and medical information, laboratory data, ECGs, MRI films, and the results of any other tests or assessments. All information on (e)CRFs must be traceable to these source documents in the patient's file. The investigator must also keep the original informed consent form signed by the patient (a signed copy is given to the patient).

The investigator must give the field monitor access to all relevant source documents to confirm their consistency with the (e)CRF entries. Novartis monitoring standards require full verification for the presence of informed consent, adherence to the inclusion/exclusion criteria, documentation of SAEs, and the recording of data that will be used for all primary and safety variables. Additional checks of the consistency of the source data with the (e)CRFs are performed according to the study-specific monitoring plan. No information in source documents about the identity of the patients will be disclosed.

8.2 Data collection

Designated investigator staff will enter the data required by the protocol into the eCRF using fully validated software that conforms to 21 CFR Part 11 requirements. Designated investigator site staff will not be given access to the electronic data capture system until they have been trained. Automatic validation programs check for data discrepancies and, by generating appropriate error messages, allow the data to be confirmed or corrected before transfer of the data to the CRO working on behalf of Novartis. The investigator must certify that the data entered into the eCRF are complete and accurate. After database lock, the investigator will receive a CD-ROM of the patient data for archiving at the investigational site.

8.3 Database management and quality control

Novartis or designated CRO working on behalf of Novartis review the data entered into the eCRFs by investigational staff for completeness and accuracy and instructs the site personnel to make any required corrections or additions. Queries are sent to the investigational site using an electronic data query. Designated investigator site staff is required to respond to the query and confirm or correct the data.

Concomitant medication entered into the database will be coded using the WHO Drug Reference List, which employs the Anatomical Therapeutic Chemical classification system. Medical history/current medical conditions and AEs will be coded using the Medical Dictionary for Regulatory Activities (MedDRA) terminology.

Laboratory samples, ECG readings, MRI readings, and PK data will be processed centrally and the results will be sent electronically to a designated CRO.

Randomization codes and data about all study drug dispensed to the patient and all IVRS recorded dosage changes will be tracked using an IVRS. The system will be supplied by a vendor, who will also manage the database. The database will be sent electronically to Novartis (or a designated CRO).

Each occurrence of a code break via IVRS will be reported to the clinical team and monitor. The code break functionality will remain available until study shut down or upon request of Novartis.

9 Data analysis

9.1 Analysis sets

The following analysis sets will be used:

- Randomized set: consists of all patients who are assigned randomization numbers. The patients in this set are called randomized patients. This set will be used to summarize patient disposition, demographic and baseline characteristics, and protocol deviation information.
- Full-analysis set (FAS): Consists of all patients who are randomly assigned and take at least 1 dose of study drug. Following the intent-to-treat principle, patients will be grouped according to the assigned treatment at randomization. Efficacy analyses will be performed on the FAS unless otherwise notified.
- Per-protocol set: Consists of all patients in the FAS who do not have any major protocol deviations that could confound the interpretation of analyses conducted on the FAS. Major protocol deviations will be determined according to the predefined protocol deviation criteria before treatment unblinding. Any efficacy data after study drug discontinuation will be excluded. The per-protocol set will only be used for the supportive analyses of the primary efficacy variable.
- Safety set: Consists of all patients in the FAS who take at least 1 dose of study drug. Patients will be analyzed according to the treatment they have actually taken. Safety and tolerability analyses will be performed on the safety set unless otherwise notified.
- Follow-up set: The follow-up set consists of all patients in the safety set who have follow-up visit data or who have at least 1 safety assessment (AEs, laboratory test, vital sign measurement, or ophthalmology assessments) 46 days or more than 46 days after study drug discontinuation. Patients will be grouped in the same way as previously described for the analysis on the safety set. Only the safety follow-up data analysis will be performed on the follow-up set.

9.2 Patient demographics and other baseline characteristics

Demographics and background information will be summarized using frequency distributions for categorical variables and descriptive statistics of mean, SD, minimum, median, and maximum for continuous variables. Background information includes prior MS treatment, relevant medical history/current medical conditions, duration of the disease, the number of relapses experienced in the last 2 years before study enrollment, baseline MRI assessments, and baseline EDSS.

9.3 Treatments (study drug, rescue medication, other concomitant therapies, compliance)

Duration (days) of exposure to study drug will be summarized by treatment group. Frequency distributions will be used to summarize patient disposition and reasons for discontinuation of study drug. Patients who prematurely discontinue the study drug will be listed along with the reason for discontinuation.

The cumulative corticosteroid dose used after the start of study drug for the treatment of MS relapses will be summarized by treatment group in dose equivalent to methylprednisolone (the conversion factors will be detailed in the statistical analysis plan). The cumulative duration of the corticosteroid use will be summarized similarly.

9.4 Analysis of the primary variable

The primary analysis will be based on the FAS.

9.4.1 Variable

The primary outcome is the ARR which is defined as the average number of confirmed relapses per year (i.e., the total number of confirmed relapses divided by the total days in the study multiplied by 365.25). For the primary analysis, the number of relapses will include all the confirmed relapses experienced during the study. The time spent in the study will correspond to the observation period for all the relapses from first dose on study drug to end of study.

9.4.2 Statistical model, hypothesis, and method of analysis

The two doses of fingolimod will be tested hierarchically versus glatiramer acetate in a step-down procedure. For each of the two fingolimod doses, the null hypothesis is that there is no difference in the ARRs between patients treated with fingolimod and those treated with glatiramer acetate versus the alternative hypothesis that there is a difference between the two treatment groups. In order to preserve the Type I experiment-wise error rate, the null hypothesis will be rejected if the observed p value for the between-treatment comparison is less than the significance level as specified in the multiplicity adjustment procedure described later in this section.

H_{01}: $\mu_{\text{FTY 0.5 mg}} = \mu_{\text{glatiramer acetate}}$ versus H_{A1}: $\mu_{\text{FTY 0.5 mg}} \neq \mu_{\text{glatiramer acetate}}$

H_{02}: $\mu_{\text{FTY 0.25 mg}} = \mu_{\text{glatiramer acetate}}$ versus H_{A2}: $\mu_{\text{FTY 0.25 mg}} \neq \mu_{\text{glatiramer acetate}}$

No formal hypothesis will be tested between the 2 fingolimod doses because the study is not powered to detect a difference in treatment effect between these doses.

The hypotheses will be tested using a negative binomial regression model with log link, using treatment, number of relapses in the previous year before study enrollment, baseline EDSS, and baseline number of Gd-enhancing T1 lesions and country (or region) as covariates. In the analysis, the response variable is the number of confirmed relapses for each patient. The patient's time in the study (natural log of time in years) is used as an offset variable to obtain the ARR, adjusted for the varying lengths of patient's time in the study (time in years). Study centers will be pooled to country or region (based on geographical proximity and/or known or expected regional differences in medical care) in order to minimize the impact of low-enrolling centers/countries on the analysis (such as nonconvergence of the analysis model). Details of pooling will be provided in the statistical analysis plan before database lock. The SAS procedure GENMOD (or other software with similar functionality) will be used to conduct the analysis.

The treatment effect of each dose of fingolimod versus glatiramer acetate will be presented as an ARR ratio with corresponding 95% confidence intervals and p values. The relative reduction in ARR will be presented as "percentage change," calculated as ($\hat{\mu}_{FTY} / \hat{\mu}_{GA}$ -1).

Multiplicity adjustment for statistical hypothesis testing

The primary hypotheses tests will be tested using a hierarchical step-down procedure to control the experiment-wise error rate.

This study is designed to compare each of 2 doses of fingolimod to glatiramer acetate based on ARR. There are 2 hypotheses being tested (H01, H02).

Because it is highly likely that the approved dose of fingolimod (0.5 mg) is more efficacious than the lower dose (0.25 mg), the approved dose of fingolimod is initially tested against glatiramer acetate at a 2-sided significance level of 0.05. Only if this initial test H01 is rejected will H02, the low dose of fingolimod, be tested against glatiramer acetate also at a 2-sided significance level of 0.05.

Different scenarios of the anticipated treatment effect between fingolimod 0.5mg or 0.25mg versus glatiramer acetate have been evaluated based on the available data on fingolimod and the cumulative literature on glatiramer acetate. A 30% treatment benefit of fingolimod 0.5mg over glatiramer acetate could be anticipated on the basis of double-blind, 24-months, placebo-controlled studies of glatiramer acetate (Johnson et al, 2001) and fingolimod [Study CFTY720D2301] alone. However, more recent studies on glatiramer acetate, in a less active population, in a total of over 3000 patients, suggest that the efficacy of glatiramer acetate on ARR and other endpoints is similar to that of IFN beta-1a SC (REGARDS) or IFN beta-1b SC (BEYOND). The observed relapse rates on glatiramer acetate were 0.29 in REGARDS and 0.34 in BEYOND, compared to 0.3 (INF beta-1a SC) and 0.33 (INF beta-1b SC), respectively. In Study FTY720D2302, a double-blind 1-year study, fingolimod 0.5 mg was directly compared to IFN beta-1a IM. The ARR (0.21) in patients treated with fingolimod 0.5 mg was reduced by 52% compared to the ARR (0.43) in patients treated with INF beta-1a IM. Overall, the reduction in ARR in patients treated with fingolimod 0.5 mg compared to those treated with glatiramer acetate can be expected in the range of 30% to 50%. For the

purpose of this study, a 35% lower ARR in patients treated with fingolimod 0.5 mg (ARR=0.195) compared to those treated with glatiramer acetate (ARR=0.30) is assumed.

Fingolimod 0.25 mg has never been tested in a clinical trial. Based on PK/PD modeling a 14% higher ARR in patients on fingolimod 0.25 mg compared to those on fingolimod 0.5 mg is anticipated. However, the uncertainty of this estimate is substantial. A 95% confidence interval from the PK/PD model for the ARR in patients on fingolimod 0.25 mg ranges from ARR=0.183 to ARR=0.303. For the purpose of this study, an ARR of 0.225 is assumed in patients treated with fingolimod 0.25 mg, which corresponds to an increase in ARR of approximately 15% compared to fingolimod 0.5 mg and a relative treatment effect of 25% compared to glatiramer acetate.

9.4.3 Handling of missing values, censoring, and discontinuations

All patients in the FAS will be included in the primary analysis, i.e., the number of confirmed relapses observed up to the end of patient's participation in the study. Patients who discontinue from study treatment will remain in the study and follow the assessment schedule. Relapses will be counted regardless of whether a patient is on or off study drug. Therefore, it is expected that all randomized patients can contribute to the primary analysis. The primary analysis model adjusts for missing information (early study discontinuations) under some statistical assumptions (noninformative dropouts and constant ARR).

Additionally the following supportive analyses will be conducted:

The primary analysis will be repeated using the per-protocol set to provide an analysis of on-treatment data from patients who have no major protocol violations. Relapses that occurred after permanent discontinuation of study drug will be excluded and log (time on study drug in years) rather than log (time on study in years) will be used as the offset variable in the negative binomial model with log link.

To assess the impact of missing data on the primary analysis, a sensitivity analysis will be performed on ARRs in the full-analysis set using a nonparametric method with imputations. The ARRs will be analyzed by means of a rank analysis of covariance (ANCOVA) with treatment, and number of relapses in the previous year before study enrollment, baseline EDSS, and baseline number of Gd-enhancing T1 lesions as covariates (Stokes 2000). An adjustment for country or region can be considered prior to database lock and will be defined in the analysis plan. A month-by-month imputation scheme will be used whereby missing data in any given month of the study will be imputed with the overall mean number of relapses (calculated from all patients across all treatment groups) in the corresponding month.

A supplementary analysis using the same negative binomial model as in the primary analysis to be performed on all relapses (i.e., confirmed and unconfirmed relapses) in the full-analysis set will be performed. Additional analyses to further assess the robustness of the results maybe prespecified in the statistical analysis plan.

9.5 Analysis of secondary variables

9.5.1 Efficacy variables

All efficacy analyses of secondary variables specified in this section will be conducted on the full-analysis set unless otherwise specified.

Magnetic resonance imaging (MRI)

The analyses on MRI parameters will be performed on the full analysis set in the subset of patients who have MRI scans done during the study.

The MRI efficacy variables are listed as following:

- Percent change in brain volume from baseline at Month 12 or end of study
- Number of new and newly enlarging T2 lesions (compared with baseline MRI scan) at Month 12 or at end of study
- Proportion of patients free of new/newly enlarging T2 lesions compared to baseline at Month 12 or end of study
- Change (and % change) from Baseline in total volume of T2 lesions at Month 12 or end of study
- Number of new T1 hypointense (acute or chronic) lesions compared to baseline, at Month 12 or end of study
- Change and % change from baseline in total volume of T1 hypointense lesions (acute or chronic) at Month 12 or end of study
- Number of Gd-enhancing T1 lesions at Month 12 or end of study
- Total volume of Gd-enhancing T1 lesions at Month 12 or end of study

The number of new or newly enlarging T2 lesions at Month 12 or end of study and number of Gd-enhancing T1 lesions at Month 12 or the end of study will be analyzed using a negative binomial regression model with log link, using treatment, age, baseline T2 lesion number, baseline number of Gd-enhancing T1 lesions, and the number of relapses experienced in the previous year before study enrollment as covariates. An adjustment for country or region can be considered prior to database lock and will be defined in the analysis plan. For the analysis of T2 lesions, an offset variable will be used in the negative binomial analysis to adjust for the time in years (since start of study drug).

The proportion type variables will be analyzed using a logistic regression model with treatment, age, corresponding baseline value, baseline number of Gd-enhancing T1 lesions, and the number of relapses experienced in the previous year before study enrollment as covariates. An adjustment for country or region can be considered prior to database lock and will be defined in the analysis plan.

The continuous variables (change, percent change, and total volume) will be analyzed using a rank ANCOVA model adjusted for treatment, age, corresponding baseline value, and the number of relapses experienced in the previous year before study enrollment as covariates. An adjustment for country or region can be considered prior to database lock and will be defined in the analysis plan. A parametric analysis, especially for the change in brain volume, may be specified in the statistical analysis plan prior to database lock.

Relapses

All relapse-related analyses use confirmed relapses only, unless otherwise specified. Additional relapse-related variables including the following:

- Time to first relapse
- Proportion of relapse-free patients
- Severity of the relapses and recovery status

Time to first relapse will be analyzed using a Cox proportional hazards model with treatment, number of relapses in the previous year before study enrollment, baseline EDSS, baseline number of Gd-enhancing T1 lesions, and baseline T2 lesion volume as covariates. An adjustment for country or region can be considered prior to database lock and will be defined in the analysis plan. The estimated hazard ratio with 95% confidence interval between treatment groups will be obtained. In addition, Kaplan-Meier curves by treatment will be used to present the time-dependent cumulative frequency and percentage of patients reaching the time to first relapse; by-treatment Kaplan-Meier estimates (with 95% confidence interval) at the end of the study will be obtained. Two-sided 95% confidence intervals of the difference in Kaplan-Meier estimates will also be presented. The log-rank test of the treatment difference in the Kaplan-Meier estimates of the events function will be performed.

Patients without a confirmed relapse will be referred to as relapse-free patients. Proportion of relapse-free patients at the end of the study will be analyzed using a logistic regression model with treatment, number of relapses in previous year before study enrollment, baseline EDSS, and baseline number of Gd-enhancing T1 lesions as covariates. An adjustment for country or region can be considered prior to database lock and will be defined in the analysis plan. When calculating proportions, patients who discontinue the study prematurely before having a confirmed relapse will be considered as having a confirmed relapse. Sensitivity analyses, however, will be performed by 1) excluding these patients from the analysis and 2) considering these patients (who are treated as having a confirmed relapse in the main analysis) as having no confirmed relapse.

Proportions of patients with mild, moderate or severe relapse will be summarized by treatment at patient level and relapse level. For summaries at patient level, a patient is counted only in the most severe category for each variable. Similarly, the proportion of patients who need hospitalization due to a relapse, the proportion of patients with incomplete recovery from a relapse, and the proportion of patients who were treated with corticosteroids due to an MS relapse, will be summarized by treatment.

Multiple Sclerosis Functional Composite Measure

There are 3 components to the MSFC: leg function, arm function, and cognitive function. The corresponding tests are 25-foot timed walk, 9-hole peg test, Paced Auditory Serial Addition Test 3 (PASAT3). Multiple trials of each test will be performed at each visit.

The MSFC z-score and the 3 subscale scores (25-foot timed walk, 9-hole peg test, PASAT3) and their change from baseline values will be summarized by visit. For the change from baseline values at each visit, rank ANCOVA adjusted for treatment, corresponding baseline values, and age will be performed for treatment comparisons. An adjustment for region can be considered prior to database lock and will be defined in the analysis plan.

Symbol Digit Modality Test

The SDMT score and its change from baseline value will be summarized by visit. For the change from baseline values at each visit, rank ANCOVA adjusted for treatment, corresponding baseline values, and age will be performed for treatment comparisons. An adjustment for region can be considered prior to database lock and will be defined in the analysis plan.

9.5.2 Safety variables

All safety analyses will be conducted on the safety set.

Safety assessments will include: AEs, infections, bradycardia events, laboratory test results, vital sign measurements, dermatological examinations, PFTs, ophthalmic examinations, ECG data, and C-SSRS.

Adverse events will be summarized by presenting, for each treatment group, the number and percentage of patients having any AE by primary system organ class and preferred term. Infections will be considered AEs and summarized with the AEs. Severe AEs, SAEs, drug-related AEs, and the AEs leading to premature discontinuation of study drug will be presented in a similar format as AEs. Infections will also be summarized separately. Notable events will include death, nonfatal SAEs (including infections), and AEs (including infections) leading to study drug discontinuation. The frequencies and percentages of patients will be tabulated by treatment group.

Laboratory data will be summarized by presenting summary statistics of raw data and change from baseline values, by presenting shift tables using clinically notable ranges (Baseline to most extreme post-baseline value), and by flagging notable values in data listings. For liver function tests, the frequencies and percentages of patients with elevations of 1, 2, 3, 5, and 10 times the upper limit of normal will be summarized by visit and treatment group.

Estimated creatinine clearance will be summarized by presenting summary statistics by visit and treatment group. Change from baseline analyses will be presented by visit and treatment group.

Vital sign data will be summarized by presenting summary statistics for change from baseline values (both for the period 6 hours after the first dose and for further assessments by visit and by end of treatment). The incidence rates of notable vital sign abnormalities will be summarized. Further, the frequency distribution for pulse by visit and the frequency distribution for percent decline in pulse during first 6 hours will be presented.

Body weight and temperature data will be summarized by presenting summary statistics for change from baseline values by visit and at end of treatment. The incidence rates of notable weight and temperature abnormalities will be summarized.

Dermatological examination tables and patient listing will be summarized by visit and treatment group.

Pulmonary function test data will consist of the following parameters: FEV_1, FVC, FEV_1/FVC, and D_LCO. Summary tables and listings will be produced for both absolute values and percentage of predicted value when applicable. The data will be summarized by

presenting summary statistics of change from baseline. A FEV_1, FVC, FEV_1/FVC, D_LCO measurement below 80% of predicted will be considered to be abnormal.

The ophthalmic data will be summarized using distribution tables, summary statistics, and change from baseline by visit and treatment group. The patient listing will also be provided. The incidence (number and percentage) of macular edema in patients with and without diabetes will be reported by treatment group. Further analyses can be specified depending on the number of patients with diabetes in the trial.

The ECG intervals will be summarized by presenting summary statistics for change from baseline values by visit. The (uncorrected) QT interval will be corrected according to the Fridericia's formula. The maximum increase in corrected QT interval from baseline will be summarized and the frequencies of patients who fulfill the abnormality criteria based on the corrected QT interval will be calculated.

Frequency distribution of treatment-emergent suicidal ideation and behaviors from C-SSRS will be summarized.

Follow-up visit data will be summarized to assess patient's safety after discontinuation of the study drug.

9.5.3 Resource utilization

Not applicable.

9.5.4 Health related quality of life

Analysis of Treatment Satisfaction Questionnaire for Medication (TSQM v1.4)

The primary analysis for the TSQM will be between group change in Global Satisfaction from baseline to Month 12. Analyses of the Effectiveness, Side Effects, and Convenience domains will also be conducted.

For the TSQM, responses to items are summed and transformed so that higher scores indicate greater satisfaction. Specifically, TSQM scale scores are computed by adding the items loading on each domain. The lowest possible score is subtracted from the composite score and divided by the greatest possible score range. This provides a transformed score between 0 and 1 that is then multiplied by 100. If more than one item is missing from a subscale of the TSQM for a particular patient, this subscale should be considered invalid for that respondent.

Analysis of PRIMUS-Activities

The PRIMUS-Activities scale will be calculated and analyzed according to the scoring manual provided by Galen Research Limited, 2007.

The PRIMUS activity scale scores and their changes from Baseline will be summarized by visit. The changes from Baseline in PRIMUS activity scale scores at each post-randomization visit will be compared between treatment groups.

Analysis of MSIS-29

MSIS-29 scores and their changes from Baseline will be summarized by visit for both the physical and psychological domains. The changes from Baseline in both the physical and psychological domain scores at each post-randomization visit will be compared between treatment groups.

9.5.5 Pharmacokinetics

A PK plan will be prepared before final clinical data lock providing details for the proposed analysis.

9.5.6 Pharmacogenetics/pharmacogenomics

Not applicable.

9.5.7 Biomarkers

Not applicable.

9.5.8 Pharmacokinetic/pharmacodynamic

Population PK/PD modeling approaches will be used to relate the individual fingolimod PK parameters to key efficacy endpoints (e.g. relapse and number of new or enlarging T2 lesions). A modeling plan will be prepared before final clinical data lock providing details for the proposed analysis.

9.6 Sample size calculation

The study will randomize a total of 1960 patients, and is planned to provide approximately 90% power for the comparison of fingolimod 0.5 mg to glatiramer acetate at a 2-sided significance level of 0.05.

The sample size calculation is based on simulations from a negative binomial distribution with a constant dispersion parameter k.

The power of the study was evaluated under various ARR assumptions and various dropout patterns based on the cumulative literature on glatiramer acetate and the Novartis data on fingolimod. The basis of the assumptions for this study and their level of uncertainty are presented in Section 9.4.2 under the sub-header “Multiplicity adjustment for statistical hypothesis testing”. The anticipated overdispersion parameter (k=0.2231) was observed in the Month-12 analysis of Study CFTY720D2301. The anticipated ARR for patients treated with 0.5 mg fingolimod is $\mu_{FTY\ 0.5\ mg}$=0.195, the ARR for those treated with glatiramer acetate is $\mu_{glatiramer\ acetate}$=0.30. Therefore, the estimated ARR reduction for fingolimod 0.5 mg versus glatiramer acetate is 35%.

The total sample size of 1960 randomized is predetermined but the exact sample size of each arm will depend on when the randomization ratio switch occurs. For example, if the randomization ratio is switched when a total of 800 patients have been randomized from an original ratio of 1:1:1 for fingolimod 0.25 mg, fingolimod 0.5 mg, or glatiramer acetate, respectively to a ratio of 5:3:2, then 847 patients will be randomized to fingolimod 0.25 mg, 615 patients to fingolimod 0.5 mg and 498 patients glatiramer acetate, which will provide

more than 90% power to demonstrate superiority of fingolimod 0.5 mg dose versus glatiramer acetate in terms of ARR at a 2-sided significance level of 0.05 assuming a 15% drop-out rate. The calculations take into account that patients who discontinue prematurely from the study can participate with partial data to the primary endpoint.

Fingolimod 0.25 mg has never been studied in a clinical trial in MS. Based on PK/PD modeling results, it is anticipated that the ARR in patients treated with fingolimod 0.25mg is approximately 15% higher than in those treated with fingolimod 0.5mg. However, the uncertainty of this estimate is high; the 95% confidence interval of the estimated ARR in fingolimod 0.25 mg group ranges from 0.18 to 0.30. It is therefore anticipated that fingolimod 0.25mg is less efficacious than fingolimod 0.5mg, but more efficacious than glatiramer acetate. In line with the PK/PD modeling the anticipated ARR in patients treated with fingolimod 0.25 mg is $\mu_{FTY\ 0.25\ mg}$ =0.225, which corresponds to a reduction in ARR of 25% in patients treated with fingolimod 0.25 mg compared to those treated with glatiramer acetate.

Following the multiplicity adjustment procedure in Section 9.4.2, the power to detect a 25% reduction in ARR for patients treated with fingolimod 0.25 mg compared with patients treated with glatiramer acetate is approximately 68% at a 2-sided significance level of 0.05, if the primary objective for the fingolimod 0.5-mg dose can be rejected first. If fingolimod 0.25 mg is similarly efficacious as fingolimod 0.5 mg (i.e., ARR reduction versus glatiramer acetate is 35% rather than 25%), the power to detect this treatment effect at a 2-sided significance level of 0.05 is approximately 88%. If fingolimod 0.25 mg is similarly efficacious as glatiramer acetate (i.e., estimated ARR in the fingolimod 0.25-mg group is at the high end of range proposed by the PK/PD model), there will be no significant difference at a 2-sided significance level of 0.05.

The statistical software R (Version 2.13.1, open source) and the R library packages “MASS” and “PSCL” were used for sample size calculations and power analysis.

9.7 Power for the key secondary hypotheses

Not applicable.

9.8 Immune-cell substudy analyses

All patients in the substudy who have contributed at least the baseline and one post-baseline sample will be included in the analysis. The objective of the analysis is the description of the effect of treatment with fingolimod on the proportions of Th17 and Treg cells, as compared to baseline.

The ratio of Treg and Th17 cells to the total lymphocyte cell number will be summarized by treatment. Other analyses may be prespecified in the analysis plan.

9.9 Interim analysis

Not applicable. No interim analysis is planned for this study.

10 Ethical considerations

10.1 Regulatory and ethical compliance

This clinical study was designed and shall be implemented and reported in accordance with the ICH Harmonized tripartite guidelines for Good Clinical Practice, with applicable local regulations (including European Directive 2001/20/EC, US Code of Federal Regulations Title 21, and Japanese Ministry of Health, Labor, and Welfare), and with the ethical principles laid down in the Declaration of Helsinki.

10.2 Informed consent procedures

Eligible patients may only be included in the study after providing written (witnessed, where required by law or regulation), IRB/IEC-approved informed consent, or, if incapable of doing so, after such consent has been provided by a legally acceptable representative of the patient. In cases where the patient's representative gives consent, the patient should be informed about the study to the extent possible given his or her understanding. If the patient is capable of doing so, he or she should indicate assent by personally signing and dating the written informed consent document or a separate assent form. Informed consent must be obtained before conducting any study-specific procedures (i.e., all of the procedures described in the protocol). The process of obtaining informed consent should be documented in the patient's source documents.

PPD will provide to investigators in a separate document a proposed informed consent form that complies with the ICH Good Clinical Practice guideline and regulatory requirements and is considered appropriate for this study. Any changes to the proposed consent form suggested by the investigator must be agreed to by PPD before submission to the IRB/IEC, and a copy of the approved version must be provided to the PPD monitor after IRB/IEC approval.

10.3 Responsibilities of the investigator and IRB/IEC

The protocol and the proposed informed consent form must be reviewed and approved by a properly constituted IRB/IEC before study start. A signed and dated statement that the protocol and informed consent have been approved by the IRB/IEC must be given to Novartis/PPD before study initiation. Before study start, the investigator is required to sign a protocol signature page confirming his/her agreement to conduct the study in accordance with these documents and all of the instructions and procedures found in this protocol and to give access to all relevant data and records to Novartis/PPD monitors, auditors, Novartis/PPD Clinical Quality Assurance representatives, designated agents of Novartis/PPD, IRBs/IECs, and regulatory authorities as required. If an inspection of the clinical site is requested by a regulatory authority, the investigator must inform Novartis and PPD immediately that this request has been made.

10.4 Publication of study protocol and results

Novartis assures that the key design elements of this protocol will be posted in a publicly accessible database such as clinicaltrials.gov. In addition, upon study completion and finalization of the study report the results of this trial will be either submitted for publication and/or posted in a publicly accessible database of clinical trial results.

11 Protocol adherence

Investigators agree that they will apply due diligence to avoid protocol deviations. Under no circumstances should the investigator contact Novartis or its agents, if any, monitoring the trial to request approval of a protocol deviation as no authorized deviations are permitted. If the investigator feels that a protocol deviation would improve the conduct of the study, this must be considered a protocol amendment. Unless such an amendment is agreed upon by Novartis and approved by the IRB/IEC, it cannot be implemented. All significant protocol deviations will be recorded and reported in the clinical study report.

11.1 Protocol amendments

Any change or addition to the protocol can only be made in a written protocol amendment that must be approved by Novartis, Health Authorities where required, and the IRB/IEC. Only amendments that are required for patient safety may be implemented before IRB/IEC approval. Notwithstanding the need for approval of formal protocol amendments, the investigator is expected to take any immediate action required for the safety of any patient included in this study, even if this action represents a deviation from the protocol. In such cases, Novartis should be notified of this action and the IRB/IEC of the study site should be informed within 10 working days.

12 References (Available Upon Request)

Atkinson MJ, Sinha A, Hass SL, et al. (2004) Validation of a general measure of treatment satisfaction, the Treatment Satisfaction Questionnaire for Medication (TSQM), using a national panel study of chronic disease. *Health Qual Life Outcomes*; 2:12.

Bar-Or A, Fawaz L, Fan B, et al. (2010) Abnormal B-cell cytokine responses a trigger of T-cell-mediated disease in MS? *Ann Neurol* 67(4):452-61.

Bornstein MB, Miller A, Slagle S, et al. (1987) A Pilot Trial of Cop 1 in Exacerbating-Remitting Multiple Sclerosis *N Engl J Med* Aug 13;317(7):408-14.

Cadavid D, Wolansky LJ, Skurnick J, et al. (2009) Efficacy of treatment of MS with IFNbeta-1b or glatiramer acetate by monthly brain MRI in the BECOME study , *Neurology*., 72(23):1976-1983.

Cohen JA, Barkhof F, Comi G, et al. (2010) Oral fingolimod or intramuscular interferon for relapsing multiple sclerosis; *N. Engl. J. Med*. 362(5), 402-15. Fingolimod Phase III trial.

Darlington PJ, Touil T, Doucet J-S, et al. (2013) Diminished Th17 (not Th1) responses underlie multiple sclerosis disease abrogation after hematopoietic stem cell transplantation. *Ann Neurol* 73(3):341-354.

Doward LC, McKenna SP, Meads DM, et al. (2009) The development of patient reported outcome indices for multiple sclerosis (PRIMUS). *Multiple Sclerosis* 15: 1092-1102.

Hobart J, Lamping D, Fitzpatrick R, et al. (2001) The Multiple Sclerosis Impact Scale (MSIS-29) A new patient-based outcome measure. *Brain* 124: 962-973

Johnson KP, Brooks BR, Cohen JA, et al.; The copolymer 1 MS study Group. (2001) Copolymer-1 reduces relapse rate and improves disability in relapsing remitting multiple

sclerosis: results of a phase III multicenter, double-blind, placebo-controlled trial. 1995. *Neurology*: 57 (12 Suppl 5):S16-24.

Kappos L, Radue EW, O'Connor P, et al. (2010) A placebo-controlled trial of oral fingolimod in relapsing multiple sclerosis. *N. Engl. J. Med.* 362(5), 387–401. Fingolimod Phase III trial.

Kebir H, Kreymborg K, Ifergan I, et al. (2007) Human Th17 lymphocytes promote blood-brain barrier disruption and central nervous system inflammation *Nature Medicine* 13(10) 1173-5.

Keegan BM, Noseworthy JH. (2002) Multiple sclerosis. *Annu Rev Med.*;53:285-302.

Keene CD, Sonnen JA, Swanson PD, et al. (2007) Neural transplantation in Huntington disease: long-term grafts in two patients. *Neurology* 68(24):2093–8.

MacIntyre N, Crapo RO, Viegi G, et al. (2005). Standardisation of the single-breath determination of carbon monoxide uptake in the lung. *Eur Respir J* 26(4):720-35.

McDonald WI, Compston A, Edan G, et al. (2001) Recommended diagnostic criteria for multiple sclerosis: guidelines from the International Panel on the diagnosis of multiple sclerosis. *Ann Neurol* 50(1):121-7.

Mehling M, Lindberg R, Raulf F, et al. (2010) Th17 central memory T cells are reduced by FTY720 in patients with multiple sclerosis *Neurology* 75(5): 403-10.

Mikol DD, Barkhof F, Chang P, et al.; REGARD study group. (2008) Comparison of subcutaneous interferon beta-1a with glatiramer acetate in patients with relapsing multiple sclerosis (the REbif vs Glatiramer Acetate in Relapsing MS Disease [REGARD] study): a multicentre, randomised, parallel, open-label trial. *Lancet Neurol* 7(10), 903-14.

Miller MR, Crapo R, Hankinson J, et al. (2005a) General considerations for lung function testing. *Eur Respir J* 26(1):153-61.

Miller MR, Hankinson J, Brusasco V, et al. (2005b) Standardisation of spirometry. *Eur Respir J* 26(2):319-38.

O'Connor P, Filippi M, Arnason B, et al. (2009) 250 microg or 500 microg interferon beta-1b versus 20 mg glatiramer acetate in relapsing-remitting multiple sclerosis: a prospective, randomised, multicentre study; *Lancet Neurol* Oct:8(10):889-97.

Panitch H, Goodin DS, Francis G, et al.; EVIDENCE Study Group; Evidence of Interferon Dose-response: European North American Compartative Efficacy; University of British Columbia MS/MRI Research Group. (2002) Randomized, comparative study of interferon beta-1a treatment regimens in MS: The EVIDENCE Trial. *Neurology* 59(10):1496-506.

Polman CH, Reingold SC, Edan G, et al. (2005) Diagnostic criteria for multiple sclerosis: 2005 revisions to the ‘‘McDonald Criteria.’’ *Ann Neurol* 58(6):840–6.

Polman CH, Reingold SC, Banwell B, et al. (2011) Diagnostic criteria for multiple sclerosis: 2010 revisions to the McDonald criteria. *Ann Neurol* 69(2):292-302.

Stokes ME, Davis CS, Koch GG. (2000) *Categorical data analysis using the SAS system*. SAS Institute, Inc., Cary, N.C.

Twiss J, Doward LC, McKenna SP, Eckert B. (2010) Interpreting scores on multiple sclerosis-specific patient reported outcome measures (the PRIMUS and U-FIS). *Health and Quality of Life Outcomes* 8:117.

Tzartos JS, Friese MA, Craner MJ, et al. (2008) Interleukin-17 production in central nervous system-infiltrating T cells and glial cells is associated with active disease in multiple sclerosis. *Am J Pathol* 172(1):146-155.

13 Appendices

Appendix 1: Clinically notable laboratory values and vital signs

Only selected lab parameters identified as notable which have been shown to be sensitive to fingolimod exposure are included.

CRITERIA FOR NOTABLE LABORATORY ABNORMALITIES

**Notable Values**		
**Laboratory Variable**	**Standard Units**	**SI Units**
**LIVER FUNCTION AND RELATED VARIABLES**		
SGOT (AST)	>82 U/L	>82 U/L
SGPT (ALT)	>90 U/L	>90 U/L
Total bilirubin	≥2.0 mg/dL	≥34.2 μmol/L
Alkaline phosphatase	>280 U/L	>280 U/L
**RENAL FUNCTION / METABOLIC AND ELECTROLYTE VARIABLES**		
Glucose `	≥200 mg/dL	≥11.11 mmol/L
Creatinine	≥2.0 mg/dL	≥176 umol/L
Amylase	≥300 U/L	≥300 U/L
Cholesterol	≥240 mg/dL	≥6.21 mmol/L
Triglycerides	≥300 mg/dL	≥3.39 mmol/L
Blood urea nitrogen (BUN)	≤2 mg/dL<br>≥30 mg/dL	≤0.7 mmol/L<br>≥10.7 mmol/L
Sodium	<125 mEq/L<br>>154 mEq/L	<125 mmol/L<br>>154 mmol/L
Chloride	≤85 mEq/L<br>≥119mEq/L	≤85 mmol/L<br>≥119 mmol/L
Potassium	≤3.0 mEq/L<br>≥6.0 mEq/L	≤3.0 mmol/L<br>≥6.0 mmol/L
Magnesium	≤1.0 mg/dL<br>≥3.0 mg/dL	≤0.40 mmol/L<br>≥1.23 mmol/L
Calcium	≤7.5 mg/dL<br>≥11.6 mg/dL	≤1.87 mmol/L<br>≥2.89 mmol/L
Phosphate	≤2.0 mg/dL<br>≥5.3 mg/dL	≤0.65 mmol/L<br>≥1.71 mmol/L

**Notable Values**		
**Laboratory Variable**	**Standard Units**	**SI Units**
**HEMATOLOGY VARIABLES**		
Hemoglobin	≤10.0 g/dL (M/F)	≤100 g/L (M/F)
Platelets (Thrombocytes)	≤100 k/mm^3<br>≥600 k/mm^3	≤100 x 10^9/L<br>≥600 x 10^9/L
Leukocytes (WBCs)	≤2.0 k/mm^3<br>≥15 k/mm^3	≤2.0 x 10^9/L<br>≥15 x 10^9/L
**HEMATOLOGY VARIABLES: DIFFERENTIAL**		
Granulocytes (Poly, Neutrophils)	≤1,000 /mm^3<br>≥12000/mm^3	≤1 x 10^9/L<br>≥12 x 10^9/L
Lymphocytes	<200/mm^3<br>≥8000/mm^3	<0.2 x 10^9/L<br>≥8 x 10^9/L
Red blood cells	<3,300,000/mm^3<br>>6,800,000/mm^3	<3.3 x 10^{12}/L<br>>6.8 x 10^{12}/L

**NOTABLE VITAL SIGNS AND BODY WEIGHT**	
**Vital Sign Variable**	**Notable Criteria**
Pulse (beats/min)	>120 bpm or Increase of ≥15 bpm from baseline<br>Or<br><50 bpm or Decrease of ≥15 bpm from baseline
Systolic BP (mm Hg)	≥160 mm Hg or Increase of ≥20 mm Hg from baseline<br>Or<br>≤90 mm Hg or Decrease of ≥20 mm Hg from baseline
Diastolic BP (mm Hg)	≥100 mm Hg or Increase of ≥15 mm Hg from baseline<br>Or<br>≤50 mm Hg or Decrease of ≥15 mm Hg from baseline
Body Temperature (°C)	>38.3°C/101°F
Body Weight (kg)	± 7% from baseline weight

Appendix 2: 2010 Revisions to the McDonald diagnosis criteria for MS

Guidelines from International Panel on the diagnosis of MS

(McDonald et al, 2001; Polman et al, 2005; Polman et al, 2011)

Clinical Presentation	Additional Data Needed for MS Diagnosis
2 or more attacks[a]; objective clinical evidence of 2 or more lesions or objective clinical evidence of 1 lesion with reasonable historical evidence of a prior attack[b]	None[c]
Two or more attacks[a]; objective clinical evidence of 1 lesion	**Dissemination in space**, demonstrated by:<br>≥1 T2 lesion in at least two out of four MS-typical regions of the CNS (periventricular, juxtacortical, infratentorial, or spinal cord)[d]<br>OR<br>Await a further clinical attack[a] implicating a different CNS site
One attack[a]; objective clinical evidence of 2 or more lesions	**Dissemination in time**, demonstrated by:<br>Simultaneous presence of asymptomatic gadolinium-enhancing and non-enhancing lesions at any time.<br>OR<br>A new T2 and/or gadolinium-enhancing lesion(s) on follow-up MRI, irrespective of its timing with reference to a baseline scan.<br>OR<br>Await a further clinical attack[a]

One attack[a]; objective clinical evidence of 1 lesion (clinically isolated syndrome)	**Dissemination in space and time, demonstrated by:**<br>**For DIS**<br>≥1 T2 lesion in at least two out of four MS-typical regions of the CNS periventricular, juxtacortical, infratentorial, or spinal cord)[d]<br>OR<br>Await a second clinical attack[a] implicating a different CNS site<br>**AND**<br>**For DIT**<br>Simultaneous presence of asymptomatic gadolinium-enhancing and non-enhancing lesions at any time.<br>OR<br>A new T2 and/or gadolinium-enhancing lesion(s) on follow-up MRI, irrespective of its timing with reference to a baseline scan.<br>**OR**<br>Await a second clinical attack[a]

If the Criteria are fulfilled the diagnosis is MS.

[a.] An attack (relapse; exacerbation) is defined this as patient-reported or objectively observed events typical of an acute inflammatory demyelinating event in the CNS, current or historical, with duration of at least 24 hours, in the absence of fever or infection. It should be documented by contemporaneous neurological examination, but some historical events with symptoms and evolution characteristic for MS, but for which no objective neurological findings are documented, can provide reasonable evidence of a prior demyelinating event. Reports of paroxysmal symptoms (historical or current) should, however, consist of multiple episodes occurring over not less than 24 hours. Before a definite diagnosis of MS can be made, at least one attack must be corroborated by findings on neurological examination, visual evoked potential (VEP) response in patients reporting prior visual disturbance, or MRI consistent with demyelination in the area of the CNS implicated in the historical report of neurological symptoms.

[b]Clinical diagnosis based on objective clinical findings for two attacks is most secure. Reasonable historical evidence for one past attack, in the absence of documented objective neurological findings, can include historical events with symptoms and evolution characteristics for a prior inflammatory demyelinating event; at least one attack, however, must be supported by objective findings.

[c]No additional tests are required. However, it is desirable that any diagnosis of MS be made with access to imaging based on these Criteria. If imaging or other tests (for instance, CSF) are undertaken and are NEGATIVE, extreme caution needs to be taken before making a diagnosis of MS and alternative diagnoses must be considered. There must be no better explanation for the clinical presentation and objective evidence must be present to support a diagnosis of MS.

[d]Gadolinium-enhancing lesions are not required; symptomatic lesions are excluded from consideration in subjects with brainstem or spinal cord syndromes.

Appendix 3: Guidance on safety monitoring

Patients whose blood pressure reading is ≥140 (systolic) and/or ≥90 mm Hg (diastolic) should have their blood pressure retested after 15 minutes of rest. If the blood pressure is still elevated, they should be followed up in one month by an unscheduled visit if the scheduled visit is not due. Should systolic BP ≥140 and/or diastolic BP ≥90 mm Hg values be confirmed at the second visit, the patient should be referred to his primary care physician, an independent internist or to the specialty hypertension clinic for evaluation, diagnosis and treatment of hypertension.

Patients with BP values of >160/100 mm Hg (confirmed by repeat testing) at any visit during the study should be immediately referred as above for evaluation, diagnosis and treatment of hypertension.

Newly diagnosed hypertension as well as an aggravation of a preexisting condition must be reported as an AE and appropriate antihypertensive medication/dosage adjustment must be considered by the investigator.

Guidance on monitoring of patients with elevated liver function tests

In case of detection of elevated ALT/AST values >2 and <5 times the ULN range, additional blood chemistry panel including ALT, AST, AP, GGT, total and conjugated bilirubin, albumin may be performed at the investigator's discretion.

If ALT/AST values reach 5 times the ULN, confirmed upon repeat testing within two weeks of the initial result or sooner at the discretion of the investigator, the study drug must be permanently discontinued. Patients who develop symptoms suggestive of hepatic dysfunction such as unexplained vomiting or jaundice, should have liver enzymes checked and fingolimod should be discontinued if significant liver injury is confirmed.

In case of isolated elevation of bilirubin over 2.0 mg/dl (34.2 umol/L) unless in context of Gilbert's syndrome, confirmed upon repeat testing within two weeks of the initial result, the investigator must discontinue the study drug. Additional evaluations may be performed at the discretion of the investigator.

For any unscheduled laboratory assessments performed locally, an identical sample should also be sent to the central laboratory for analysis and capture in the central database.

An interruption or discontinuation of the study drug should be clearly documented and reflected on Dosage Administration Record CRF. AE/SAEs need to be filed as appropriate.

Guidance on monitoring of patients with notable lymphopenia

Fingolimod results in sequestration of a proportion of the circulating lymphocytes in lymph nodes with resultant reduction in circulating lymphocyte counts. Average circulating lymphocytes counts are expected to be around 0.5 -0.6 $x10^9$ /L or 500- 600 cells/mm^3. Please see Investigator Brochure for more details. As such, the absolute total WBC, neutrophil and lymphocyte counts will be measured at each visit by the central laboratory, but will be reported blinded for subjects randomized to fingolimod. Lymphocyte counts below $0.2x10^9$/L in patients randomized to fingolimod treatment are reported unblinded and require repeat

testing within 2 weeks. If the repeat test confirms lymphocyte counts below $0.2x10^9/L$, the investigator should perform at least monthly retests. Results will only be reported blinded again once they reach $0.6x10^9/L$.

If the repeat test confirms the lymphocyte count is below $0.2x10^9/L$ or 200 cells/mm^3 and there are no signs or symptoms of infection, the patient can continue on study medication at the discretion of the investigator with regular monitoring for signs of infections.

If the patient presents with signs of infection and the lymphocyte count is below $0.2x10^9/L$, the study drug must be temporarily interrupted, and the investigator should consider treatment with a specific therapy on the basis of the clinical diagnosis in consultation with an infectious disease specialist. Reinitiation of the study drug can only be considered once the lymphocyte count reaches 600 cells/mm^3 as confirmed by the central laboratory and after discussion with the Medical Monitor.

If the patient is randomized to the glatiramer acetate treatment group and presents with significant lymphopenia (<50% baseline) confirmed on repeat test by central laboratory within two weeks, study drug must be interrupted and further diagnostic work-up needs to be initiated. The investigator should only consider re-initiation of the glatiramer acetate after a thorough diagnostic workup has been performed, diagnosis/cause for the lymphopenia has been established, treatment has been initiated as adequate, and only after discussion with Medical Monitor and if the patient shows no signs or symptoms of infection or malignancy.

Guidance on monitoring of patients with symptoms of neurological deterioration, inconsistent with MS course

Should a patient develop any manifestations that, in opinion of the investigator, are atypical for multiple sclerosis including unexpected neurological or psychiatric symptom/signs (e.g. rapid cognitive decline, behavioral changes, cortical visual disturbances or any other neurological cortical symptoms/sign), or any symptom/sign suggestive of an increase of intracranial pressure or accelerated neurological deterioration, the investigator should schedule a complete physical and neurological examination and an MRI as soon as possible and before beginning any steroid treatment. Conventional MRI as defined in the protocol as well as Fluid-attenuated Inversion Recovery (FLAIR) and Diffusion-weighted imaging (DWI) sequences are recommended for differential diagnosis of Posterior reversible encephalopathy syndrome. The MRI must be evaluated by the local neuroradiologist. The investigator will contact the Medical Advisor at Novartis to discuss findings and diagnostic possibilities as soon as possible. AE/SAEs need to be filed as appropriate.

In case of new findings in the MRI images in comparison with the previous available MRI which are not compatible with MS lesions, the study drug will be discontinued and other diagnostic evaluations need to be performed at the discretion of the investigator. In case of presence of new hyperintense T2-weighted lesions in the MRI which may be infectious in origin it is recommended to collect a cerebrospinal fluid sample if indicated. Analysis of the CSF sample including cellular, biochemical and, microbiological analysis (e.g. herpes virus, JC virus), to confirm/exclude an infection (e.g. PML) should be performed. In the event of suspected CNS infection, a CSF aliquot should be sent to a central laboratory (designated by the sponsor) for confirmatory testing.

Only when the differential diagnosis evaluations have excluded other possible diagnosis than MS and after discussion with the Medical Advisor at Novartis, the study drug may be restarted.

Guidance on monitoring of patients with infections

All infections that develop during the study will be reported as AEs. Investigators are requested to specifically ask about infections at each visit. Treatment and additional evaluations will be performed at discretion of the investigator.

The investigator should be vigilant for risk of infections including opportunistic infections due to bacterial infections (e.g., atypical mycobacteria), viral infections (e.g., HSV, VZV, JCV), or fungal infections (e.g., cryptococcus agents) and should remind the patient of the risk of infections and to instruct them to promptly report any symptoms of infections to the investigator. The patients must also be reminded to always carry their Patient Information Card (with site contact information and which identifies them as participants in a clinical study with an agent with potential immunosuppressive effects) and to show this to any local healthcare provider they may consult and ask that the investigator be contacted.

When evaluating a patient with a suspected infection, the most sensitive tests available should be used (i.e. that directly detect the pathogen, as with PCR).

The investigator should consider early treatment with specific therapy on the basis of clinical diagnosis or suspicion thereof (e.g., antiviral treatment for herpes simplex or VZV; treatment for cryptococcus) in consultation with infectious disease experts, as appropriate. Investigators should be aware that in the post-marketing setting with fingolimod, isolated cases of cryptococcal meningitis have been reported. Patients reporting symptoms and signs (such as, but not limited to, headache accompanied with stiff neck, sensitivity to light, fever, confusion, tiredness, body aches, chills, vomiting, and/or nausea) consistent with meningitis should undergo prompt diagnostic evaluation. If (cryptococcal) meningitis is diagnosed, appropriate antibiotic treatment should be initiated as soon as possible. The investigator should inform the Novartis medical expert and the CRO’s medical expert of any such cases.

Suspension of treatment with fingolimod should be considered if a patient develops a serious infection, and consideration of benefit-risk should be undertaken prior to re-initiation of therapy.

Investigators should consider the added immunosuppressive effects of corticosteroid therapy for treatment of MS attack/relapse and increase vigilance regarding infections during such therapy and in the weeks following administration.
Patients should be evaluated for evidence of immunity to VZV based on any of the following:

- Laboratory evidence of immunity or laboratory confirmation of disease
- Diagnosis or verification of a history of VZV or herpes zoster by a health care provider

To verify a history of VZV, health care providers should inquire about:

- An epidemiologic link to another typical VZV case or to a laboratory confirmed case, or
- Evidence of laboratory confirmation, if testing was performed at the time of acute disease

The VZV vaccination of antibody-negative patients should be considered prior to commencing treatment with fingolimod, following which initiation of study drug should be postponed for at least 1 month to allow full effect of vaccination to occur.

Serology testing for antibody status of HSV-1 and HSV-2 and rubeola (measles) is performed at screening to profile infection risk in study patients. The investigator should inform the patients of their immune status based on these serology results and of the potential risks of primary infections or viral reactivation while taking fingolimod.

A positive IgG antibody test result does not indicate active infection per se, but only evidence of exposure to viral antigens via past infection or vaccination. These patients may however, be at risk for viral reactivation, which may manifest as:

- VZV virus IgG positive: Shingles
- HSV-1 IgG positive: Cold sores
- HSV-2 IgG positive: Herpes genitalis

The investigator should instruct the patient to be alert to and report any symptoms or signs suggestive of cold sores, genital ulcers or shingles, so that appropriate anti-viral treatment can be initiated in consultation with a local infectious disease expert (if needed).

A negative IgG antibody test result for HSV-1, HSV-2, and rubeola places patients at risk for more severe and atypical manifestations of primary infection in the event they are exposed to these viruses while they are immunosuppressed (taking study drug and/or corticosteroids).

Patients who are negative for HSV-1 IgG, HSV-2 IgG and rubeola antibodies should be instructed to promptly report any exposure to these viruses i.e. to a person with cold sores, herpes genitalis, respectively. In case of exposure, early treatment with appropriate antiviral drugs should be considered in consultation with a local infectious diseases expert.

It is also important to ask the patient receiving fingolimod to report if they are exposed to anyone who has recently received a live or live attenuated vaccine and manifested a skin rash after the vaccination so that it can be decided, in consultation with an infectious disease expert, if antiviral therapy is warranted.

It should be noted that live or live attenuated vaccines are prohibited while fingolimod patients are taking study drug and for 8 weeks after study drug discontinuation.

Guidance on monitoring pulmonary function

Patients reporting any respiratory symptoms such as dyspnea, shortness of breath, chest tightness, wheezing should be seen at an unscheduled visit for clinical assessment. A full pulmonary function test (FEV_1, FVC, and D_LCO_c [i.e., D_LCOcorrected]) and a bronchodilator reversibility test need to be performed, preferably on the same day. If PFTs demonstrate reduction of FEV_1 or FVC values to 80% or below of baseline values, initiation of treatment with bronchodilators will be considered.

In case of reduction of FEV_1, FVC, and/or D_LCO_c below 80% of baseline values, the patient's pulmonary status will require a follow-up as soon as possible within no more than 1 month, including history of respiratory symptoms (eg, dyspnea, shortness of breath, chest tightness, wheezing), physical examination, FEV_1, FVC, D_LCO_c measurement, and a bronchodilator reversibility test.

Should repeat PFT values (FEV_1, FVC, and/or D_LCO_c) remain below 80% of baseline values or should a worsening of the previously reported respiratory symptoms be observed, the patient will be referred to pneumologist (pulmonologist) for further evaluation, including chest HRCT and treatment. A standard referral letter explaining a reason for referral should be accompanied by the sponsor's information letter about the investigational drug (FTY720).

In case of reduction of FEV_1, FVC, and/or D_LCO_c below 60% of baseline value in any visit, the study drug should be discontinued and the patient must be immediately referred to pneumologist (pulmonologist) for further evaluation, including chest HRCT and treatment.

In case of persistent reduction of FEV_1, FVC, and/or D_LCO_c below 80% over 3-month period despite appropriate treatment, the Primary Treating Physician should consider an interruption of the study drug. This decision may be discussed with Medical Monitor.

Appendix 4: Guidance for ophthalmic monitoring

In MS studies, macular edema was reported in 0.4% of patients receiving fingolimod 0.5 mg, and the majority occurred in the first 3-4 months. Some patients presented with blurred vision or decreased visual acuity, but others were asymptomatic and diagnosed during routine ophthalmological examinations. Macular edema generally improved or resolved spontaneously after discontinuation; continuation of fingolimod in patients with macular edema has not been evaluated. Patients with diabetes mellitus or with uveitis are at increased risk of macular edema. Fingolimod has not been studied in multiple sclerosis patients with concomitant diabetes mellitus.

Ophthalmic examinations, including optical coherence tomography (OCT) and fluorescein angiography (FA), for assessment of macular edema are scheduled as outlined in Table 6-1. Similar assessments must be performed as part of an unscheduled ophthalmology visit for any patient who presents with new visual symptoms. If patients report visual disturbances at any time while on therapy, evaluation of the fundus, including the macula, should be carried out by an ophthalmologist.

Guidance on monitoring patients for possible macular edema

If macular edema is suspected during the study, an OCT must be performed as a confirmatory test. If macular edema diagnosis is confirmed on OCT, study drug must be permanently discontinued.

These patients must be followed-up with monthly ophthalmologic evaluations, including OCT, until such time as resolution is confirmed or no further improvement is expected by the ophthalmologist (based on a follow-up period of not less than three months). If the patient does not show definite signs of improvement on examination by specialist testing (e.g., OCT) after 6-8 weeks after discontinuation of study drug, then therapy for macular edema in conjunction with an ophthalmologist experienced in the management of this condition should be initiated and further managed by the ophthalmologist until either resolution of the macular edema or there is absence of any evidence of further improvement.

The discontinuation of the study drug should be clearly documented and reflected on Dosage Administration Record CRF. AE/SAEs need to be filed as appropriate. For patients discontinuing study drug due to a diagnosis of macular edema, copies of the colored OCT as well as source documents of ophthalmic examination should be kept at the site as source documents. These documents may need to be submitted for review by an independent panel if needed.

Guidance on monitoring patients with uveitis

Patients with a history of uveitis or findings compatible with active uveitis at screening can enter the study given that there is no evidence of macular edema in the screening ophthalmic examination.

In order to specifically assess the risk of macular edema in the MS population with co-existing uveitis, each patient with findings in any ophthalmic examination compatible with active uveitis (e.g., significant anterior chamber cell or flare, vitreous cell or flare, pars

planitis, vasculitis, chorioretinitis) under the discretion of the investigator should undergo more frequent ophthalmic examinations. It is the discretion of the investigator to determine the frequency of these ophthalmic examinations. Adjustments to the schedule can be made to align these evaluations with other planned study visits.

A suspicion of macular edema will require an OCT to be performed (refer to Guidance for monitoring of patients with macular edema above).

Appendix 5: Multiple sclerosis functional composite measure (MSFC)

MSFC-INSTRUCTIONS

(from the "Administration and Scoring Manual for the Multiple Sclerosis Functional Composite Measure (MSFC)" by Fischer JS, Jak JS, Kniker JE, Rudick RA and Cutter G)

STANDARDIZING MSFC ADMINISTRATION

The MSFC should be administered as close to the beginning of a study visit as possible, but definitely before the patient does a distance walk. MSFC components should be administered in the following order:

1. Trial 1, Timed 25-Foot Walk
2. Trial 2, Timed 25-Foot Walk
3. Trial 1, Dominant Hand, 9-HPT
4. Trial 2, Dominant Hand, 9-HPT
5. Trial 1, Non-Dominant Hand, 9-HPT
6. Trial 2, Non-Dominant Hand, 9-HPT
7. PASAT

INSTRUCTIONS FOR THE TIMED 25 FOOT WALK

DESCRIPTION

The Timed 25-Foot Walk is a quantitative measure of lower extremity function. It is the first component of the MSFC administered at each visit. The patient is directed to one end of a clearly marked 25-foot (7.62 m) course and is instructed to walk 25 feet (7.62 meter) as quickly as possible, but safely. The task is immediately administered again by having the patient walk back the same distance. Patients may use assistive devices when doing this task. In clinical trials, it is recommended that the study physician selects the appropriate assistive device for each patient.

MATERIALS NEEDED

Stopwatch, clipboard, Timed 25-Foot Walk Record Form, marked 25-foot (7.62 m) distance in an unobstructed hallway, assistive device (if needed).

TIME LIMIT PER TRIAL

3 minutes (180 seconds) per trial.

INSTRUCTIONS FOR THE 9-HOLE PEG TEST

DESCRIPTION

The 9-HPT is a quantitative measure of upper extremity (arm and hand) function. The 9-HPT is the second component of the MSFC to be administered. Both the dominant and non-dominant hands are tested twice (two consecutive trials of the dominant hand, followed

immediately by two consecutive trials of the non-dominant hand). It is important that the 9-HPT be administered on a solid table (not a rolling hospital bedside table) and that the small rubber feet are fixed under the 9-HPT apparatus (or the apparatus be anchored by other method).

MATERIALS NEEDED

9-HPT apparatus, stopwatch, clipboard, 9-HPT Record Form

TIME LIMIT PER TRIAL

5 minutes (300 seconds)

INSTRUCTIONS FOR THE PACED AUDITORY SERIAL ADDITION TEST (PASAT)

DESCRIPTION

The PASAT is a measure of cognitive function that specifically assesses auditory information processing speed and flexibility, as well as calculation ability. The PASAT is the last measure administered at each visit. It is presented on audio CD to control the rate of stimulus presentation. Single digits are presented every 3 seconds and the patient must add each new digit to the one immediately prior to it. The test result is the number of correct sums given (out of 60 possible). To minimize familiarity with stimulus items in clinical trials and other serial studies, two alternate forms have been developed; the order of these should be counterbalanced across testing sessions.

MATERIALS NEEDED

CD player, audio CD with PASAT stimuli, clipboard, PASAT Record Forms

NOVARTIS

Clinical Development

FTY720D (fingolimod)

Clinical Trial Protocol CFTY720D2312

A 12-month, randomized, rater- and dose-blinded study to compare the efficacy and safety of fingolimod 0.25 mg and 0.5 mg administered orally once daily with glatiramer acetate 20 mg administered subcutaneously once daily in patients with relapsing-remitting multiple sclerosis

RAP Module 3 – Detailed Statistical Methodology

**Author:**	[redacted]
**Document type:**	RAP Documentation
**Document status:**	Final Version 2.6 Addendum 1
**Release date:**	02Nov2018
**Number of pages:**	67

Property of Novartis
Confidential
May not be used, divulged, published or otherwise disclosed
without the consent of Novartis

Document History – Changes compared to previous version of RAP module 3.

Version	Date	Changes
Final V2.1	28MAY2014	Changes to follow updated protocol amendment 1
Final V2.2	06APR2015	Added sensitivity analysis to the primary analysis by excluding one misconduct site
	26AUG2015	Updated per protocol final amendment 2
Final V2.3	17FEB2017	Updated the sample size calculation per study early termination approved by FDA in December 2016
	24MAR2017	Added general statistical analysis plan for the sub-study, Section 10
Final V2.4	20OCT2017	Added two Canada provinces (British Columbia and Manitoba) in Section 3.9 (Pooling of centers).
		Updated the sections related to C-SSRS per specific eCRF implemented via IVRS for this study. There is no visit information in IVRS data, so a visit window is added for C-SSRS.
	06DEC2017	Clarified nominal visits for dermatology and OCT assessment to be consistent with the TLF shells.
	28DEC2017	Updates to MRI model specifications to match with the protocol.
	10JAN2018	Vital signs: updated per the study data collection specifics (sitting for old CRFs and supine/standing for later CRFs). Data summaries are updated to present for each position separately.
	10JAN2018	Bradycardia event from first dose monitoring: Clarified that bradycardia events that do not have symptoms are not collected in the bradycardia CRFs and will be identified from the Adverse Event CRF.
	07FEB2018	Added a sentence to specify MedDRA version.
		Final sample number is updated to 1064, instead of 1063.
		Added wording about two discontinuation listings per FDA request.
	09FEB2018	Moved C-SSRS section to Safety Section 8.9.
	02MAY2018	Clarified last dose date definition.
		Clarified regular ECG summaries at Baseline and Month 12/EOT to align with RAP M7.1 and study protocol.
		Added the decision about following the old Novartis PD process, instead of the new Novartis PD process.
		Removed the figures for individual subjects meeting liver function safety criteria and those meeting hematology notable criteria as they do not provide any additional information to the existing abnormality tables per dry run 1 team decision.
		Removed the section about baseline comparability tests to be consistent with the recently completed FTY2201 study and considering this is a randomized study.
	04MAY2018	Section 6 (Sub-study analyses): Removed the original description about a separate sub-study. Added details about the T cell subsets

Version	Date	Changes
		provided by Q2 lab and also descriptive statistics recommended by Ryan Winger.
		Section 10 (Sub-study analyses): Duplicate section, removed.
		Section 11 (Documentation of changes from the protocol): Added this new section to document secondary and exploratory objectives for sub-study will not be performed due to limited data or not data collected.
	10MAY2018	Updated Section 6 per Nadia comments (NVS).
		Updated to remove 'unenhanced' in the label for T1 lesion to be consistent with source data label and also per Susanne comment (NVS).
	14MAY2018	Updated Section 3.9 Pooling of Centers by added a few more US states per the final clinical document.
		Added a complete list of centers with geographic locations and region classification in Appendix 1.
Final 2.5	22Aug2018	For PFT section, a note is added to clarify how to handle a few outliers (unrealistic values) in the final analysis.
	28Aug2018	Section 8.1.2.1 Occurrence of SAEs and NSAEs: Added the entire section to add two tables to meet the CTSD requirement.
		Section 8.1.3 Data summaries for AEs: Added two bullets for the two new tables described in Section 8.1.2.1.
	10Sep2018	Section 3.6 Study Medication: Added data handling rule for partial end date for the last dosing interval.
		Section 3.8.7 Last dose day of randomized study medication: Added data handling rule for partial or completely missing last dose date on the CMP panel.
		Section 4.1.3 Time in study (years) for aggregate ARR: Updated to use the new definition by excluding FU visits and removing the truncation at 367 days.
		Section 4.2.1.2 Supportive analysis: Moved the old definition for time in study for ARR calculation to this section and kept it as one supportive analysis.
		Section 4.2.1.2 Supportive analysis: Added additional sensitivity analysis by defining confirmed relapse strictly based on EDSS scores.
		Section 4.2.2.1 MRI: Added sensitivity analyses to exclude baseline assessments > 45 days before randomization and end of treatment assessments > 45 days after last dose.
	13Sep2018	Section 8.1 Adverse Events: Added summaries for risk AEs and updated wording for occurrence rate of AEs to be consistent with study D2311.
Final 2.6	02Nov2018	Section 4.2.2.1: Added new analyses for annualized rate of brain atrophy (ARBA) at Month 12/end of treatment. This is to adjust for study duration and also other MRI covariates due to some differences between treatment groups.

Table of contents

Table of contents 4
List of tables 6
1 Statistical methods planned in the protocol and determination of sample size 8
2 Statistical and analytical plans 8
3 Subjects and treatments, general rules and definitions 9
3.1 Analysis sets 9
3.2 Subject disposition 9
3.3 Protocol deviations 10
3.4 Background and demographic characteristics 11
3.5 MS disease and medical history 11
3.6 Study medication 13
3.7 Concomitant medication/therapy 13
3.8 Visit windows and cutoffs for efficacy and safety analyses 15
3.8.1 Study Day 1 and other study days 15
3.8.2 Screening/Baseline 16
3.8.3 Nominal visits and visit windows 16
3.8.3.1 Nominal visits 16
3.8.3.2 Visits windows 17
3.8.4 Time points for first dose monitoring ECG and Vital signs 18
3.8.5 Safety data cutoff and visit windows for summaries on follow-up set 19
3.8.6 Multiple assessments within visit windows 19
3.8.7 Last dose day of randomized study medication 20
3.9 Pooling of centers 20
4 Efficacy evaluation 21
4.1 Primary and key secondary efficacy variables 21
4.1.1 Primary efficacy variable 21
4.1.2 Key secondary efficacy variable 21
4.1.3 Time in study (years) for aggregate ARR 21
4.2 Statistical methodology 22
4.2.1 Primary analysis 22
4.2.1.1 Multiplicity adjustment for statistical hypothesis testing 23
4.2.1.2 Supportive analyses of primary endpoint 24
4.2.1.3 Handling dropouts in ARR calculations 26
4.2.1.4 Missing MS relapse dates 27
4.2.2 Analyses of secondary and exploratory variables 27

4.2.2.1 Magnetic resonance imaging (MRI) 28
4.2.2.2 Relapses 30
4.2.2.3 Multiple Sclerosis Functional Composite Measure 31
4.2.2.4 Symbol Digit Modality Test 33
4.2.2.5 EDSS Scores 33
5 Pharmacokinetic/pharmacodynamics evaluations 33
6 Regulatory immune-cells substudy analyses 33
7 Health-related Quality of Life (QoL) 34
7.1 PRIMUS-Activities 34
7.2 MSIS-29 35
7.2.1 Physical impact score 35
7.2.2 Psychological impact score 35
7.2.3 Missing data 35
7.3 TSQM (Version 1.4) 36
8 Safety evaluation 37
8.1 Adverse events 37
8.1.1 Adverse event date imputation 37
8.1.2 Adverse events 38
8.1.2.1 Occurrence of SAEs and NSAEs (non-serious AEs) 39
8.1.3 Data summaries for AEs 40
8.2 Laboratory data 40
8.2.1 SI Units 40
8.2.2 Clinically notable criteria 40
8.2.3 Data Summaries for lab tests 43
8.3 Vital signs 44
8.3.1 Vital signs measurements 44
8.3.2 Vital signs notable criteria 44
8.3.3 Data summaries for vital signs 45
8.4 Electrocardiograms (ECG) 45
8.4.1 Corrected QT interval 45
8.4.2 Abnormality criteria for corrected QT interval 45
8.4.3 ECG findings 46
8.4.4 Data summaries for ECG data 46
8.5 Pulmonary function tests (PFT) 46
8.5.1 Predicted values and units of PFTs 46
8.5.2 Percent predicted PFTs 47
8.5.3 Data summaries for PFTs 47
8.6 Ophthalmic evaluations 48

8.6.1 Data summaries for ophthalmic evaluations 48
8.7 Dermatology assessment 48
8.8 First dose (second dose or restart dose) of fingolimod monitoring 49
8.8.1 Vital signs...49
8.8.2 ECG...49
8.8.3 Dose monitoring experience 49
8.8.4 Data summaries for first dose monitoring..49
8.9 Columbia Suicide Severity Rating Scale (C-SSRS)...50
9 Interim analyses..52
10 Determination of sample size 52
11 Documentation of changes from the protocol 53
12 Clinical Study Report - Appendix 16.1.9 Documentation of statistical methods 54
12.1 Statistical methods and analysis outputs...54
12.1.1 Statistical methods 54
12.1.1.1 Negative binomial regression model...54
12.1.1.2 Rank Analysis of Covariance (ANCOVA) and Rank Analysis of Variance (ANOVA)...........55
12.1.1.3 Logistic regression for proportion variables 57
12.1.1.4 Survival analysis for time to event variables 58
12.1.1.5 Between-treatment group comparisons for other efficacy variables.....................59
12.1.1.6 Analysis of Covariance (ANCOVA) for quality of life (QoL) variables 60
12.1.1.7 Wilcoxon sign test for quality of life (QoL) variables...............................61
12.1.2 Statistical analysis outputs 62
13 References 62
14 Appendices 63
14.1 Appendix 1: Geographic location (state/providence and country) of the study centers.........63

List of tables

Document History – Changes compared to previous version of RAP module 3. 2
Table 1-1 The analysis sets and severity codes 10
Table 1-1a The derivation formula for creatinine clearance 11
Table 1-2 Display of drug names 12
Table 1-3 Definitions of time-intervals used to summarize exposure to study medication 13
Table 1-4 CMD date imputation..15
Table 2-1 Visit windows for vital signs and laboratory values 17
Table 2-2 Visit windows for PFTs 18

Table 2-3 Visit windows for ophthalmologic examination (excluding OCT)..........................18
Table 2-4 Visit windows for ECG..18
Table 2-5 Visit windows for C-SSRS...18
Table 3-1 Primary efficacy endpoint, supportive endpoints & analyses..................................24
Table 3-2 Example of imputation...26
Table 3-3 MRI endpoints & analyses...28
Table 3-4 Relapse-related endpoints & analyses...30
Table 3-5 MSFC endpoints & analyses..32
Table 3-6 List of T Cell Subsets Measured by Flow Cytometry..34
Table 4-1 AE date imputation 38
Table 4-2 Criteria for clinically notable abnormalities 41
Table 4-3 The lower limit of detections of laboratory parameters...42
Table 4-4 Abnormal criteria 42
Table 4-5 Criteria for clinically notable vital signs..44
Table 4-6 Abnormality criteria for corrected QT interval...45
Table 12-1 Confidence Interval calculations...59

1 Statistical methods planned in the protocol and determination of sample size

Data will be analyzed by PPD statisticians and statistical programmers assigned to this study according to the data analysis section 9 of the study protocol and protocol amendments which is available in Appendix 16.1.1 of the CSR. Important information is given in the following sections and details are provided, as applicable, in Appendix 16.1.9 of the CSR.

2 Statistical and analytical plans

Unless otherwise stated, summary tables/figures/listings will be on all subjects included in the respective analysis set.

Categorical data will be presented as frequencies and percentages. For continuous data, mean, standard deviation, median, 25th and 75th percentiles, minimum, and maximum will be presented.

The contrast for between-treatment comparisons (ex. ARR, ARR-ratio) and corresponding p-values will be provided in section 16 SAS output tables. But between treatment comparison and corresponding p-value for the comparison between the two FTY doses (ex. ARR-ratio) will not be presented in the in-text and post-text tables. On in-text and post-text tables, the comparisons for FTY720 0.5 mg vs GA 20 mg, FTY720 0.25 mg vs GA 20 mg and the corresponding p-values will be displayed. In addition, the p-values generated from analysis models will be displayed up to 4 decimal places for reuse in publication as well as in CSR.

The primary objective of the study is to demonstrate that at least one dose (tested hierarchically 0.5 mg followed by 0.25 mg) of fingolimod is superior to glatiramer acetate 20 mg SC in reducing the ARR up to 12 months in subjects with relapsing-remitting MS.

A substudy will be conducted in about 10 select sites in the US and Canada in approximately 24 subjects randomized to the FTY arm to assess the impact of chronic fingolimod treatment on the proportions of Th17 and Treg cells in the blood of MS subjects as part of a post-approval commitment for the CHMP of the European Medicines Agency. The primary objective of the immune-cell substudy is to measure the effect of fingolimod treatment on the ratio of Th17 and Treg cells to total lymphocyte number upon reaching steady state and under chronic exposure.

A general note on considering country or region in an efficacy analysis model: An adjustment for country or region will be considered in the initial model. If the initial model can not generate estimates with region as a factor, then country or region will be removed from the model for further specified analysis.

3 Subjects and treatments, general rules and definitions

3.1 Analysis sets

The following analysis sets will be used:

- **Randomized set (RS):** consists of all subjects who are assigned randomization numbers. The subjects in this set are called randomized subjects. This set will be used to summarize subject disposition, demographic and baseline characteristics, and protocol deviation information. Subjects will be grouped according to randomized treatment.
- **Full-analysis set (FAS):** Consists of all subjects who are randomly assigned and take at least 1 dose of study drug. Following the intent-to-treat principle, subjects will be grouped according to the assigned treatment at randomization. Efficacy analyses will be performed on the full-analysis set unless otherwise notified.
- **Per-protocol set (PPS):** Consists of all subjects in the FAS who do not have any major protocol deviations that could confound the interpretation of analyses conducted on the FAS. Major protocol deviations will be determined according to the predefined protocol deviation criteria before treatment unblinding. Any efficacy data after study drug discontinuation will be excluded. The per-protocol set will only be used for the supportive analyses of the primary efficacy variable.
- **Safety set (SS):** Consists of all subjects in the FAS who take at least 1 dose of study drug. Subjects will be analyzed according to the treatment they have actually taken. Safety and tolerability analyses will be performed on the safety set unless otherwise specified.
- **Follow-up set (FUS):** The follow-up set consists of all subjects in the safety set who have follow-up visit data or who have at least 1 safety assessment (AEs, laboratory test, vital sign measurement, PFTs or ophthalmology assessments) 46 days or more than 46 days after study drug discontinuation. Subjects will be grouped in the same way as previously described for the analysis on the safety set. Only the safety follow-up data analysis will be performed on the follow-up set.

3.2 Subject disposition

Subject disposition will be summarized on the randomized set. The number and proportion of subjects who complete the study or discontinue the study prematurely along with the reason for discontinuation will be presented. The number and proportion of subjects who discontinue the study drug prematurely along with the reason for study drug discontinuation will be summarized and presented separately. A listing will also be provided with treatment group and subject number.

Additionally two types of subject discontinuation listings will be provided per FDA request.

- Discontinuation of study drug after which patients are still allowed to continue to have efficacy/safety assessment according to abbreviated schedule without receiving study drug treatment until the end of study.
- Discontinuation from the study in which no further safety/efficacy will be assessed except for follow-up.

Due to the non-specific nature of the HA communication, both types of listings are provided with the additional detail requested (under the heading "**Relevant information related to reason**").

Reasons for screen failure will be summarized for all screened subjects.

3.3 Protocol deviations

Protocol deviations will be summarized by treatment group on the randomized set. The major protocol deviations (severity codes of 0, 1, and 8) resulting in subjects' exclusion from analysis sets are defined in study CFTY720D2312 VAP Module 3 – Protocol Deviation. Please refer to study VAP M3 for details.

The analysis sets and severity codes used in the trial programming are listed in Table 1-1, which includes the analysis set codes 1-5 and the severity codes 0, 1, 5, 8 and 49 for the double-blind treatment period.

Table 1-1 The analysis sets and severity codes

Analysis set codes	Severity Codes				
1 = Randomized set<br>2 = Full analysis set<br>3 = Per protocol set<br>4 = Safety set<br>5 = Follow-up set	0 = Exclude subject from all efficacy analyses<br>1 = Exclude subject from per-protocol analysis<br>5 = Exclude subject from all safety analyses<br>8 = Exclude subject from all analyses<br>49 = Include subject in all analyses				
	**Deviation Severity Codes**				
**Analysis set (code)**	**0**	**1**	**5**	**8**	**49**
Randomized set (1)	*Yes*	*Yes*	*Yes*	*Yes*	*Yes*
Full analysis set (2)	*No*	*Yes*	*Yes*	*No*	Yes
Per protocol set (3)	*No*	*No*	*Yes*	*No*	Yes
Safety set (4)	*Yes*	*Yes*	*No*	*No*	Yes
Follow-up set (5)	*Yes*	*Yes*	*No*	*No*	*Yes*

Note: 'Yes' means include in the analysis set, 'No' means exclude from the analysis set.

Additionally, unblinding per protocol due to safety reasons may occur during the study. If a subject's treatment code is revealed to any observer or study center personnel who are intended to be blinded during the evaluation period (as marked on the study completion CRF page), that subject will also be excluded from the per-protocol set. A list of those subjects will also be presented.

Note: Novartis study team discussed and agreed that FTYD2312 should follow the old PD process due to the following key reasons:

- FTYD2312 Data Management has been following the old process and data is collected on paper forms
- The dates of the FTYD2312 Initial SAP (10/18/2011) and the 1st amendment of the SAP (6/3/2014) are prior to the roll-out date specified for the new PD Process (3/28/2016

3.4 Background and demographic characteristics

Background and demographic characteristics include age at screening derived from date of birth and the screening assessment date, gender, race, and ethnicity collected on the demography CRF, height, and body weight recorded at baseline on the vital signs CRF, and body mass index (BMI) calculated as (body weight in kilograms) / (height in meters)^2.

These variables recorded at baseline will be summarized on the randomized set by presenting frequency distributions (for categorical variables) or summary statistics (for continuous variables).

It is anticipated that in some cases baseline body weight may be missing. For baseline characteristics, the algorithm to derive baseline body weight is as follows: it is the last assessment of any evaluations done up to the time of first dose; if there is no such assessment, it will be the first assessment of any evaluations done after first dose. In the situation that baseline and post-baseline weight summaries are part of the vital sign summaries, missing body weights will be treated as missing.

In the computation of creatinine clearance using the Cockcroft-Gault formula, the last assessment of body weight made on or prior to the day when the subject takes the laboratory test will be used.

Table 1-1a The derivation formula for creatinine clearance

Creatinine clearance [mL/min] using Cockcroft-Gault formula (Cockcroft and Gault, 1976)	$= (140 - A) \times W \ / (72 \times C) \times G$<br>Where<br>*A* is age [years]<br>*W* is body weight [kg]<br>*C* is the serum concentration of creatinine [mg/dL]<br>*G* is a gender: *G*=1 for males and *G*=0.85 for females.
Creatinine clearance [mL/min] using abbreviated MDRD formula (Levey et al, 2000)	$= 186.3 \times C^{-1.154} \times A^{-0.203} \times E \times S$<br>Where<br>*C* is the serum concentration of creatinine [mg/dL],<br>*A* is age [years],<br>*E* is ethnicity: *E*=1.212 if patient is black, else E=1,<br>*S* is gender: *S*=0.742 (if patient is female), else S=1.

3.5 MS disease and medical history

Any condition entered on the relevant medical history/current medical conditions CRF will be coded using the MedDRA dictionary.

Relevant medical history/current medical condition, pulmonary history, MS disease history and duration of the disease, MS related eye history, MS medication history (including history of disease-modifying drugs and other drugs), MS symptoms and number of relapses in the

past 2 years before study enrolment, and MS disease baseline characteristics including baseline MRI assessments and baseline EDSS will all be summarized on the randomized set.

The relevant medical history and continuing medical conditions will be summarized by primary system organ class (SOC) and preferred term (PT). MS medical history of other drugs will be summarized by preferred term.

For the MS disease history summary, duration of MS since diagnosis (years) will be derived as follows: (the first dose date – the MS diagnosis date + 1)/365.25; duration of MS since first symptom (years) will be derived as follows: (the first dose date – the first MS symptom date + 1)/365.25; time since the onset of the most recent relapse (months) will be derived as follows: (the first dose date – the most recent relapse onset date + 1)/30.

For the MS diagnosis date and the first MS symptom date, the following rule will be used when imputing a partial date (complete missing date will not be imputed):

a) Only year yyyy is available. The imputed date will be 1-July-yyyy.

b) Both year yyyy and month mmm are available. The imputed date will be 15-mmm-yyyy.

For the most recent relapse onset date, the following rule will be used when imputing a partial date:

a) Only year yyyy is available. The imputed date will be 1-Jan-yyyy.

b) Both year yyyy and month mmm are available. The imputed date will be 01-mmm-yyyy.

For the MS medication history of disease-modifying drugs (DMDs), the treatment naïve subjects are defined as those who never took the approved MS medications or other MS treatment as specified in Table 1-2.

Any subject previously treated with any MS therapies (marked as ‘Yes’ on “Previous MS treatment” CRF page) will not be considered treatment naïve. When displaying these drugs in the tables, the names specified in Table 1-2 will be used.

Table 1-2 Display of drug names

Drug name collected in database	Drug name to be used in display
Azasan®, Azimune, Azoprin, Azathine, Azoran, Imuzat, Zymurine, Imuran®	Azathioprine
Interferon beta-1a (Avonex®)	Interferon beta-1a i.m.
Interferon beta-1a (Rebif®)	Interferon beta-1a s.c.
Interferon beta-1b (Betaferon®/Betaseron®/Extavia®)	Interferon beta-1b s.c.
Glatiramer acetate (Copaxone®)	Glatiramer acetate
Natalizumab (Tysabri®/Antegren®)	Natalizumab
Rheumatrex®, Trexall®, Amethopterin, MTX	Methotrexate
Aubagio	Teriflunomide
Tecfidera	Dimethyl fumarate (BG12)
New approved drugs	

Drug name collected in database	Drug name to be used in display
Other	Other MS medications

3.6 Study medication

Duration of exposure to randomized study medication will be summarized on the safety set by presenting the number (and percentage) of subjects being exposed for a minimum duration of time as specified in Table 1-3 Duration in terms of number of days as shown in the second column will be used in the summary for the display of duration.

Duration of exposure is the number of days on study drug during the core study phase. The days when the subject does not take the study drug will be excluded. That is, duration of exposure will exclude periods of temporary interruption of study medication. Note: for the last dosing interval entered on the Dosage Administration Record CRF, if the dosing end date is partial date, impute the first day for missing day and January for missing month.

Summary statistics of duration of exposure to study medication will also be presented, which included mean, SD, minimum, maximum, etc.

For each treatment group, the patient-years, which will be calculated as (the sum of the number of days on study drug for all subjects in the group)/365.25, will also be presented.

Table 1-3 Definitions of time-intervals used to summarize exposure to study medication

Duration	Duration in terms of number of days
Any Exposure	≥1 day
≥1 week	≥7 days
≥2 weeks	≥14 days
≥1 month	≥30 days
≥2 months	≥60 days
≥3 months	≥90 days
≥6 months	≥180 days
≥9 months	≥270 days
≥12 months	≥360 days

3.7 Concomitant medication/therapy

Free-text records on the Concomitant medications / significant non-drug therapies CRF will be coded using NOVDTD drug dictionary (Novartis updated version of WHODRUG).

All medications recorded on the Concomitant medications / significant non-drug therapies form will be classed as prior or concomitant medications and summarized separately, in alphabetical order, by ATC class and preferred term. Tables will show number of subjects (receiving at least one drug of a particular ATC class and at least one drug in a particular preferred term) and percentages.

Prior medications will be drugs taken prior to first dose of randomized study medication but not at any time after baseline; any medication given at least once between the day of first dose of randomized study medication and the last day of randomized study medication will be a concomitant medication. Concurrent medications could start before first dose of randomized

study medication but continue through after the first dose of randomized study medication. Any medication started after the discontinuation of randomized study medication will not be considered concomitant medication. Medications will be categorized into one (and only one) of above classes based on imputed start and end dates.

Concomitant medication (CMD) date imputation will be based on a comparison between the partial CMD start date and the treatment start date and if necessary, on the CMD type as well which is also recorded on the CRF (i.e., the variable CMDTYP1C with 3 possible outcomes: prior medication, concomitant medication, or prior/concomitant medication). Date comparisons will be based on the year and month values only (i.e., day values will be ignored) as shown in Table 1-4.

1. If the CMD start date year value is missing, the following imputation rules will apply.
    a. If the CMD type is either prior medication or prior/concomitant medication, the CMD start date will be imputed as the date one day before the treatment start date.
    b. If the CMD type is concomitant medication or missing, the CMD start date will be imputed as the date one day after the treatment start date.
2. If the CMD start date year value is less than the treatment start date year value, then the CMD must have started before treatment.
    a. If the CMD month is missing, the CMD start date will be imputed as the mid year date (01JulYYYY).
    b. If the CMD month is not missing, the CMD start date will be imputed as the mid month date (15MONYYYY).
3. If the CMD start date year value is greater than the treatment start date year value, then the CMD must have started after treatment.
    a. If the CMD month is missing, the CMD start date will be imputed as the year start date (01JanYYYY).
    b. If the CMD month is not missing, the CMD start date will be imputed as the month start date (01MONYYYY).
4. If the CMD start date year value is equal to the treatment start date year value, then the month values will be compared as follows.
    a. If the CMD month is either missing or equal to the treatment start month, the following imputation rules will apply.
        i. If the CMD type is either prior medication or prior/concomitant medication, the CMD start date will be imputed as the date one day before the treatment start date.
        ii. If the CMD type is concomitant medication or missing, the CMD start date will be imputed as the date one day after the treatment start date.
    b. If the CMD month is less than the treatment start month, the CMD start date will be imputed as the mid month date (15MONYYYY).

c. If the CMD month is greater than the treatment start month, the CMD start date will be imputed as the start month date (01MONYYYY).

Table 1-4 CMD date imputation

	MON MISSING	MON < CFM	MON = CFM	MON > CFM
YYYY MISSING	(B)<br>Uncertain	(B)<br>Uncertain	(B)<br>Uncertain	(B)<br>Uncertain
YYYY < CFY	(D)=01JULYYYY<br>Before Treatment Start	(C)=15MONYY<br>Before Treatment Start	(C)=15MONYY<br>Before Treatment Start	(C)=15MONYY<br>Before Treatment Start
YYYY = CFY	(B)<br>Uncertain	(C)=15MONYY<br>Before Treatment Start	(B)<br>Uncertain	(A)=01MONYYYY<br>After Treatment Start
YYYY > CFY	(E)= 01JANYYYY<br>After Treatment Start	(A)=01MONYYYY<br>After Treatment Start	(A)=01MONYYYY<br>After Treatment Start	(A)=01MONYYYY<br>After Treatment Start

Before Treatment Start	Partial indicates date prior to Treatment Start Date
After Treatment Start	Partial indicates date after Treatment Start Date
Uncertain	Partial insufficient to determine relationship to Treatment Start

LEGEND:	
(A)	01MONYYYY
(B)	IF CMDTYP1C IN (1,3) THEN TRTSTD-1<br>ELSE IF CMDTYP1C in(.,2) THEN TRTSTD+1
(C)	15MONYYYY
(D)	01JULYYYY
(E)	01JANYYYY

3.8 Visit windows and cutoffs for efficacy and safety analyses

This section defines the data cutoffs within which data will be included in the analysis and time points for which data will be summarized.

Due to the study design, both data collected at Month 12 visit as end of the treatment (EOT) (also as end of study (EOS) visit) and data collected at the study drug discontinuation visit as EOT will be recorded in the CRF at EOT (corresponding to Visit 777 in the database). For subjects who discontinue study drug early but continue through the study per abbreviated schedule, the EOT visit and EOS visit may be different, in which case data collected at the EOS visit will be recorded in the CRF at study completion visit (corresponding to Visit 778 in the database). For subjects whose EOT visit and EOS visit are the same, data will be collected only once at the EOT visit. Therefore, there will be no corresponding Visit 778 in the database for these subjects. In the subject completion (CMP) data set, data collected from the study drug discontinuation CRF (Visit 777) and study completion CRF (Visit 778) will be differentiated by visit number although there is no actual study visit corresponding to Visit 778 for subjects whose EOT visit and EOS visit are the same. .

3.8.1 Study Day 1 and other study days

The first day of administration of randomized study medication (i.e., the first dose date of the study drug) is defined as Study Day 1 or Day 1.

All other study days will be labeled relative to Day 1. For event dates on or after Day 1, study day for an event date is calculated as (event date – first dose date +1), which could be Day 1, Day 2, Day 3, etc. For event dates before Day 1, study day for an event date is calculated as

(event date – first dose date), which could be Day -1, Day -2, etc., referring to one day, two days, etc., before Day 1, respectively. Duration of an event will be calculated as (event end date – event start date + 1).

One month is defined as 30 days.

3.8.2 Screening/Baseline

Screening refers to any procedures (e.g. checking inclusion and exclusion criteria) performed prior to first dose date. Subject informed consent is generally obtained prior to any assessment. Any assessment obtained prior to first dose date will be called screening assessment.

In general, a baseline value refers to the last measurement made prior to the administration of the first dose of the study drug. Assessments made on Day 1 may occur before or after the first dose. The following rules will be applied to determine which record is the last measurement prior to the first dose (i.e., the baseline value).

- Consider all records with an assessment date before or on the first dose date. Baseline is the last record after sorting all above records with respect to assessment date, visit and repeat visit number.
- Some data specific rules for determining baseline will be applied as described below or in data specific sections if applicable.

For vital signs, the average of the 3 readings from Day 1 pre-dose at each position (sitting, supine or standing) will be used as baseline for that position. If unavailable, the last available average value from visits (including unscheduled) before the first dose will be used. For sitting pulse the last available single value before the first dose will be used as baseline.

For ECG, the Day 1 pre-dose value will be used as baseline. If unavailable, the last available (including unscheduled) value before the first dose will be used.

The pre-dose time point is the record at scheduled time point 0 (i.e., variable STM2N equals 0).

3.8.3 Nominal visits and visit windows

3.8.3.1 Nominal visits

For EDSS, MSFC, SDMT, PRO variables (except C-SSRS), dermatology exam, MRI and OCT, the nominal visit will be used in the by-visit summaries. Data from unscheduled visits for these assessments will not be summarized unless otherwise specified.

The end of treatment or early discontinuation visit (Visit 777) will be the nominal 12 month visit for subjects who complete the study on study drug. For subjects who discontinue the study drug prematurely, the end of treatment or early discontinuation visit (Visit 777), the follow-up visit (Visit 501) and the end of study visit (Visit 778) which are not timepoint specific visits will be remapped based on the following rules:

- If these visits fall into a specific visit window and the corresponding scheduled visit is missing, then these visits will be remapped to be that scheduled visit. The visit windows

are defined as within 5 days of the scheduled visit (+/-5 days from the target day at each visit).

- If these visits fall into a specific visit window and the corresponding scheduled visit (e.g., Visit X) is not missing, then these visits will be assigned a visit number with a decimal place where the visit number depends on whether the corresponding scheduled visit occurs before (e.g., Visit X.1 or Visit X.2 if Visit X.1 already exist etc.) or after these visits (e.g., Visit X-1.1 or Visit X-1.2 if Visit X-1.1 already exist etc.)
- If these visits do not fall into a specific visit window but fall in between 2 consecutive scheduled visit windows (e.g., between visit windows of Visit X and Visit X+1), then these visits will be assigned a visit number with a decimal place (e.g., Visit X.1 or Visit X.2 if Visit X.1 already exist etc.).

The remapped visits (e.g., Visit X) will be treated as nominal visits in the by-visit summaries.

For dermatology exam and OCT the above visits (Visit 777, Visit 501 and Visit 778) will not be remapped.

3.8.3.2 Visits windows

For data not listed in Nominal visit Section 3.8.3.1, visit windows will be defined and used in the by-visit summaries. Visit windows will only be defined for post-baseline visits and applied to post-baseline data (including both scheduled and unscheduled visits). Based on the study assessment schedule (see Table 6-1 and Table 6-2 in the study protocol), visit windows are defined by a set of days around each nominal visit target day. For any assessment, there are at most 6 scheduled visits around which visit windows will be created: Months 1, 3, 6, 9, 12 and Follow-up.

Visit windows for vital signs, lab, ophthalmologic examination (excluding OCT), pulmonary function tests (PFTs), ECG, and C-SSRS are provided in Table 2-1 to Table 2-5. These visit windows will not be applied to any data related to the first dose or second dose or restart dose monitoring which will be summarized separately.

When visit windows are used, all post-baseline visits will be re-aligned, i.e., they will be mapped into one of the visit windows. E.g., if a subject's *Month 1* visit is delayed and occurs on Day 47, then it will be re-aligned to visit window *Month 2*. As a result, it is possible that several assessments may fall into one particular visit window. Statistical approaches to handle multiple visits in a given visit window are described in Multiple assessments within visit windows Section 3.8.6.

Table 2-1 Visit windows for vital signs and laboratory values

Visit	Start day	Target Day	End day
Month 1	1	30	60
Month 3	61	90	135
Month 6	136	180	225
Month 9	226	270	315
Month 12	316	360	last dose date day +45

Follow-up	Last dose date day + 46	450	

Table 2-2 Visit windows for PFTs

Visit	Start day	Target Day	End day
Month 6	1	180	270
Month 12	271	360	last dose date day +45
Follow-up	Last dose date day + 46	450	

Table 2-3 Visit windows for ophthalmologic examination (excluding OCT)

Visit	Start day	Target Day	End day
Month 3	1	90	135
Month 6	136	180	270
Month 12	271	360	last dose date day +45
Follow-up	Last dose date day + 46	450	

Table 2-4 Visit windows for ECG

Visit	Start day	Target Day	End day
Month 12	1	360	last dose date day +45
Follow-up	Last dose date day + 46	450	

Table 2-5 Visit windows for C-SSRS

Visit	Start day	Target Day	End day
Screening	-45	NA	-1
Baseline	1	1	1
Month 1	2	30	60
Month 3	61	90	135
Month 6	136	180	225
Month 9	226	270	315
Month 12	316	360	last dose date day +45
Follow-up	Last dose date day + 46	450	

3.8.4 Time points for first dose monitoring ECG and Vital signs

For the first dose monitoring ECG, data will be summarized for the following time points:

- Day 1 pre-dose: day 1 ECG value at 0 hour per time label

- Day 1 post-dose (6 hours): day 1 ECG value at 6 hours per time label.
- Day 1 post-dose (>6 hours): the mean of all day 1 ECG values after 6 hours per time label (including unscheduled values).

Unscheduled ECG measured between the day 1 pre-dose and the 6 hours post-dose will not be summarized but reported in the data listing only. For the second dose monitoring ECG, data will be summarized similarly.

For the first dose monitoring vital signs, data will be summarized for the following time points:

- Day 1 pre-dose: day 1 vital sign value at 0 hour per time label
- 1 hour post-dose
- 2 hours post-dose
- 3 hours post-dose
- 4 hours post-dose
- 5 hours post-dose
- 6 hours post-dose

The vital sign values at specified hours per time label post-dose will be used. Extended first dose monitoring after the 6 hours post-dose will not be summarized but reported in the data listing only. For the second dose or restart dose monitoring vital signs, data will be summarized similarly.

3.8.5 Safety data cutoff and visit windows for summaries on follow-up set

For safety summaries on the safety set, only data which are within 45 days (5 times the half life of FTY720, 9 days) after the last dose of study drug will be considered.

For safety summaries on the follow-up set, all data (including assessments or events more than 45 days after the last dose of study drug) will be considered.

The visit windows below will be used in summaries on the follow-up set.

1) Last assessment on study drug
2) Day 1 to 45 after study drug discontinuation
3) Month 3 (day 46 to 135) after study drug discontinuation
4) Month 6 (day 136 to 270) after study drug discontinuation
5) Month 12 (day 271 to 540) after study drug discontinuation

For the visit window 1), last assessment on or before the last dose date will be used. For visit windows 2) to 5), assessments collected during the specified day intervals after the last dose date will be used.

3.8.6 Multiple assessments within visit windows

For visit windows defined in Visit windows Section 3.8.3.2, multiple records may exist in one particular visit window. The following rules (unless otherwise specified) will be applied to obtain one value per subject per visit window which will be used in the by-visit summaries.

- For quantitative variables (i.e., continuous variables for which summary statistics and change from baseline tables will be provided), the average of the multiple records will be used.
- For qualitative variables (i.e., binary or nominal variables for which incidence rates and frequency distribution tables will be provided), the worst record will be used. It is noted that in the analyses performed, worst case is always well defined.

Note that "Last assessment on study drug" is not like other visit windows but more like a single time point. The assessment to be summarized at that time point is the last on drug observation which is the last observation a subject has while he or she is on study drug. Therefore, the above multiple assessment rules do not apply.

In addition, the multiple assessment rules do not apply to the first dose/second dose monitoring data and the restart dose monitoring data.

For tables to display worst case scenario, such as shift tables, and on listings, multiple assessments will be used as they are and no average values will be taken.

3.8.7 Last dose day of randomized study medication

The last dose date of the randomized study drug will be collected on three CRFs:
1) The last date subject took study drug on the study completion CRF (CMP panel),
2) The last date subject took study drug on the study drug discontinuation CRF (CMP panel),
3) The last end date on the study drug administration record CRF (DAR panel).

The last dose date recorded in the CMP panel will be used. If the last dose date in the CMP panel is partial date, impute the first day for missing day and January for missing month. If the last dose date in the CMP panel is completely missing, the last dose date recorded on the DAR panel will be used.

3.9 Pooling of centers

Total 6 countries participate in this study (Argentina, Brazil, Canada, Chile, Mexico and US) with majority of subjects from US. Region will be defined by pooling of centers as follows and will be used in the analysis as a factor.

- Centers from Latin American countries (i.e. Argentina, Brazil, Chile, and Mexico) will be pooled as a region called Latin American region.
- Centers from US/Canada will be pooled according to their geographic location and the number of subjects randomized as follows.

    1. Centers from US states of CA, WA, OR, NV, AZ, MT, UT and Canadian western provinces of British Columbia, Alberta, Saskatchewan, and Manitoba will be pooled as a region called West region.
    2. Centers from states of TX, CO, NM, KS, OK, MO will be pooled as a region called Southwest region.

3. Centers from states of WI, IA, IL, MI, IN, OH, KY, WV will be pooled as a region called Midwest region.
4. Centers from US states of NJ, PA, MD, DC, DE, VT, NH, MA, CT, NY and Canadian Eastern provinces of Quebec, Ontario and Nova Scotia will be pooled as a region called Northeast region.
5. Centers from states of FL, TN, AL, GA, LA, VA, NC, SC, PR will be pooled as a region called Southeast region.

The pooling of centers is performed so that there will be sufficient number of subjects in each region. If a statistical model stratified by region does not converge due to small number of subjects or small number of events in one or more regions, then regions will be further combined as needed.

4 Efficacy evaluation

4.1 Primary and key secondary efficacy variables

4.1.1 Primary efficacy variable

The primary efficacy variable is the annualized relapse rate (ARR) which will be analyzed on both the full analysis set (primary analysis) and the per-protocol set (supportive analysis). The ARR can be calculated in two ways:

1. Group level (aggregate ARR)

The ARR of a treatment group is calculated as the sum of the number of confirmed relapses of all subjects in the group divided by the sum of the number of days in study of all subjects in the group and multiplied by 365.25.

The group level ARR is the primary efficacy variable.

2. Subject level

The ARR of an individual subject is calculated as the number of confirmed relapses divided by the number of days in study and multiplied by 365.25. The ARR of a treatment group is the mean of ARRs of all subjects in the group.

4.1.2 Key secondary efficacy variable

Not applicable.

4.1.3 Time in study (years) for aggregate ARR

Time in study is the number of days from the first study drug dose date to the study completion visit date or the last study drug dose date whichever occurs later. It will be calculated as:

Time in study = Max (Study completion visit date, last study drug dose date) – first study drug dose date + 1

Note that the study complete visit date is defined as follows:

1. For subjects who completed study drug, or completed the abbreviated schedule of assessments after discontinuing the study drug prematurely, the study completion visit date is the Visit 7/Month 12 visit date.
2. For subjects who discontinued study drug prematurely and did not complete the abbreviated schedule of assessments, the study completion visit date is the end of study (EOS) visit date. If a subject did not have an EOS visit date, then the last study visit date excluding follow-up visit will be used

The time in study (years) will be calculated as the number of days in study/365.25.

The confirmed relapses within this time period will be counted for each subject and will be used in the ARR computation.

4.2 Statistical methodology

4.2.1 Primary analysis

The 2 doses of fingolimod will be tested hierarchically vs. glatiramer acetate in a step-down procedure. For each of the 2 fingolimod doses, the null hypothesis is that there is no difference in the ARRs between subjects treated with fingolimod and those treated with glatiramer acetate versus the alternative hypothesis that there is a difference between the 2 treatment groups. In order to preserve the Type I experiment-wise error rate, the null hypothesis will be rejected if the observed *p*-value for the between-treatment comparison is less than the significance level as specified in the multiplicity adjustment procedure described later in this section.

H_{01}: $\mu_{\text{FTY 0.5 mg}} = \mu_{\text{glatiramer acetate}}$ versus H_{A1}: $\mu_{\text{FTY 0.5 mg}} \neq \mu_{\text{glatiramer acetate}}$

H_{02}: $\mu_{\text{FTY 0.25 mg}} = \mu_{\text{glatiramer acetate}}$ versus H_{A2}: $\mu_{\text{FTY 0.25 mg}} \neq \mu_{\text{glatiramer acetate}}$

No formal hypothesis will be tested between the 2 fingolimod doses because the study is not powered to detect a difference in treatment effect between the 2 fingolimod doses.

The hypotheses will be tested using a negative binomial regression model with log link, using treatment as main effect, using the number of relapses in the previous year before study enrollment, baseline EDSS, and baseline Gd + T1 lesion count and country (or region) as covariates.

In the negative binomial regression model, the response variable is the number of confirmed relapses for each subject. The subject's time in the study (natural log of time in years) is used as an offset variable to obtain the ARR, adjusted for the varying lengths of subject's time in the study (time in years). Study centers will be pooled to country or region (based on geographical proximity and/or known or expected regional differences in medical care) in order to minimize the impact of low enrolling centers/countries on the analysis (such as non-convergence of the analysis model). Details of pooling will be provided in the statistical

analysis plan before database lock. The SAS procedure GENMOD (or other software with similar functionality) will be used to conduct the analysis.

The treatment effect of each dose of fingolimod versus glatiramer acetate will be presented as an ARR ratio ($\hat{\mu}_{FTY} / \hat{\mu}_{GA}$) with corresponding 95% confidence intervals and p values. In addition, the relative reduction in ARR will be presented as “percentage change,” calculated as ($\hat{\mu}_{FTY} / \hat{\mu}_{GA}$ -1).

The primary analysis will be conducted on the full analysis set. Only confirmed relapses will be counted.

4.2.1.1 Multiplicity adjustment for statistical hypothesis testing

The primary hypotheses tests will be tested using a hierarchical step-down procedure to control the experiment-wise error rate.

This study is designed to compare each of 2 doses of fingolimod to glatiramer acetate based on ARR. There are 2 hypotheses being tested (H01, H02).

Because it is highly likely that the approved dose of fingolimod (0.5 mg) is more efficacious than the lower dose (0.25 mg), the approved dose of fingolimod is initially tested against glatiramer acetate at a 2-sided significance level of 0.05. Only if this initial test H01 is rejected, will the low dose of fingolimod, be tested against glatiramer acetate also at a 2-sided significance level of 0.05.

Different scenarios of the anticipated treatment effect between fingolimod 0.5mg or 0.25mg versus glatiramer acetate have been evaluated based on the available data on fingolimod and the cumulative literature on glatiramer acetate. A 30% treatment benefit of fingolimod 0.5mg over glatiramer acetate could be anticipated on the basis of double-blind, 24-months, placebo-controlled studies of glatiramer acetate (Johnson et al, 2001) and fingolimod [Study CFTY720D2301] alone. However, more recent studies on glatiramer acetate, in a less active population, in a total of over 3000 patients, suggest that the efficacy of glatiramer acetate on ARR and other endpoints is similar to that of IFN beta-1a SC (REGARDS) or IFN beta-1b SC (BEYOND). The observed relapse rates on glatiramer acetate were 0.29 in REGARDS and 0.34 in BEYOND, compared to 0.3 (INF beta-1a SC) and 0.33 (INF beta-1b SC), respectively. In Study FTY720D2302, a double-blind 1-year study, fingolimod 0.5 mg was directly compared to IFN beta-1a IM. The ARR (0.21) in patients treated with fingolimod 0.5 mg was reduced by 52% compared to the ARR (0.43) in patients treated with INF beta-1a IM. Overall, the reduction in ARR in patients treated with fingolimod 0.5 mg compared to those treated with glatiramer acetate can be expected in the range of 30% to 50%. For the purpose of this study, a 35% lower ARR in patients treated with fingolimod 0.5 mg (ARR=0.195) compared to those treated with glatiramer acetate (ARR=0.30) is assumed.

Fingolimod 0.25 mg has never been tested in a clinical trial. Based on PK/PD modeling a 14% higher ARR in patients on fingolimod 0.25 mg compared to those on fingolimod 0.5 mg is anticipated. However, the uncertainty of this estimate is substantial. A 95% confidence interval from the PK/PD model for the ARR in patients on fingolimod 0.25 mg ranges from ARR=0.183 to ARR=0.303. For the purpose of this study, an ARR of 0.225 is assumed in patients treated with fingolimod 0.25 mg, which corresponds to an increase in ARR of

approximately 15% compared to fingolimod 0.5 mg and a relative treatment effect of 25% compared to glatiramer acetate.

4.2.1.2 Supportive analyses of primary endpoint

Supportive analyses will be performed for the primary endpoint as well as some variations of the primary endpoint (for sensitivity analysis purpose). All primary and supportive analyses are summarized below and in Table 3-1. The full analysis set will be used unless otherwise specified.

Table 3-1 Primary efficacy endpoint, supportive endpoints & analyses

**Endpoint**	**Analysis method**	
Aggregate ARR (confirmed relapses only) **(i.e., the primary efficacy variable)**	Negative binomial model (treatment as main effect, number of relapses in previous year, and baseline EDSS, baseline Gd + T1 lesion count as covariates) (region will be considered)	**Primary analysis**
	Negative binomial model (treatment)	Supportive analysis 1
	Negative binomial model (treatment as main effect, number of relapses in previous year, and baseline EDSS, baseline Gd + T1 lesion count as covariates) (region will be considered) on the full analysis set excluding subjects from site # 5147	Supportive analysis 2
	Negative binomial model (treatment as main effect, number of relapses in previous year, and baseline EDSS, baseline Gd + T1 lesion count as covariates) (region will be considered) on the per-protocol set	Supportive analysis 3
Aggregate ARR (confirmed and unconfirmed relapses)	Negative binomial model (treatment as main effect, number of relapses in previous year, and baseline EDSS, baseline Gd + T1 lesion count as covariates) (region will be considered)	Supportive analysis 4
Subject-level ARR (with imputation; confirmed relapses only)	Rank ANCOVA (treatment as main effect, number of relapses in previous year, and baseline EDSS, baseline Gd + T1 lesion count as covariates) (region will be considered)	Supportive analysis 5
Aggregate ARR (confirmed relapses only and time in study truncated at 367 days)	Negative binomial model (treatment as main effect, number of relapses in previous year, and baseline EDSS, baseline Gd + T1 lesion count as covariates) (region will be considered)	Supportive analysis 6
Aggregate ARR	Negative binomial model (treatment	Supportive analysis 7

(confirmed relapses derived by EDSS)	as main effect, number of relapses in previous year, and baseline EDSS, baseline Gd + T1 lesion count as covariates) (region will be considered)	

1) For the primary endpoint, a supportive analysis will be performed using a negative binomial regression model adjusted for treatment group only.

2) For the primary endpoint, a supportive analysis will be performed using the same negative binomial model as it is in the primary analysis but based on the full analysis set excluding all subjects from the site # 5147. Subjects in site #5147 were mismanaged by the primary investigator at this site.

3) For the primary endpoint, a supportive analysis will be performed using the same negative binomial model as it is in the primary analysis but on the per-protocol set. Relapses that start after study drug discontinuation will be excluded and log (time on study drug in years) rather than log (time in study in years) will be used as the offset variable in the negative binomial model with log link. The number of days on study drug is calculated as (the last study drug dose date – 1st study drug dose date +1) which will be used. This is an efficacy analysis of on-treatment data for subjects without major protocol deviations.

4) Analyses described in the primary analysis section will be repeated for the ARR counting all relapses (including both confirmed and unconfirmed relapses).

5) For the subject level ARR with imputation, a supportive analysis will be performed using Rank Analysis of Covariance (ANCOVA) with treatment group as main effect, number of relapses in the previous year before study enrollment, baseline EDSS, and baseline Gd + lesion count as covariates [Stokes (2000)]. An adjustment for country or region will be considered. For subjects who do not complete the treatment phase, relapses will be imputed (see Handling dropouts in ARR calculations Section). Additionally for subject-level ARR without imputation, summaries by treatment group will be presented.

6) Analyses described in the primary analysis sections will be repeated for the confirmed relapses but the calculation for time in study is different. For this supportive analysis the time in study is the number of days from the first study drug dose date to the study completion visit date or the last study drug dose date whichever occurs later. If more than 367 (=12*30+7) days is obtained, then it will be cutoff to be 367 days which corresponds to 12 months plus a 7-day window. If the study completion visit date is not available, the latest study visit date will be used instead. The time in study (years) will be calculated as the number of days in study/365.25. The confirmed relapses within this time period will be counted for each subject and will be used in the ARR computation.

7) Analyses described in the primary analysis sections will be repeated for the confirmed relapses where confirmation is derived based on EDSS/FS scores, not on the MS Relapses CRF. For each relapse reported on the MS Relapses CRF the confirmation status is derived as the following:

- If no EDSS is performed between start and end dates of the relapse, then the relapse is “unconfirmed” ;

- If an EDSS is performed between start and end dates of the relapse, then compare the EDSS/FS scores to the last available EDSS excluding EDSS recorded during the previous MS relapses. If there is an increase of 0.5 in EDSS or one point on two different FS or 2 points on one FS (all FS except bowel/bladder and Cerebral), the relapse is “confirmed”; otherwise it is “unconfirmed”.

4.2.1.3 Handling dropouts in ARR calculations

1) Negative binomial regression model for aggregate ARR on the full analysis set: For subjects who discontinue the study prematurely, the number of relapses recorded in the study will be used irrespective of whether the subject is on or off study drug and no imputation will be applied.

2) Negative binomial regression model for aggregate ARR on the per-protocol set: For subjects who discontinued the study drug prematurely, the number of relapses that start prior to or on the same day as the last dose date will be used and no imputation will be applied.

3) Rank ANCOVA model for subject-level ARR with imputation on the full analysis set:

For subjects who discontinue the study prematurely, the number of relapses per month for the months after discontinuation will be imputed by the corresponding monthly mean number of relapses of subjects across all treatment groups who has data of either 0 (no relapse) or 1 (one relapse) in that month. That is, for each month with completely missing data, the imputed number of relapses for the month is the average number of relapses for the corresponding month based on the available data from all subjects.

Subjects may have data from part of the month during which they drop out. The number of relapses for that month will be the observed number of confirmed relapse (either 0 or 1) during that partial month. That is, if a subject drops out with one relapse during the dropout month, the subject will be counted as having one relapse for the entire month. If no relapse is observed in the dropout month, the subject is assumed to have no relapse for the entire month. This is accurate because a subject could only have at most one confirmed relapse in each month due to the definition of confirmed relapse in the protocol (Appearance of a new neurological abnormality or worsening of previously stable or improving pre-existing neurological abnormality, separated by at least 30 days from onset of a preceding clinical demyelinating event).

Only the relapse start date will be used to determine the month in which a relapse will be counted when calculating the number of relapses per month.

The ARR of these subjects will be calculated based on the observed number of relapses before dropping out and the imputed number of relapses after dropping out. For example, suppose that the average number of relapses for each month based on all subjects contributing data to the month is as shown in Table 3-2.

Table 3-2 Example of imputation

Month	Average # of relapses
1	0.20
2	0.19

3	0.18
4	0.17
5	0.16
6	0.15
7	0.14
8	0.13
9	0.11
10	0.11
11	0.11
12	0.11

If a subject discontinues the study on Day 130 (i.e., after 4 months and 10 days) after having one confirmed relapse, his number of relapses from Month 6 on will be imputed from the above table. His total number of relapses will be the sum of 1, 0.15, 0.14, 0.13, 0.11...0.11, which equals to 1.86. Note that the imputed portion of the number of relapses will be the same for subjects who drop out in the same month.

The imputation will only apply to the dropout subjects up to 360 days (i.e., 12 months). After the imputation, the number of days in study for all dropout subjects is 360 days. For those who complete the study, the number of days in study will remain the same as defined in Time in study for aggregate ARR Section 4.1.3.

4) Summary of subject-level ARR without imputation on the full analysis set: For subjects who discontinue the study prematurely, the number of relapses recorded in the study will be used and no imputation will be applied.

4.2.1.4 Missing MS relapse dates

The following obvious corrections will be implemented by programming if the relapse partial start/or end date occurs where only day is missing.

For an incomplete relapse start date where day is missing but month and year are available, the day will be entered as the first day of the month. In case the relapse partial start date is in the same month as the first study drug dose date, the relapse start date will be entered as the first study drug dose date.

For an incomplete relapse end date where day is missing but month and year are available, the day will be entered as the last day of the month. In case the relapse partial end date is in the same month as the last visit date, the relapse end date will be entered as the last visit date. For a relapse is still ongoing at the last visit the relapse end date will be entered as the last visit date.

4.2.2 Analyses of secondary and exploratory variables

Secondary efficacy variables will be analyzed on the full analysis set unless otherwise specified. For most analyses, all three pair-wise treatment comparisons (fingolimod 0.5 mg vs. glatiramer acetate, 0.25 mg vs. glatiramer acetate, and 0.5 mg vs. 0.25 mg) will be provided even though no formal hypothesis will be tested between the 2 fingolimod doses.

4.2.2.1 Magnetic resonance imaging (MRI)

The analyses on MRI parameters will be performed on the full analysis set in the subset of subjects who have MRI scans done during the study. For all MRI data, no visit mapping will be performed for any visit and all analyses will be performed according to the visit recorded in the MRI data set.

The number and volume of Gd + lesion data obtained less than 30 days after use of steroid for treatment of relapses will be excluded from the analysis.

Analyses of MRI variables are summarized in Table 3-3 below and details will be provided in the following section.

Table 3-3 MRI endpoints & analyses

Endpoint	Analysis
Brain atrophy - Percent brain volume change (PBVC) from baseline at Month 12/end of treatment	Rank ANCOVA (treatment, age, baseline normalized brain volume (NBV), and the number of relapses experienced in the previous year before study enrollment) (region will be considered)
	ANCOVA (treatment, age, baseline normalized brain volume, and the number of relapses experienced in the previous year before study enrollment) (region will be considered)
	ANCOVA (treatment, age, baseline Gd + T1 lesion count, and baseline T2 lesion volume (in cc). (region will be considered)
New/newly enlarged T2 lesion count (compared with baseline MRI scan) at Month 12/end of treatment	Negative binomial (treatment, age, baseline T2 lesion count, baseline Gd + T1 lesion count, and the number of relapses experienced in the previous year before study enrollment) (region will be considered)
	Rank ANOVA (treatment, region)
Proportion of subjects free of new/newly-enlarged T2 lesions compared to baseline at Month 12/end of treatment	Logistic regression (treatment, age, baseline T2 lesion count, baseline Gd + T1 lesion count, and the number of relapses experienced in the previous year before study enrollment) (region will be considered)
Change (and % change) from Baseline in T2 lesion volume at Month 12/end of treatment	Rank ANCOVA (treatment, age, baseline T2 lesion volume, and the number of relapses experienced in the previous year before study enrollment) (region will be considered)
Gd + T1 lesion count at Month 12/end of treatment	Negative binomial (treatment, age, baseline T2 lesion count, baseline Gd + T1 lesion count, and the number of relapses experienced in the previous year before study enrollment) (region will be considered)
Proportion of subjects free of Gd + T1	Logistic regression (treatment, age, baseline Gd

lesions at baseline, Month 12/end of treatment	+ T1 lesion count, and the number of relapses experienced in the previous year before study enrollment) (region will be considered)
Gd + T1 lesion volume at Month 12/end of treatment	Rank ANCOVA (treatment, age, baseline Gd + T1 lesion volume, and the number of relapses experienced in the previous year before study enrollment) (region will be considered)
Proportion of subjects free of new T1 lesions compared to baseline at Month 12/end of treatment	Logistic regression (treatment, age, baseline T1 lesion count, baseline Gd + T1 lesion count, and the number of relapses experienced in the previous year before study enrollment) (region will be considered)
Change and % change from baseline in T1 lesion volume at Month 12/end of treatment	Rank ANCOVA (treatment, age, baseline T1 lesion volume, and the number of relapses experienced in the previous year before study enrollment) (region will be considered)
Percent brain volume change (PBVC) from baseline at Month 12/end of treatment<br>New/newly enlarged T2 lesion count at Month 12/end of study<br>Gd + T1 lesion count at Month 12/end of treatment<br>Proportion of subjects free of new/newly enlarged T2 lesions and proportion of subjects free of Td + T1 lesions at Month 12/end of treatment	**Sensitivity analyses** to repeat the same analyses descripted above, but exclude baseline assessments performed > 45 days before randomization and end of treatment assessments performed > 45 days after the last dose of study drug.
Annualized rate of brain atrophy (ARBA) at Month 12/end of treatment	**Sensitivity analyses** to adjust for study duration and MRI covariates:<br>Rank ANCOVA (treatment, age, baseline NBV, baseline Gd + T1 lesion count, and baseline T2 lesion volume, and the number of relapses experienced in the previous year before study enrollment) (region was considered)<br>ANCOVA (treatment, age, baseline NBV, baseline Gd + T1 lesion count, and baseline T2 lesion volume, and the number of relapses experienced in the previous year before study enrollment (region was considered)

The number of new or newly enlarging T2 lesions at Month 12/end of treatment and number of Gd-enhancing T1 lesions at Month 12/end of treatment will be analyzed using a negative binomial regression model with log link, using treatment as main effect, age, baseline T2 lesion count, baseline Gd-enhancing T1 lesion count, and the number of relapses experienced in the previous year before study enrollment as covariates. An adjustment for region will be considered in the model. For the analysis of T2 lesions, an offset variable will be used in the negative binomial analysis to adjust for the time in years (since start of study drug).

The proportion type variables will be analyzed using a logistic regression model with treatment as main effect, age, corresponding baseline value, baseline Gd-enhancing T1 lesion count, and the number of relapses experienced in the previous year before study enrollment as covariates. An adjustment for country or region will be considered in the model.

The continuous variables (change, percent change, and total volume) will be analyzed using a rank ANCOVA model with treatment as main effect, adjusted for age, corresponding baseline value, and the number of relapses experienced in the previous year before study enrollment as covariates. An adjustment for country or region will be considered. ANCOVA analysis for the percent change in brain volume with the above variables as covariates will be considered as well. In addition, an ANCOVA model with treatment, baseline Gd + T1 lesion count and the T2 volume (in cc) will be performed.

4.2.2.2 Relapses

All relapse-related analyses use confirmed relapses only, unless otherwise specified. Additional relapse-related variables including the following:

- Time to first relapse up to Month 12
- Proportion of relapse-free subjects up to Month 12
- Severity of the relapses and recovery status

Analyses of relapse-related variables are summarized in Table 3-4 below and details will be provided in the following section.

Table 3-4 Relapse-related endpoints & analyses

Endpoint	Analysis method
Time to first confirmed relapse up to Month 12	Log-rank test
	Cox's proportional hazard model (treatment, number of relapses in the previous year before study enrollment, baseline EDSS, baseline Gd + lesion count, and baseline T2 lesion volume) (region will be considered.)
Proportion of subjects free of relapse up to Month 12	Logistic regression model (treatment as main effect, number of relapses in previous year before study enrollment, baseline EDSS, and baseline Gd + lesion count as covariates) (region will be considered.)

Time to first relapse will be analyzed using a Cox proportional hazards model with treatment as main effect, number of relapses in the previous year before study enrollment, baseline EDSS, baseline Gd + T1 lesion count, and baseline T2 lesion volume as covariates. An adjustment for country or region will be considered in the specified model. The estimated hazard ratio with 95% confidence interval between treatment groups will be obtained. In addition, Kaplan-Meier curves by treatment will be used to present the time-dependent cumulative frequency and percentage of subjects reaching the time to first relapse; by-

treatment Kaplan-Meier estimates (with 95% confidence interval) at Month 12 will be obtained. Two-sided 95% confidence intervals of the difference in Kaplan-Meier estimates will also be presented. The log-rank test of the treatment difference in the Kaplan-Meier estimates of the events function will be performed.

Subjects without a confirmed relapse will be referred to as relapse-free subjects. Proportion of relapse-free subjects at Month 12 will be analyzed using a logistic regression model with treatment as main effect in the model, number of relapses in previous year before study enrollment, baseline EDSS, and baseline Gd + T1 lesion count as covariates. An adjustment for country or region will be considered. When calculating proportions, subjects who discontinue the study prematurely before having a confirmed relapse will be considered as having a confirmed relapse. Sensitivity analyses, however, will be performed by 1) excluding these subjects from the analysis and 2) considering these subjects (who are treated as having a confirmed relapse in the main analysis) as having no confirmed relapse.

The following relapse characteristics will be summarized at relapse level: severity (mild/moderate/severe), affecting daily activities (yes/no), steroid used (yes/no), hospitalization (yes/no), recovery status (none/partial/complete) and duration of relapse (days).

The following relapse characteristics will be summarized at subject level: severity, affecting daily activities, steroid used hospitalization, and recovery status. For summaries at subject level, a subject is counted only in the most severe category for each variable.

Summaries of the above mentioned relapse characteristics will be performed for confirmed relapses and for all relapses (confirmed and unconfirmed) as well.

4.2.2.3 Multiple Sclerosis Functional Composite Measure

There are 3 components to the MSFC: leg, arm, and cognitive function. The corresponding tests are 25-foot Timed Walking Test (25’TWT), 9-Hole Peg Test (9-HPT), and Paced Auditory Serial Addition Test 3” (PASAT3). Multiple trials of each test will be performed at each visit.

The MSFC z-scores are not collected on CRFs and thus need to be calculated. The instructions for deriving the subscale scores and the MSFC z-scores from the MSFC are provided below.

Firstly, the mean score is calculated for each component per subject per visit to obtain the subscale score.

- The average scores of the two 25’TWT trials:

  $$25'\mathrm{TWT}_{\mathrm{mean}} = \tfrac{1}{2}\left(25'\mathrm{TWT}_1 + 25'\mathrm{TWT}_2\right)$$

  In case of missing data, the average of the non-missing values will be taken.

- The average scores of the four 9-HPT trials (two for each hand):

  $$9HPT_{mean} = \tfrac{1}{4}(9HPT_{left1} + 9HPT_{left2} + 9HPT_{right1} + 9HPT_{right2})$$

  In case of missing data, the average of the non-missing values will be taken.

The above is used to derive the 9-HTP subscale score. In computing the z-score for 9-HTP, the mean 9-HTP will be computed by taking the average of 2 trials for each hand separately first and then taking the average of the reciprocals of the 2 averages, i.e.

$$9HPT_{mean} = \frac{1}{2}\left(\frac{1}{\frac{1}{2}(9HPT_{left1} + 9HPT_{left2})} + \frac{1}{\frac{1}{2}(9HPT_{right1} + 9HPT_{right2})} \right)$$

In case of missing data, the average of the non-missing values will be taken whenever an average needs to be computed.

- The number of correct answers from the PASAT 3, i.e.

$$PASAT3_{mean} = \#correct(PASAT3)$$

Secondly, a z-score is computed for each component. Z-scores are obtained by subtracting the mean and dividing by the standard deviation of a reference population. The baseline evaluations of all subjects where all treatment groups are pooled will be used as the reference population.

- The z-scores for the 25'TWT

$$25'\mathrm{TWT}_Z = \left(25'\mathrm{TWT}_{\mathrm{mean}} - 25'\mathrm{TWT}_{\mathrm{baseline-mean}}\right) / 25'\mathrm{TWT}_{\mathrm{baseline-stdev}}$$

- The z-scores for the 9-HPT

$$9\mathrm{HPT}_Z = \left(9\mathrm{HPT}_{\mathrm{mean}} - 9\mathrm{HPT}_{\mathrm{baseline-mean}}\right) / 9\mathrm{HPT}_{\mathrm{baseline-stdev}}$$

- The z-scores for the PASAT3

$$PASAT3_Z = \left(PASAT3_{\mathrm{mean}} - PASAT3_{\mathrm{baseline-mean}}\right) / PASAT3_{\mathrm{baseline-stdev}}$$

Finally, these individual z-scores are averaged to create the MSFC z-score:

$$MSFC_Z = \frac{1}{3}\left\{9HPT_Z - 25'TWT_Z + PASAT3_Z\right\}$$

The negative value of the 25'TWT z-score is taken to make the direction of change the same as the other components (i.e., a positive change indicates improvement in disability). In computing the MSFC z-score for each subject, if any of the individual z-scores ($9HPT_Z$, $25'TWT_Z$, or $PASAT3_Z$) are missing, the MSFC z-score will be missing.

Analyses of MSFC variables are summarized in Table 3-5 below and details will be provided in the following section. The full analysis set will be used unless otherwise specified.

Table 3-5 MSFC endpoints & analyses

Endpoint	Analysis
Change from baseline in MSFC (z-score, 25'TWT, 9-HPT, and PASAT3) by visit	Rank ANCOVA (treatment, region, corresponding baseline value, and age)

The MSFC z-score and the 3 subscale scores (25-foot timed walk, 9-hole peg test, PASAT3) and their change from baseline values will be summarized by visit. For the change from

baseline values at each visit, rank ANCOVA adjusted for treatment, corresponding baseline values, and age will be performed for treatment comparisons. An adjustment for region can be considered prior to database lock and will be defined in the analysis plan.

4.2.2.4 Symbol Digit Modality Test

The SDMT score and its change from baseline value will be summarized by visit. For the change from baseline values at each visit, rank ANCOVA adjusted for treatment, corresponding baseline values, and age will be performed for treatment comparisons. An adjustment for region can be considered prior to database lock and will be defined in the analysis plan.

4.2.2.5 EDSS Scores

Summary statistics will be presented on the FAS for the observed EDSS scores by visit and treatment. The change from baseline to end of study in EDSS score will be summarized on the FAS as well.

5 Pharmacokinetic/pharmacodynamics evaluations

All FTY720 subjects with quantifiable pharmacokinetic (PK) measurements will be included in the pharmacokinetic data analysis based on the FAS.

Biofluid concentrations will be expressed in mass per volume units. All concentrations below the limit of quantification or missing data will be labeled as such in the concentration data listings. In addition, sample number, concentration, sample date, sample time at pre-dose and minutes pre-dose will also be listed by treatment.

PK concentrations will be summarized by visit and treatment. In addition to mean, standard deviation (SD), median, min, max and quartiles, the geometric mean and coefficient of variation (CV) will also be presented. The formula for deriving the geometric mean and CV (%) as following:

- CV (%) = (SD/mean)*100,
- Geometric mean=exp(mean(log(x))).

Population PK/PD modeling approaches will be used to relate the individual fingolimod PK/PD parameters to key efficacy endpoints (e.g. relapse and number of new or enlarging T2 lesions). A modeling plan providing details for the proposed analysis will be prepared in a separate document by Novartis Modeling & Simulation group (M&S) before final clinical data lock and the corresponding PK/PD analysis will be performed by Novartis M&S group as well. The analysis will be reported separately from the CSR.

6 Regulatory immune-cells substudy analyses

All subjects in the substudy who have contributed at least the baseline and one post-baseline sample will be included in the analysis based on the FAS. The objective of the analysis is the description of the effect of treatment with fingolimod on the proportions of Th17 and Treg cells, as compared to baseline.

Flow cytometry assay will be used to measure the proportion of Treg and Th17. Table 3-6 below provides the complete list of T cell subsets with avialable results.

Table 3-6 List of T Cell Subsets Measured by Flow Cytometry

Flow Cytometry Panel	Parameter	T Cell Subset
A295 TReg (FoxP3/CD45RA) Assay	$CD3^+CD4^+CD25^+CD127^{-/lo}FoxP3^+$ (%CD4)	Treg Cell
	$CD3^+CD4^+CD25^+CD127^{-/lo}FoxP3^+CD45RA^+$ (%CD4)	CD45RA+ Treg Cell
	$CD3^+CD4^+CD25^+CD127^{-/lo}FoxP3^+CD45RA^-$ (%CD4)	CD45RA- Treg Cell
	$CD3^+CD4^+CD25^+CD127^{-/lo}$ (%CD4)	Surface Treg Cell
A219 Th17 (ic.IL-17) Assay	$CD3^+CD8^-CD4^+IL\text{-}17^+$ (%CD4)	Th17 Cell
A190 Th17 & CD45RA/ CD197 Assay	$CD3^+CD4^+CD45RA^-CD183^-CD194^+CD196^+$ (%CD4)	Surface Th17 Cell
	$CD3^+CD4^+CD45RA^+CD197^+$ (%CD4)	Naïve $CD4^+$ T Cell
	$CD3^+CD4^+CD45RA^-CD197^+$ (%CD4)	Central Memory $CD4^+$ T Cell
	$CD3^+CD4^+CD45RA^-CD197^-$ (%CD4)	Effector Memory $CD4^+$ T Cell
	$CD3^+CD8^+CD45RA^+CD197^+$ (%CD8)	Naïve $CD8^+$ T Cell
	$CD3^+CD8^+CD45RA^-CD197^+$ (%CD8)	Central Memory $CD8^+$ T Cell
	$CD3^+CD8^+CD45RA^-CD197^-$ (%CD8)	Effector Memory $CD8^+$ T Cell
	$CD3^+CD8^+CD45RA^+CD197^-$ (%CD8)	Effector $CD8^+$ T Cell

Due to the small sample size, the analyses for the sub-study will be purely descriptive without formal statistical test. For each T cell subset the assay results will be summarized by visit and treatment (including overall). The following summary statistics will be presented: number of observations, mean, standard deviation, standard error of mean, 95 confidence internal, minimum, first quartile, median, third quartile, interquartile range, and maximum. Percent change from baseline mean should be also reported.

Note: For most exploratory objectives specified in the protocol they will not be analyzed due to either limited data or samples collected.

7 Health-related Quality of Life (QoL)

7.1 PRIMUS-Activities

The Subject Reported Indices of Multiple Sclerosis **(**PRIMUS) instrument will be performed at Canada and US sites only. The PRIMUS - activities scale will be calculated and analyzed according to the scoring manual provided by Galen Research Limited, 2007.

The activities scale in this trial contains 15 items. Each of the 15 items will be given a score of 0 (able to do on own without difficulties) or 1 (able to do on own with difficulties) or 2

(unable to do on own). All 15 item scores will be summed to obtain a total score ranging from 0 (good) to 30 (poor), which is the PRIMUS activities scale score. Higher summary scores indicate worse health.

In case of missing item scores while calculating the scale score, the following rule applies: if more than 20% (i.e., three) of the item scores are missing, the scale score will not be calculated and set to missing. If no more than 20% of the item scores are missing, the scale score will be imputed as the average of the non-missing item scores multiplied by 15.

The PRIMUS activities scale score and its change from baseline will be summarized by visit. The change from baseline in PRIMUS activities scale score at each post-baseline visit will be compared between treatment groups using the Wilcoxon rank-sum test.

7.2 MSIS-29

The MS IMPACT SCALE (MSIS) in this trial contains 29 items with 5 possible outcomes for each item: 1 (Not at all), 2 (A little), 3 (Moderately), 4 (Quite a bit) and 5 (Extremely).

For the data listings, the item score will be used. For the summary of MSIS data, two summary scales: physical impact score (20 items) and psychological impact score (9 items) will be calculated according to the scoring manual provided by Hobart et al. in Improving the evaluation of therapeutic interventions in multiple sclerosis (originally published in 1995, updated in 2007). Higher summary scores indicate worse health. The detailed scoring algorithms are as following:

7.2.1 Physical impact score

The physical impact score is computed by summing items number 1-20 inclusive. This score can then be transformed to a score on a scale of 0 -100 using the formula below: transformed score = 100 x (observed score minus lowest possible score)/ (maximum possible score minus minimum possible score). For example, the MSIS-29 physical scale where min possible score = 20, max possible score = 100, range = (100 – 20) = 80 then physical impact score=100 x (observed score - 20) /(100 – 20). Note that transforming scores to have a range of 0 – 100 is for ease of interpretation. It doesn’t affect the properties of the scale.

7.2.2 Psychological impact score

The psychological score is computed by summing items number 21-29 inclusive. This score can then be transformed to a score on a scale of 0-100 using the formula below: 100 x (observed score - 9) / (45 – 9).

7.2.3 Missing data

For respondents with missing data, but where at least 50% of the items in a scale have been completed, a respondent–specific mean score computed from the completed items can be used for imputation. For example, consider person X who has completed 15 items in the physical scale. Sum the completed items and divide it by 15 to get person X’s respondent–specific mean score. Then use this value as the score for each of the missing 5 items. Then generate a total score as usual by summing the values of the 15 completed items and the 5 imputed items.

Note: respondents must have completed a minimum of 10 items in the physical scale, or 5 items in the psychological scale to use this imputing method.

The MSIS summary scores and the corresponding changes from baseline will be summarized by visit. The change from baseline in MSIS summary scores at each post-baseline visit will be compared between treatment groups using the ANCOVA analysis adjusting for age, region, and corresponding baseline variable.

7.3 TSQM (Version 1.4)

Treatment Satisfaction Questionnaire for Medication (TSQM) was developed and validated as a general measure for treatment satisfaction. The TSQM version 1.4 modified will be used, which contains 14 items assessing the following 4 domains: Global Satisfaction, Effectiveness, Side Effects, and Convenience. This patient reported outcome (PRO) must be completed prior to any other study visit assessments at the specified visits according to Table 6-1 assessment schedule in study protocol.

Scores for the TSQM version 1.4 will be generated using the authors' scoring algorithm. Missing data will be treated in accordance with the authors' guidelines (Atkinson et al 2004) as follows:

EFFECTIVENESS:

Score = {[(sum of items 1-3) – 3] / 18} * 100

If only one item is missing, Score = {[(sum of available items in 1-3) – 2] / 12} * 100

SIDE EFFECTS:

If Question 4 is answered "No" then Score = 100.

Otherwise:

Score = {[(sum of items 5-8) – 4] / 16} * 100

If only one item is missing, Score = {[(sum of available items in 5-8) – 3] / 12} * 100

CONVENIENCE:

Score = {[(sum of items 9-11) – 3] / 18} * 100

If only one item is missing, Score = {[(sum of available items in 9-11) – 2] / 12} * 100

GLOBAL SATISFACTION:

Score = {[(sum of items 12-14) – 3] / 14} * 100

If only one item is missing:

Item 12 or 13 missing: Score = {[(sum of available items in 12-14) – 2] / 10} * 100

Item 14 missing: Score = {[(sum of available items in 12-14) – 2] / 8} * 100

The higher summary scores indicate better satisfaction with study drug.

Scores for each scale at scheduled visits will be summarized by treatment group with n, mean SD, minimum, Q1, median, Q3, and maximum. The change from baseline score will be

compared within treatment group by Wilcoxon signed-rank test (also known simply as the Wilcoxon sign test).

8 Safety evaluation

Safety assessments include adverse events, infections, bradycardia events, laboratory tests, vital signs, ECG, pulmonary function tests (PFTs), ophthalmic examinations, and dermatology assessment.

All safety data will be summarized on the safety set. Some safety data (AEs, vital signs, labs, PFTs, and ophthalmic evaluations) will also be summarized on the follow-up set.

Unless otherwise specified, safety assessments more than 45 days after the study drug discontinuation will not be included in the summaries on the safety set but will be considered in the summaries on the follow-up set. The 45 days rule should be applied as follows:

45 days after the last study drug dose date will be the cutoff for safety data to be included in the summaries.

Adverse events starting/ending before the first dose date will be excluded from the adverse event summaries i.e. only treatment emergent AEs will be summarized by treatment.

Details of the safety data summaries will be provided in the sections below. All safety data will also be presented in the listings.

8.1 Adverse events

8.1.1 Adverse event date imputation

To ensure that all reported AEs are summarized, a conservative approach will be taken to impute the AE date when a partial date is available. AE date imputation is based on a comparison of the partial AE start date to the treatment start date (Table 4-1).

1. If the AE start date year value is missing, the AE start date will be imputed as the treatment start date.
2. If the AE start date year value is less than the treatment start date year value, the AE must have started before treatment. The following imputation rules will apply.
    a. If the AE month is missing, the AE start date will be imputed as the mid year date (01JulYYYY).
    b. If the AE month is not missing, the AE start date will be imputed as the mid month date (15MONYYYY).
3. If the AE start date year value is greater than the treatment start date year value, the AE must have started after treatment. The following imputation rules will apply.
    a. If the AE month is missing, the AE start date will be imputed as the year start date (01JanYYYY).
    b. If the AE month is not missing, the AE start date will be imputed as the month start date (01MONYYYY).

4. If the AE start date year value is equal to the treatment start date year value, the month values will be compared as follows.
    a. If the AE month is missing or the AE month is equal to the treatment start month, the AE start date will be imputed as the date one day after treatment start date.
    b. If the AE month is less than the treatment start month, the AE start date will be imputed as the mid month date (15MONYYYY).
    c. If the AE month is greater than the treatment start month, the AE start date will be imputed as the start month date (01MONYYYY).

Table 4-1 AE date imputation

MON MISSING	MON < CFM	MON = CFM	MON > CFM
(F)=TRTSTD<br>Uncertain	(F)=TRTSTD<br>Uncertain	(F)=TRTSTD<br>Uncertain	(F)=TRTSTD<br>Uncertain
(D) = 01JULYYYY<br>Before Treatment Start	(C)= 15MONYYYY<br>Before Treatment Start	(C)= 15MONYYYY<br>Before Treatment Start	(C)= 15MONYYYY<br>Before Treatment Start
(B)=<br>TRTSTD+1<br>Uncertain	(C)= 15MONYYYY<br>Before Treatment Start	(A)=<br>TRTSTD+1<br>Uncertain	(A)=<br>01MONYYYY<br>After Treatment Start
(E)=<br>01JANYYYY<br>After Treatment Start	(A)=<br>01MONYYYY<br>After Treatment Start	(A)=<br>01MONYYYY<br>After Treatment Start	(A)=<br>01MONYYYY<br>After Treatment Start

Before Treatment Start	Partial indicates date prior to Treatment Start Date
After Treatment Start	Partial indicates date after Treatment Start Date
Uncertain	Partial insufficient to determine relationship to Treatment Start Date

LEGEND:	
(A)	MAX(01MONYYYY,TRTSTD+1)
(B)	TRTSTD+1
(C)	15MONYYYY
(D)	01JULYYYY
(E)	01JANYYYY
(F)	TRTSTD

8.1.2 Adverse events

In this section, the term adverse event (AE) will refer to any entry on the adverse events CRFs. All AEs including infections will be coded using the MedDRA dictionary. The MedDRA version 20.0 or higher will be used depending when the study is completed.

Adverse events will be summarized by presenting, for each treatment group, the number and percentage of subjects having any AE by primary system organ class and preferred term. SAEs, death, drug-related AEs, the AEs leading to premature discontinuation of study drug, AEs requiring study-drug dose adjustment or interruption and AEs requiring additional therapy will be presented in a similar format as AEs. Notable events will include death, nonfatal SAEs (including infections), and AEs (including infections) leading to study drug discontinuation. AEs that fulfill the risk search terms (defined in the case retrieval sheet) with RMP risk will be summarized by risk name and lower level terms.

SAEs include death and non-fatal SAEs. All SAEs starting after the first dose date, including those starting more than 45 days after study drug discontinuation, will be included in the summaries on the safety set.

Additionally, occurrence rates of AEs, i.e. occurrence of AEs per 100 patient year by primary organ class and preferred term will be summarized. The occurrence rate of an event is calculated as the number (n) of all such events that patients experience divided by the patient years (Ny), where patient years are calculated as the sum of the number of days on study drug of all patients divided by 365.25. The occurrence of AEs per 100 patient years, which is calculated as n/Ny*100, will be presented.

AE summaries on the follow-up set will be conducted for the following time periods defined by days relative to study drug discontinuation: days 1 to 45 after study drug discontinuation, day 46 or later after study drug discontinuation.

8.1.2.1 Occurrence of SAEs and NSAEs (non-serious AEs)

For the legal requirements of ClinicalTrials.gov and EudraCT, two required tables on on-treatment adverse events which are not serious adverse events with an incidence greater than X% and on on-treatment serious adverse events and SAE suspected to be related to study treatment will be provided by system organ class and preferred term on the safety set population.

If for a same patient, several consecutive AEs (irrespective of study treatment causality, seriousness and severity) occurred with the same SOC and PT:

- a single occurrence will be counted if there is ≤ 1 day gap between the end date of the preceding AE and the start date of the consecutive AE
- more than one occurrence will be counted if there is > 1 day gap between the end date of the preceding AE and the start date of the consecutive AE

For occurrence, the presence of at least one SAE / SAE suspected to be related to study treatment / non SAE has to be checked in a block e.g., among AE's in a ≤ 1 day gap block, if at least one SAE is occurring, then one occurrence is calculated for that SAE.

The number of deaths resulting from SAEs suspected to be related to study treatment and SAEs irrespective of study treatment relationship will be provided by SOC and PT.

8.1.3 Data summaries for AEs

Summaries on the safety set by treatment group include:

- Incidence of AEs by SOC and PT
- Incidence of AEs that fulfill the risk search terms with RMP risk by risk name and level terms
- Incidence of AEs by SOC and PT and severity
- Incidence of deaths by SOC and PT
- Incidence of SAE by SOC and PT
- Incidence of SAEs that fulfill the risk search terms with RMP risk by risk name and level terms
- Incidence of AEs leading to study drug discontinuation by SOC and PT
- Incidence of AEs requiring study-drug dose adjustment or interruption by SOC and PT
- Incidence of AEs requiring additional therapy by SOC and PT
- Incidence of most frequent (>=1% for any group) AEs by PT
- Incidence of AEs related to study drug by SOC and PT
- Occurrence of AEs per 100 patient-years by SOC and PT
- Occurrence of SAEs by SOC and PT
- Occurrence of NSAEs by SOC and PT

Summaries on the follow-up set by treatment group include:

- Incidence of AEs by SOC and PT (days 1 – 45 after study drug discontinuation, and more than 45 days after study drug discontinuation)

8.2 Laboratory data

8.2.1 SI Units

Data summaries will be provided in SI units. The standard conversion tool (UNITCONV) will be used to convert all values in different units to SI units.

8.2.2 Clinically notable criteria

Clinically notable criteria are summarized in Table 4-2.

All applicable post-baseline laboratory results will be checked against the respective notable criteria. For a particular notable criterion, a subject will be counted in the notable abnormal category as long as one of the results meets the criterion. Note that a subject can be counted in both low and high abnormal categories.

For some laboratory parameters (as listed in Table 4-3), subjects may have a character value of “<x.x” or “<x” instead of the actual numeric value which are below the lower limit of detections (LLOD). The 50% of the LLOD rule will be applied to transform a character value into a numerical value as specified in Table 4-3. This rule will be applied to all applicable lab values prior to checking against the notable criteria as well as computation of any statistics.

For the laboratory parameters listed in Table 4-4, results will be summarized based on the abnormal criteria which are defined by pre-specified level of abnormality in terms of either LLN or ULN as listed in Table 4-4.

In addition, the patients meeting modified Hy's Law will be included in the summary of newly occurring or worsening post-baseline liver function test abnormalities. The modified Hy's Law biochemical criteria is defined as at least one of (ALT or AST > 3 x ULN) and (total bilirubin >=2 x ULN) and (alkaline phosphatase < 2 x ULN) at a same visit.

Table 4-2 Criteria for clinically notable abnormalities

**Laboratory Variable**	**Standard Units**	**SI Units**
**LIVER FUNCTION AND RELATED VARIABLES**		
SGOT (AST)	> 82 U/L	> 82 U/L
SGPT (ALT)	> 90 U/L	> 90 U/L
Gamma glutamyl transferase (GGT) (U/L)	> 130 U/L	> 130 U/L
Bilirubin (total)	≥ 2.0 mg/dL	≥ 34.2 μmol/L
Alkaline phosphatase, serum	> 280 U/L	> 280 U/L
**RENAL FUNCTION / METABOLIC AND ELECTROLYTE VARIABLES**		
Glucose	≥ 200 mg/dL	≥ 11.11 mmol/L
Creatinine	≥ 2.0 mg/dL	≥ 176 umol/L
Amylase	≥ 300 U/L	≥ 300 U/L
Cholesterol (total)	≥ 240 mg/dL	≥ 6.21 mmol/L
Triglycerides	≥ 300 mg/dL	≥ 3.39 mmol/L
BUN	≤ 2 mg/dL<br>≥ 30 mg/dL	≤ 0.7 mmol/L<br>≥ 10.7 mmol/L
Sodium	< 125 mEq/L<br>> 154 mEq/L	< 125 mmol/L<br>> 154 mmol/L
Chloride	≤ 85 mEq/L<br>≥ 119 mEq/L	≤ 85 mmol/L<br>≥ 119 mmol/L
Potassium	≤ 3.0 mEq/L<br>≥ 6.0 mEq/L	≤ 3.0 mmol/L<br>≥ 6.0 mmol/L
Magnesium	≤ 1.0 mg/dL<br>≥ 3.00 mg/dL	≤ 0.40 mmol/L<br>≥ 1.23 mmol/L
Calcium	≤ 7.5 mg/dL<br>≥ 11.6 mg/dL	≤ 1.87 mmol/L<br>≥ 2.89 mmol/L
Phosphate	≤ 2.0 mg/dL<br>≥ 5.3 mg/dL	≤ 0.65 mmol/L<br>≥ 1.71 mmol/L
**HEMATOLOGY VARIABLES**		
Haemoglobin	≤ 10.0 g/dL	≤ 100 g/L
Platelets (Thrombocytes)<br><br><labeled as Platelet count (direct) in database>	≤ 100 k/mm^3<br>≥ 600 k/mm^3	≤ 100 x 10^9/L<br>≥ 600 x 10^9/L
Leukocytes (WBCs)	≤ 2.0 k/mm^3	≤ 2.0 x 10^9/L

<labeled as WBC (total) in database>	≥ 15 k/mm^3	≥ 15 x 10^9/L
**HEMATOLOGY VARIABLES: DIFFERENTIAL**		
Granulocytes (Poly, Neutrophils)<br><br><labeled as Neutrophils (Seg. + Bands)>	≤ 1,000 /mm^3<br>≥ 12000/mm^3	≤ 1 x 10^9/L<br>≥ 12 x 10^9/L
Lymphocytes	< 200/mm^3<br>≥ 8000/mm^3	< 0.2 x 10^9/L<br>≥ 8 x 10^9/L
Red blood cells (RBCs)	< 3,300,000/mm^3<br>> 6,800,000/mm^3	< 3.3 x 10^{12}/L<br>> 6.8 x 10^{12}/L

*"Americas" refers to North (Canada, US), Central, and South America. It is applicable only to Canada and US in this study.

Table 4-3 The lower limit of detections of laboratory parameters

**Laboratory test**	**Character value**	**50% Lower limit of detection (LLOD)**
Absolute Monocytes	ABSMON < 0.1	ABSMON= 0.05 x 10^9/L
Absolute Lymphocytes	ABSLYM < 0.1	ABSLYM = 0.05 x 10^9/L
Alkaline Phosphatase	ALKPHS < 3	ALKPHS = 1.5 U/L
Amylase	AMY < 3	AMY= 1.5 U/L
SGOT (AST)	SGOT < 5	SGOT = 2.5 U/L
SGPT (ALT)	SGPT < 4	SGPT = 2 U/L
Gamma Glutamyltransferase (GGT)	GGT < 5	GGT = 2.5 U/L
Bilirubin (total)	TBIL < 2	TBIL = 1 μmol/L
Bilirubin (direct/conjugated)	DBIL < 3	DBIL = 1.5 μmol/L
HCG	HCG < 2	HCG = 1 IU/L
Herpes Simplex Virus 2 IgG	HSVIGG4 < 0.5	HSVIGG4= 0.25
IgG antibody to varicella zoster virus	IGGVZV2 < 10	IGGVZV2 =5 U/L
Glucose	GLUC < 1 mmol/L	GLUC= 0.5 mmol/L
Urine Protein Dipstick test	UPROTST < 0.1	UPROTST =0.05

Table 4-4 Abnormal criteria

**Laboratory Variable (unit)**	**Criteria**
Albumin (g/L)	<LLN
Bilirubin (direct/conjugated) (umol/L))	>ULN<br>>2xULN
Gamma Glutamyltransferase (GGT) (U/L)	>ULN<br>>3xULN
Glucose (mmol/L)	>ULN

Cholesterol (HDL) (mmol/L)	>ULN
Cholesterol (LDL) (mmol/L)	>ULN
SGOT (AST) (U/L)	>ULN<br>>2xULN<br>>3xULN<br>>5xULN
SGPT (ALT) (U/L)	>ULN<br>>2xULN<br>>3xULN<br>>5xULN
Cholesterol (total) (mmol/L)	>ULN

8.2.3 Data Summaries for lab tests

All lab parameters (excluding all hematology % values except % for white cell differential values) will be summarized.

Summaries on the safety set by treatment group include:

- Summary statistics of lab results (hematology, biochemistry, and urinalysis) by visit
- Changes from baseline in lab results (hematology, biochemistry, and urinalysis) by visit
- Summary statistics of percent of baseline in hematology results by visit
- Incidence rates of newly occurring or worsening abnormalities based on clinically notable laboratory abnormality criteria (based on criteria in Table 4-2)
- Incidence rates of clinically notable laboratory abnormalities by visit
- Incidence rates of laboratory results meeting abnormal criteria (in Table 4-4) by visit
- Frequency (%) distributions of liver function test results and hematology results in pre-defined categories (as specified in the table shells)
- Shift table (low, normal, or high as defined by the normal ranges) from baseline to post-baseline extreme values in hematology, biochemistry and continuous urinalysis results
- Shift table (negative, +, 2+, 3+, or 4+) from baseline to post-baseline extreme values in urinalysis results
- Frequency (%) distributions of serology results for subjects with serology test

Summaries on the follow-up set by treatment group include:

- Summary statistics of lab parameters (hematology, biochemistry, and urinalysis) by visit
- Change from baseline in lab parameters (hematology, biochemistry, and urinalysis) by visit

8.3 Vital signs

8.3.1 Vital signs measurements

The vital sign CRF collects systolic blood pressure (SBP) and diastolic blood pressures (DBP), pulse, body temperature, and body weight. The mean arterial blood pressure will be derived from the DBP and SBP using the formula below

- Mean arterial blood pressure = [2*DBP + SBP]/3.

Before the protocol amendment 1, the CRF collects vital signs only at sitting position at each visit. After the protocol amendment 1, the CRF collects vital signs at supine and standing position at each visit

For all visits (excluding the first or second or restart dose monitoring data) one measurement of pulse and 3 measurements of blood pressure (SBP and DBP) are collected before the protocol amendment 1 and three measurements of pulse and blood pressure (SBP and DBP) are collected.

At first or second or restart dose monitoring, pulse, SBP and DBP are measured 3 times at the scheduled pre-dose time point. The average of available values among three pulse (or SBP or DBP) measurements at the same position will be calculated.

For post-baseline assessments (excluding the first or second or restart dose monitoring data), the pulse or blood pressure value will be the average of the non-missing values of the 3 measurements, if it is not a single measurement, at the same position. If more than one blood pressure assessment after the average of the 3 measurements at the same position at the same date (scheduled or unscheduled, except for the first dose or second dose or restart dose monitoring data) exists in a particular visit as defined by the visit windows, the blood pressure value will be the average of all assessments at the same position.

The vital sign (pulse, SBP and DBP) during the first dose administration on Day 1 or Day 2 and restart data will not be put into visit windows, but will be summarized separately by the hour in which the measurement was reported.

8.3.2 Vital signs notable criteria

Clinical notable criteria in vital signs are summarized in Table 4-5.

Table 4-5 Criteria for clinically notable vital signs

Vital Sign Variable	Notable Criteria
Pulse (beats/min)	>120bpm or Increase of ≥15 bpm from baseline<br>or<br>< 50bpm or Decrease of ≥15 bpm from baseline
Systolic BP (mmHg)	≥160 mm Hg or Increase of ≥20 mm Hg from baseline<br>or<br>≤ 90 mm Hg or Decrease of ≥ 20 mm Hg from baseline
Diastolic BP (mmHg)	≥ 100 mmHg or Increase of ≥ 15 mm Hg from baseline<br>or<br>≤ 50 mmHg or Decrease of ≥ 15 mm Hg from baseline

Temperature (°C)	>38.3 °C/101°F
Body weight (kg)	± ≥ 7% from baseline weight

8.3.3 Data summaries for vital signs

Pulse and blood pressure will be reported separately for sitting, supine and standing position. Incidence rates of clinically notable, post-baseline abnormalities and orthostatic hypotension will be provided by treatment. Orthostatic hypotension is defined as a reduction of systolic blood pressure of at least 20 mm Hg or diastolic blood pressure of at least 10 mm Hg from supine to standing position.

The following summaries will exclude hourly vital sign data from the first dose or second dose or restart dose monitoring.

Summaries on the safety set by treatment group include:

- Summary statistics of vital sign parameters by visit
- Changes from baseline in vital sign parameters by visit
- Frequency (%) distributions of highest SBP, DBP, and lowest pulse in pre-defined categories respectively
- Incidence rates of notable vital sign abnormalities based on the notable criteria in Table 4-5
- Incidence rates of orthostatic hypotension by treatment and visit

Summaries on the follow-up set by treatment group include:

- Summary statistics of vital sign parameters by visit
- Change from baseline of vital sign parameters by visit

8.4 Electrocardiograms (ECG)

The ECG data includes quantitative variables such as ventricular rate, PQ or PR interval, R-R interval, QRS duration, and uncorrected QT interval as well as categorical variables such as ECG interpretation (normal or abnormal), ECG evaluation type (rhythm, conduction, etc) and ECG findings (prolonged QTc, low voltage, etc).

8.4.1 Corrected QT interval

The uncorrected QT interval will be corrected using the Bazett and Fridericia formulas. The Bazett formula corrects the QT interval by dividing by the square root of the R-R interval (secs). The Fridericia formula corrects the QT interval by dividing by the cubic root of the R-R interval (secs).

8.4.2 Abnormality criteria for corrected QT interval

Abnormality criteria for the corrected QT interval (Bazett and Fridericia) are listed in Table 4-6.

Table 4-6 Abnormality criteria for corrected QT interval

	Male Subjects	Female Subjects
1	>450 msec	>470 msec
2	>500 msec	>520 msec

3	30 - 60 msec increase from Baseline
4	>60 msec increase from Baseline

8.4.3 ECG findings

ECG findings are associated with the ECG evaluation type and thus will be summarized by the ECG evaluation type.

8.4.4 Data summaries for ECG data

The following summaries will exclude ECG data from the first dose or second dose or restart dose monitoring.

Summaries on the safety set by treatment group include:

- Summary statistics of quantitative ECG parameters by visit
- Changes from baseline in quantitative ECG parameters by visit
- Incidence rates of abnormal corrected QT interval (Bazett and Fridericia) as defined by the abnormality criteria in Table 4-6
- Frequency (%) distribution of ECGs interpretation (normal/abnormal) by visit
- Frequency (%) distribution of ECG findings by ECG evaluation type by visit

Note: For this study the only post-baseline visit is Month 12/EOT (end of treatment). So, all summaries above will be present for Baseline and then Month 12/EOT.

8.5 Pulmonary function tests (PFT)

8.5.1 Predicted values and units of PFTs

For each subject, the predicted values of the PFT parameters (Forced expiratory volume in 1 second (FEV_1), Forced vital capacity (FVC), FEV_1/FVC, and Diffusion capacity of carbon monoxide (D_LCO)) will be calculated based on the formulas below.

Reference equations to calculate the predicted values of FEV_1, FVC, FEV_1/FVC

- Forced expiratory volume in one second (FEV_1) (Unit: Liter/second):
  male: (4.3 · height (m)) – (0.029 · age (yr)) – 2.49 [±0.51]
  female: (3.95 · height (m)) - (0.025 · age (yr)) – 2.6 [±0.38]
- Forced vital capacity (FVC) (Unit: Liter):
  male: (5.76 · height (m)) – (0.026 · age (yr)) – 4.34 [±0.61]
  female: (4.43 · height (m)) – (0.026 · age (yr)) – 2.89 [± 0.43]
- FEV_1/FVC (Unit: %):
  male: - 0.18 · age (yr) + 87.21 [± 7.17]
  female: 0.19 · age (yr) + 89.10 [± 6.51]

Reference equations to obtain predicted values of D_LCO

- Single breath diffusion capacity (D_LCO) (Unit: ml/min/mmHg):
  Male: (0.3319 · height (cm)) – (0.1971 · age (yr)) – 18.006
  Female: (0.2441 · height (cm)) – (0.1436 · age (yr)) – 8.20

In the above calculations, the height of a subject is from the screening visit and the age should be calculated based on the date when he or she performs the PFTs.

For D_LCO, three units may be collected in the CRFs, which are ml/min/torr, ml/min/mmHg, and mmol/min/kpa. The PFT tables and listings will use the unit ml/min/mmHg. The other two units will be converted to ml/min/mmHg, prior to any calculations, as follows:

- 1 ml/min/torr = 1 ml/min/mmHg;
- 1 mmol/min/kpa = 2.986 ml/min/mmHg.

8.5.2 Percent predicted PFTs

The percent predicted PFT value, for each subject, is defined as (absolute PFT value / predicted PFT value) * 100.

The change from baseline in percent predicted PFT value is defined as (post-baseline percent predicted PFT value - baseline percent predicted PFT value).

The percent of baseline in absolute value of a PFT is calculated as (post-baseline absolute PFT value / baseline absolute PFT value) * 100.

The percent predicted PFT value will be categorized by pre-defined intervals. Details will be specified in the table shell document (RAP Module 7.1)

Relevant PFT changes are defined as below 80% of baseline and below 60% of baseline in absolute PFT values at 2 consecutive visits.

For non by-visit summaries, visits defined by visit windows will not be used. All available data from scheduled and unscheduled visits will be considered when checking 2 consecutive visits.

8.5.3 Data summaries for PFTs

Summaries on the safety set by treatment group include:

- Summary statistics in absolute values of FEV_1, FVC, FEV_1/FVC, and D_LCO and in percent predicted values of FEV_1, FVC, FEV_1/FVC, and D_LCO by visit
- Changes from baseline in absolute values of FEV1, FVC, FEV1/FVC, and D_LCO and in percent predicted values of FEV_1, FVC, FEV_1/FVC, and D_LCO by visit
- Number (%) of subjects meeting the following criteria as compared to the baseline (separately for FEV_1, FVC, D_LCO): below 80% at any visit; below 80% at 2 consecutive visits; below 60% at any visit; and below 60% at 2 consecutive visits
- Shift tables from baseline to the lowest post-baseline values in pre-defined categories of the percent predicted values of FEV_1, FVC, and D_LCO
- Number (%) of subjects with relevant PFT changes (for FEV_1, FVC, and D_LCO) by baseline percent predicted PFT categories

Summaries on the follow-up set include:

- Summary statistics in absolute values of FEV_1, FVC, FEV_1/FVC, and D_LCO and in percent predicted values of FEV_1, FVC, FEV_1/FVC, and D_LCO by visit
- Change from baseline in absolute values of FEV_1, FVC, FEV_1/FVC, and D_LCO and in percent predicted values of FEV_1, FVC, FEV_1/FVC, and D_LCO by visit

Note: Site 1004 had 5 subjects with unrealistic values (>80L) reported for FEV1/FVC in the CRF. The site is not responsive. On August 22nd, 2018, the study team reviewed the issue and agreed to set them as missing (unknown) in the analysis.

8.6 Ophthalmic evaluations

The ophthalmic evaluation includes assessment of visual acuity, assessment of optical coherence tomography (OCT), fluorescein angiography, and assessment of macular edema.

For the assessment of visual acuity, if the decimal score is not available but the Corrected Snellen equivalent is available, the decimal score will be calculated by division (e.g., Corrected Snellen equivalent = 10/20 infers that decimal score = 0.5). All decimal scores will be converted to the LogMAR (log of the minimum angle of resolution) equivalent by taking the negative of the common logarithm (i.e., $-\log_{10}$ (decimal acuity score)) (Holladay 1997). Summaries of the visual acuity will be performed on the converted scores (i.e., LogMAR equivalent).

If two or more visual acuity values for the same eye of a subject are available at a post-baseline visit window, the worst of all values will be used in the summaries. Note that the worst value for visual acuity refers to the smallest value in the decimal score (or equivalently refers to the largest value in the logMAR scale).

Visual acuity and change from baseline in visual acuity will be categorized by pre-defined intervals. Details will be specified in the table shell document (RAP Module 7.1).

The central foveal thickness (CFT), macular volume data from OCT and fluorescein angiography data will be displayed in listings.

8.6.1 Data summaries for ophthalmic evaluations

Summaries on the safety set by treatment group include:

- Summary statistics of visual acuity by visit
- Changes from baseline in visual acuity by visit
- Frequency (%) distribution of categorized visual acuity by visit
- Frequency (%) distribution of categorized change from baseline in visual acuity by visit
- Summary of diagnosis of macular edema

8.7 Dermatology assessment

Dermatology examinations will be performed at screening, and Month 12/end of study visits according to a protocol. Results of the dermatology exams are categorical (normal or abnormal with abnormal types) and will be summarized by treatment group for each visit as well as for the unscheduled visit. For unscheduled visit summary, if a subject has 2 or more unscheduled assessments, only the worst result will be summarized.

One shift table from baseline assessment to Month 12/end of treatment assessment will also be presented.

8.8 First dose (second dose or restart dose) of fingolimod monitoring

8.8.1 Vital signs

Hourly vital signs including pulse, SBP and DBP are collected at pre-dose and 6 hours after the first dose, second dose (if necessary), and restarting of dose after interruption (if applicable). The resting pulse and its change from pre-dose and percent change from pre-dose will also be categorized by pre-specified cutoffs as given in the table shells. The bradycardia events, bradycardia symptoms, and bradycardia treatment during the first dose, second dose (if necessary), and restarting of dose after interruption (if applicable) monitoring will be collected and summarized as well.

The bradycardia events, bradycardia symptoms, and bradycardia treatment will be collected on the corresponding CRFs. Note: Bradycardia events that do not have symptoms or require treatment are not collected in those CRFs and they are recorded in the Adverse Event CRF.

8.8.2 ECG

ECG will be performed at pre-dose and 6 hours post dose during the first dose, second dose (if necessary), and restarting of dose after interruption (if applicable) monitoring.

8.8.3 Dose monitoring experience

The overall dose monitoring experience refers to the following information: whether subjects are discharged after 6 hours post-dose or extended monitoring is required after 6 hours post-dose, whether subjects are hospitalized, whether subjects are required monitoring on the next day, whether subjects have symptomatic and/or treated bradycardia, whether subjects discontinue the study drug permanently, whether SAE is reported, and whether subjects are discharged but returned to clinic.

8.8.4 Data summaries for first dose monitoring

Summaries on the safety set by treatment group include:

- Summary statistics of vital signs by hour (1st dose, 2nd dose, and restarts)
- Changes from pre-dose in vital signs by hour (1st dose, 2nd dose, and restarts)
- Frequency (%) distribution of categorized supine and standing pulse or change (or percent change) from pre-dose supine and standing pulse during first dose administration, respectively
- Summary of the overall dose monitoring experience (1st dose, 2nd dose, and restarts)
- Incidence rates of bradycardia events (1st dose and 2nd dose)
- Incidence rates of medications used for bradycardia (1st dose and 2nd dose)
- Incidence rates of bradycardia symptoms by SOC, PT, and severity (1st and 2nd dose)
- Incidence rates of notable vital sign abnormalities based on the notable criteria in Table 4-5 (1st dose and 2nd dose)
- Summary statistics of ECG parameters by time point during 1st and 2nd dose administration

- Changes from pre-dose in ECG parameters by time point during 1st and 2nd dose administration
- Incidence rates of abnormal QTc interval (Bazett and Fridericia) as defined by the abnormality criteria in Table 4-6 during 1st and 2nd dose administration
- Frequency (%) distribution of ECGs interpretation (normal/abnormal) by time point during 1st and 2nd dose administration
- Frequency (%) distribution of ECG findings by ECG evaluation type by time point during 1st and 2nd dose administration

8.9 Columbia Suicide Severity Rating Scale (C-SSRS)

General

The Columbia Suicide Severity Rating Scale (C-SSRS) is a questionnaire that prospectively assesses suicidal ideation and behavior (SIB). There are two versions of the questionnaire:

- The "baseline/screening" version, which is to be performed at the first visit (Visit 1). It assesses SIB during the subject's lifetime.
- The "since last visit" version to be used at subsequent visits (Visit 2 to Visit 7), assessing SIB since last visit.

A copy of the questionnaires can be found in protocol amendment Appendix or CRF package.

The 11 categories described in Table 7-1 have been adopted as standard by FDA to report SIB ([Food and Drug Administration, 2012], [Nilsson et al, 2013]). These categories include five levels of suicidal ideation, five levels of suicidal behavior and the category self-injurious behavior, no suicidal intent. Each category has a binary response (yes/no). The C-SSRS questionnaire allows suicidal ideation and behavior to be classified into these 11 preferred categories.

Note: the categories in Table 7-1 have been reordered (based on [Nilsson et al 2013]) compared to the categories in the actual C-SSRS (paper) scale and the categories listed in the FDA guidance, in order to facilitate the definitions of endpoints and enable clarity in the presentation of results.

Table 7-1 Standard SIB events

Category number	C-SSRS category
**Suicidal Ideation**	
1	Wish to be dead
2	Non-specific active suicidal thoughts
3	Active suicidal ideation with any methods (not plan) without intent to act
4	Active suicidal ideation with some intent to act, without specific plan
5	Active suicidal ideation with specific plan and intent
**Suicidal behavior**	
6	Preparatory acts or behavior
7	Aborted attempt
8	Interrupted attempt

Category number	C-SSRS category
9	Actual attempt
10	Completed suicide
**Self-injurious behavior, without suicidal intent**	
11	Non-suicidal self-injurious behavior

In this study, the C-SSRS questionnaire will be administered electronically (eC-SSRS), via an interactive voice response (IVR) system with the patient entering responses directly into the IVRS. Caregivers will not be allowed to answer the C-SSRS questions on behalf of the patient. Sites must review reports received from the system for any answers indicative of suicidal ideation and adverse event AEs. Adverse events ascertained through the administration of the C-SSRS will be documented. No safety cut-off will be applied for the SIB data reporting, i.e. all collected data will be used in the analysis including data collected off-treatment, if any.

Definition of 'all prior history'

All prior history will be defined as the SIB results obtained from the *lifetime* assessment at the first visit (Visit 1).

Worst case is defined by an answer 'yes' to the SIB category.

Data summaries at the study level

SIB data will be summarized for the Safety Set. The number and percentage of subjects with suicidal ideation, suicidal behavior and self-injurious behavior without suicidal intent will be presented by analysis-period (all prior history and any time post-baseline) and treatment group. The following 14 events will be included in the summary table:

- Each of the 11 categories listed in Table 7-1, separately
- Any suicidal ideation or behavior (a 'yes' answer to at least one of the 10 suicidal ideation and behavior questions in analysis-period of interest)
- Any suicidal ideation (answered 'yes' to at least one of the 5 suicidal ideation questions in analysis-period of interest)
- Any suicidal behavior (answered 'yes' to at least one of the 5 suicidal behavior questions in analysis-period of interest).

For those analyses, each subject can only be counted once for each event. However, a subject can be counted in several different events.

Suicidal ideation and behavior data will be listed. Detailed answers to C-SSRS items will be listed separately for subjects with any suicidal ideation at any time post-baseline (i.e. a 'yes' answer to at least one of the five suicidal ideation questions at any time post-baseline) and for subjects with any suicidal behavior at any time post-baseline (i.e. a 'yes' answer to at least one of the five suicidal behavior questions at any time post-baseline).

9 Interim analyses

No efficacy interim analyses are planned. On an as-needed basis, safety interim analysis for the DSMB will be performed by an independent statistician and independent statistical programmer. The analysis plan for these safety interim analyses will be provided in a separate document.

10 Determination of sample size

The study will randomize a total of 1960 patients, and is planned to provide approximately 90% power for the comparison of fingolimod 0.5 mg to glatiramer acetate at a 2-sided significance level of 0.05.

The sample size calculation is based on simulations from a negative binomial distribution with a constant dispersion parameter k.

The power of the study was evaluated under various ARR assumptions and various dropout patterns based on the cumulative literature on glatiramer acetate and the Novartis data on fingolimod. The basis of the assumptions for this study and their level of uncertainty are presented in Section 9.4.2 under the sub-header “Multiplicity adjustment for statistical hypothesis testing”. The anticipated overdispersion parameter (k=0.2231) was observed in the Month-12 analysis of Study CFTY720D2301. The anticipated ARR for subjects treated with 0.5 mg fingolimod is $\mu_{\text{FTY 0.5 mg}} = 0.195$, the ARR for those treated with glatiramer acetate is $\mu_{\text{glatiramer acetate}} = 0.30$. Therefore, the estimated ARR reduction for fingolimod 0.5 mg versus glatiramer acetate is 35%.

The total sample size of 1960 randomized is predetermined but the exact sample size of each arm will depend on when the randomization ratio switch occurs. For example, if the randomization ratio is switched when a total of 800 patients have been randomized from an original ratio of 1:1:1 for fingolimod 0.25 mg, fingolimod 0.5 mg, or glatiramer acetate, respectively to a ratio of 5:3:2, then 847 patients will be randomized to fingolimod 0.25 mg, 615 patients to fingolimod 0.5 mg and 498 patients glatiramer acetate, which will provide more than 90% power to demonstrate superiority of fingolimod 0.5 mg dose versus glatiramer acetate in terms of ARR at a 2-sided significance level of 0.05 assuming a 15% drop-out rate. The calculations take into account that patients who discontinue prematurely from the study can participate with partial data to the primary endpoint.

Fingolimod 0.25 mg has never been studied in a clinical trial in MS. Based on PK/PD modeling results, it is anticipated that the ARR in subjects treated with fingolimod 0.25 mg is approximately 15% higher than in those treated with fingolimod 0.5 mg. However, the uncertainty of this estimate is high; the 95% confidence interval of the estimated ARR in fingolimod 0.25 mg group ranges from 0.18 to 0.30. It is therefore anticipated that fingolimod 0.25 mg is less efficacious than fingolimod 0.5 mg, but more efficacious than glatiramer acetate. In line with the PK/PD modeling the anticipated ARR in subjects treated with fingolimod 0.25 mg is $\mu_{\text{FTY 0.25 mg}} = 0.225$, which corresponds to a reduction in ARR of 25% in subjects treated with fingolimod 0.25 mg compared to those treated with glatiramer acetate.

Following the multiplicity adjustment procedure in Multiplicity adjustment section 4.2.1.1, the power to detect a 25% reduction in ARR for subjects treated with fingolimod 0.25 mg

compared with subjects treated with glatiramer acetate is approximately 68% at a 2-sided significance level of 0.05, if the primary objective for the fingolimod 0.5 mg dose can be rejected first; the corresponding marginal power with multiplicity adjustment is approximately 61%. If fingolimod 0.25 mg is similarly efficacious as fingolimod 0.5 mg (i.e., ARR reduction versus glatiramer acetate is 35% rather than 25%), the power to detect this treatment effect at a 2-sided significance level of 0.05 is approximately 88%. If fingolimod 0.25 mg is similarly efficacious as glatiramer acetate (i.e., estimated ARR in the fingolimod 0.25 mg group is at the high end of range proposed by the PK/PD model), there will be no significant difference at a 2-sided significance level of 0.05.

Due to the study early termination approved in December 2016 the final number of randomized patients is 1064 with 370 for fingolimod 0.25 mg, 352 for fingolimod 0.5 mg and 342 for glatiramer acetate. The statistical power based on the final sample size is approximately 71% for comparison of fingolimod 0.5 mg vs glactiramer acetate. For the comparison of fingolimod 0.25 vs glactiramer acetate the statistical power is approximately 44% without multiplicity adjustment and 31% with multiplicity adjustment.

The statistical software R (Version 2.13.1, open source) and the R library packages “MASS” and “PSCL” were used for sample size calculations and power analysis.

11 Documentation of changes from the protocol

For immune-cell sub-study analyses the following secondary and exploratory objectives will not be performed to limited data or samples collected:

Secondary objectives:

- :To evaluate the effect of chronic fingolimod 0.5 mg and 0.25 mg treatment on the ratio of CD4+ and CD8+ T-cells

Exploratory objectives

- To explore the effect of chronic fingolimod 0.5 mg and 0.25 mg treatment on the total number of CD4+ and CD8+ T-cells
- To assess the relationship between T cell subsets stratified based on CD45RA and CCR7 expression and relative to CD4 lymphocyte counts
- To assess the relationship between PK measures and immune subset pharmacodynamics (PD) measures noted above
- To examine chronic fingolimod treatment effects on expression of selected mRNA molecules that are (i) involved in Th17 and Treg differentiation, (ii) implicated as abnormal in MS immune cells, and (iii) implicated as potential markers of response to therapy
- To measure serum levels of molecules that reflect the balance between pro- andanti-inflammatory immune responses, particularly Th17 and Treg responses (possiblyincluding IL-1b, IL-2, IL-6, IL-7, IL-10, IL-15, IL-22, and TGF-β), and molecules that may reflect CNS injury (possibly including neurofilament light chain and heavy chain) and repair (such as brain-derived neutrotrophic factor)
- To explore the effect of chronic fingolimod 0.5 and 0.25 mg treatment on B cellproportions and relationship to absolute lymphocyte counts

12 Clinical Study Report - Appendix 16.1.9 Documentation of statistical methods

12.1 Statistical methods and analysis outputs

12.1.1 Statistical methods

Statistical Analysis System (SAS) version 9.2 or higher will be used to perform all the statistical analyses in the report.

Pair-wise treatment comparisons will be performed using separated data for treatment groups (A vs. C, B vs. C, and A vs. B) for the following analyses: rank ANCOVA/ANOVA, survival analyses, Wilcoxon rank-sum test, and Fisher's exact test. Data from all treatment groups (A, B, and C) will be used for the following analyses: negative binomial regression, logistic regression, and ANCOVA.

12.1.1.1 Negative binomial regression model

The primary efficacy variable (aggregate ARR) will be analyzed using a negative binomial regression model. This analysis will be performed on the full analysis set and the per-protocol set. All 3 treatment groups will be used to fit the regression model in the analysis. In addition, the negative binomial regression model will be used for several supportive analyses for aggregate ARR and number of new or newly enlarged T2 lesions.

Negative binomial regression is used for modeling count variables, usually for overdispersed count outcome variables, while adjusting for one or more covariates. In general, Negative binomial regression analyzes the distributed data in the following form

$$Y_i \sim NB(r_i, p_i),\ for \quad i = 1, \cdots, n.$$

where the **negative binomial distribution** is a discrete probability distribution of the number of successes in a sequence of Bernoulli trials before a specified (non-random) number of failures (denoted *r*) occur with a probability p.

The log of the count variables are modeled as a linear function of the X_i.

$$\log(y_i) = \beta_0 + \beta_1 x_{1,i} + \cdots + \beta_k x_{k,i}$$

Below is an example of the SAS codes used to perform the analysis of the negative binomial regression. The independent variables and covariates in the model may change per analysis defined in this document.

```
**************************************************************************
* SAS Codes: Negative binomial regression model
* Variables in the model:
* relnum = number of relapses
* trt = treatment group code
* reg1a = region code
* n2 = number of relapses in previous year
* bledss = baseline EDSS
* sid1a = subject ID
```

```
* lnday = log(days in study/365.25)
***************************************************************************;
proc genmod data=data1;
     class trt reg1a;
     model relnum=trt reg1a n2 bledss / dist=nb
                                        link=log
                                        offset=lnday maxiter=500 lognb;
     lsmeans trt /cl;
     estimate 'A – B' trt  1  -1  0/exp;
     estimate 'A – C' trt  1   0 -1/exp;
     estimate 'B – C' trt  0   1 -1/exp;

     ods output estimates=est;
     ods output lsmeans=lst;
run;
```

12.1.1.2 Rank Analysis of Covariance (ANCOVA) and Rank Analysis of Variance (ANOVA)

Rank Analysis of Covariance (ANCOVA)

The supportive efficacy variable, subject-level ARR (confirmed relapse only), will be analyzed using the rank ANCOVA. Other secondary efficacy variables, subject-level ARR (confirmed and unconfirmed relapse), change from baseline in MSFC z-score and subscales, and MRI variables, will be analyzed similarly on the full analysis set.

The rank ANCOVA is a non-parametric statistical method described in Stokes, Davis, and Koch (2000). It can be considered as an extension to the Wilcoxon rank-sum test with the ability to adjust for covariates in the model.

The analysis can be easily implemented using SAS by the following three steps:

1) compute the ranks of the response variable and covariate in the combined group of treatment using PROC RANK;

2) calculate the residuals from the linear regression of the response variable ranks vs. ranks of the covariates (without treatment) using PROC REG;

3) use the CMH mean score statistics to compare the mean values of the residuals in treatment by using TABLE scores (default scores) in PROC FREQ.

Below is an example of the SAS codes used to perform this rank ANCOVA. The independent variables and covariates in the model may change per analysis defined in this document.

```
***************************************************************************
* SAS Codes: Rank ANCOVA model
* Variables in the model:
* relann = subject-level annualized relapse rates
* trt = treatment group code
* reg1a = region code
* n2 = number of relapses in previous year
* bledss = baseline EDSS
```

```
* other covariates as well
* sid1a = subject ID
****************************************************************************;
Data eff;
     Set eff;
     If reg1a=' ' or n2=. or bledss=. or relann=.then delete;
run;

proc rank data=eff nplus1 ties=mean out=ranks;
     by reg1a;
     var n2 bledss relann more convariates…;
run;

proc reg data=ranks noprint;
     by reg1a;
     model relann=n2 bledss more convariates…;
     output out=residual r=resid;
run;

proc freq data=residual;
     tables reg1a*trt*resid / noprint cmh2;
     where trt in ('A','B');
     output out=rancova cmh2;
     *** SELECT P_CMHRMS;
run;
```

Rank Analysis of Variance (ANOVA)

Number of new or newly-enlarged T2 lesions will be analyzed using the rank ANOVA adjusted for treatment and region on the full analysis set. Unlike Rank ANCOVA where continuous covariates exist, the PROC RANK and PROC REG steps are not necessary. The van Elteren's test will be used for treatment comparisons. The van Elteren's test is a rank based test and can be implemented in PROC FREQ with options CMH2 and score equal to MODRIDIT. The van Elteren's test statistic corresponds to the row mean scores statistic labeled "Row Mean Scores Differ" in the CMH output.

Below is an example of the SAS codes used to perform this rank ANOVA. The independent variables and covariates in the model may change per analysis defined in this document.

```
****************************************************************************
* SAS Codes: Rank ANOVA model
* Variables in the model:
* RSLVAL1N = number of new or newly-enlarged T2
* trt = treatment group code
* reg1a = region code
* sid1a = subject ID
****************************************************************************;
proc freq data= eff;
     where trt in ('A','B');
     tables reg1a*trt*RSLVAL1N / noprint cmh2 scores=MODRIDIT;
```

```
     output out=ranova cmh2;
     *** SELECT P_CMHRMS;
run;
```

12.1.1.3 Logistic regression for proportion variables

The other secondary/other efficacy variables (proportion of subjects free of relapses, proportion of subjects free of new or newly enlarging T2 lesions, proportion of subjects free of Gd + lesions, proportion of subjects free of new T1 lesion) will be analyzed using the logistic regression on the full analysis set. All 3 treatment groups will be used in the model.

Logistic regression is a model for prediction of the probability of occurrence of an event. It can be used to analyze the dichotomous response data while adjusting for one or more covariates. Usually, Logistic regression analyzes binomially distributed data of form

$Y_i \sim B(n_i, p_i),\ for \quad i = 1, \cdots, n.$

The logits of the unknown binomial probabilities (*i.e.*, the logarithms of the odds) are modeled as a linear function of the X_i.

$$\log\left(\frac{p_i}{1-p_i}\right) = \beta_0 + \beta_1 x_{1,i} + \cdots + \beta_k x_{k,i}.$$

Below is an example of the SAS codes used to perform the analysis of the LOGISTIC regression. The independent variables and covariates in the model may change per analysis defined in this document.

```
***************************************************************************
* SAS Codes: LOGISTIC model
* Variables in the model:
* mrifree = proportion of subjects free of Gd + lesions
* reg1a = region code
* blmri = baseline number of Gd + lesions
* more covariates could be added as needed
***************************************************************************;
proc logistic data=eff descending;
     class reg1a trt /param=ref;
     model mrifree = reg1a trt blmri (more covariated as needed);

     contrast ' A - B '  trt  1 -1  0/estimate=exp;
     contrast ' A - C '  trt  1  0 -1/estimate=exp;
     contrast ' B - C '  trt  0  1 -1/estimate=exp;

     ods output Contrastestimate=est;
run;
```

For this study, proc logistic is performed including all 3 treatment levels with the contrast specifying 2 levels.

12.1.1.4 Survival analysis for time to event variables

Log-rank test will be used to compare the survival distributions of two treatment groups for secondary efficacy time to event variables, for example time to first confirmed relapse. These analyses will be performed on the full analysis set.

The logrank test is a hypothesis test to compare the survival distributions of two samples. It is a nonparametric test and appropriate to use when the data are right skewed and censored (technically, the censoring must be non-informative). It is constructed by computing the observed and expected number of events in one of the groups at each observed event time and then adding these to obtain an overall summary across all time points where there is an event.

Cox proportional hazards model is to estimate the effect parameter(s) without any consideration of the hazard function assuming the proportional hazards assumption holds. The proportional hazards assumption is the assumption that effect parameters multiply hazard: for example, if taking drug X halves your hazard at time 0, it also halves your hazard at time 1, or time 0.5, or time t for any value of t. The effect parameter(s) estimated by any proportional hazards model can be reported as hazard ratios.

Below is an example of the SAS codes used to perform the Log-rank test with Kaplan-Meier Method and the Cox proportional hazards model. The independent variables and covariates in the model may change per analysis defined in this document.

```
**************************************************************************
* SAS Codes: LOG-RANK test with Kaplan-Meier Method
* Variables in the model:
* reltm = time to first confirmed relapse
* trt = treatment group code
* relcens = censor flag (1=censored, 0=not censored)
**************************************************************************;
proc lifetest data=eff method=km;
     where trt in ('A','B');
     time reltm*relcens(1);
     strata trt;
run;
**************************************************************************
* SAS Codes: Cox's regression model
* Variables in the model:
* reltm = time to first confirmed relapse
* trt = treatment group code
* reg1a = region code
* n2 = number of relapses in previous year
* bledss = baseline EDSS
* relcens = censor flag (1=censored, 0=not censored)
* more covariates could be added as needed
**************************************************************************;
```

```
***include all 3 treatment arms data in one model fitting***
proc phreg data=datain nosummary;
  class trt reg1a
  model reltm*relcens(1)=trt n2 bledss reg1a (more covariates as needed)/rl
ties=exact;
  contrast "A vs C" trt 1 0 -1/estimate=exp;
  contrast "B vs C" trt 0 1 -1/estimate=exp;
  contrast "A vs B" trt 1 -1 0/estimate=exp;
  ods output parameterestimates=pest;
  ods output contrastestimate=est;
run;
**Note that data set pest contains Hazard ratio estimates from model
statement where only A vs C and B vs C are available. If parameter='trt',
then SELECT probchisq hazardratio hrlowercl hruppercl for results;
**However, data set est contains Hazard ratio estimates from contrast
statement where all 3 pairwise comparisons are available (result variable
names: estimate lowerlimit upperlimit probchisq);
```

12.1.1.4.1 Estimation of 95% confidence intervals

Approximate 95% confidence intervals will be generated for several estimates: (1) at selected time-points, the (Kaplan and Meier 1958) estimates of the proportion of subjects experiencing a specific event, (2) at selected time-points, the between-treatment group difference in Kaplan-Meier estimates of the proportion of subjects experiencing a specific event, (3) for certain study periods, the between-treatment group difference in the proportion of subjects experiencing a specific event, and (4) at selected time-points, the between-treatment group difference in means. Details of such confidence intervals are displayed in Table 5-1.

Table 12-1 Confidence Interval calculations

	**Approximate Confidence Interval (CI)**	**Lower Limit**	**Upper Limit**
1	x% CI for the Kaplan-Meier estimate KM	$KM - c_x{*}SE$	$KM + c_x{*}SE$
	Notation: SE = estimated standard error of KM;<br>estimation of standard error uses Greenwood's formula,<br>c_x = $\Phi^{-1}(½ + x/200)$, where Φ is the normal distribution function.		
2	x% CI for the difference in Kaplan-Meier estimates $KM_2\text{-}KM_1$	$KM_2\text{-}KM_1 - c_x{*}SE_d$	$KM_2\text{-}KM_1 + c_x{*}SE_d$
	Notation: KM_i = Kaplan-Meier estimate for group i = 1,2,<br>$SE_d = \sqrt{(SE_1^2 + SE_2^2)}$, where SE_i^2 = estimated standard error of KM_i, i = 1,2;<br>estimation of standard error uses Greenwood's formula,<br>c_x = $\Phi^{-1}(½ + x/200)$, where Φ is the normal distribution function.		
Examples: for x = 95, $c_x = \Phi^{-1}(0.975) = 1.95996$; for x = 97.5, $c_x = \Phi^{-1}(0.9875) = 2.24140$			

12.1.1.5 Between-treatment group comparisons for other efficacy variables

Generally, non-stratified, non-parametrical tests will be used to perform between-treatment group comparisons on MS confirmed relapse characteristics and change from baseline in disability, MRI variables (Gd + T1 lesion count, T2 lesion volume, Gd + lesion volume, T1 hypointense lesion volume, and changes from baseline in T2 lesion volume and Gd + lesion volume, percentage change from baseline in brain volume). Tests applied will be the rank ANCOVA. For a small number of comparisons, Wilcoxon rank-sum test for continuous variables will be utilized; Fisher's exact test will be used for categorical variables; for variables with more than five categories it will be replaced with the chi-square test. Wilcoxon

rank-sum test is also used for comparison between the treatment groups in change from baseline in PRIMUS activities scale at post-baseline visits.

Below is an example of the SAS codes used to perform these analyses.

```
*****************************************************************************
* SAS Codes: Wilcoxon rank-sum test
* Variables in the model:
* c_msact = change from baseline in PRIMUS activities scale
* trt = treatment group code
*****************************************************************************;
proc npar1way data=eff wilcoxon noprint;
     where trt in ('A','B');
     class trt;
     var C_MSACT;
run;
*****************************************************************************
* SAS Codes: Fisher's exact test
* Variables in the model:
* pevt = 1-subject with confirmed relapses;
         0-subject without confirmed relapses;
* trt = treatment group code
*****************************************************************************;
proc freq data=eff noprint;
     where trt in ('A','B');
     tables trt*pevt / exact;
run;
* Select p-value for normal approximation from Wilcoxon Two-Sample Test
```

12.1.1.6 Analysis of Covariance (ANCOVA) for quality of life (QoL) variables

The quality of life variables: change from baseline in MSIS physical impact score and psychological impact score converted from MSIS items will be analyzed on the full analysis set by visit. The ANCOVA model includes treatment as the main effect, covariates of region, baseline responding QoL score and age. Below is an example of the SAS codes used to perform the analysis. All 3 treatment groups will be used in the model.

```
*****************************************************************************
* SAS Codes: ANCOVA model
* Variables in the model:
* c_msphs = change from baseline in MSIS physical impact score
* trt = treatment group code
* reg1a = region code
* age_1n = age at baseline
* blMSPHYS = baseline MSIS physical impact score
*****************************************************************************;
```

```
proc mixed data=input dataset;
     class reg1a trt;
     model c_msphys = reg1a trt age_1n blmsphys;
     lsmeans  trt /pdiff cl;
run;
```

One sample t-test for quality of life (QoL) variables

The regular one-sample t-test will be used to perform within-group comparisons of continuous (or quantitative) efficacy variables where the normality assumption is held reasonably well. The within-group analyses for continuous / quantitative variables will compare assessments for the end of treatment vs. baseline within each treatment group. Examples of such analysis include the within group comparisons of the quality of life variable: change from baseline in TSQM total score.

Below is an example of the SAS codes used to perform the analysis. All 3 treatment groups will be used in the model.

```
*************************************************************************
* * SAS Codes: One sample t-test
* V1 = First timepoint --- e.g.:(baseline);
* V2 = Second timepoint --- e.g.:(post-baseline visit, end of treatment);
* PID = Subject ID or number of observations;
* diff= difference between two timepoints (higher scores indicate better
results);
* NOTE: First, prepare and extract the data for each treatment group.
* Then, in each treatment group, break up the data into the 2 timepoints.
* Ensure that each subject has a value at both timepoints (V1 and V2).
*************************************************************************;
data one;
   input PID V1 V2;
   diff = V2 - V1;

proc ttest data = one sides=u;
   by treat;
   var = diff;
run;
```

12.1.1.7 Wilcoxon sign test for quality of life (QoL) variables

Wilcoxon signed-rank test (also known simply as the Wilcoxon sign test) can be thought of as the non-parametric equivalent of the regular one-sample t-test. It will be used to perform all within-group comparisons of continuous (or quantitative) efficacy variables. The within-group analyses for continuous / quantitative variables will compare assessments for the end of treatment vs. baseline within each treatment group. Examples of such analysis include the within group comparisons of the quality of life variable: change from baseline in TSQM total score.

Below is an example of the SAS codes used to perform the analysis. All 3 treatment groups will be used in the model.

```
*************************************************************************
* * SAS Codes: Wilcoxon signed-rank test (or Wilcoxon sign test)
* V1 = First timepoint --- e.g.:(baseline);
* V2 = Second timepoint --- e.g.:(post-baseline visit, end of treatment);
* PID = Subject ID or number of observations;
* diff= difference between two timepoints;
* NOTE: First, prepare and extract the data for each treatment group.
* Then, in each treatment group, break up the data into the 2 timepoints.
* Ensure that each subject has a value at both timepoints (V1 and V2).
*************************************************************************;
data one;
   input PID V1 V2;
   diff = V2 - V1;

proc univariate data = one;
   by treat;
   var = diff;
run;
```

12.1.2 Statistical analysis outputs

Results from the primary and secondary efficacy analyses will be presented in Section 16.1.9 of the clinical study report.

The list of outputs that will be generated is provided in RAP Module 7.1.

13 References

Atkinson MJ, Sinha A, Hass SL et al. Validation of a general measure of treatment satisfaction, the Treatment Satisfaction Questionnaire (TSQM) using a national panel study of chronic disease. Health and Quality of Life Outcomes 2004; 2:12

Bretz, F, Maurer, W, Brannath, W et al (2009) A graphical approach to sequentially rejective multiple test procedures Stat Med 28(4), 586–604.

Doward LC, McKenna SP, Meads DM, et al. (2009) The development of patient reported outcomes indices for multiple sclerosis (PRIMUS) Multiple Sclerosis 15: 1092-1102.

FDA (2012) Guidance for industry. Suicidal Ideation and Behavior: Prospective Assessment of Occurrence in Clinical Trials (last draft August 2012, available under http://www.fda.gov/downloads/Drugs/Guidances/UCM225130.pdf)

Gassmann-Mayer et al (2011) Clinical and statistical assessment of suicidal ideation and behavior in pharmaceutical trials. *Clin Pharmacol Ther. 2011 Oct;90(4):554-60.*

Hobart J, Lamping D, Fitzpatrick R, et al. (2001) The Multiple Sclerosis Impact Scale (MSIS-29) A new patient-based outcome measure. Brain 124: 962-973

Kaplan, EL, Meier, P. (1958) Nonparametric estimation from incomplete observations. J. Amer. Statist. Assn. 53: 457-481.

Keene ON, Jones MRK, Lane PW, Anderson J., et al. (2007) Analysis of exacerbation rates in asthma and chronic obstructive pulmonary disease: example from the TRISTAN study. Pharmaceutical Statistics 6: 89-97.

Nilsson M. et al (2013) Columbia-Suicide Severity Rating Scale. Scoring and data analysis guide. (available at http://www.cssrs.columbia.edu/C-SSRS%20Scoring%20and%20Data%20Analysis%20Guide_Cross-Industry%20Working%20Group_v2.0Final.pdf)

Stokes ME, Davis CS, Koch GG (2000). Categorical data analysis using the SAS system. Second edition. SAS Institute, Inc., Cary, N.C.

Twiss J, Doward LC, McKenna SP, Eckert B, (2010) Interpreting scores on multiple sclerosis-specific patient reported outcome measures (the PRIMUS and U-FIS). Health and Quality of Life Outcomes 8:117.

14 Appendices

14.1 Appendix 1: Geographic location (state/providence and country) of the study centers

Refer to clinical document CFTY720D2312 Global Qualified Sites list for study centers and their geographic location information. Below is the list of study centers with at least one randomized subject and also their region classification.

Center Number	Country	State/Province	Region
0101	Argentina	CABA	Latin American region
0106	Argentina	CABA	Latin American region
0301	Brazil	MG	Latin American region
0302	Brazil	RS	Latin American region
0303	Brazil	SC	Latin American region
0304	Brazil	RJ	Latin American region
0305	Brazil	RS	Latin American region
0307	Brazil	GO	Latin American region
0309	Brazil	SP	Latin American region
0310	Brazil	PR	Latin American region
0403	CANADA	Nova Scotia	Northeast region
0404	CANADA	Ontario	Northeast region
0405	CANADA	Alberta	West region
0407	CANADA	Quebec	Northeast region

Center Number	Country	State/Province	Region
0408	CANADA	Quebec	Northeast region
0411	CANADA	Quebec	Northeast region
0413	CANADA	British Columbia	West region
0501	Chile	Santiago	Latin American region
0502	Chile	Santiago	Latin American region
1001	Mexico	Chihuahua	Latin American region
1002	Mexico	DF	Latin American region
1004	Mexico	Chihuahua	Latin American region
1005	Mexico	Nvo Leon	Latin American region
1008	Mexico	Nvo Leon	Latin American region
1009	Mexico	Aguascalientes	Latin American region
1010	Mexico	San Luis Potosi	Latin American region
1012	Mexico	DF	Latin American region
5002	UNITED STATES	MI	Midwest region
5006	UNITED STATES	IN	Midwest region
5007	UNITED STATES	FL	Southeast region
5009	UNITED STATES	OH	Midwest region
5010	UNITED STATES	FL	Southeast region
5012	UNITED STATES	AZ	West region
5014	UNITED STATES	GA	Southeast region
5017	UNITED STATES	NJ	Northeast region
5018	UNITED STATES	FL	Southeast region
5020	UNITED STATES	TX	Southwest region
5024	UNITED STATES	MI	Midwest region
5025	UNITED STATES	CA	West region
5026	UNITED STATES	OH	Midwest region
5027	UNITED STATES	CO	Southwest region
5030	UNITED STATES	OK	Southwest region
5031	UNITED STATES	FL	Southeast region
5037	UNITED STATES	NM	Southwest region
5039	UNITED STATES	TX	Southwest region
5040	UNITED STATES	IL	Midwest region
5043	UNITED STATES	WA	West region
5045	UNITED STATES	NJ	Northeast region
5046	UNITED STATES	NY	Northeast region
5047	UNITED STATES	UT	West region

Center Number	Country	State/Province	Region
5048	UNITED STATES	KS	Southwest region
5052	UNITED STATES	TN	Southeast region
5053	UNITED STATES	TX	Southwest region
5054	UNITED STATES	MI	Midwest region
5057	UNITED STATES	TN	Southeast region
5059	UNITED STATES	KS	Southwest region
5061	UNITED STATES	VA	Southeast region
5063	UNITED STATES	OH	Midwest region
5065	UNITED STATES	IL	Midwest region
5068	UNITED STATES	CO	Southwest region
5069	UNITED STATES	NY	Northeast region
5070	UNITED STATES	AZ	West region
5071	UNITED STATES	NC	Southeast region
5072	UNITED STATES	OH	Midwest region
5074	UNITED STATES	AZ	West region
5075	UNITED STATES	FL	Southeast region
5076	UNITED STATES	TN	Southeast region
5078	UNITED STATES	DE	Northeast region
5080	UNITED STATES	FL	Southeast region
5082	UNITED STATES	NY	Northeast region
5083	UNITED STATES	CA	West region
5084	UNITED STATES	NY	Northeast region
5085	UNITED STATES	VA	Southeast region
5086	UNITED STATES	CO	Southwest region
5089	UNITED STATES	NY	Northeast region
5090	UNITED STATES	OK	Southwest region
5092	UNITED STATES	TX	Southwest region
5094	UNITED STATES	DC	Northeast region
5096	UNITED STATES	FL	Southeast region
5097	UNITED STATES	IL	Midwest region
5098	UNITED STATES	CO	Southwest region
5100	UNITED STATES	FL	Southeast region
5101	UNITED STATES	MO	Southwest region
5102	UNITED STATES	NJ	Northeast region
5105	UNITED STATES	MO	Southwest region
5106	UNITED STATES	VA	Southeast region

Center Number	Country	State/Province	Region
5108	UNITED STATES	AL	Southeast region
5109	UNITED STATES	IN	Midwest region
5111	UNITED STATES	FL	Southeast region
5112	UNITED STATES	NY	Northeast region
5113	UNITED STATES	NC	Southeast region
5119	UNITED STATES	IA	Midwest region
5120	UNITED STATES	IL	Midwest region
5121	UNITED STATES	NV	West region
5122	UNITED STATES	OK	Southwest region
5123	UNITED STATES	MO	Southwest region
5124	UNITED STATES	OH	Midwest region
5125	UNITED STATES	MA	Northeast region
5126	UNITED STATES	MA	Northeast region
5127	UNITED STATES	KY	Midwest region
5128	UNITED STATES	OH	Midwest region
5131	UNITED STATES	CO	Southwest region
5132	UNITED STATES	PA	Northeast region
5133	UNITED STATES	FL	Southeast region
5134	UNITED STATES	TX	Southwest region
5136	UNITED STATES	MO	Southwest region
5137	UNITED STATES	CT	Northeast region
5138	UNITED STATES	NY	Northeast region
5140	UNITED STATES	TN	Southeast region
5142	UNITED STATES	AZ	West region
5143	UNITED STATES	WA	West region
5146	UNITED STATES	VA	Southeast region
5147	UNITED STATES	MI	Midwest region
5152	UNITED STATES	TX	Southwest region
5157	UNITED STATES	FL	Southeast region
5158	UNITED STATES	LA	Southeast region
5159	UNITED STATES	SC	Southeast region
5160	UNITED STATES	NC	Southeast region
5161	UNITED STATES	MI	Midwest region
5162	UNITED STATES	WA	West region
5163	UNITED STATES	MT	West region
5164	UNITED STATES	PR	Southeast region

Center Number	Country	State/Province	Region
5165	UNITED STATES	OR	West region
5169	UNITED STATES	WI	Midwest region
5170	UNITED STATES	FL	Southeast region
5171	UNITED STATES	VA	Southeast region
5175	UNITED STATES	OH	Midwest region
