# Supplementary material for: Efficacy and Safety of 2 Fingolimod Doses vs Glatiramer Acetate for the Treatment of Patients With Relapsing-Remitting Multiple Sclerosis: A Randomized Clinical Trial
Source: JAMA Neurol. 2020 Aug 24;78(1):1–13. doi: 10.1001/jamaneurol.2020.2950 (PMC7445630; doi:10.1001/jamaneurol.2020.2950)
Supplement: Supplement 2. — eMethods. ASSESS Steering Committee, Exclusion Criteria, Definition of Confirmed Relapse, and Statistical Analyses for Secondary and Exploratory End Points eResults. MSFC z Score and Subscales, SDMT Scores, HR-QoL Outcomes, and Post Hoc Analysis of Selection Bias eTable 1. Exploratory End Points eTable 2. Relapse Outcomes eTable 3. Negative Binomial Regression of Confirmed Relapses Up to 12 Months eFigure. Study Design eReferences [file jamaneurol-e202950-s002.pdf]

Supplementary Online Content

Cree BAC, Goldman MD, Corboy JR; ASSESS Trial Investigators. Efficacy and safety of 2 fingolimod doses vs glatiramer acetate for the treatment of patients with relapsing-remitting multiple sclerosis: a randomized clinical trial. *JAMA Neurol.* Published August 24, 2020. doi:10.1001/jamaneurol.2020.2950

eMethods. ASSESS Steering Committee, Exclusion Criteria, Definition of Confirmed Relapse, and Statistical Analyses for Secondary and Exploratory End Points

eResults. MSFC *z* Score and Subscales, SDMT Scores, HR-QoL Outcomes, and Post Hoc Analysis of Selection Bias

eTable 1. Exploratory End Points

eTable 2. Relapse Outcomes

eTable 3. Negative Binomial Regression of Confirmed Relapses Up to 12 Months

eFigure. Study Design

eReferences

This supplementary material has been provided by the authors to give readers additional information about their work.

© 2020 Cree BAC et al. *JAMA Neurology*.

eMethods. ASSESS Steering Committee, Exclusion Criteria, Definition of Confirmed Relapse, and Statistical Analyses for Secondary and Exploratory End Points

ASSESS Steering Committee

- Bruce Cree (Head of the SC, principal investigator)
- Myla Goldman (Coordinating investigator)
- Jack Antel
- Douglas Arnold
- John Corboy
- Corey Ford
- Edward Fox
- Barry Singer

Exclusion Criteria

Patients with a history of malignancy other than cutaneous basal cell carcinoma in the last 5 years, active infection, diabetes mellitus, macular edema or clinically significant systemic disease, active chronic diseases of the immune system other than MS, severe hepatic injury or other hepatic conditions, certain electrocardiogram findings, or history of certain cardiovascular conditions within 6 months were excluded. Patients were also excluded if they received the following treatments prior to randomization: immunosuppressive/chemotherapeutic medications within 6 months; immunoglobulins within 4 weeks; natalizumab within 2 months; rituximab, alemtuzumab, ofatumumab, ocrelizumab, or cladribine within one year, or teriflunomide within 3.5 months. A washout period was not necessary for patients treated with dimethyl fumarate, interferon β, or GA.

The detailed exclusion criteria are mentioned below.

Subjects who fulfilled any of the following criteria were not eligible for inclusion in this study:

1. Subjects with a history of malignancy of any organ system (other than cutaneous basal cell carcinoma) in the last 5 years that did not have confirmation of absence of a malignancy prior to randomization.
2. Subjects with an active chronic disease (or stable but treated with immune therapy) of the immune system other than MS (e.g. rheumatoid arthritis, scleroderma, Sjogren's syndrome, Crohn's disease, ulcerative colitis) or with a known immunodeficiency syndrome (human immunodeficiency virus [HIV]-antibody positive, acquired immune deficiency syndrome [AIDS], hereditary immune deficiency, drug-induced immune deficiency).
3. Subjects who had been treated with:
   - IV immunoglobulin (Ig) within 4 weeks before randomization.
   - Immunosuppressive/chemotherapeutic medications (e.g. azathioprine, cyclophosphamide, methotrexate) within 6 months before randomization.
   - Natalizumab within 2 months before randomization.
   - Previous treatment with lymphocyte-depleting therapies (e.g. rituximab, alemtuzumab, ofatumumab, ocrelizumab, or cladribine) within 1 year before randomization.
   - Previous treatment with mitoxantrone within 6 months before randomization.
   - Use of teriflunomide within 3.5 months prior to randomization, except if active washout (with either cholestyramine or activated charcoal) was done. In that case, plasma levels were required to be measured and be below 0.02 mg/L before randomization.

© 2020 Cree BAC et al. *JAMA Neurology*.

No washout period was necessary for subjects treated with dimethyl fumarate, IFN beta, or GA. Subjects being treated with dimethyl fumarate, GA, or IFN beta at the Screening Visit were allowed to continue drug intake up to the day before Day 1 of the study.

4. Subjects who had been treated with systemic corticosteroids or adrenocorticotropic hormones in the past 30 days prior to the screening MRI procedure.
5. Subjects with uncontrolled diabetes mellitus (glycosylated hemoglobin [HbA1c] > 9%) or with diabetic neuropathy.
6. Subjects with a diagnosis of macular edema during screening (subjects with a history of macular edema were allowed to enter the study provided that they did not have macular edema at Screening).
7. Subjects with severe active bacterial, viral, or fungal infections.
8. Subjects without acceptable evidence of immunity to varicella zoster virus (VZV) at randomization.
9. Subjects who had received any live or live-attenuated vaccines (including for VZV, herpes simplex, or measles) within 1 month before randomization.
10. Subjects who had received total lymphoid irradiation or bone marrow transplantation.
11. Subjects with any unstable medical/psychiatric condition, as assessed by the primary treating physician at each site.
12. Subjects who in the last 6 months experienced any of the following cardiovascular conditions or findings in the screening electrocardiogram (ECG):
    - Myocardial infarction
    - Unstable angina
    - Stroke
    - Transient ischemic attack
    - Decompensated heart failure requiring hospitalization or Class III/IV heart failure.
13. Subjects with history or presence of a Mobitz Type II atrioventricular (AV) block, or a third-degree AV block or sick sinus syndrome, unless the subject had a functioning pacemaker.
14. Subjects with baseline QTc interval > 500 ms.
15. Subjects receiving Class Ia (e.g. ajmaline, disopyramide, procainamide, quinidine) or Class III antiarrhythmic drugs (e.g. amiodarone, bretylium, sotalol, ibutilide, azimilide, dofetilide).
16. Subjects with severe respiratory disease, pulmonary fibrosis, or Class III or IV chronic obstructive pulmonary disease, or with clinically significant lung pathology on chest x-ray. Subjects with controlled asthma were allowed to enter the study.
17. Subjects with any of the following hepatic conditions:
    - Severe hepatic injury (Child-Pugh Class C)
    - Total bilirubin greater than 2 times the upper limit of the reference range, unless in the context of Gilbert's syndrome
    - Conjugated bilirubin greater than 2 times the upper limit of the reference range
    - AST or ALT greater than 2 times the upper limit of the reference range
    - Alkaline phosphatase greater than 2 times the upper limit of the reference range
    - GGT greater than 2 times the upper limit of the reference range
18. Subjects with a screening WBC count < 3500/mm^3 or lymphocyte count < 800/mm^3.
19. Subjects with any of the following neurologic/psychiatric disorders:
    - History of substance abuse (drug or alcohol) in the past 5 years or any other factor (i.e. serious psychiatric condition) that may have interfered with the subject's ability to cooperate and comply with the study procedures
    - progressive psychiatric/neurological condition that may affect participation in the study
20. Subjects who had received an investigational drug or therapy within 180 days or 5 half-lives of randomization, whichever was longer.
21. Subjects with a history of hypersensitivity to any of the study drugs, to drugs of similar chemical classes, or to mannitol.

© 2020 Cree BAC et al. *JAMA Neurology*.

22. Pregnant or nursing (lactating) women, where pregnancy was defined as the state of a female after conception and until the termination of gestation, confirmed by a positive human chorionic gonadotropin laboratory test.
23. Women of childbearing potential, defined as all women physiologically capable of becoming pregnant, UNLESS they were using highly effective contraception during dosing with study treatment. Highly effective contraception included the following:
    - Total abstinence (when this is in line with the preferred and usual lifestyle of the subject). Periodic abstinence (e.g. calendar, ovulation, symptothermal, postovulation methods) and withdrawal are not acceptable methods of contraception.
    - Female sterilization (had surgical bilateral oophorectomy with or without hysterectomy) or tubal ligation at least 6 weeks before taking study treatment. In case of oophorectomy alone, only when the reproductive status of the woman had been confirmed by follow up hormone level assessment was she considered not of childbearing potential.
    - Male partner sterilization (at least 6 months prior to screening). For female subjects on the study, the vasectomized male partner was to have been the sole partner for that subject.
    - Use of oral, injected, or implanted hormonal methods of contraception or other forms of hormonal contraception that have comparable efficacy (failure rate < 1%), for example, hormone vaginal ring or transdermal hormone contraception, placement of an intrauterine device or intrauterine system.

    Women were considered postmenopausal and not of childbearing potential if they had 12 months of natural (spontaneous) amenorrhea with an appropriate clinical profile (e.g. age appropriate, history of vasomotor symptoms) or had surgical bilateral oophorectomy (with or without hysterectomy) or tubal ligation at least 6 weeks prior to Baseline. In the case of oophorectomy alone, only when the reproductive status of the woman was confirmed by follow up hormone level assessment was she considered not of childbearing potential.
24. Subjects who had previously been treated with GA discontinued treatment due to lack of efficacy or tolerability.
25. Subjects with a history of treatment with FTY720.
26. Subjects with a score of 4 or 5 on the Suicidal Ideation item of the Columbia-Suicide Severity Rating Scale (C-SSRS) within 2 years of Screening, or any "yes" on the Suicidal Behavior item of the C-SSRS at Screening.

Definition of Confirmed Relapse

A relapse was confirmed when it was accompanied by an increase of ≥0.5 on the EDSS or an increase of 1 point on 2 different functional systems (FSs) of the EDSS or 2 points on 1 of the FS (excluding bowel and bladder and cerebral FSs).

Statistical Analyses for Secondary and Exploratory End Points

The negative binomial regression model used for analyzing the new or new enlarging and Gd-enhancing T1 lesions used treatment as main effect, age, baseline T2 lesion count, baseline Gd-enhancing T1 lesion count, and the number of relapses experienced in the previous year before study enrollment as covariates. An adjustment for region was considered in the model.

The logistic regression model used for analyzing proportion type variables used treatment as main effect, age, corresponding baseline value, baseline Gd-enhancing T1 lesion count, and the number of relapses experienced in the previous year before study enrollment as covariates. An adjustment for country or region was considered in the model.

Total brain volume, change, percent change were analyzed using a rank ANCOVA model with treatment as main effect, adjusted for age, corresponding baseline value, and the number of relapses experienced in the previous year before study enrollment as covariates. An adjustment for country or region was considered. An ANCOVA analysis for the percent change in brain

© 2020 Cree BAC et al. *JAMA Neurology*.

volume with the above variables as covariates was considered as well. In addition, an ANCOVA model with treatment, baseline Gd-enhancing T1 lesion count, and the T2 volume (in cc) was used. The rank ANCOVA is a non-parametric statistical method described in Stokes, Davis, and Koch (2000)[1] which does not rely on distribution of data (e.g. normality and homogeneity of variance) and can be considered as an extension to the Wilcoxon rank-sum test with the ability to adjust for covariates in the model.

Time to first relapse was analyzed using a Cox proportional hazards model with treatment as main effect, number of relapses in the previous year before study enrollment, baseline EDSS, baseline Gd-enhancing T1 lesion count, and baseline T2 lesion volume as covariates.

Both MSFC z-score and SDMT scores and their change from baseline values were summarized by visit. Change from baseline values between the treatment groups were compared using rank ANCOVA adjusted for treatment, corresponding baseline values, and age.

For HRQoL analyses, no adjustment for multiplicity was performed. The change from baseline in the PRIMUS-Activities scale scores were compared between the treatment groups by Wilcoxon rank-sum test. The change from baseline in MSIS-29 summary scores were compared across treatment groups using ANCOVA, adjusted for age, region, and corresponding baseline variable. Scores for TSQM scales were summarized by treatment group and change from baseline scores within the treatment groups were compared by a Wilcoxon signed-rank test.

© 2020 Cree BAC et al. *JAMA Neurology*.

eResults. MSFC *z* Score and Subscales, SDMT Scores, HR-QoL Outcomes, and Post Hoc Analysis of Selection Bias

MSFC *z* Score and Subscales

Fingolimod 0.5 mg showed numerically greater improvements in MSFC z-score and its subscales compared with GA from baseline to Month 12. A significant improvement was observed in the PASAT subscale. Fingolimod 0.25 mg showed a numerical trend of better impact on 9-HPT and PASAT-3 subscales compared with GA (**eTable 1**).

SDMT Scores

Improvement in the SDMT scores was observed at Month 12 across treatment groups. However, the improvement observed with fingolimod doses was not significant compared with GA 20 mg (**eTable 1**).

HR-QoL Outcomes

- **PRIMUS-Activities**

  Fingolimod 0.5 mg significantly improved the daily activities score at Month 12 compared with GA. Fingolimod 0.25 gm group showed less deterioration in performing daily activities compared with GA, however, this improvement did not reach statistical significance (**eTable 1**).

- **MSIS-29 scale**

  A significant improvement was observed with fingolimod 0.5 mg versus GA 20 mg in both physical and psychological impact scale scores at Month 12, while fingolimod 0.25 mg showed a numerical trend of better effect on both subscales (**eTable 1**).

- **TSQM scale**

  Treatment satisfaction improved significantly from Baseline to Month 12 in all TSQM domains (global satisfaction, effectiveness, side effects) across treatment groups except for improvement in convenience domain with GA 20 mg. Improvement in treatment satisfaction was 2-fold higher in both fingolimod dose groups than in GA 20 mg group.

Post Hoc Analysis of Selection Bias

Due to slower than expected recruitment, many sites enrolled only a few participants (30% of sites have 3 or fewer participants). To assess whether the results could be driven by a particular subgroup several post-hoc analyses were explored. First, sites were grouped using six pre-defined geographic regions. A negative binomial regression model was developed in which treatment-by-region was included as a random effect. The results are similar to the fixed effects for the primary analysis, suggesting that the treatment effect can be generalized beyond the regions evaluated in the study (Table 3). Further by-Region subgroup analysis showed ARR and ARR ratios generally in keeping with the

© 2020 Cree BAC et al. *JAMA Neurology*.

results for the overall population. Additional analyses using baseline age, sex, and presence of gadolinium-DPTA enhancing lesions yielded similar findings.

© 2020 Cree BAC et al. *JAMA Neurology*.

eTable 1. Exploratory End Points

Change from baseline to Month 12/ end of treatment	Fingolimod 0.5 mg N=345	Fingolimod 0.25 mg N=366	GA 20 mg N=324
**MSFC z-score**			
**n**	287	313	274
**Mean (SD)**	0.09 (0.6)	0.03 (0.6)	0.03 (0.6)
p value[a] vs. GA 20 mg	*0.05*	*0.52*	–
**25-foot timed walking test**			
**n**	306	325	286
**Mean (SD)**	–0.45 (9.4)	0.91 (11.7)	–0.37 (9.8)
p value[a] vs GA 20 mg	*0.13*	*0.96*	–
**9-hole peg test**			
**n**	308	327	286
**mean (SD)**	-0.80 (25.3)	–0.31 (20.2)	0.29 (18.0)
p value[a] vs GA 20 mg	*0.17*	*0.23*	–
**Paced auditory serial addition test 3**			
**n**	291	317	276
**mean (SD)**	2.4 (9.5)	1.7 (10.0)	1.1 (8.0)
p value[a] vs. GA 20 mg	***0.01****	*0.26*	–
**SDMT**			
**n**	301	319	278
**Mean (SD)**	6.2 (12.6)	6.6 (12.3)	5.1 (13.5)
p value[a] vs. GA 20 mg	*0.29*	*0.19*	–
**HR-QoL outcomes**			
**PRIMUS-Activities**			
**n**	261	281	230
**Mean (SD)**	-0.12 (4.2)	0.17 (4.9)	0.55 (4.1)
p value[b] vs. GA 20 mg	***0.008***	*0.53*	–
**MSIS-29**			
**Physical impact score**			
**n**	315	336	291
**Mean (SD)**	–3.5 (16.2)	–1.5 (15.3)	–0.8 (17.1)
p value[c] vs. GA 20 mg	***0.007***	*0.15*	–
**Psychological impact score**			
**n**	312	336	290
**Mean (SD)**	-6.6 (19.9)	-3.3 (19.8)	–2.2 (21.4)
p value[c] vs. GA 20 mg	***0.001***	*0.12*	–

[a]rank ANCOVA; [b]Wilcoxon rank sum test; [c]ANCOVA

GA, glatiramer acetate; MSFC, Multiple Sclerosis Functional Composite; MSIS-29, Multiple Sclerosis Impact Scale, PASAT, Paced Auditory Serial Addition Test; SD, standard deviation

© 2020 Cree BAC et al. *JAMA Neurology*.

eTable 2. Relapse Outcomes

	**Fingolimod 0.5 mg N=345**	**Fingolimod 0.25 mg N=366**	**GA 20 mg N=324**
**K-M estimates of relapse-free patients at Month 12, % (SE)**	86.8 (1.9)	83.8 (2.0)	80.2 (2.3)
p value (Log-rank test) vs. GA	0.03*	0.18	
**Risk reduction of confirmed relapse**	34%	18%	
Hazard ratio vs. GA 20 mg, (95% CI)	0.66 (0.45; 0.98)	0.82 (0.57; 1.19)	
Cox proportional hazards regression p value	0.04*	0.29	

CI, confidence interval; GA, glatiramer acetate; SE, standard error

* Indicates 2-sided statistical significance at 0.05 level

eTable 3. Negative Binomial Regression of Confirmed Relapses Up to 12 Months

	**Fingolimod 0.25 mg N=366**	**Fingolimod 0.5 mg N=345**	**GA 20 mg N=324**
Relapses	74	50	74
ARR	0.22	0.16	0.27
ARR (negative binomial)	0.22	0.15	0.26
95% CI	(0.16, 0.30)	(0.11, 0.22)	(0.19, 0.35)
Rate ratio	0.85	0.59	
95% CI	(0.56, 1.31)	(0.37, 0.95)	
p value	0.43	0.01	

CI, confidence interval; GA, glatiramer acetate

Pair-wise comparisons of treatment groups based on a negative binomial regression model adjusted for treatment, region, number of relapses experienced in the previous year, baseline EDSS, and baseline gadolinium-enhancing T1 lesion count, using time on study as offset. Random treatment-region interaction effect was included in the model (using PROC GLIMIXX).

© 2020 Cree BAC et al. *JAMA Neurology*.

eFigure. Study Design

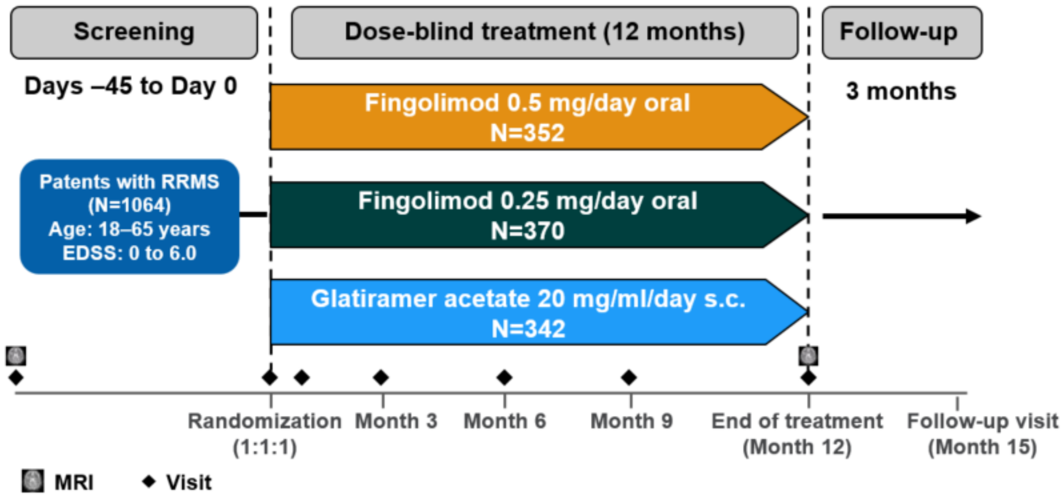

© 2020 Cree BAC et al. *JAMA Neurology*.

eReferences

1. Stokes ME, Davis CS, Koch GG (2000). Categorical data analysis using the SAS system. Second edition. SAS Institute, Inc., Cary, N.C.

© 2020 Cree BAC et al. *JAMA Neurology*.
