# Supplementary material for: Efficacy and Safety of 2 Fingolimod Doses vs Glatiramer Acetate for the Treatment of Patients With Relapsing-Remitting Multiple Sclerosis: A Randomized Clinical Trial
Source: JAMA Neurol. 2020 Aug 24;78(1):1–13. doi: 10.1001/jamaneurol.2020.2950 (PMC7445630; doi:10.1001/jamaneurol.2020.2950)
Supplement: Supplement 3. — Data Sharing Statement [file jamaneurol-e202950-s003.pdf]

Data Sharing Statement

Cree. Efficacy and Safety of 2 Fingolimod Doses vs Glatiramer Acetate for the Treatment of Patients With Relapsing-Remitting Multiple Sclerosis. *JAMA Neurol*. Published August 24, 2020. 10.1001/jamaneurol.2020.2950

Data
Data available: Yes
Data types: Deidentified participant data
How to access data: Anonymized data from the study are available on reasonable request from the authors, provided that it is in line with current ethical and intellectual property requirements surrounding the use of data. Requests should be directed to the corresponding author by email. Bruce.Cree@UCSF.edu
When available: With publication

Supporting Documents
Document types: None

Additional Information
Who can access the data: Anonymized data from the study are available on reasonable request from the authors, provided that it is in line with current ethical and intellectual property requirements surrounding the use of data. Requests should be directed to the corresponding author by email. Bruce.Cree@UCSF.edu
Types of analyses: Anonymized data from the study are available on reasonable request from the authors, provided that it is in line with current ethical and intellectual property requirements surrounding the use of data. Requests should be directed to the corresponding author by email. Bruce.Cree@UCSF.edu
Mechanisms of data availability: Anonymized data from the study are available on reasonable request from the authors, provided that it is in line with current ethical and intellectual property requirements surrounding the use of data. Requests should be directed to the corresponding author by email. Bruce.Cree@UCSF.edu
